# Discovery of the First Efficacious Adenosine 2A Receptor Negative Allosteric Modulators for High Adenosine Cancer Immunotherapies

**DOI:** 10.1021/acs.jmedchem.4c01691

**Published:** 2025-01-24

**Authors:** Margot Boujut, Margaux Héritier, Aurélie Gouiller, Camille Süess, Alessandro Scapozza, Thibaut De Smedt, Maxime Guibert, Sébastien Tardy, Hesham M. Ismail, David Pejoski, Leonardo Scapozza

**Affiliations:** † School of Pharmaceutical Sciences, University of Geneva, 1206 Geneva, Switzerland; ‡ Institute of Pharmaceutical Sciences of Western Switzerland, University of Geneva, 1206 Geneva, Switzerland; § Adoram Therapeutics, 1212 Grand-Lancy, Switzerland

## Abstract

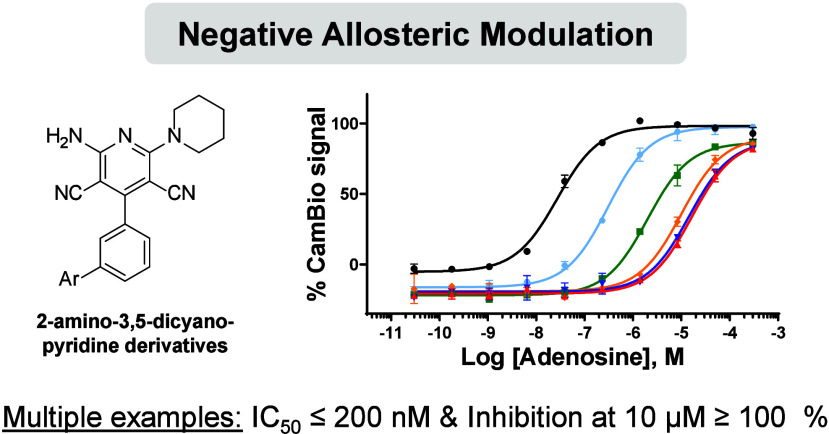

Inhibition of the adenosine 2A receptor (A_2A_R) is recognized as a promising immunotherapeutic strategy but is challenged by the ubiquity of A_2A_R function in the immune system. To develop a safe yet efficacious immunotherapy, the discovery of a novel negative allosteric modulator (NAM) was preferred. Leveraging an in-house, sensitive, high-throughput screening cellular assay, novel A_2A_R NAM scaffolds were identified, followed by an extensive structure–activity relationship (SAR) study, leading to the discovery of potent 2-amino-3,5-dicyanopyridine derivatives. The allosteric mode of action of active compounds was confirmed by progressive fold-shift assay, nonlinearity of the Schild plot analysis, biophysical measurements, and retained satisfactory potencies in high-adenosine concentrations. Further correlation of A_2A_R engagement and downstream signaling was done in a human blood translational assay, clearly showcasing the potential of A_2A_R allosteric modulation as a novel approach for efficient and safer cancer immunotherapies.

## Introduction

For almost two decades now, the modulation of adenosine receptors has been recognized as a promising therapeutic strategy for many diseases and disorders including, but not limited to, neurological, cardiovascular, renal, intestinal, inflammatory, and pulmonary conditions.^1−4^ This especially broad list is due to the many regulatory mechanisms involving adenosine and the adenosine receptors throughout the body. The adenosine receptor family is part of the G-protein-coupled receptor superfamily and is composed of four receptor subtypes: A_1_R and A_3_R, which are G_i_-coupled receptors, and A_2A_R and A_2B_R, which are G_s_-coupled receptors.^5^ The adenosine 2A receptor (A_2A_R), for example, is found both in the central nervous system and in the periphery, mainly in the immune system (spleen, thymus, leucocytes, and blood platelets) and at intermediate levels in the heart, blood vessels, and lungs.^6^ This ubiquity of the adenosine receptors poses a challenge to the development of safe and potent therapies.^1,3^

Adenosine exerts a cytoprotective effect by engaging A_2A_R in the immune system, resulting in an anti-inflammatory and pro-resolving response.^7^ However, accumulation of extracellular adenosine is one of the widespread immunosuppressive mechanisms by which antitumor immunity is reduced. High adenosine in the tumor microenvironment (TME) indeed correlates with tumor aggressiveness.^8^ It was of interest to discover and optimize safe ligands that are able to outcompete high adenosine concentrations.

**Figure 1:**
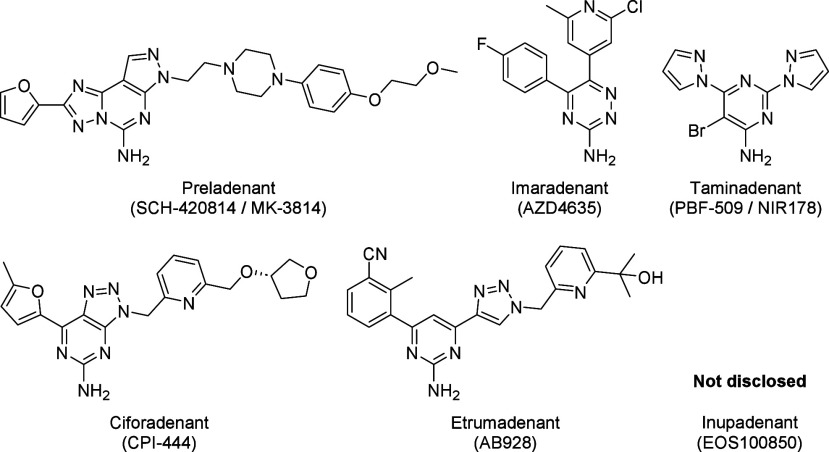
Clinical candidates for cancer immunotherapies by A_2A_R orthosteric inhibitors.

The first A_2A_R orthosteric ligands, such as preladenant, imaradenant, taminadenant or ciforadenant (Figure 1), were developed for or derived from treatments for Parkinson’s disease where adenosine concentrations are lower than in the TME.^9^ When repurposed as cancer immunotherapies, they resulted in low response rates and/or safety concerns due to high dosing, leading to the discontinuation of most clinical trials. Exciting progress was made in the more recent development of these cancer immunotherapies by combination of immune checkpoint inhibitors and A_2A_R antagonists developed to withstand high adenosine concentrations, such as etrumadenant and inupadenant (Figure 1).^10^

One important consideration in the clinical use of A_2A_R antagonists is the safety of the treatments due to the ubiquity of adenosine signaling in physiological processes.^4,7^ Negative allosteric modulators (NAMs) represent an attractive type of ligand to overcome this challenge. NAMs are characterized by their allosteric probe-dependent and saturable mode of action. Negative allosteric modulation is characterized by decreasing the affinity and/or the efficacy of orthosteric ligands, including the natural ligand(s) of a receptor, which has a saturable effect once all allosteric sites are occupied. This saturable effect of NAMs allows them to work at the minimum efficacious dosing of a treatment, as increased dosage does not result in increased therapeutic effect, limiting the risk of off-target side effects.^11,12^ Off-target side effects are also expected to be lowered in the case of allosteric ligands as allosteric pockets are postulated to be less conserved due to their remote positioning with respect to the orthosteric binding site; hence it should be possible to achieve higher receptor selectivity.^12,13^

Despite the now large body of literature surrounding the A_2A_R protein structure^14^ (76 structures available in the RCSB protein data bank as of October fourth, 2023), only the sodium binding site has been well identified and characterized as an allosteric pocket.^15,16^ Different approaches have been employed to try and propose allosteric sites for A_2A_R,^17^ but to this day, experimental data are lacking to validate these hypothesis. This lack of rationale might explain the very small number of known A_2A_R allosteric modulators in comparison to the intense development of its orthosteric ligands. Few positive allosteric modulators have been reported to improve CGS21680 (a specific agonist) A_2A_-mediated pharmacological effects in *ex vivo* experiments,^18^ to attenuate inflammation in preclinical models of psoriasis-like dermatitis,^19,20^ or to be able to induce slow-sleep wave in mania and schizophrenia-like behaving mice.^21,22^ Advances on A_2A_R negative allosteric modulators are more scarce in the literature (Supporting Information Figure S1); amiloride and its derivatives (nonselective A_2A_R binders) were shown to accelerate the dissociation rate of radiolabeled ZM241385, most likely favoring a closed, inactive conformation of A_2A_R. This binding was outcompeted by sodium addition in a dose-dependent manner, hinting that amiloride and sodium act through the same allosteric pocket.^23^ Fragments acting as A_2A_R NAMs, such as Fr754, have been described more recently by Lu et al. using a novel affinity mass spectrometry technique and suggesting a different binding pocket.^24^ However, no further developments have been published.

**Figure 2:**
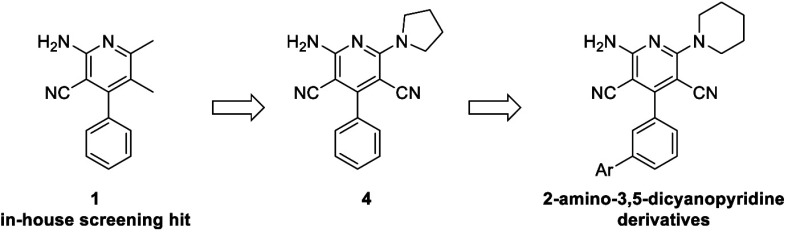
From hit compound **1** to 2-amino-3,4-dicyanopyiridine derivatives.

In this work, our in-house dynamic cAMP measurement assay (CamBio assay) was used in a high-throughput screening campaign to discover A_2A_R antagonist scaffolds and novel A_2A_R NAMs. To distinguish between orthosteric inhibitors (and inverse agonists) and fully allosteric hits, these scaffolds were further characterized by progressive fold-shift assays.^12^ This method resulted in the discovery of compound **1**, which demonstrated a desirable NAM profile. We presently describe the first extensive structure–activity relationship (SAR) campaign for A_2A_R allosteric modulators following the discovery of compound **1**, which led to the discovery of potent and A_2A_R-selective 2-amino-3,5-dicyanopyridine derivatives (Figure 2). Rigorous experimentation was then used to validate the allosteric mode of action of the compounds and to assess their receptor subtype selectivity. A proof-of-concept of compound activity could be observed in human translational assays, clearly showcasing the potential of these compounds as novel immunotherapies for high adenosine cancers.

## Results and Discussion

### Synthetic Routes

**Scheme 1:**
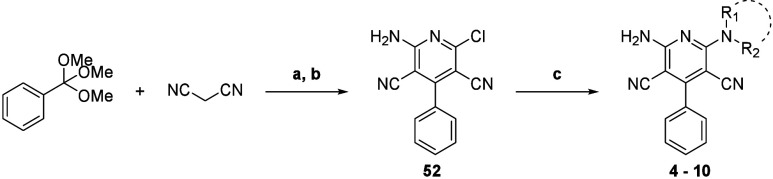
Synthesis of Compounds **4**–**10** (a) Pyridine, 100 °C, argon, 1 h; (b) HCl 37%, 0 °C, 5 min then 80 °C, 2 h, 18% isolated yield over two steps; (c) NHR_1_R_2_, THF/EtOH (3:1), 120 °C, microwave irradiation, 30 min, 13%–19% isolated yields.

Compounds **4**–**10** were first synthesized via a 2-step process^25^ (Scheme 1). Trimethyl orthobenzoate and 2 equiv of malononitrile were first heated in pyridine and then subjected to strong acidic conditions to give compound **52**. This chloropyridine intermediate **52** can be substituted by an excess of primary or secondary amine under microwave irradiation to yield the desired compounds **4**–**10**.

**Scheme 2:**
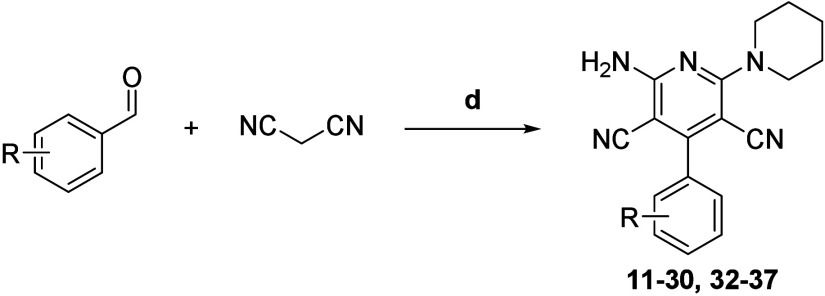
Synthesis of Compounds **11**–**30** and **32**–**37** (d) Piperidine, 4-DMAP, MeOH, 0 °C to rt, air, 16 h, 7%–30% isolated yields.

**Scheme 3:**
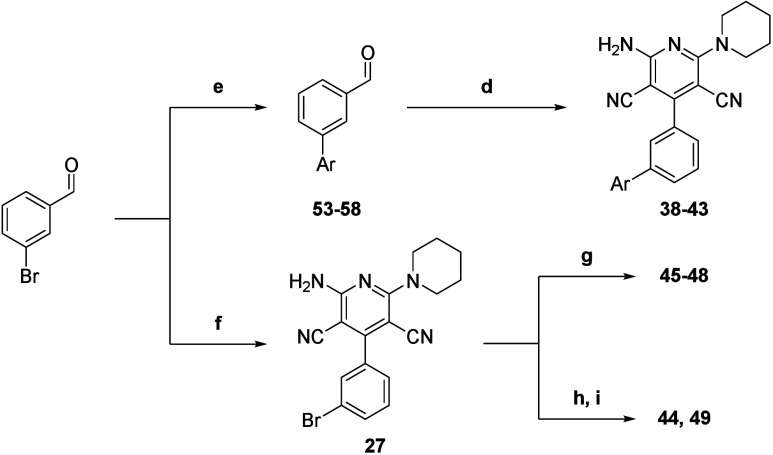
Synthesis of Compounds **38**–**49** (e) Boronic acid, K_2_CO_3,_ PdCl_2_, toluene, 110 °C, air, 24 h, 15%–77% isolated yield; (f) malononitrile, piperidine, MeOH, rt, air, 16 h, 20% isolated yield; (g) boronic acid, K_2_CO_3_, Pd(PPh_3_)_4_, THF/H_2_O (2:1), 100 °C, microwave irradiation, 15–30 min, argon, 15%–77% isolated yield; (h) bis(pinacolato)diboron, K_2_CO_3_, Pd(PPh_3_)_4_, toluene, 110 °C, argon, 16 h, 59% isolated yield; (i) bromoaryl, K_2_CO_3_, Pd(PPh_3_)_4_, THF/H_2_O (2:1), 100 °C, microwave irradiation, 15–30 min, argon, 15%–77% isolated yield.

The general one-pot cyclization used for the obtention of compounds **11**–**30** and **32**–**37** is presented in Scheme 2; compounds **38**–**43** presented in Scheme 3 were also obtained via this procedure. This protocol has been adapted from Sarkar et al.^26^ with moderate success; however, it happened to be quite tolerant to various chemical functionality and highly reproducible.

Starting from 3-bromobenzaldehyde, various bis aryl derivatives could be isolated after a Suzuki–Miyaura cross-coupling reaction in the presence of substituted phenylboronic acids (Scheme 3). These intermediate aldehydes **53**–**58** could be subjected to the general procedure presented above to obtain compounds **38**–**43** in moderate to good yields. Alternatively, 3-bromobenzaldehyde can directly be cyclized to compound **27** and used as a synthetic intermediate.

A gram-scale protocol can be achieved in a semisequential manner, in the absence of the catalytic 4-DMAP and by using a slight excess of reactive piperidine instead. Compound **27** was used as a starting aryl bromide in Suzuki–Miyaura cross-coupling activated by microwave irradiation to access other desired compounds **45**–**48**. For more reluctant substrates or noncommercial boronic acids, the boronic pinacol ester could be directly installed on compound **27** under traditional heating and reacted with commercial bromoaryls in similar microwave-activated fashion to yield compounds **44** and **49** (Scheme 3).

**Scheme 4:**
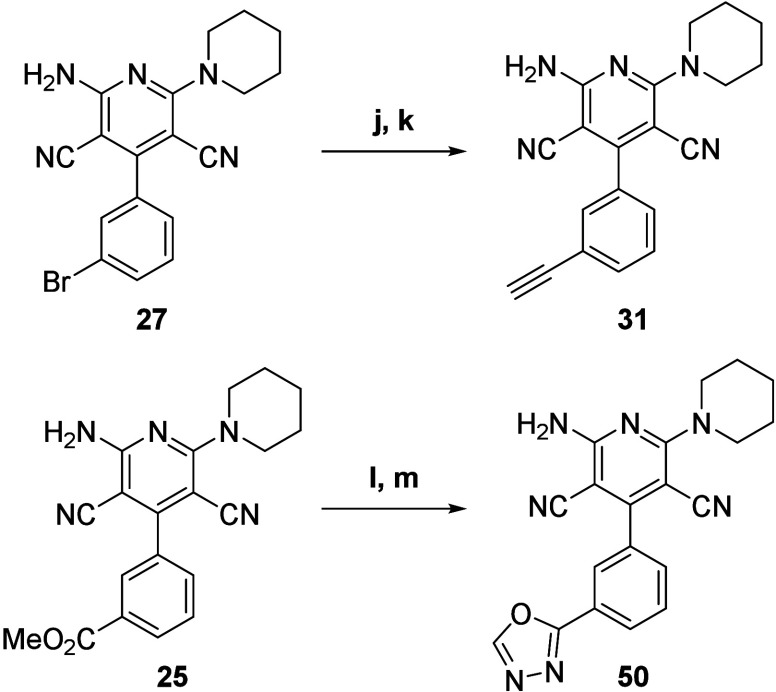
Synthesis of Compounds **31** and **50** (j) TIPS-acetylene, NH(nPr)_2_, CuI, Pd(dppf)Cl_2_, THF, 80 °C, argon, 18 h,, 52% isolated yield; (k) TBAF, THF, 0 °C, 15 min, quantitative isolated yield; (l) N_2_H_4_·H_2_O, MeOH, 80 °C, argon, 16 h; then (m) CH(OEt)_3_, argon, 100 °C, 20 h, 58% isolated yield over two steps.

The intermediate **27** could also be used in other types of metal cross-couplings like Sonogashira copper–palladium coupling to obtain compound **31** after deprotection of the triisopropylsilyl terminal alkyne with a TBAF solution. Other late-stage intermediates could be employed for further functionalization like compound **25** which was used for the cyclization of an oxadiazole in two steps for the synthesis of compound **50** (Scheme 4).

**Scheme 5:**
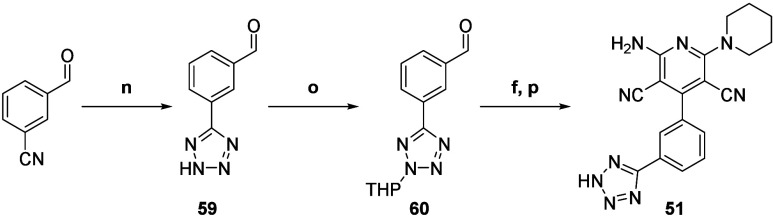
Synthesis of Compound **51** (n) NaN_3_, ZnCl_2_, H_2_O/DMF (99:1), 110 °C, argon, 48 h, 42% isolated yield; (o) 3,4-dihydro-2*H*-pyrane, TFA, toluene, 110 °C, argon, 24 h, 26% isolated yield; (f) malononitrile, piperidine, MeOH, rt, air, 16 h; then (p) Dowex 50WX8 H^+^, EtOH/H_2_O (98:2), 80 °C, 16 h, 11% isolated yield over two steps.

Finally, some protection/deprotection strategies had to be applied for more reactive derivatives such as the tetrazole **51** which was first built from the 3-formylbenzonitrile to the aldehyde **59** (Scheme 5). After THP protection, aldehyde **60** could be further cyclized to the 2-amino-3,5-dicyanopyridine core and deprotected by treatment with a slightly acidic resin to desired compound **51** (Scheme 5).

### High Throughput Screening Assay Identified Novel NAM Scaffolds

Though A_2A_R is a well-studied target, the ligands described in the literature are mostly orthosteric inhibitors. To free ourselves from this orthosteric mode of action, novel hits must be discovered. The strategy of biological assay development followed by an in house high-throughput screening campaign was in line with this need for original scaffolds.

In short, our cAMP measuring (“CamBio”) assay is a high-throughput screening assay, selective, sensitive, and reproducible, developed for allosteric modulator detection. The assay allows the direct, real-time monitoring of intracellular cAMP concentration thanks to a BRET-based biosensor containing a cAMP binding domain coexpressed with A_2A_R, or any GPCR, in CHO cells. Moreover, its BRET-based biosensor enables dynamic measurement of both G_s_- and G_i_-coupled receptor activation without artificially modifying their coupling mechanism, which is key for allosteric modulator identification. High-throughput screening capabilities are achieved by monitoring using an FDSS/μCELL platform.

For the hit identification campaign, an in-house, manually curated library of 2440 compounds (representative of 6 million of commercially available scaffolds spanning over a large spectrum of biologically relevant targets)^27^ was screened. Testing compounds using a dose of adenosine corresponding to the concentration at which 80% of the receptor is activated (EC_80_, [adenosine] = 400 nM) allowed us to identify novel A_2A_R-antagonizing scaffolds (data not shown). The hits were chosen based on their activity in the CamBio assay at 10 μM and then were confirmed by a dose response curve; finally their mode of action was tested on the same cell line for the distinction between orthosteric inhibitors and allosteric modulators by highlighting the noncompetitive binding mode of the latter; this is materialized by a stacking of the dose response curves of adenosine in the presence of various compound concentrations in a progressive fold-shift assay (more details in the following sections). Only the resulting 6 structurally diverse NAMs were labeled as hits.

**Figure 3:**
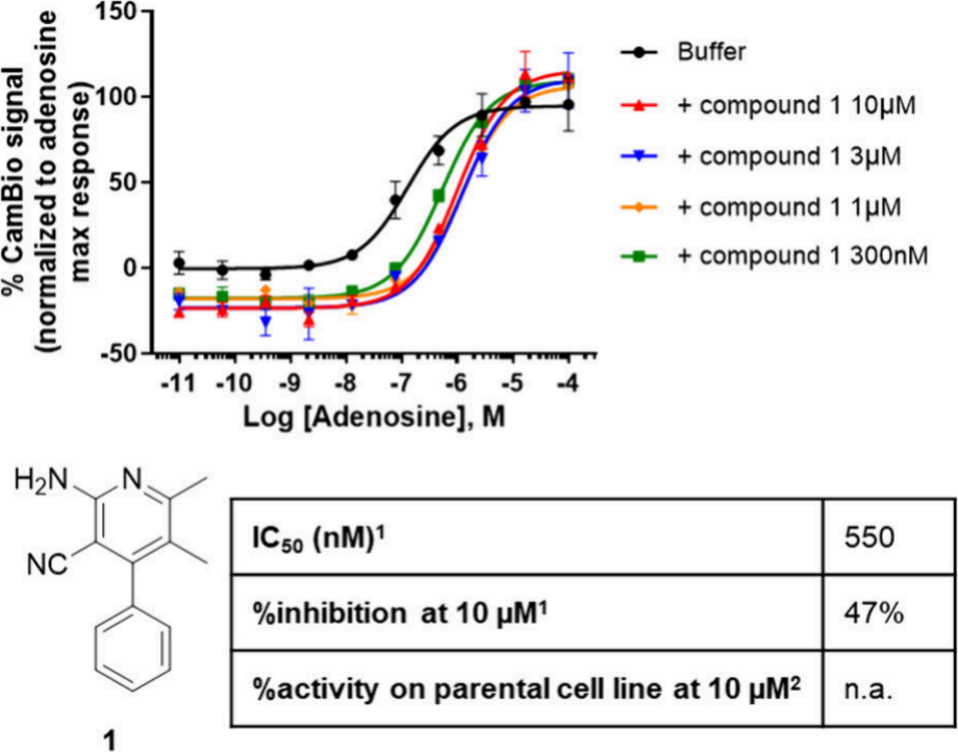
Structure, activity, and confirmation of the allosteric profile by progressive fold-shift assay of hit compound **1**. ^1^IC_50_ (nM) in the presence of 400 nM adenosine and % inhibition at 10 μM values are given by CamBio assay on CHO cells expressing A_2A_R; experimental repetitions *N* = 2–5. ^2^Specificity is given by CamBio assay on the parental CHO cell; experimental repetitions *N* = 2–3. n.a. = non active.

All hit compounds already had hit-like physiochemical properties by design of the library (Lipinski rule of 5 and chemical tractability). Compound **1** was selected over other hits due to its promising IC_50_ of 0.55 μM and its specificity for the adenosine receptor pathway (i.e., nonactive on the parental cell line). However, compound **1** exhibited only partial inhibition of the receptor (47% at 10 μM compound; Figure 3) despite its low IC_50_. Whether this partial inhibition of the system was due to the compound’s partial activity or inherent to its allosteric mode of action could only be solved later in the medicinal chemistry campaign. This effect could be expected since the relationship between target occupancy and an allosteric antagonist’s response is nonlinear.^28^ Compound **1** in the progressive fold-shift assay demonstrated the expected curve stacking pattern for NAMs; thus it was labeled as one of the initial hits.

### SAR Exploration

**Figure 4:**
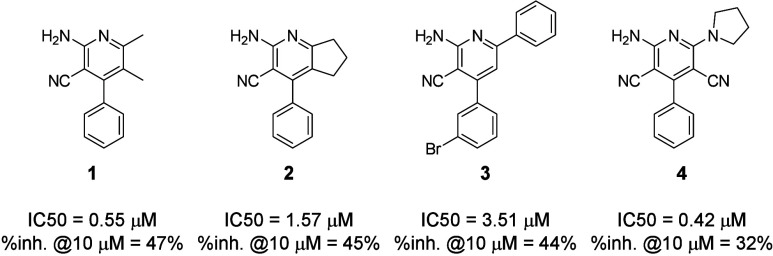
Structure and biological effects on A_2A_R of compounds **1**–**4** resulting from a rapid scaffold hopping from commercially available analogs.

Using compound **1** as our starting point, we first evaluated the biological effect on A_2A_R of several compounds from commercially available libraries using our CamBio assay in the presence of an EC_80_ of adenosine, as reported in Figure 4. All compounds demonstrated moderately good activity with IC_50_ in the 10^–7^–10^–6^ M range and similar percentage of inhibition at 10 μM of compound (%inh. @10 μM). Formation of a bicyclic system in compound **2** resulted in a 3-fold loss of potency. While two modifications were introduced in compound **3** and direct comparison cannot be made, it demonstrated that both aromatic substitution and larger substituents on position 6 of the pyridine could be tolerated to a certain extent. Still, compound **3**’s overly aromatic nature prevented it from being a good starting block and finally, compound **4** was retained for its good solubility as predicted by Chemicalize (https://chemicalize.com/welcome), its lower IC_50_ for the same percentage of inhibition compared to the hit, its maintained allosteric profile (see Supporting Information), its most favorable chemical tractability, and its novelty necessary for filing intellectual property.

As it was the largest point of modification between the initial scaffolds, the influence of cyclic amine substitution was first explored. The nature of the amine, ring size, and ring substituents were varied (Table 1). Going from a 5- to a 6-membered ring proved beneficial with a 2-fold improvement of the IC_50_ (**4**, IC_50_ = 420 nM; **5**, IC_50_ = 220 nM; Table 1) and a satisfactory increase of the % inhibition (**4**, %inh. @10 μM = 32%; **5**, %inh. @10 μM = 70%). Furthermore, ring enlargement resulted in only a drop of activity for compound **6**, most likely due to the overly increased steric hindrance. Similar observations can be made if the nature of the disubstituted amine goes from a better constrained cyclic structure to flexible branched amines (**5** vs **7** and **8**). Hence, the cyclic restriction was kept, and substitutions of the piperidine moiety were tested to try increasing either its hydrophobicity or its hydrophilicity. This only resulted in a loss in A_2A_R activity, 12- and 6-fold for compounds **9** and **10** respectively. This further highlighted the likely limited space and hydrophobic nature of the pocket that accommodates this part of the molecule.

**Table 1:**
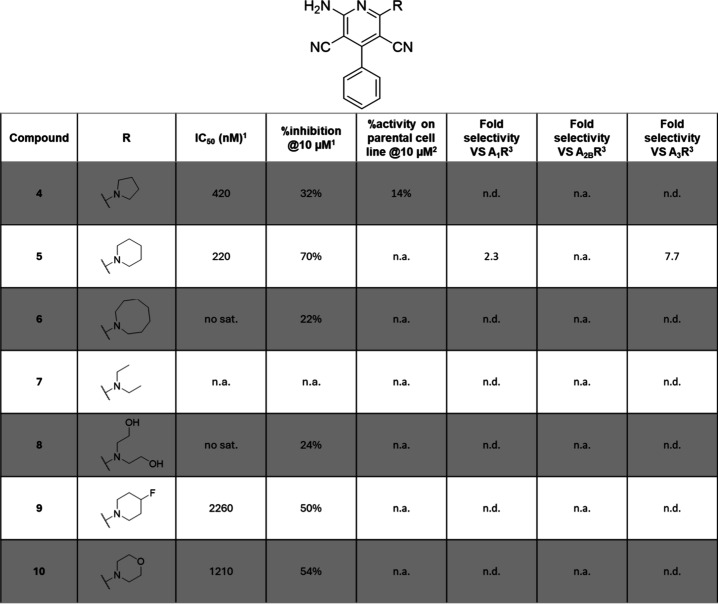
Biological Effects of Compounds **4**–**10**^a^

a n.a. = non active; no sat. = no saturation of the dose response curve was reached, and IC_50_ cannot be calculated; n.d. = not determined.

1 IC_50_ (nM) in the presence of 400 nM adenosine and % inhibition at 10 μM values are given by CamBio assay on CHO cells expressing A_2A_R; experimental repetitions *N* = 2–5.

2 The % activity in CamBio assay on the parental CHO cells is indicative of the specificity; experimental repetitions *N* = 2–3.

3 Fold selectivity is given as a ratio of the IC_50_ measured by CamBio assay on CHO cells expressing the corresponding receptor to the IC_50_ measured by CamBio assay on CHO cells expressing A_2A_R; experimental repetitions *N* = 2–3; selectivity based on the ratios: 0 to <2 = none; 2 to <5 = poor, 5 to <15 = agreeable, ≥15 = good.

The specificity was assessed by our CamBio assay on the parental CHO cell line. To account for the variability of the CamBio assay, we applied the cutoff of 20% to define the specificity: compounds with a percentage of activity lower than 20% were considered specific, which is the case of all compounds **4** to **10**.

Interestingly, most of the tested scaffolds presented a good selectivity profile compared to the closest isoform A_2B_R; additionally, compound **5** activity on A_3_R was judged as a good starting point while A_1_R selectivity remained to be improved.

As demonstrated by the early explorations, substituents seemed to be tolerated around the phenyl in position 4 of the pyridine core and called for further inquiries (compound **3**, Figure 4). Still, direct comparison of compounds **3** and **27** was eventually possible and reenforced our choice of the current core as a better and more potent structure (IC_50_ = 3.51 and 1.04 μM, respectively). All biological effects for mono- and disubstitution patterns are recapitulated in Table 2 and Table 3, respectively.

**Table 2:**
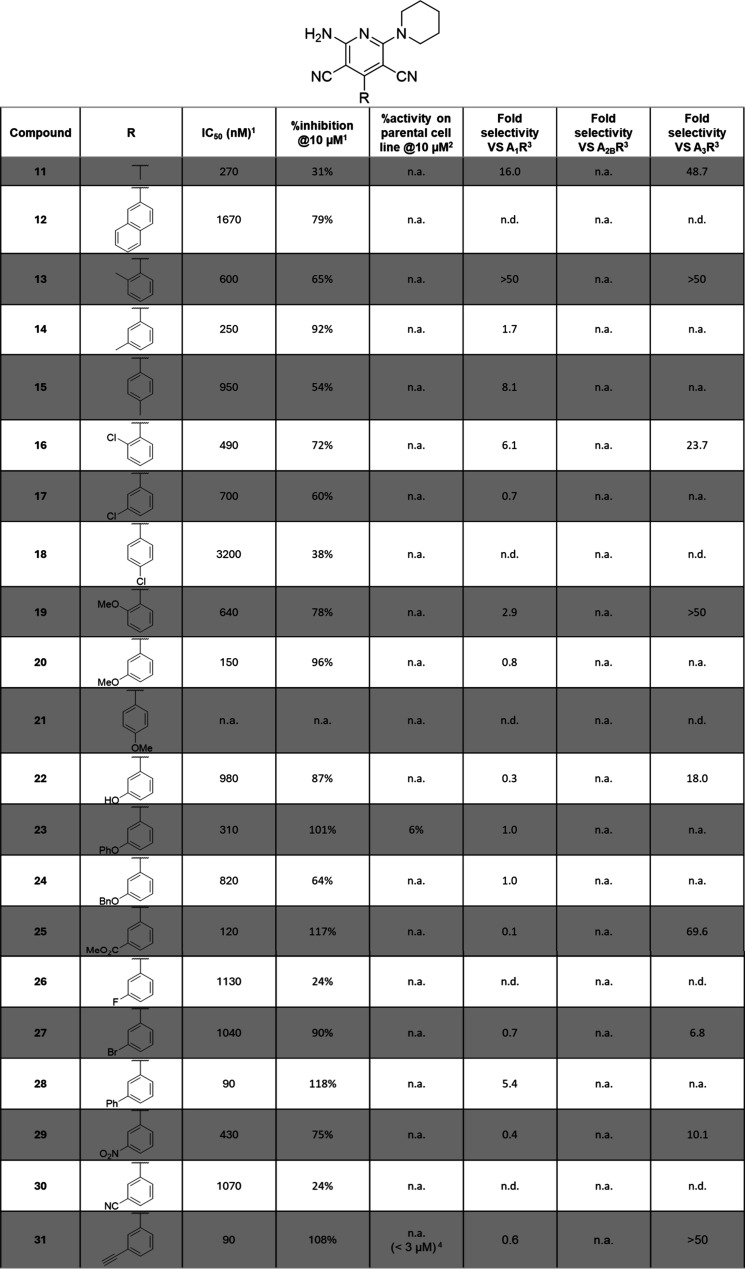
Biological Effects of Compounds **11**–**31**^a^

a n.a. = non active; no sat. = no saturation of the dose response curve was reached, and IC_50_ cannot be calculated; n.d. = not determined.

1 IC_50_ (nM) in the presence of 400 nM adenosine and % inhibition at 10 μM values are given by CamBio assay on CHO cells expressing A_2A_R; experimental repetitions *N* = 2–5.

2 % activity in the CamBio assay on the parental CHO cells is indicative of the specificity; experimental repetitions *N* = 2–3.

3 Fold selectivity is given as a ratio of the IC_50_ measured by CamBio assay on CHO cells expressing the corresponding receptor to the IC_50_ measured by CamBio assay on CHO cells expressing A_2A_R; experimental repetitions *N* = 2–3; selectivity based on the ratios: 0 to <2 = none, 2 to <5 = poor, 5 to <15 = agreeable, ≥15 = good.

4 Specificity detected for concentrations lower than the concentration indicated in brackets.

**Table 3:**
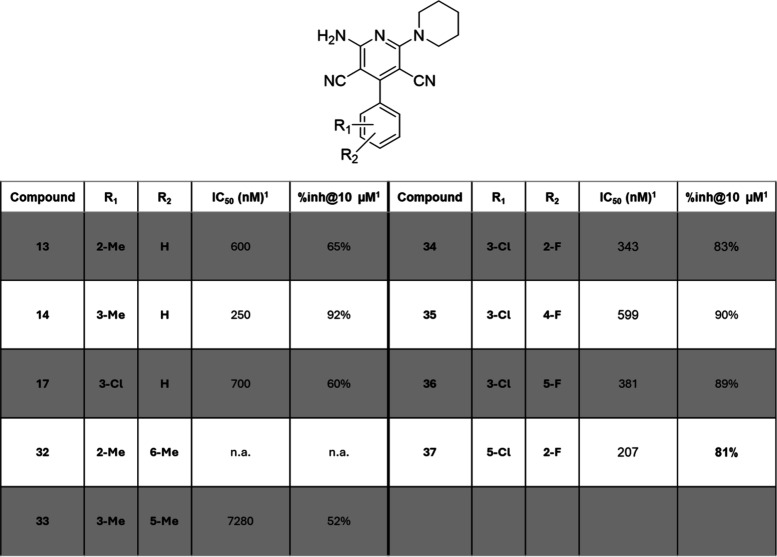
Biological Effects of Monosubstituted Compounds (**13**, **14**, and **17**) and Disubstituted Compounds (**32**–**37**)

1 IC_50_ (nM) in presence of 400 nM adenosine and % inhibition at 10 μM values are given by CamBio assay on CHO cells expressing A_2A_R; experimental repetitions *N* = 2–5.

As the biological effect of the aromatic depletion in compound **11** was comparable to the parent hit compound **4** the aromatic core in position 4 was maintained (Table 2). Larger groups such as naphthyl (compound **12**) and para substituents (compounds **15**, **18**, and **21**) resulted in drops of 10-fold or more in IC_50_, up to a complete loss of activity (compound **21**). Ortho substitution of the ring was well tolerated and even favored for moderately electron-withdrawing atoms such as chlorine (compound **16**), but meta substitutions yielded the best results, achieving almost full inhibition of the system with the lowest IC_50_ when compared in the same series (compounds **14** vs **13** and **15**, **20** vs **19** and **21**). For this reason, the following efforts were made on the nature of substitutions in the meta position. Medium to strong electron withdrawing groups tended to reduce to both the IC_50_ and maximum potency of the compounds (compounds **26**, **27**, and **30**), but this effect appeared to be countered by the presence of hydrogen bond acceptors (compounds **25** and **29**). Surprisingly, larger and more rigid substituents were tolerated for this meta exit vector, with improved IC_50_ in the low nanomolar range and the desired full inhibition of A_2A_R activity (**28**, IC_50_ = 90 nM and %inh. @ 10 μM = 118%; **31**, IC_50_ = 90 nM and %inh. @ 10 μM = 108%), resulting in the characterization of the first optimized A_2A_R NAMs.

Most of these compounds were also tested for specific and selective activity and, while A_2B_R and A_3_R selectivity profiles remained favorable, meta substituents presented only little selectivity against A_1_R until a larger phenyl group was introduced on compound **28**.

Having improved receptor subtype selectivity and being encouraged by the current research surrounding A_2A_R orthosteric ligands, we believed that better potencies could be achieved with our NAMs and decided to investigate further this versatile aromatic substitution. Biological activities of some disubstitution patterns are presented in Table 3.

Dimethyl substitution patterns of compounds **32** and **33** turned out to be largely disfavored compared to the monosubstituted analog (**32** and **33** vs **13** and **14**); hence our focus shifted to the introduction of single atom differences. As often encountered in a medicinal chemistry campaign,^29^ the change of a hydrogen to a fluorine, or “fluorine walk”, was not benign and influenced both the IC_50_ and % inhibition of the synthetic precursor **17**, with variable results depending on the fluorine position. Interestingly, the presence of an additional fluorine atom positively influenced the compounds’ activities, with IC_50_ cut in half compared to parent compound **17**, for all except compound **35** (**34**, IC_50_ = 343 nM, **36**, IC_50_ = 381 nM, **37**, IC_50_ = 207 nM vs **17**, IC_50_ = 700 nM) and % inhibition at 10 μM increased by 1.5-fold, up to almost full inhibition of the system. These results could be combined with the optimized monosubstitution for the future developments of the lead compounds.

All of the previous results combined suggested that ortho substitutions are suboptimal. This could arise from unfavorable interactions with the cyano group, which locked the compound in a less bioactive conformation. Another potential explanation is the existence of a hydrophobic groove/pocket for which the meta position of a phenyl ring creates an ideal orientation. Bisphenyl substitution appeared to be quite potent and offered a solid platform to start exploring further this new space (Table 4). Small substituents of a hydrophobic nature, either electron withdrawing or donating, were well accommodated in both ortho and meta positions (compounds **38**, **39**, **41**, and **42**; IC_50_ = 160–290 nM and above full % inhibition); however, para substituents had more than 10-fold losses in IC_50_ (compounds **40** and **43**). Introduction of soft sulfur atoms or various harder heteroatoms in five-membered rings did not strongly influence the inhibitory profile either (compounds **47**–**51**). The use of a hydrogen bond donor did not bring anything more to the system (compound **49**) and was even unfavored in the case of a strongly acidic proton (tetrazole in compound **51**). On the other hand, when the hydrogen bond donor capabilities of the protein were explored by a simple nitrogen ring road, ortho and meta pyridines were largely favored (**44** and **45**; respectively, 6-fold and 10-fold IC_50_ improvements compared to compound **28**), yielding compounds with IC_50_ in the targeted low nanomolar range. The allosteric mode-of-action of some selected compounds was confirmed once more by progressive fold-shift assay, assuring that the whole family was indeed maintaining its allosteric characteristics (see Supporting Information). Addition of this bisphenyl substitution conferred a good selectivity profile over A_2B_R and A_3_R overall while A_1_R selectivity profile appeared more unpredictable. Still, the activity and selectivity profiles on cells of compounds **28** and **44**–**48** were recognized as good, and they were pushed for further testing.

**Table 4:**
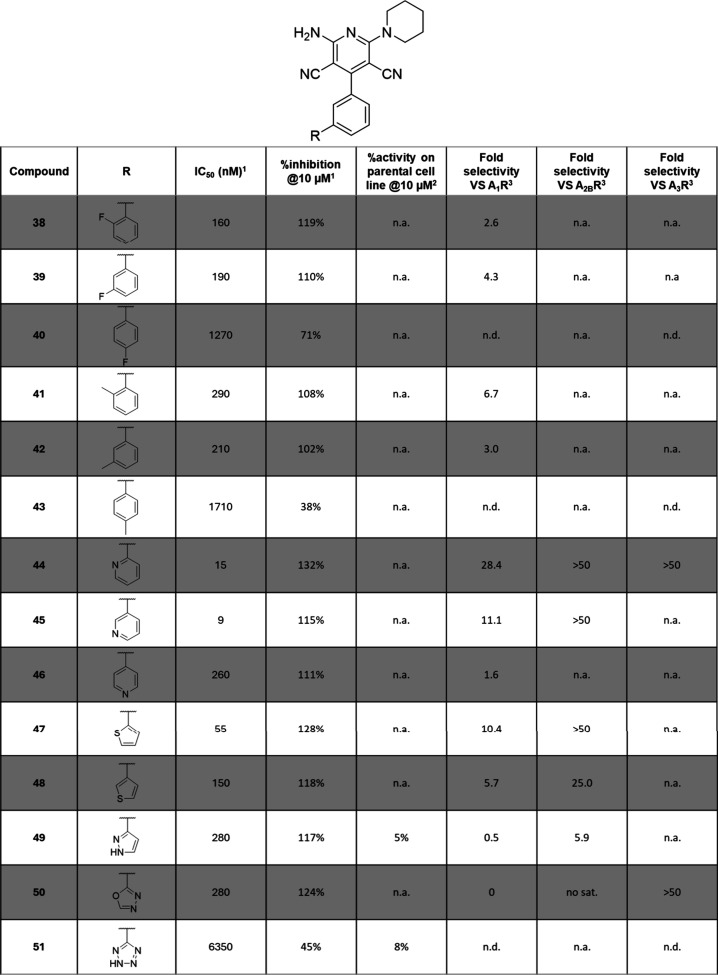
Biological Effects of Compounds **38**–**51**^a^

a n.a. = non active; no sat. = no saturation of the dose response curve was reached, and IC_50_ cannot be calculated; n.d. = not determined.

1 IC_50_ (nM) in presence of 400 nM of adenosine and % inhibition at 10 μM values are given by CamBio assay on CHO cells expressing A_2A_R; experimental repetitions *N* = 2–5.

2 % activity in the CamBio assay on the parental CHO cells is indicative of the specificity; experimental repetitions *N* = 2–3.

3 Fold selectivity is given as a ratio of the IC_50_ measured by CamBio assay on CHO cells expressing the corresponding receptor to the IC_50_ measured by CamBio assay on CHO cells expressing A_2A_R; experimental repetitions *N* = 2–3; selectivity based on the ratios: 0 to <2 = none, 2 to <5 = poor, 5 to <15 = agreeable, ≥15 = good.

### Compounds **4** and **5** Engage A_2A_R in an Allosteric Manner

As this discovery program focused on the development of allosteric modulators, extra care was taken to identify the compounds’ mode of action. By definition, allosteric modulators will exert an effect only in the presence of orthosteric ligands and reach a saturable effect once the system is fully occupied. In the case of negative allosteric modulators, probe-dependency is harder to demonstrate, as protein activity will be lowered in cellular assays whether binding is competitive (i.e., orthosteric) or not, hampering many of the NAM discovery programs. However, allosteric modulators exert an effect on ligand binding cooperativity: they affect ligand binding kinetics by affecting the protein’s conformation.

**Figure 5:**
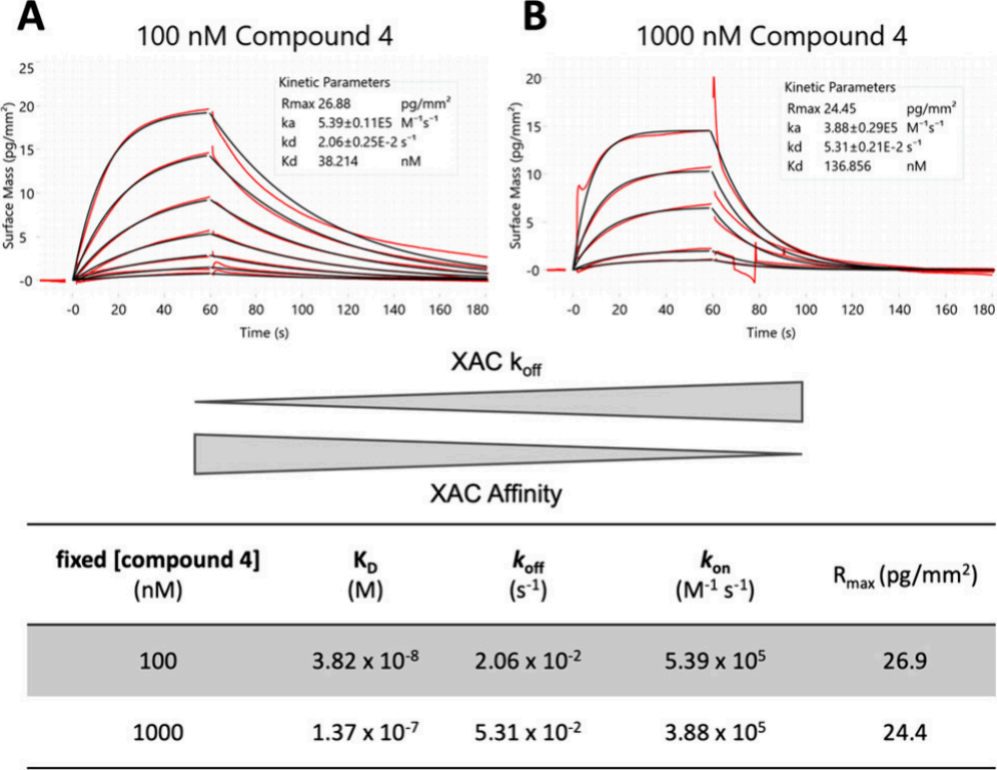
GCI kinetic characterization of XAC binding to A_2A_R at different concentrations of compound **4**: (A) 100 nM and (B) 1000 nM. Double-referenced binding signals are shown in red; 1:1 Langmuir interaction model fits are shown in black. Kinetic parameters in the table were derived from fitting double-referenced binding signals to a 1:1 interaction model at two concentrations of compound **4**.

Grating-coupled interferometry (GCI) is a sensitive, label-free method to characterize ligand binding to membrane proteins immobilized on a surface. Dissociation constants (*K*_D_) and kinetic rates (*k*_on_, *k*_off_) of XAC, a xanthine-based A_2A_R orthosteric antagonist (Supporting Information Figure S2),^30,31^ binding to A_2A_R were measured at increasing concentrations of compound **4** present throughout the measurement from the baseline to XAC dissociation. Notably, compound **4** was able to negatively modulate XAC affinity toward A_2A_R, with an approximately 4-fold increase in *K*_D_ (Figure 5 and Supporting Information Figure S3 for compound **5**). Both, association and dissociation rates contribute to the difference in affinity, showing a decrease in *k*_on_ as well as an increase in *k*_off_ (shorter residence time) at higher concentrations of compound **4**. Furthermore, the calculated binding signal at XAC saturation (*R*_max_) remained at the same level, which indicates the absence of any binding competition and therefore independent, allosteric interaction of compound **4** with A_2A_R. Together, these findings support a mode of action of compound **4** as a NAM.

### The Allosteric Mode of Action Is Conserved Throughout the Chemical Family: From Compound **5** to **28** to **45**

**Figure 6:**
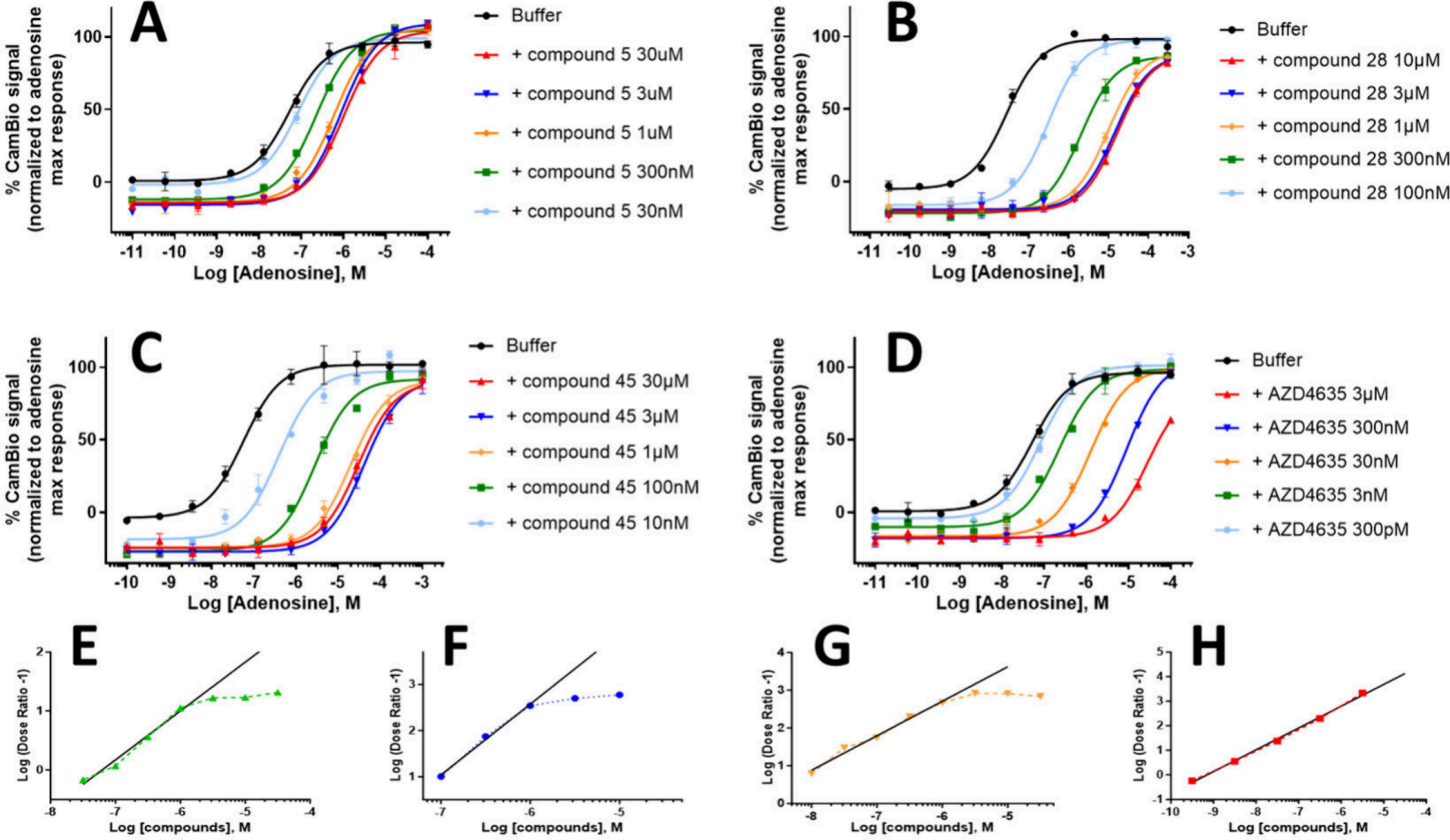
Respective progressive fold-shift assays and Schild plot analysis for compounds **5** (A, E), **28** (B, F), and **45** (C, G) and imaradenant (D, H). The curve stacking in the progressive fold-shift assay confirms an allosteric compound mode of action.

The allosteric mode of action can further be supported by demonstrating the non competitive binding of the NAMs and the saturable antagonism effect of the system through binding at an allosteric site. Saturation can be shown in a cellular assay by doing several dose response curves of an agonist with fixed concentrations of the tested compound, termed a progressive fold-shift assay. Results for selected examples and imaradenant (AZD4635), an orthosteric antagonist, are presented in Figure 6. The curve stacking pattern observed for the compounds **5**, **28**, and **45**, Figure 6A–C (and not for imaradenant, Figure 6D, or compounds in Supporting Information Table S1), is characteristic of their allosteric mode of action^12,32^ and is conserved across the chemical family and more specifically compounds presented in Table 4 and Supporting Information Table S2.

These progressive fold-shift assay results can be exploited in a Schild plot analysis (regression of log(Dose Ratio – 1) versus log[compound]) to quantify compounds’ allosteric effect.^12,32^ Maximum NAM antagonist activity is expected to occur when all of the allosteric sites are occupied by allosteric modulators in the presence of orthosteric agonists. Binding of both ligands being noncompetitive, the corresponding Schild plot will present nonlinear proportions. This is indeed observed for NAMs **5**, **28**, and **45** (Figure 6E–G), highlighted in contrast to the linear dependency of the log[compound] with the log(Dose Ratio – 1) for orthosteric ligands (Figure 6H).

Allosteric modulators can exert an independent effect on both the binding kinetics and the operational efficacy of a protein–ligand complex, which are reflected in the binding cooperativity factor α and the binding efficacy factor β. GCI measurements already showed that these NAMs acts on protein cooperativity, but the lack of variation in the maximum response of adenosine in the progressive fold-shift assays demonstrates that they act solely on the A_2A_R–ligand complex formation (Figure 6A–C). Hence, their Schild plots follow equation 1, from which numeric values such as the binding cooperativity factor α and the equilibrium dissociation constant of the modulator-receptor complex *K*_B_ can be calculated (Table 5). This allowed us to quantify the improvement of the allosteric properties of the NAMs throughout the series. A difference of a thousand-fold was found between the α of the hit compound **5** and compound **45** demonstrating the strong participation of the additional substituents to the allosteric binding cooperativity. Additionally, the binding constant *K*_B_ was gradually lowered throughout the optimization process, supporting the idea that the various substituents directly improved the NAMs’ affinity for A_2A_R.
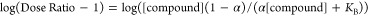
1

**Table 5:** Determined binding cooperativity factor α and dissociation constant K_B_ for chosen NAM compounds

Compound	α	K_B_
**5**	0.04	48.6 nM
**28**	0.001	2.39 nM
**45**	0.00004	0.58 nM

### NAMs Retain Their Potency in High Adenosine Environments

**Figure 7:**
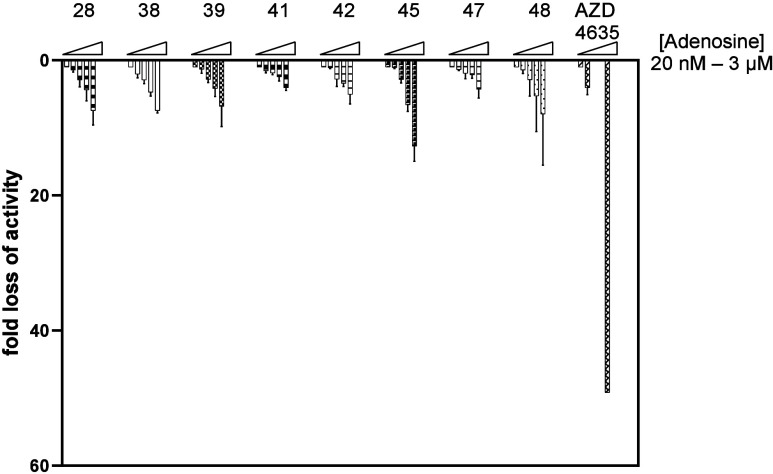
Fold loss of A_2A_R activity at increasing adenosine concentrations for selected NAMs and the orthosteric antagonist imaradenant (AZD4635) given relative to their activity in the presence of 20 nM adenosine.

Allosteric modulators being noncompetitive ligands for protein binding, it is expected that their potency is retained even when endogenous ligand concentration is high. In the case of A_2A_R cancer immunotherapies, the drugs must be able to maintain potency in the TME which can accumulate up to μM concentrations of adenosine.^3^ Their fold loss of activity can be measured as a fold change in IC_50_ from low to highly immunosuppressive adenosine concentrations in cellular assays (20 nM to 3 μM, Figure 7). The NAMs exhibited various behaviors across the series with most compounds exhibiting a loss of activity smaller than 10-times (e.g., compounds **28**, **38**, **41**, **42**, and **48**), which is expected to result in a retained potency in high adenosine environments. Other compounds, such as **45** and **48**, exhibited larger fold changes, easily explained by their greater potency, resulting in wider shifts. Comparatively to orthosteric compounds (more examples in Supporting Information Figure S4), all NAMs showed significantly lower fold loss of activity inherent to their allosteric, noncompetitive, binding mode.

### Proof of Target Engagement and Downstream Cascade Activation for Optimized Compounds

**Figure 8:**
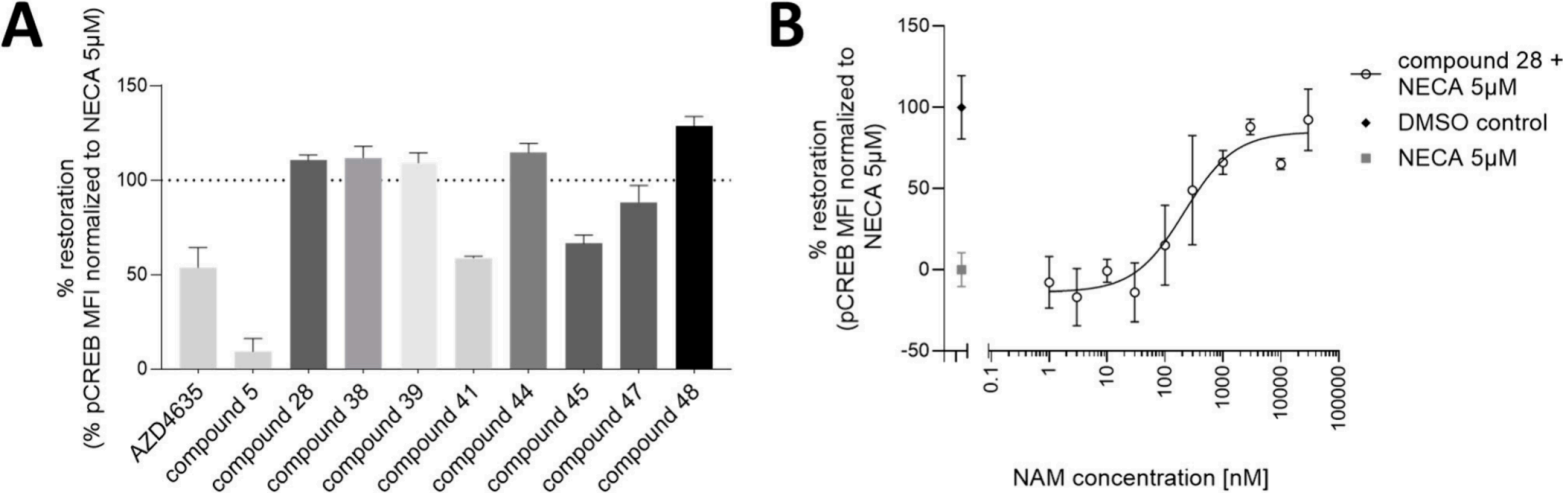
(A) Restoration of a normal immune response by representative compounds measured by the diminution of pCREB in CD4^+^ T cells immunosuppressed by 5 μM NECA, mimicking an adenosine-rich TME. All compounds were tested at 1 μM; repetitions *N* = 2–15. (B) Compound **28** titration in the presence of 5 μM NECA. Normal level of pCREB is given by the DMSO control, whereas maximal immunosuppression is shown in the “NECA 5 μM” control.

The ability of selected NAMs to act on the A_2A_R-immunomodulatory pathway was analyzed by the measurement of downstream signaling effectors. As A_2A_R is known to act through the cAMP–PKA–CREB pathway,^1^ the downstream phosphorylation of CREB could be measured by phospho-flow cytometry in human blood immune cell populations obtained from healthy volunteers. Peripheral blood mononuclear cells (PBMCs) were cocultured in the presence of NAMs then exposed to the highly immunosuppressive condition of 5 μM NECA (a stable adenosine analogue), recapitulating an adenosine-rich TME. The results for pCREB intracellular staining in CD4^+^ T lymphocytes (known to naturally express high levels of A_2A_R) are presented as % restoration of a normal immune response (Figure 8). Early development compound **5** demonstrated no immunomodulatory activity on CD4^+^ T cells. Interestingly, the orthosteric antagonist imaradenant (AZD4635), which was dropped from clinical trials, demonstrated only partial restoration (54% ± 10%). Meanwhile, optimized compounds such as **28**, **38**, and others showed the ability to fully restore the baseline levels of pCREB, indicating complete immune restoration at 1 μM compound (Figure 8A). Titration of compound **28** revealed that immunological responsiveness of CD4^+^ T cells could be partially restored in translational settings using other concentrations of NAMs, including sub-micromolar concentrations (Figure 8B).

In a retrospective analysis for the NAM compounds, it is possible to correlate these encouraging translational results with these compounds’ full % inhibition of A_2A_R activity rather than based on their primary screen IC_50_ alone.

### *In Vitro* ADME Profiling of Compound **45** Reveals a Starting Point for Future *In Vivo* Proof-of-Concept Experiments

Based on its single-digit nanomolar potency and its adequate selectivity profile vis-à-vis the other adenosine receptors, compound **45** was selected to undergo further *in vitro* tests for gut permeability and metabolic stability (both humans and mice). The gut permeability was tested in a Caco-2 monolayer bidirectional assay (Supporting Information Table S3). The apparent permeability coefficient from the apical to the basolateral chambers (*P*_app_(A → B)) was found to be 3.18 × 10^–6^ cm/s with an efflux ratio of 1.25. This indicates that the compound is highly permeable in the assay and expected to be a poor efflux transporter substrate; thus it is likely to be absorbed from the gastrointestinal tract. However, a low recovery percentage was observed in the assay which could arise from cellular retention, rapid metabolism, or adsorption. While all hypotheses remain plausible, rapid metabolism seems more likely as demonstrated by liver microsome stability experiments where the assay half-life values were around 1.5 min and the intrinsic clearance values were >900 μL/min/mg for both human and mouse (Supporting Information Table S4).

Compound **45** was predicted to be moderately to poorly soluble, which is in accordance with laboratory observations. Taken together with the good oral absorption predicted by the Caco-2 cell permeability assay, it would place compound **45** in class II drugs based on the biopharmaceutics classification system (BCS). In the drug development process, the poor solubility of these BCS class II drugs is commonly addressed using formulations such as lipidic carriers or solid dispersions.^33,34^ A first proof-of-concept study in mice could be achieved by dosing compound **45** by intraperitoneal injection (ip), avoiding the hepatic first-pass effect and slowing its rapid metabolism. Other potent compounds of the series, with a good selectivity profile versus the other adenosine receptors, could be nominated for this *in vivo* study, granted that they achieve a better *in vitro* absorption profile and/or metabolic stability than compound **45**. The aim of this proof-of-concept study would be to demonstrate the safety of our A_2A_R NAMs and their efficacy as immunotherapies.

## Conclusion

In conclusion, we identified A_2A_R allosteric hits in a high-throughput screening campaign and confirmed their NAM mode-of-action. The following SAR campaign is the first reported for A_2A_R allosteric modulators and led to the discovery of potent 2-amino-3,5-dicyanopyridine derivatives such as compounds **28** and **45**, with respective IC_50_ values of 90 and 9 nM in the presence of 400 nM adenosine and a full % inhibition of receptor activity at 10 μM. Both compounds’ allosteric mode of action could be demonstrated by progressive fold-shift assay and non-linearity of the Schild plot analysis and retained satisfactory potencies in high-adenosine concentrations. Correlating A_2A_R engagement and downstream signaling was possible thanks to the phosphorylation measurement of the downstream effector CREB for all the optimized NAMs (with IC_50_ < 200 nM and full % inhibition of receptor activity), clearly showcasing the potential of A_2A_R allosteric modulation as a novel approach for efficient and safer cancer immunotherapies.

## Material and Methods

Unless otherwise noted, all reagents were obtained from commercial sources and used without further purification. All compounds tested in translational assays and intended for further *in vivo* experiments are >95% pure by HPLC analysis. Anhydrous solvents such as methanol, DMF, or toluene were purchased over molecular sieves, enclosed by AcroSeal bottles. All preparative columns were performed by flash chromatography on a Büchi Pure C-815 Flash system with a UV detector. The corresponding PureFlash ID cartridges (4, 12, or 24 g, amorphous silica, 35–45 μm mesh) were purchased from Büchi, and the flow rates were set according to the preset parameters (15, 30, and 32 mL/min, respectively). Samples were loaded as a solid deposit prepared with amorphous silica 40–60 μm mesh. All described yields are isolated unless stated otherwise. The ^1^H NMR spectra were recorded on a Bruker 600 MHz spectrometer equipped with a cryoprobe and calibrated on the residual protonated solvent. The ^13^C NMR spectra were recorded at 125 MHz, and the solvent resonance was used as an internal standard. Both ^1^H NMR and ^13^C NMR chemical shifts are reported in parts per million downfield from tetramethylsilane. Low resolution mass spectra were recorded on an Advion Pression^L^, coupled to an ESI source, operating in positive and negative ion mode simultaneously. HRMS were recorded on a Xevo G2 TOF, coupled to an ESI source, operating either in positive or in negative acquisition mode.

### General Procedures

#### General Procedure A

Malononitrile (2 equiv), methanol (0.5 M), aldehyde (1 equiv), 4-DMAP (0.2 equiv), and piperidine (1.2 equiv) were sequentially added to a dry 10 mL round-bottom flask equipped with a magnetic stirrer. The flask was closed by a guard funnel filled with CaCl_2_ beads (to allow air exchange) and stirred at rt (rt range = 19–22 °C) for 16 h. Completion was assessed by TLC (cyclo/EtOAc 8:2). The medium was diluted with DCM (5 × *V*_MeOH_), silica was directly added on top of the mixture, and the solvent was removed under reduced pressure. The crude product was purified by flash chromatography on a prepacked silica cartridge (12 g cartridge for 0.5 to 1 mmol of starting aldehyde) with a cyclo/EtOAc gradient (9:1 → 8:2, 6 CV; 8:2 → 1:1, 3 CV; 1:1, 3 CV; methanol wash).

#### General Procedure B

Malononitrile (1 equiv), methanol (0.5 M), aldehyde (1 equiv), and 4-DMAP (0.2 equiv) were sequentially added to a dry 10 mL round-bottom flask equipped with a magnetic stirrer. The flask was closed by a guard funnel filled with CaCl_2_ beads (to allow air exchange) and stirred at rt for 15 min. The solution was cooled at 0 °C before the addition of more malononitrile (1 equiv) and the dropwise addition of piperidine. The solution was slowly brought back to rt and stirred for 16 h overall. Completion was assessed by TLC (cyclo/EtOAc 8:2). The medium was diluted with DCM (5 × *V*_MeOH_), silica was directly added on top of the mixture, and the solvent was removed under reduced pressure. The crude product was purified by flash chromatography (12 g cartridge for 0.5 to 1 mmol of starting aldehyde) with a cyclo/EtOAc gradient (9:1 → 8:2, 6 CV; 8:2, 3 CV; 8:2 → 1:1, 3 CV; methanol wash).

#### General Procedure C

In a small round-bottom flask equipped with a stir bar, the aldehyde (1 equiv) and the malononitrile (1 equiv) were solubilized in methanol (0.2 M) (solution A). Solution A was vigorously stirred right away. In the case that the aldehyde contained a basic site (e.g., pyridine), no catalyst was needed; else, in a small vial, a solution of cyclic amine (1.2 equiv) in MeOH (0.2 M) was prepared (solution B). A drop or two of solution B was added to solution A. After stirring for 30 min at rt, more malononitrile (1 equiv) was added followed by dropwise addition of the remaining solution B. The reaction was stirred at rt open to air until completion, which was assessed by TLC (cyclo/EtOAc 8:2). The medium was diluted with DCM (5 × *V*_MeOH_), silica was directly added on top of the mixture, and the solvent was removed under reduced pressure. The crude product was purified by flash chromatography (12 g cartridge for 0.5 to 1 mmol of starting aldehyde) with a cyclo/EtOAc gradient (95:5, 3 CV; 95:5 → 8:2, 12 CV; 8:2, 6 CV; methanol wash).

#### General Procedure D

In a 10 mL round-bottom flask equipped with a magnetic stirrer, 2-amino-4-(3-bromophenyl)-6-(piperidin-1-yl)pyridine-3,5-dicarbonitrile (1 equiv), boronic acid (3 equiv), potassium carbonate (3 equiv), and palladium chloride (0.05 equiv) were consecutively added to toluene (0.15 M). The mixture was stirred at 110 °C for 3 h open to air. Completion was assessed by TLC (cyclo/DCM/EtOAc 6:3:1). Palladium and salts were removed by filtration on a Celite pad, and the residue was washed with DCM (2 × 5 mL). Finally, the solvents were removed under reduced pressure, and the crude product was purified by flash chromatography on a silica cartridge (4 g cartridge for 0.15 mmol of starting aldehyde), solid deposit, with a cyclo/EtOAc gradient (10:0, 3 CV; 10:0 → 8:2, 9 CV; 8:2, 3 CV; methanol wash).

#### General Procedure E

In a 2–5 mL microwave tube equipped with a magnetic stirrer, 2-amino-4-(3-bromophenyl)-6-(piperidin-1-yl)pyridine-3,5-dicarbonitrile (1 equiv), boronic acid (1.2 or 3 equiv), and potassium carbonate (3 equiv) were solubilized in a THF/H_2_O 2:1 mixture (0.05 M). The solution was bubbled with argon for 10 min before addition of tetrakis(triphenylphosphine)palladium(0) (0.05 equiv). The tube was sealed and heated at 80 or 100 °C for 15 to 30 min under microwave irradiation. Completion could not be assessed by TLC. The medium was diluted with water and the crude product was extracted by EtOAc × 3. The organic phases were combined, washed with brine, dried over MgSO_4_, and filtered, and the solvent was removed under reduced pressure. The crude product was purified by flash chromatography on a silica cartridge (4 g cartridge for less than 0.5 mmol of starting bromoaryl), solid deposit, with a cyclo/EtOAc gradient (9:1, 6 CV; 9:1 → 6:4, 20 CV; 6:4, 6 CV; methanol wash).

#### General Procedure F

In a 2–5 mL microwave tube equipped with a magnetic stirrer, (3-(2-amino-3,5-dicyano-6-(piperidin-1-yl)pyridin-4-yl)phenyl)boronic acid pinacol ester (1 equiv), bromoaryl (1.2 equiv), and potassium carbonate (3 equiv) were solubilized in a THF/H_2_O 2:1 mixture (0.05 M). The solution was bubbled with argon for 10 min before addition of tetrakis(triphenylphosphine)palladium(0) (0.05 equiv). The tube was sealed and heated at 80 °C for 15 min under microwave irradiation. The medium was diluted with water, and the crude product was extracted by EtOAc × 3. The organic phases were combined, washed with brine, dried over MgSO_4_, and filtered and the solvent was removed under reduced pressure. The crude product was purified by flash chromatography on a silica cartridge (4 g cartridge for less than 0.5 mmol of starting bromoaryl), solid deposit, with a cyclo/(toluene/acetone 4:1) gradient (5:5, 6 CV; 5:5 → 0:10, 6 CV; 0:10, 6 CV; methanol wash).

#### General Procedure G

In a microwave compatible tube equipped with a magnetic stirrer, 2-amino-6-chloro-4-phenylpyridine-3,5-dicarbonitrile (1 equiv) was solubilized in an anhydrous THF/EtOH 4:1 mixture (0.15 M). The desired amine (6 equiv) was added dropwise, and the tube was sealed and heated 30 min to 1 h at 120 °C under microwave irradiation. The reaction progress was assessed by TLC (cyclo/EtOAc 6:4). Brine and a few drops of 1 M HCl (final pH ∼ 1) were added, and the crude product was extracted with EtOAc × 3. The organic phases were combined, dried over MgSO_4_, and filtered, and the solvent was removed under reduced pressure. The crude product was purified by flash chromatography on a silica cartridge (4 g cartridge for 50 mg of starting halogenoaryl), solid deposit, with either an isocratic cyclo/EtOAc gradient (8:2, 6 CV; methanol wash) or a DCM/MeOH gradient (10:0, 3 CV; 10:0 → 0:10, 9 CV; methanol wash).

### Compound Descriptions

#### 2-Amino-4-phenyl-6-(piperidin-1-yl)pyridine-3,5-dicarbonitrile (**5**)

The product was prepared following general procedure C starting from benzaldehyde (0.05 mL, 0.5 mmol, 1 equiv) and was isolated as a white powder (38 mg, 0.13 mmol, 25% isolated yield). **^1^H NMR (600 MHz, CDCl_3_) δ**: 7.56–7.42 (m, 4H), 5.36 (s, 2H), 3.84–3.78 (m, 4H), 1.74–1.67 (m, 6H). **^13^C NMR (151 MHz, CDCl_3_) δ** 162.5, 161.3, 159.5, 134.9, 130.6, 128.9, 128.8, 117.8, 116.7, 83.8, 81.8, 49.3, 26.1, 24.6. **MS (ESI^+^) calculated for C_18_H_17_N_5_:** [M + H]^+^*m*/*z* = 304.2, found *m*/*z* = 304.0. **HRMS (ESI^+^) calculated for C_18_H_17_N_5_:** [M + H]^+^*m*/*z* = 304.1557, found *m*/*z* = 304.1559.

#### 2-Amino-6-(azocan-1-yl)-4-phenylpyridine-3,5-dicarbonitrile (**6**)

The product was prepared following general procedure G starting from 2-amino-6-chloro-4-phenylpyridine-3,5-dicarbonitrile (40 mg, 0.16 mmol, 1 equiv) and azocane (0.04 mL, 2 equiv) and was isolated as a white amorphous powder (7 mg, 0.02 mmol, 13% isolated yield). **^1^H NMR (600 MHz, CDCl_3_) δ** 7.53–7.49 (m, 3H), 7.49–7.44 (m, 2H), 5.64 (s, 2H), 3.92 (t, *J* = 5.8 Hz, 4H), 1.92–1.86 (m, 4H), 1.64–1.61 (m, 4H), 1.58–1.54 (m, 2H). **^13^C NMR (151 MHz, CDCl_3_) δ** 163.4, 158.7, 157.6, 135.1, 130.6, 128.9, 128.7, 117.9, 116.4, 81.9, 81.8, 52.5, 27.2, 27.1, 25.1. **MS (ESI^+^) calculated for C_20_H_21_N_5_:** [M + H]^+^*m*/*z* = 332.2, found *m*/*z* = 332.1. **HRMS (ESI^+^) calculated for C_20_H_21_N_5_:** [M + H]^+^*m*/*z* = 332.1870, found *m*/*z* = 332.1863.

#### 2-Amino-6-(diethylamino)-4-phenylpyridine-3,5-dicarbonitrile (**7**)

The product was prepared following general procedure G starting from 2-amino-6-chloro-4-phenylpyridine-3,5-dicarbonitrile (43 mg, 0.17 mmol, 1 equiv) and diethylamine (0.10 mL, 6 equiv) and was isolated as a white amorphous powder (6 mg, 0.02 mmol, 13% isolated yield). **^1^H NMR (600 MHz, CDCl_3_) δ** 7.50 (q, *J* = 3.7 Hz, 3H), 7.45 (dd, *J* = 6.7, 2.9 Hz, 2H), 5.37 (s, 2H), 3.74 (q, *J* = 7.0 Hz, 4H), 1.30 (t, *J* = 7.0 Hz, 6H). **^13^C NMR (151 MHz, CDCl_3_) δ** 163.0, 159.1, 158.6, 135.3, 130.4, 128.9, 128.7, 118.1, 116.7, 81.8, 81.5, 44.8, 13.7. **MS (ESI^+^) calculated for C_17_H_17_N_5_:** [M + H]^+^*m*/*z* = 292.2, found *m*/*z* = 292.1. **HRMS (ESI^+^) calculated for C_17_H_17_N_5_:** [M + H]^+^*m*/*z* = 292.1557, found *m*/*z* = 292.1555.

#### 2-Amino-6-(bis(2-hydroxyethyl)amino)-4-phenylpyridine-3,5-dicarbonitrile (**8**)

The product was prepared following general procedure G starting from 2-amino-6-chloro-4-phenylpyridine-3,5-dicarbonitrile (40 mg, 0.16 mmol, 1 equiv) and diethanolamine (0.03 mL, 2 equiv) and was isolated as a white amorphous powder (10 mg, 0.06 mmol, 19% isolated yield). **^1^H NMR (600 MHz, MeOD) δ** 7.54–7.48 (m, 3H), 7.48–7.41 (m, 2H), 3.97 (t, *J* = 5.7 Hz, 4H), 3.86 (t, *J* = 5.7 Hz, 4H). **^13^C NMR (151 MHz, MeOD) δ** 164.6, 161.1, 160.6, 137.3, 131.0, 129.7, 119.5, 117.1, 82.7, 81.7, 61.6, 54.9. **MS (ESI^+^) calculated for C_17_H_17_N_5_O_2_:** [M + Na]^+^*m*/*z* = 346.1, found *m*/*z* = 346.0. **HRMS (ESI^+^) calculated for C_17_H_17_N_5_O_2_:** [M + H]^+^*m*/*z* = 324.1456, found *m*/*z* = 324.1433.

#### 2-Amino-4-methyl-6-(piperidin-1-yl)pyridine-3,5-dicarbonitrile (**11**)

The product was prepared following general procedure C starting from excess acetaldehyde (1 mL), solventless, and a drop of piperidine. The intermediate was dried under a N_2_ flux and isolated as a translucent liquid. The second step was performed on 2-ethylidenemalononitrile and piperidine (0.05 mL, 1.2 equiv) and gave a white powder (9 mg, 0.04 mmol, 4% overall yield). **^1^H NMR (600 MHz, CDCl_3_) δ** 5.49 (s, 2H), 3.82–3.76 (m, 4H), 2.53 (s, 3H), and 1.77–1.59 (m, 6H). **^13^C NMR (151 MHz, CDCl_3_) δ** 158.9, 117.2, 116.0, 49.4, 26.1, 24.5, 20.4. **MS (ESI^+^) calculated for C_13_H_15_N_5_:** [M + H]^+^*m*/*z* = 242.1, found *m*/*z* = 242.2. **HRMS (ESI^+^) calculated for C_13_H_15_N_5_:** [M + H]^+^*m*/*z* = 242.1401, found *m*/*z* = 242.1405.

#### 2-Amino-4-(naphthalen-2-yl)-6-(piperidin-1-yl)pyridine-3,5-dicarbonitrile (**12**)

The product was prepared following general procedure A starting from 2-naphthaldehyde (78 mg, 0.5 mmol, 1 equiv) and was isolated as a light orange paste (36 mg, 0.10 mmol, 20% isolated yield). **^1^H NMR (600 MHz, CDCl_3_) δ** 8.00 (d, *J* = 1.8 Hz, 1H), 7.98 (d, *J* = 8.5 Hz, 1H), 7.93 (dd, *J* = 7.7, 1.8 Hz, 1H), 7.56 (td, *J* = 7.6, 1.6 Hz, 3H), 5.38 (s, 2H), 3.85–3.80 (m, 4H), 1.73–1.69 (m, 6H). **^13^C NMR (151 MHz, CDCl_3_) δ** 162.4, 161.3, 159.6, 134.2, 133.0, 132.3, 129.1, 128.85, 128.83, 128.0, 127.6, 126.9, 125.7, 117.9, 116.8, 84.0, 82.0, 49.4, 26.2, 24.6. **MS (ESI^+^) calculated for C_22_H_19_N_5_:** [M + H]^+^*m*/*z* = 354.2, found *m*/*z* = 354.4. **HRMS (ESI^+^) calculated for C_22_H_19_N_5_:** [M + Na]^+^*m*/*z* = 376.1533, found *m*/*z* = 376.1524.

#### 2-Amino-6-(piperidin-1-yl)-4-(*o*-tolyl)pyridine-3,5-dicarbonitrile (**13**)

The product was prepared following general procedure A starting from 2-methylbenzaldehyde (0.06 mL, 0.5 mmol, 1 equiv) and was isolated as a white powder (41 mg, 0.13 mmol, 26% isolated yield). **^1^H NMR (600 MHz, CDCl_3_) δ** 7.38 (td, *J* = 7.5, 1.4 Hz, 1H), 7.34–7.27 (m, 2H), 7.17 (dd, *J* = 7.6, 1.4 Hz, 1H), 5.53 (s, 2H), 3.85–3.80 (m, 4H), 2.27 (s, 3H), 1.74–1.69 (m, 6H). **^13^C NMR (151 MHz, CDCl_3_) δ** 163.4, 159.7, 158.9, 135.1, 134.8, 130.9, 130.2, 128.1, 126.4, 116.8, 115.8, 84.6, 82.8, 49.5, 26.1, 24.4, 19.5. **MS (ESI^+^) calculated for C_19_H_19_N_5_:** [M + H]^+^*m*/*z* = 318.2, found *m*/*z* = 318.2. **HRMS (ESI^+^) calculated for C_19_H_19_N_5_:** [M + H]^+^*m*/*z* = 318.1714, found *m*/*z* = 318.1730.

#### 2-Amino-6-(piperidin-1-yl)-4-(*m*-tolyl)pyridine-3,5-dicarbonitrile (**14**)

The product was prepared following general procedure A starting from 3-methylbenzaldehyde (0.06 mL, 0.5 mmol, 1 equiv) and was isolated as a white powder (45 mg, 0.14 mmol, 28% isolated yield). **^1^H NMR (600 MHz, CDCl_3_) δ** 7.39 (t, *J* = 8.0 Hz, 1H), 7.31 (d, *J* = 7.2 Hz, 1H), 7.30–7.25 (m, 2H), 5.52 (s, 2H), 3.85–3.78 (m, 4H), 2.43 (s, 3H), 1.74–1.69 (m, 6H). **^13^C NMR (151 MHz, CDCl_3_) δ** 162.9, 160.7, 159.3, 138.7, 134.7, 131.5, 129.3, 128.9, 125.9, 117.6, 116.4, 83.9, 82.0, 49.7, 26.1, 24.5, 21.6. **MS (ESI^+^) calculated for C_19_H_19_N_5_:** [M + H]^+^*m*/*z* = 318.2, found *m*/*z* = 318.2. **HRMS (ESI^+^) calculated for C_19_H_19_N_5_:** [M + H]^+^*m*/*z* = 318.1714, found *m*/*z* = 318.1730.

#### 2-Amino-6-(piperidin-1-yl)-4-(*p*-tolyl)pyridine-3,5-dicarbonitrile (**15**)

The product was prepared following general procedure A starting from 4-methylbenzaldehyde (0.06 mL, 0.5 mmol, 1 equiv) and was isolated as a white powder (35 mg, 0.11 mmol, 22% isolated yield). **^1^H NMR (600 MHz, CDCl_3_) δ** 7.39 (d, *J* = 7.9 Hz, 2H), 7.31 (d, *J* = 7.8 Hz, 2H), 5.40 (s, 2H), 3.82–3.77 (m, 4H), 2.42 (s, 3H), 1.73–1.68 (m, 6H). **^13^C NMR (151 MHz, CDCl_3_) δ** 162.7, 161.1, 159.5, 140.9, 131.9, 129.7, 128.8, 117.9, 116.7, 83.8, 81.8, 49.5, 26.1, 24.5, 21.6. **MS (ESI^+^) calculated for C_19_H_19_N_5_:** [M + H]^+^*m*/*z* = 318.2, found *m*/*z* = 318.2. **HRMS (ESI^+^) calculated for C_19_H_19_N_5_:** [M + H]^+^*m*/*z* = 318.1714, found *m*/*z* = 318.1702.

#### 2-Amino-4-(2-chlorophenyl)-6-(piperidin-1-yl)pyridine-3,5-dicarbonitrile (**16**)

The product was prepared following general procedure A starting from 2-chlorobenzaldehyde (0.06 mL, 0.5 mmol, 1 equiv) and was isolated as a white powder (48 mg, 0.14 mmol, 28% isolated yield). **^1^H NMR (600 MHz, CDCl_3_) δ** 7.54 (dd, *J* = 7.9, 1.4 Hz, 1H), 7.44 (td, *J* = 7.7, 1.9 Hz, 1H), 7.40 (td, *J* = 7.5, 1.4 Hz, 1H), 7.31 (dd, *J* = 7.5, 1.9 Hz, 1H), 5.36 (s, 2H), 3.84–3.79 (m, 4H), 1.75–1.66 (m, 7H). **^13^C NMR (151 MHz, CDCl_3_) δ** 160.3, 159.6, 158.9, 134.1, 132.3, 131.6, 130.5, 129.9, 127.5, 116.6, 115.5, 84.7, 82.7, 49.5, 26.1, 24.4. **MS (ESI^+^) calculated for C_18_H_16_N_5_Cl:** [M + H]^+^*m*/*z* = 338.1, found *m*/*z* = 338.0. **HRMS (ESI^+^) calculated for C_18_H_16_N_5_Cl:** [M + H]^+^*m*/*z* = 338.1167, found *m*/*z* = 338.1168.

#### 2-Amino-4-(3-chlorophenyl)-6-(piperidin-1-yl)pyridine-3,5-dicarbonitrile (**17**)

The product was prepared following general procedure A starting from 3-chlorobenzaldehyde (0.06 mL, 0.5 mmol, 1 equiv) and was isolated as a white powder (18 mg, 0.05 mmol, 11% isolated yield). **^1^H NMR (600 MHz, CDCl_3_) δ** 7.49 (ddd, *J* = 8.1, 2.3, 1.3 Hz, 1H), 7.47–7.42 (m, 2H), 7.36 (dt, *J* = 7.6, 1.5 Hz, 1H), 5.43 (s, 2H), 3.81 (dd, *J* = 6.4, 3.8 Hz, 4H), 1.73–1.67 (m, 6H). **^13^C NMR (151 MHz, CDCl_3_) δ** 161.0, 160.5, 159.2, 136.5, 134.9, 130.8, 130.4, 128.8, 127.0, 117.3, 116.1, 83.6, 81.8, 49.5, 26.1, 24.5. **MS (ESI^+^) calculated for C_18_H_16_N_5_Cl:** [M + H]^+^*m*/*z* = 338.1, found *m*/*z* = 338.1. **HRMS (ESI^+^) calculated for C_18_H_16_N_5_Cl:** [M + H]^+^*m*/*z* = 338.1167, found *m*/*z* = 338.1168.

#### 2-Amino-4-(4-chlorophenyl)-6-(piperidin-1-yl)pyridine-3,5-dicarbonitrile (**18**)

The product was prepared following general procedure A starting from 4-chlorobenzaldehyde (70 mg, 0.5 mmol, 1 equiv) and was isolated as a white powder (42 mg, 0.12 mmol, 25% isolated yield). **^1^H NMR (600 MHz, CDCl_3_) δ** 7.48 (d, *J* = 8.5 Hz, 1H), 7.43 (d, *J* = 8.5 Hz, 1H), 5.40 (s, 1H), 3.82–3.77 (m, 2H), 1.72–1.66 (m, 3H). **^13^C NMR (151 MHz, CDCl_3_) δ** 161.3, 160.7, 159.3, 137.0, 133.2, 130.2, 129.4, 117.5, 116.3, 83.5, 81.7, 49.5, 26.1, 24.5. **MS (ESI^+^) calculated for C_18_H_16_N_5_Cl:** [M + H]^+^*m*/*z* = 338.1, found *m*/*z* = 338.0. **HRMS (ESI^+^) calculated for C_18_H_16_N_5_Cl:** [M + H]^+^*m*/*z* = 338.1167, found *m*/*z* = 338.1168.

#### 2-Amino-4-(2-methoxyphenyl)-6-(piperidin-1-yl)pyridine-3,5-dicarbonitrile (**19**)

The product was prepared following general procedure B starting from 2-methoxybenzaldehyde (0.06 mL, 0.5 mmol, 1 equiv) and was isolated as a white powder (16 mg, 0.05 mmol, 10% isolated yield). **^1^H NMR (600 MHz, CDCl_3_) δ** 7.46 (ddd, *J* = 8.4, 7.5, 1.7 Hz, 1H), 7.25 (dd, *J* = 7.5, 1.8 Hz, 1H), 7.07 (td, *J* = 7.5, 1.0 Hz, 1H), 7.04 (dd, *J* = 8.4, 1.0 Hz, 1H), 5.55 (s, 2H), 3.87 (s, 3H), 3.86–3.78 (m, 4H), 1.73–1.70 (m, 6H). **^13^C NMR (151 MHz, CDCl_3_) δ** 160.3, 159.0, 156.3, 132.1, 130.1, 123.8, 121.0, 117.3, 116.3, 111.8, 85.3, 83.2, 55.9, 49.5, 26.1, 24.5. **MS (ESI^+^) calculated for C_19_H_19_N_5_O:** [M + H]^+^*m*/*z* = 334.2, found *m*/*z* = 334.5. **HRMS (ESI^+^) calculated for C_19_H_19_N_5_O:** [M + H]^+^*m*/*z* = 334.1663, found *m*/*z* = 334.1641.

#### 2-Amino-4-(3-methoxyphenyl)-6-(piperidin-1-yl)pyridine-3,5-dicarbonitrile (**20**)

The product was prepared following general procedure B starting from 3-methoxybenzaldehyde (0.06 mL, 0.5 mmol, 1 equiv) and was isolated as a white powder (18 mg, 0.05 mmol, 11% isolated yield). **^1^H NMR (600 MHz, CDCl_3_) δ** 7.42 (t, *J* = 8.0 Hz, 1H), 7.05 (tdd, *J* = 7.6, 2.1, 0.8 Hz, 2H), 6.99 (dd, *J* = 2.5, 1.6 Hz, 1H), 5.60 (s, 2H), 3.85 (s, 3H), 3.84–3.79 (m, 4H), 1.74–1.69 (m, 6H). **^13^C NMR (151 MHz, CDCl_3_) δ** 162.5, 160.6, 159.7, 159.2, 135.9, 130.1, 121.0, 117.4, 116.5, 116.3, 114.2, 83.9, 82.0, 55.6, 49.6, 26.1, 24.4. **MS (ESI^+^) calculated for C_19_H_19_N_5_O:** [M + H]^+^*m*/*z* = 334.2, found *m*/*z* = 334.5. **HRMS (ESI^+^) calculated for C_19_H_19_N_5_O:** [M + H]^+^*m*/*z* = 334.1663, found *m*/*z* = 334.1641.

#### 2-Amino-4-(4-methoxyphenyl)-6-(piperidin-1-yl)pyridine-3,5-dicarbonitrile (**21**)

The product was prepared following general procedure B starting from 4-methoxybenzaldehyde (0.06 mL, 0.5 mmol, 1 equiv) and was isolated as a white powder (24 mg, 0.07 mmol, 14% isolated yield). **^1^H NMR (600 MHz, CDCl_3_) δ** 7.47 (d, *J* = 9.4 Hz, 2H), 7.01 (d, *J* = 9.4 Hz, 2H), 5.46 (s, 2H), 3.86 (s, 3H), 3.85–3.77 (m, 4H), 1.75–1.70 (m, 6H). **^13^C NMR (151 MHz, CDCl_3_) δ** 162.2, 161.6, 161.3, 159.6, 130.6, 126.9, 118.1, 116.9, 114.4, 83.7, 81.7, 55.5, 49.5, 27.1, 26.1, 24.5. **MS (ESI^+^) calculated for C_19_H_19_N_5_O:** [M + H]^+^*m*/*z* = 334.2, found *m*/*z* = 334.5. **HRMS (ESI^+^) calculated for C_19_H_19_N_5_O:** [M + H]^+^*m*/*z* = 334.1663, found *m*/*z* = 334.1641.

#### 2-Amino-4-(3-hydroxyphenyl)-6-(piperidin-1-yl)pyridine-3,5-dicarbonitrile (**22**)

The product was prepared following general procedure A starting from 3-hydroxybenzaldehyde (61 mg, 0.5 mmol, 1 equiv) and was isolated as a white powder (38 mg, 0.12 mmol, 24% isolated yield). **^1^H NMR (600 MHz, (CD_3_)_2_SO) δ** 9.75 (s, 1H), 7.39 (s, 2H), 7.31 (t, *J* = 7.9 Hz, 1H), 6.91 (ddd, *J* = 8.2, 2.5, 1.0 Hz, 1H), 6.86 (dt, *J* = 7.5, 1.3 Hz, 1H), 6.82 (t, *J* = 2.0 Hz, 1H), 3.73–3.68 (m, 4H), 1.69–1.54 (m, 6H). **^13^C NMR (151 MHz, (CD_3_)_2_SO) δ** 161.8, 160.7, 159.7, 157.2, 136.5, 129.7, 119.2, 117.7, 116.8, 116.2, 115.3, 81.4, 80.7, 48.5, 25.6, 23.9. **MS (ESI^+^) calculated for C_18_H_17_N_5_O:** [M + H]^+^*m*/*z* = 320.2, found *m*/*z* = 320.4. **HRMS (ESI^+^) calculated for C_18_H_17_N_5_O:** [M + H]^+^*m*/*z* = 320.1506, found *m*/*z* = 320.1500.

#### 2-Amino-4-(3-phenoxyphenyl)-6-(piperidin-1-yl)pyridine-3,5-dicarbonitrile (**23**)

The product was prepared following general procedure A starting from 3-phenoxybenzaldehyde (0.09 mL, 0.5 mmol, 1 equiv) and was isolated as a white powder (36 mg, 0.09 mmol, 18% isolated yield). **^1^H NMR (600 MHz, CDCl_3_) δ** 7.47 (t, *J* = 8.0 Hz, 1H), 7.36 (tt, *J* = 7.4, 2.1 Hz, 2H), 7.19 (dt, *J* = 7.7, 1.4 Hz, 1H), 7.17–7.15 (m, 1H), 7.15–7.08 (m, 4H), 5.44 (s, 2H), 3.82–3.76 (m, 4H), 1.72–1.67 (m, 6H). **^13^C NMR (151 MHz, CDCl_3_) δ** 161.8, 160.8, 159.3, 157.6, 156.7, 136.4, 130.5, 130.1, 123.8, 123.4, 120.9, 119.4, 118.9, 117.5, 116.3, 83.7, 81.8, 49.4, 26.1, 24.5. **MS (ESI^+^) calculated for C_24_H_21_N_5_O:** [M + H]^+^*m*/*z* = 396.2, found *m*/*z* = 396.1. **HRMS (ESI^+^) calculated for C_24_H_21_N_5_O:** [M + H]^+^*m*/*z* = 396.1819, found *m*/*z* = 396.1806.

#### 2-Amino-4-(3-(benzyloxy)phenyl)-6-(piperidin-1-yl)pyridine-3,5-dicarbonitrile (**24**)

The product was prepared following general procedure A starting from 3-benzyloxybenzaldehyde (106 mg, 0.5 mmol, 1 equiv) and was isolated as a white powder (61 mg, 0.15 mmol, 30% isolated yield). **^1^H NMR (600 MHz, CDCl_3_) δ** 7.47–7.38 (m, 5H), 7.34 (t, *J* = 7.4 Hz, 1H), 7.13–7.07 (m, 3H), 5.47 (s, 2H), 5.10 (s, 2H), 3.83–3.78 (m, 4H), 1.73–1.68 (m, 6H). **^13^C NMR (151 MHz, CDCl_3_) δ** 162.3, 160.9, 159.4, 159.0, 136.7, 136.1, 130.1, 128.7, 128.2, 127.8, 121.4, 117.6, 117.3, 116.5, 115.2, 83.7, 81.9, 70.4, 49.4, 26.1, 24.5. **MS (ESI^+^) calculated for C_25_H_23_N_5_O:** [M + H]^+^*m*/*z* = 410.2, found *m*/*z* = 410.1. **HRMS (ESI^+^) calculated for C_25_H_23_N_5_O:** [M + Na]^+^*m*/*z* = 432.1795, found *m*/*z* = 432.1769.

#### Methyl 3-(2-Amino-3,5-dicyano-6-(piperidin-1-yl)pyridin-4-yl)benzoate (**25**)

The product was prepared following general procedure C starting from methyl 3-formylbenzoate (492 mg, 3 mmol, 1 equiv) and piperidine (0.59 mL, 2 equiv) and was isolated as a white powder (179 mg, 0.5 mmol,16% isolated yield). **^1^H NMR (600 MHz, CDCl_3_) δ** 8.19 (dt, *J* = 7.8, 1.5 Hz, 1H), 8.16 (t, *J* = 1.7 Hz, 1H), 7.67 (dt, *J* = 7.7, 1.5 Hz, 1H), 7.61 (t, *J* = 7.7 Hz, 1H), 5.49 (s, 2H), 3.94 (s, 3H), 3.85–3.79 (m, 4H), 1.74–1.69 (m, 6H). **^13^C NMR (151 MHz, CDCl_3_) δ** 166.2, 161.4, 160.5, 159.2, 135.1, 132.9, 131.5, 130.9, 129.9, 129.1, 117.2, 116.1, 83.6, 81.7, 52.4, 49.3, 26.0, 24.4. **MS (ESI^+^) calculated for C_20_H_19_N_5_O_2_:** [M + H]^+^*m*/*z* = 362.2, found *m*/*z* = 362.5. **HRMS (ESI^+^) calculated for C_20_H_19_N_5_O_2_:** [M + H]^+^*m*/*z* = 362.1612, found *m*/*z* = 362.1608.

#### 2-Amino-4-(3-fluorophenyl)-6-(piperidin-1-yl)pyridine-3,5-dicarbonitrile (**26**)

The product was prepared following general procedure A starting from 3-fluorobenzaldehyde (0.05 mL, 0.5 mmol, 1 equiv) and was isolated as a white powder (34 mg, 0.06 mmol, 11% isolated yield). **^1^H NMR (600 MHz, CDCl_3_) δ** 7.55 (t, *J* = 7.9 Hz, 1H), 7.43 (d, *J* = 7.7 Hz, 1H), 7.40–7.34 (m, 2H), 5.38 (s, 2H), 3.83–3.79 (m, 4H), 1.74–1.67 (m, 6H). **^13^C NMR (151 MHz, CDCl_3_) δ** 163.5, 161.0, 160.7 (d, *J* = 353.3 Hz), 159.4, 136.9 (d, *J* = 7.0 Hz), 130.8 (d, *J* = 8.3 Hz), 124.6 (d, *J* = 3.1 Hz), 117.6 (d, *J* = 20.9 Hz), 117.4, 116.3, 116.1 (d, *J* = 23.1 Hz), 83.6, 81.7, 49.3, 26.1, 24.5. **MS (ESI^+^) calculated for C_18_H_16_N_5_F:** [M + H]^+^*m*/*z* = 322.2, found *m*/*z* = 322.7. **HRMS (ESI^+^) calculated for C_18_H_16_N_5_F:** [M + H]^+^*m*/*z* = 322.1463, found *m*/*z* = 322.1478.

#### 2-Amino-4-(3-bromophenyl)-6-(piperidin-1-yl)pyridine-3,5-dicarbonitrile (**27**)

For the small batch, the product was prepared following general procedure B starting from 3-bromobenzaldehyde (0.06 mL, 0.5 mmol, 1 equiv) and was isolated as a white powder (41 mg, 0.11 mmol, 21% isolated yield).

For the large batch, the product was prepared following general procedure C starting from 3-bromobenzaldehyde (5.83 g, 31.5 mmol, 1.05 equiv) at 3 M in MeOH and piperidine (0.15 mL, 0.05 equiv), and the intermediate was isolated as a white powder (6.76 g, 29 mmol, 97% isolated yield). The second step was performed twice on (3-bromobenylidene)malononitrile (3.50 and 3.21 g, 15 and 13.8 mmol, 1 equiv) and piperidine (1.55 and 1.34 mL, 1.05 equiv) and gave a white powder (1.05 and 1.03 g, 2.7 and 2.7 mmol, 18% and 20% isolated yield, 17% and 19% overall yield).

**^1^H NMR (600 MHz, CDCl_3_) δ** 7.65 (dt, *J* = 7.3, 1.9 Hz, 1H), 7.61 (t, *J* = 1.8 Hz, 1H), 7.43–7.36 (m, 2H), 5.50 (s, 2H), 3.84–3.79 (m, 4H), 1.76–1.68 (m, 6H). **^13^C NMR (151 MHz, CDCl_3_) δ** 160.9, 160.5, 159.3, 136.8, 133.7, 131.6, 130.6, 127.4, 122.9, 117.3, 116.1, 83.6, 81.8, 49.5, 26.1, 24.5. **MS (ESI^+^) calculated for C_18_H_16_N_5_Br:** [M + H]^+^*m*/*z* = 382.1, 384.1, found *m*/*z* = 382.1, 384.1. **HRMS (ESI^+^) calculated for C_18_H_16_N_5_Br:** [M + H]^+^*m*/*z* = 382.0662, found *m*/*z* = 382.0667.

#### 4-([1,1′-Biphenyl]-3-yl)-2-amino-6-(piperidin-1-yl)pyridine-3,5-dicarbonitrile (**28**)

For the small batch, the product was prepared following general procedure A starting from [1,1′-biphenyl]-3-carbaldehyde (0.080 mL, 0.5 mmol, 1 equiv) and was isolated as a white powder (13 mg, 0.03 mmol, 7% isolated yield).

For the large batch, the product was prepared following general procedure E at 100 °C for 30 min to 1 h (depending on the catalyst’s batch), starting from 2-amino-4-(3-bromophenyl)-6-(piperidin-1-yl)pyridine-3,5-dicarbonitrile (400 mg, 1.05 mmol, 1 equiv) and phenylboronic acid monohydrate (438 mg, 3 equiv). The crude product’s treatment was performed as followed: (1) dilution of the medium by addition of H_2_O (20 mL); (2) extraction by EtOAc (3 × 15 mL); (3) organic phases were dried with MgSO_4_, filtered on a Celite pad, then concentrated under reduced pressure; (4) the crude residue was stirred at rt in a minimum of Et_2_O for 10 min then filtered; step (4) was repeated until pure. The product was isolated as a white powder (290 mg, 0.15 mmol, 73% isolated yield).

**^1^H NMR (600 MHz, CDCl_3_) δ** 7.73–7.70 (m, 2H), 7.66–7.62 (m, 2H), 7.58 (t, *J* = 7.7 Hz, 1H), 7.49 (dt, *J* = 7.7, 1.5 Hz, 1H), 7.45 (t, *J* = 7.7 Hz, 2H), 7.37 (tt, *J* = 7.4, 1.3 Hz, 1H), 5.49 (s, 2H), 3.86–3.80 (m, 4H), 1.73–1.70 (m, 6H). **^13^C NMR (151 MHz, CDCl_3_) δ** 162.4, 161.1, 159.5, 142.0, 140.6, 135.2, 129.5, 129.4, 129.0, 127.9, 127.8, 127.7, 127.6, 117.8, 116.6, 83.8, 81.9, 49.5, 26.2, 24.5. **MS (ESI^+^) calculated for C_24_H_21_N_5_:** [M + H]^+^*m*/*z* = 380.2, found *m*/*z* = 380.1. **HRMS (ESI^+^) calculated for C_24_H_21_N_5_:** [M + H]^+^*m*/*z* = 380.1870, found *m*/*z* = 380.1859.

#### 2-Amino-4-(3-nitrophenyl)-6-(piperidin-1-yl)pyridine-3,5-dicarbonitrile (**29**)

The product was prepared following general procedure A starting from 3-nitrobenzaldehyde (75 mg, 0.5 mmol, 1 equiv) and was isolated as a white powder (28 mg, 0.08 mmol, 16% isolated yield). **^1^H NMR (600 MHz, CDCl_3_) δ** 8.39 (ddd, *J* = 8.2, 2.3, 1.1 Hz, 1H), 8.37 (t, *J* = 2.0 Hz, 1H), 7.81 (dt, *J* = 7.7, 1.4 Hz, 1H), 7.73 (t, *J* = 7.9 Hz, 1H), 5.48 (s, 2H), 3.86–3.82 (m, 4H), 1.77–1.68 (m, 6H). **^13^C NMR (151 MHz, CDCl_3_) δ** 160.4, 159.8, 159.3, 148.4, 136.5, 134.8, 130.3, 125.3, 124.2, 117.2, 115.9, 83.3, 81.6, 49.3, 26.2, 24.5. **MS (ESI^+^) calculated for C_18_H_17_N_5_O:** [M + H]^+^*m*/*z* = 349.1, found *m*/*z* = 349.4. **HRMS (ESI^+^) calculated for C_18_H_17_N_5_O:** [M + H]^+^*m*/*z* = 349.1408, found *m*/*z* = 349.1392.

#### 2-Amino-4-(3-cyanophenyl)-6-(piperidin-1-yl)pyridine-3,5-dicarbonitrile (**30**)

The product was prepared following general procedure A starting from 3-cyanobenzaldehyde (66 mg, 0.5 mmol, 1 equiv) and was isolated as a light orange solid (24 mg, 0.04 mmol, 7% isolated yield). **^1^H NMR (600 MHz, CDCl_3_) δ** 7.74 (dt, *J* = 7.7, 1.4 Hz, 1H), 7.69 (t, *J* = 1.7 Hz, 1H), 7.64 (dt, *J* = 7.8, 1.5 Hz, 2H), 7.58 (t, *J* = 7.7 Hz, 1H), 5.34 (s, 2H), 3.78–3.73 (m, 4H), 1.69–1.60 (m, 6H). **^13^C NMR (151 MHz, CDCl_3_) δ** 160.6, 159.9, 159.3, 136.3, 133.9, 133.1, 132.3, 130.0, 118.0, 117.3, 116.0, 113.5, 111.6, 83.2, 81.5, 49.3, 26.1, 24.5. **MS (ESI^+^) calculated for C_19_H_16_N_6_:** [M + H]^+^*m*/*z* = 329.2, found *m*/*z* = 329.4. **HRMS (ESI^+^) calculated for C_19_H_16_N_6_:** [M + Na]^+^*m*/*z* = 351.1329, found *m*/*z* = 351.1334.

#### 2-Amino-4-(3-ethynylphenyl)-6-(piperidin-1-yl)pyridine-3,5-dicarbonitrile (**31**)

This synthesis is a two-step procedure starting with Sonogashira cross-coupling and followed by silyl deprotection. (a) In a 5 mL sealed tube purged with argon and equipped with a magnetic stirrer, anhydrous THF (1.5 mL) was bubbled with argon for 5–10 min. 2-Amino-4-(3-bromophenyl)-6-(piperidin-1-yl)pyridine-3,5-dicarbonitrile (57 mg, 0.15 mmol, 1 equiv), copper iodide (1.4 mg, 0.05 equiv), and Pd(dppf)Cl_2_ (5.5 mg, 0.05 equiv) were loaded, and the vessel was purged with argon. Dipropylamine (0.08 mL, 4 equiv) and triisopropylsilylacetylene (0.04 mL, 1.2 equiv) were added with a syringe. The reaction mixture was sealed and heated to 80 °C for 18 h. Completion was assessed by TLC (*R*_*f*_ = 0.25 in cyclo/EtOAc 8:2). The palladium and salts were removed by filtration on a Celite pad, the residue was washed with EtOAc (3 × 5 mL), and all solvents were removed under reduced pressure. The crude intermediate was purified by flash chromatography on a 12 g silica cartridge, solid deposit, with a cyclo/EtOAc gradient (95:5 → 8:2, 12 CV) and isolated as a white powder (38 mg, 0.08 mmol, 52% isolated yield). (b) In a 5 mL round-bottom flask with a magnetic stirrer, the intermediate (20 mg, 0.04 mmol, 1 equiv) was solubilized in anhydrous THF (1.5 mL). The mixture was cooled to 0 °C then a TBAF solution (1 M in THF, 0.08 mL, 2 equiv) was slowly added. The reaction was left stirring for 15 min at 0 °C. Completion was assessed by TLC (*R*_*f*_ = 0.15 in cyclo/EtOAc 8:2). The reaction was quenched by addition of water (10 mL) and brine (5 mL). The crude product was extracted by EtOAc (3 × 10 mL), dried over MgSO_4_, filtered, isolated under reduced pressure, and used as such (19 mg, 0.04 mmol, quantitative isolated yield). **^1^H NMR (600 MHz, CDCl_3_) δ** 7.62 (dt, *J* = 7.4, 1.7 Hz, 1H), 7.59 (d, *J* = 1.7 Hz, 1H), 7.48 (t, *J* = 7.6 Hz, 1H), 7.45 (dt, *J* = 7.8, 1.8 Hz, 1H), 5.36 (s, 2H), 3.86–3.76 (m, 4H), 3.12 (s, 1H), 1.75–1.68 (m, 6H). **^13^C NMR (151 MHz, CDCl_3_) δ** 161.3, 161.0, 159.4, 135.3, 134.1, 132.3, 129.07, 129.05, 123.2, 117.5, 116.3, 83.6, 81.7, 78.6, 49.3, 26.2, 24.5. **MS (ESI^+^) calculated for C_20_H_17_N_5_:** [M + H]^+^*m*/*z* = 328.2, found *m*/*z* = 328.1. **HRMS (ESI^+^) calculated for C_20_H_17_N_5_:** [M + H]^+^*m*/*z* = 328.1557, found *m*/*z* = 328.1543.

#### 2-Amino-4-(2,6-dimethylphenyl)-6-(piperidin-1-yl)pyridine-3,5-dicarbonitrile (**32**)

The product was prepared following general procedure A starting from 2,6-dimethylbenzaldehyde (67 mg, 0.5 mmol, 1 equiv) and was isolated as a white powder (35 mg, 11 mmol, 21% isolated yield). **^1^H NMR (600 MHz, CDCl_3_) δ** 7.26 (t, *J* = 7.6 Hz, 1H), 7.14 (d, *J* = 7.6 Hz, 2H), 5.69 (s, 2H), 3.87–3.82 (m, 4H), 2.16 (s, 6H), 1.74–1.71 (m, 6H). **^13^C NMR (151 MHz, CDCl_3_) δ** 163.6, 159.1, 134.7, 134.4, 129.7, 128.2, 116.4, 115.3, 84.3, 82.5, 49.5, 26.1, 24.4, 19.8. **MS (ESI^+^) calculated for C_20_H_21_N_5_:** [M + H]^+^*m*/*z* = 332.2, found *m*/*z* = 332.1. **HRMS (ESI^+^) calculated for C_20_H_21_N_5_:** [M + Na]^+^*m*/*z* = 354.1690, found *m*/*z* = 354.1679.

#### 2-Amino-4-(3,5-dimethylphenyl)-6-(piperidin-1-yl)pyridine-3,5-dicarbonitrile (**33**)

The product was prepared following general procedure A starting from 3,5-dimethylbenzaldehyde (0.07 mL, 0.5 mmol, 1 equiv) and was isolated as a white powder (64 mg, 0.19 mmol, 39% isolated yield). **^1^H NMR (600 MHz, CDCl_3_) δ** 7.13 (s, 1H), 7.07 (s, 2H), 5.62 (s, 2H), 3.83–3.79 (m, 4H), 2.38 (s, 6H), 1.76–1.67 (m, 6H). **^13^C NMR (151 MHz, CDCl_3_) δ** 163.2, 159.2, 138.5, 134.7, 132.5, 126.4, 117.6, 116.4, 83.9, 82.0, 49.7, 26.1, 24.4, 21.5. **MS (ESI^+^) calculated for C_20_H_21_N_5_:** [M + H]^+^*m*/*z* = 332.2, found *m*/*z* = 332.1. **HRMS (ESI^+^) calculated for C_20_H_21_N_5_:** [M + Na]^+^*m*/*z* = 354.1690, found *m*/*z* = 354.1679.

#### 2-Amino-4-(3-chloro-2-fluorophenyl)-6-(piperidin-1-yl)pyridine-3,5-dicarbonitrile (**34**)

The product was prepared following general procedure C starting from 3-chloro-2-fluorobenzaldehyde (79 mg, 0.5 mmol, 1 equiv) and piperidine (0.06 mL, 1.2 equiv) and isolated as a white powder (39 mg, 0.11 mmol, 22% isolated yield). **^1^H NMR (600 MHz, CDCl_3_) δ** 7.58–7.52 (m, 1H), 7.30–7.26 (m, 1H), 7.24 (t, *J* = 7.8 Hz, 1H), 5.37 (s, 2H), 3.86–3.77 (m, 4H), 1.76–1.65 (m, 6H). **^13^C NMR (151 MHz, CDCl_3_) δ** 160.3, 159.2, 155.6, 153.9 (d, *J* = 252.2 Hz), 133.0, 128.8 (d, *J* = 1.4 Hz), 125.2 (d, *J* = 4.7 Hz), 124.4 (d, *J* = 15.3 Hz), 122.5 (d, *J* = 17.8 Hz), 116.9, 115.8, 84.4, 82.3, 49.2, 26.1, 24.5. **MS (ESI^+^) calculated for C_18_H_15_N_5_ClF:** [M + H]^+^*m*/*z* = 356.1, found *m*/*z* = 356.1. **HRMS (ESI^+^) calculated for C_18_H_15_N_5_ClF:** [M + H]^+^*m*/*z* = 356.1073, found *m*/*z* = 356.1070.

#### 2-Amino-4-(3-chloro-4-fluorophenyl)-6-(piperidin-1-yl)pyridine-3,5-dicarbonitrile (**35**)

The product was prepared following general procedure C starting from 3-chloro-4-fluorobenzaldehyde (317 mg, 2 mmol, 1 equiv) and piperidine (0.24 mL, 1.2 equiv) and isolated as a white powder (125 mg, 0.35 mmol, 18% isolated yield). **^1^H NMR (600 MHz, CDCl_3_) δ** 7.55 (dd, *J* = 6.8, 2.2 Hz, 1H), 7.37 (ddd, *J* = 8.4, 4.4, 2.2 Hz, 1H), 7.29 (t, *J* = 8.6 Hz, 1H), 5.50 (s, 2H), 3.84–3.79 (m, 4H), 1.75–1.67 (m, 6H). **^13^C NMR (151 MHz, CDCl_3_) δ** 160.5, 160.1, 159.5 (d, *J* = 253.6 Hz), 159.3, 131.9 (d, *J* = 4.2 Hz), 131.3, 129.0 (d, *J* = 7.8 Hz), 122.1 (d, *J* = 18.1 Hz), 117.4 (d, *J* = 21.9 Hz), 117.3, 116.1, 83.5, 81.7, 49.4, 26.1, 24.5. **MS (ESI^+^) calculated for C_18_H_15_N_5_FCl:** [M + H]^+^*m*/*z* = 356.1, found *m*/*z* = 356.3. **HRMS (ESI^+^) calculated for C_18_H_15_N_5_ClF:** [M + Na]^+^*m*/*z* = 378.0893, found *m*/*z* = 378.0892.

#### 2-Amino-4-(3-chloro-5-fluorophenyl)-6-(piperidin-1-yl)pyridine-3,5-dicarbonitrile (**36**)

The product was prepared following general procedure C starting from 3-chloro-5-fluorobenzaldehyde (317 mg, 2 mmol, 1 equiv) and piperidine (0.24 mL, 1.2 equiv) and isolated as a white powder (136 mg, 0.38 mmol, 19% isolated yield). **^1^H NMR (600 MHz, CDCl_3_) δ** 7.26–7.22 (m, 2H), 7.09 (dt, *J* = 8.1, 1.7 Hz, 1H), 5.51 (s, 2H), 3.84–3.80 (m, 4H), 1.73–1.67 (m, 6H). **^13^C NMR (151 MHz, CDCl_3_) δ** 162.6 (d, *J* = 252.3 Hz), 160.3, 159.7 (d, *J* = 2.3 Hz), 159.2, 137.7 (d, *J* = 8.9 Hz), 136.0 (d, *J* = 10.7 Hz), 124.9 (d, *J* = 3.1 Hz), 118.3, 117.0, 115.8, 114.7 (d, *J* = 23.0 Hz), 83.3, 81.6, 49.4, 26.1, 24.4. **MS (ESI^+^) calculated for C_18_H_15_N_5_FCl:** [M + H]^+^*m*/*z* = 356.1, found *m*/*z* = 356.4. **HRMS (ESI^+^) calculated for C_18_H_15_N_5_ClF:** [M + H]^+^*m*/*z* = 356.1073, found *m*/*z* = 356.1070.

#### 2-Amino-4-(5-chloro-2-fluorophenyl)-6-(piperidin-1-yl)pyridine-3,5-dicarbonitrile (**37**)

The product was prepared following general procedure C starting from 5-chloro-2-fluorobenzaldehyde (79 mg, 0.5 mmol, 1 equiv) and piperidine (0.06 mL, 1.2 equiv) and isolated as a white powder (35 mg, 0.10 mmol, 20% isolated yield). **^1^H NMR (600 MHz, CDCl_3_) δ** 7.45 (ddd, *J* = 8.8, 4.4, 2.7 Hz, 1H), 7.35 (dd, *J* = 6.0, 2.6 Hz, 1H), 7.19 (t, *J* = 8.9 Hz, 1H), 5.36 (s, 2H), 3.85–3.79 (m, 4H), 1.75–1.66 (m, 6H). **^13^C NMR (151 MHz, CDCl_3_) δ** 160.3, 159.2, 157.7 (d, *J* = 250.3 Hz), 155.2, 132.4 (d, *J* = 8.4 Hz), 130.3 (d, *J* = 2.5 Hz), 129.9 (d, *J* = 3.6 Hz), 124.4 (d, *J* = 17.2 Hz), 118.1 (d, *J* = 23.2 Hz), 116.9, 115.7, 84.3, 82.3, 49.2, 26.1, 24.5. **MS (ESI^+^) calculated for C_18_H_15_N_5_ClF:** [M + H]^+^*m*/*z* = 356.1, found *m*/*z* = 356.1. **HMRS (ESI^+^) calculated for C_18_H_15_N_5_ClF:** [M + H]^+^*m*/*z* = 356.1073, found *m*/*z* = 356.1070.

#### 2-Amino-4-(2′-fluoro-[1,1′-biphenyl]-3-yl)-6-(piperidin-1-yl)pyridine-3,5-dicarbonitrile (**38**)

The product was prepared following general procedure A starting from 2′-fluoro-[1,1′-biphenyl]-3-carbaldehyde (100 mg, 0.5 mmol, 1 equiv) and was isolated as a white powder (45 mg, 0.11 mmol, 23% isolated yield). **^1^H NMR (600 MHz, CDCl_3_) δ** 7.72–7.67 (m, 2H), 7.59 (t, *J* = 7.6 Hz, 1H), 7.51 (dt, *J* = 7.7, 1.5 Hz, 1H), 7.44–7.39 (m, 2H), 7.35–7.31 (m, 1H), 7.09–7.03 (m, 1H), 5.38 (s, 2H), 3.88–3.80 (m, 4H), 1.78–1.63 (m, 6H). **^13^C NMR (151 MHz, CDCl_3_) δ** 163.3 (d, *J* = 246.0 Hz), 162.1, 161.3, 159.6, 142.8 (d, *J* = 7.5 Hz), 140.7 (d, *J* = 2.1 Hz), 135.5, 130.5 (d, *J* = 8.4 Hz), 129.5 (d, *J* = 26.6 Hz), 128.2, 127.9, 123.3, 117.9, 116.7, 114.6 (d, *J* = 21.0 Hz), 114.4 (d, *J* = 22.0 Hz), 83.7, 81.8, 49.4, 26.2, 24.6. **MS (ESI^+^) calculated for C_24_H_20_N_5_F:** [M + H]^+^*m*/*z* = 398.2, found *m*/*z* = 398.8. **HRMS (ESI^+^) calculated for C_24_H_20_N_5_F:** [M + Na]^+^*m*/*z* = 420.1595, found *m*/*z* = 420.1594.

#### 2-Amino-4-(3′-fluoro-[1,1′-biphenyl]-3-yl)-6-(piperidin-1-yl)pyridine-3,5-dicarbonitrile (**39**)

The product was prepared following general procedure A starting from 3′-fluoro-[1,1′-biphenyl]-3-carbaldehyde (100 mg, 0.5 mmol, 1 equiv) and was isolated as a white powder (34 mg, 0.08 mmol, 17% isolated yield). **^1^H NMR (600 MHz, CDCl_3_) δ** 7.71 (dq, *J* = 7.7, 1.6 Hz, 1H), 7.68 (q, *J* = 1.6 Hz, 1H), 7.58 (d, *J* = 7.7 Hz, 1H), 7.55–7.49 (m, 2H), 7.37–7.30 (m, 1H), 7.23 (td, *J* = 7.5, 1.2 Hz, 1H), 7.17 (ddd, *J* = 10.9, 8.2, 1.2 Hz, 1H), 5.37 (s, 2H), 3.83–3.79 (m, 4H), 1.71 (t, *J* = 3.1 Hz, 6H). **^13^C NMR (151 MHz, CDCl_3_) δ** 162.0, 161.4, 159.9 (d, *J* = 248.7 Hz), 159.6, 136.5, 135.1, 131.3 (d, *J* = 3.3 Hz), 131.1 (d, *J* = 3.2 Hz), 129.59 (d, *J* = 1.1 Hz), 129.55 (d, *J* = 4.0 Hz), 129.1, 128.1, 124.7 (d, *J* = 3.6 Hz), 117.8, 116.7, 116.3 (d, *J* = 22.5 Hz), 83.8, 81.8, 49.4, 26.1, 24.6. **MS (ESI^+^) calculated for C_24_H_20_N_5_F:** [M + H]^+^*m*/*z* = 398.2, found *m*/*z* = 398.8. **HRMS (ESI^+^) calculated for C_24_H_20_N_5_F:** [M + Na]^+^*m*/*z* = 420.1595, found *m*/*z* = 420.1594.

#### 2-Amino-4-(4′-fluoro-[1,1′-biphenyl]-3-yl)-6-(piperidin-1-yl)pyridine-3,5-dicarbonitrile (**40**)

The product was prepared following general procedure A starting from 4′-fluoro-[1,1′-biphenyl]-3-carbaldehyde (100 mg, 0.5 mmol, 1 equiv) and was isolated as a white powder (34 mg, 0.08 mmol, 17% isolated yield). **^1^H NMR (600 MHz, CDCl_3_) δ** 7.68–7.64 (m, 2H), 7.61–7.55 (m, 3H), 7.48 (dt, *J* = 7.7, 1.5 Hz, 1H), 7.18–7.11 (m, 2H), 5.37 (s, 2H), 3.84–3.79 (m, 4H), 1.76–1.70 (m, 6H). **^13^C NMR (151 MHz, CDCl_3_) δ** 162.9 (d, *J* = 246.7 Hz), 162.2, 161.3, 159.6, 141.0, 136.8 (d, *J* = 3.4 Hz), 135.4, 129.5, 129.3, 129.2 (d, *J* = 8.2 Hz), 127.8 (d, *J* = 19.2 Hz), 117.9, 116.8, 115.9 (d, *J* = 21.5 Hz), 83.7, 81.8, 49.4, 26.2, 24.6. **MS (ESI^+^) calculated for C_24_H_20_N_5_F:** [M + H]^+^*m*/*z* = 398.2, found *m*/*z* = 398.8. **HRMS (ESI^+^) calculated for C_24_H_20_N_5_F:** [M + Na]^+^*m*/*z* = 420.1595, found *m*/*z* = 420.1594.

#### 2-Amino-4-(2′-methyl-[1,1′-biphenyl]-3-yl)-6-(piperidin-1-yl)pyridine-3,5-dicarbonitrile (**41**)

The product was prepared following general procedure D starting from 2-amino-4-(3-bromophenyl)-6-(piperidin-1-yl)pyridine-3,5-dicarbonitrile (60 mg, 0.16 mmol, 1 equiv) and 2-methylphenylboronic acid (63 mg, 3 equiv) and was isolated as a white powder (30 mg, 0.08 mmol, 49% isolated yield). **^1^H NMR (600 MHz, CDCl_3_) δ** 7.55 (t, *J* = 7.7 Hz, 1H), 7.47 (ddt, *J* = 7.4, 5.8, 1.4 Hz, 2H), 7.43 (t, *J* = 1.8 Hz, 1H), 7.32–7.25 (m, 2H), 5.35 (s, 2H), 3.83–3.76 (m, 4H), 2.32 (s, 3H), 1.86–1.66 (m, 6H). **^13^C NMR (151 MHz, CDCl_3_) δ** 162.3, 161.3, 159.6, 142.7, 141.1, 135.8, 134.8, 131.4, 130.4, 130.1, 129.7, 128.7, 127.7, 127.3, 126.0, 117.8, 116.7, 83.9, 81.9, 49.4, 26.1, 24.6, 20.7. **MS (ESI^+^) calculated for C_24_H_21_N_5_:** [M + H]^+^*m*/*z* = 394.2, found *m*/*z* = 394.9. **HRMS (ESI^+^) calculated for C_24_H_21_N_5_:** [M + Na]^+^*m*/*z* = 416.1846, found *m*/*z* = 416.1857.

#### 2-Amino-4-(3′-methyl-[1,1′-biphenyl]-3-yl)-6-(piperidin-1-yl)pyridine-3,5-dicarbonitrile (**42**)

The product was prepared following general procedure D starting from 2-amino-4-(3-bromophenyl)-6-(piperidin-1-yl)pyridine-3,5-dicarbonitrile (60 mg, 0.16 mmol, 1 equiv) and 3-methylphenylboronic acid (63 mg, 3 equiv) and was isolated as a white powder (47 mg, 0.12 mmol, 76% isolated yield). **^1^H NMR (600 MHz, CDCl_3_) δ** 7.70 (ddd, *J* = 7.7, 1.9, 1.2 Hz, 1H), 7.69 (t, *J* = 1.8 Hz, 1H), 7.57 (t, *J* = 7.7 Hz, 1H), 7.47–7.46 (m, 1H), 7.43 (d, *J* = 8.2 Hz, 1H), 7.34 (t, *J* = 7.9 Hz, 1H), 7.18 (d, *J* = 7.2 Hz, 1H), 5.36 (s, 2H), 3.82 (m, 4H), 2.43 (s, 3H), 1.72 (m, 6H). **^13^C NMR (151 MHz, CDCl_3_) δ** 162.4, 161.4, 159.6, 142.2, 140.6, 138.5, 135.2, 129.3, 128.9, 128.5, 128.3, 127.9, 127.5, 124.7, 117.9, 116.8, 83.8, 81.9, 49.4, 26.2, 24.6, 21.7. **MS (ESI^+^) calculated for C_24_H_21_N_5_:** [M + H]^+^*m*/*z* = 394.2, found *m*/*z* = 394.9. **HRMS (ESI^+^) calculated for C_24_H_21_N_5_:** [M + Na]^+^*m*/*z* = 416.1846, found *m*/*z* = 416.1857.

#### 2-Amino-4-(4′-methyl-[1,1′-biphenyl]-3-yl)-6-(piperidin-1-yl)pyridine-3,5-dicarbonitrile (**43**)

The product was prepared following general procedure A starting from 4′-methyl-[1,1′-biphenyl]-3-carbaldehyde (98 mg, 0.5 mmol, 1 equiv) and was isolated as a white powder (56 mg, 0.14 mmol, 28% isolated yield). **^1^H NMR (600 MHz, CDCl_3_) δ** 7.71–7.67 (m, 2H), 7.56 (t, *J* = 7.6 Hz, 1H), 7.53 (d, *J* = 7.9 Hz, 2H), 7.45 (dd, *J* = 7.7, 1.5 Hz, 1H), 7.26 (d, *J* = 8.1 Hz, 2H), 5.37 (s, 2H), 3.84–3.79 (m, 4H), 1.74–1.69 (m, 6H). **^13^C NMR (151 MHz, CDCl_3_) δ** 162.4, 161.4, 159.6, 141.9, 137.8, 137.6, 135.2, 129.7, 129.31, 129.28, 127.7, 127.40, 127.36, 117.9, 116.8, 83.8, 81.8, 49.4, 26.2, 24.6, 21.3. **MS (ESI^+^) calculated for C_24_H_21_N_5_:** [M + H]^+^*m*/*z* = 394.2, found *m*/*z* = 394.8. **HRMS (ESI^+^) calculated for C_24_H_21_N_5_:** [M + Na]^+^*m*/*z* = 416.1846, found *m*/*z* = 416.1857.

#### 2-Amino-6-(piperidin-1-yl)-4-(3-(pyridin-2-yl)phenyl)pyridine-3,5-dicarbonitrile (**44**)

The product was prepared following general procedure F starting from (3-(2-amino-3,5-dicyano-6-(piperidin-1-yl)pyridin-4-yl)phenyl)boronic acid pinacol ester (64 mg, 0.15 mmol, 1 equiv) and 2-bromopyridine (28 mg, 1.2 equiv) and was isolated as a white powder (17 mg, 0.04 mmol, 30% isolated yield). **^1^H NMR (600 MHz, CDCl_3_) δ** 8.92 (s, 1H), 8.65 (s, 1H), 7.99 (dt, *J* = 8.0, 1.8 Hz, 1H), 7.75–7.68 (m, 2H), 7.64 (t, *J* = 7.7 Hz, 1H), 7.56 (dt, *J* = 7.7, 1.4 Hz, 1H), 7.44 (t, *J* = 5.9 Hz, 1H), 5.40 (s, 2H), 3.86–3.80 (m, 4H), 1.76–1.70 (m, 6H). **^13^C NMR (151 MHz, CDCl_3_) δ** 162.0, 161.1, 159.4, 135.4, 129.39, 129.36, 129.0, 127.5, 122.7, 121.1, 117.6, 116.5, 83.7, 81.7, 49.2, 26.0, 24.4. **MS (ESI^+^) calculated for C_23_H_20_N_6_:** [M + H]^+^*m*/*z* = 381.2, found *m*/*z* = 381.0. **HRMS (ESI^+^) calculated for C_23_H_20_N_6_:** [M + H]^+^*m*/*z* = 381.1823, found *m*/*z* = 381.1803.

#### 2-Amino-6-(piperidin-1-yl)-4-(3-(pyridin-3-yl)phenyl)pyridine-3,5-dicarbonitrile (**45**)

The product was prepared following general procedure E at 100 °C for 30 min, starting from 2-amino-4-(3-bromophenyl)-6-(piperidin-1-yl)pyridine-3,5-dicarbonitrile (58 mg, 0.15 mmol, 1 equiv) and 3-pyridineboronic acid (55 mg, 3 equiv) and was isolated as a white powder (79 mg, 0.15 mmol, quantitative isolated yield). **^1^H NMR (600 MHz, CDCl_3_) δ** 8.92 (s, 1H), 8.65 (s, 1H), 7.99 (dt, *J* = 8.0, 1.8 Hz, 1H), 7.75–7.68 (m, 2H), 7.64 (t, *J* = 7.7 Hz, 1H), 7.56 (dt, *J* = 7.7, 1.4 Hz, 1H), 7.44 (t, *J* = 5.9 Hz, 1H), 5.40 (s, 2H), 3.86–3.80 (m, 4H), 1.76–1.70 (m, 6H). **^13^C NMR (151 MHz, CDCl_3_) δ** 161.9, 161.2, 159.6, 149.1, 148.6, 138.7, 136.1, 135.7, 134.9, 129.8, 129.4, 128.5, 127.9, 123.8, 117.9, 116.7, 83.6, 81.8, 49.4, 26.2, 24.6. **MS (ESI^+^) calculated for C_23_H_20_N_6_:** [M + H]^+^*m*/*z* = 381.2, found *m*/*z* = 381.1. **HRMS (ESI^+^) calculated for C_23_H_20_N_6_:** [M + H]^+^*m*/*z* = 381.1823, found *m*/*z* = 381.1836.

#### 2-Amino-6-(piperidin-1-yl)-4-(3-(pyridin-4-yl)phenyl)pyridine-3,5-dicarbonitrile (**46**)

The product was prepared following general procedure E at 80 °C for 15 min, starting from 2-amino-4-(3-bromophenyl)-6-(piperidin-1-yl)pyridine-3,5-dicarbonitrile (58 mg, 0.15 mmol, 1 equiv) and 4-pyridineboronic acid (55 mg, 3 equiv) and was isolated as a white powder (5 mg, 0.013 mmol, 9% isolated yield). **^1^H NMR (600 MHz, CDCl_3_) δ** 8.71–8.67 (m, 2H), 7.79–7.74 (m, 2H), 7.65 (t, *J* = 8.1 Hz, 1H), 7.61–7.58 (m, 3H), 5.41 (s, 2H), 3.86–3.80 (m, 4H), 1.75–1.69 (m, 6H). **^13^C NMR (151 MHz, CDCl_3_) δ** 161.7, 161.2, 159.6, 149.3, 138.6, 136.0, 132.4, 132.3, 130.1, 129.4, 128.8, 128.7, 128.1, 122.6, 117.9, 116.8, 83.6, 81.8, 49.5, 26.3, 24.6. **MS (ESI^+^) calculated for C_23_H_20_N_6_:** [M + H]^+^*m*/*z* = 381.2, found *m*/*z* = 381.1. **HRMS (ESI^+^) calculated for C_23_H_20_N_6_:** [M + H]^+^*m*/*z* = 381.1823, found *m*/*z* = 381.1836.

#### 2-Amino-6-(piperidin-1-yl)-4-(3-(thiophen-2-yl)phenyl)pyridine-3,5-dicarbonitrile (**47**)

The product was prepared following general procedure E at 100 °C for 30 min, starting from 2-amino-4-(3-bromophenyl)-6-(piperidin-1-yl)pyridine-3,5-dicarbonitrile (58 mg, 0.16 mmol, 1 equiv) and 2-thiopheneboronic acid (23 mg, 3 equiv) and was isolated as a white powder (58 mg, 0.16 mmol, quantitative isolated yield). **^1^H NMR (600 MHz, CDCl_3_) δ** 7.74 (ddd, *J* = 7.8, 1.9, 1.1 Hz, 1H), 7.71 (t, *J* = 1.8 Hz, 1H), 7.52 (t, *J* = 7.8 Hz, 1H), 7.39 (ddd, *J* = 7.7, 1.8, 1.1 Hz, 1H), 7.37 (dd, *J* = 3.6, 1.2 Hz, 1H), 7.31 (dd, *J* = 5.1, 1.2 Hz, 1H), 7.09 (dd, *J* = 5.1, 3.6 Hz, 1H), 5.44 (s, 2H), 3.85–3.80 (m, 4H), 1.75–1.69 (m, 6H). **^13^C NMR (151 MHz, CDCl_3_) δ** 162.0, 161.0, 159.4, 143.4, 135.5, 135.2, 129.6, 128.25, 128.16, 127.7, 126.3, 125.6, 124.1, 117.6, 116.5, 83.8, 81.9, 49.5, 26.2, 24.5. **MS (ESI^+^) calculated for C_22_H_19_N_5_S:** [M + H]^+^*m*/*z* = 386.1, found *m*/*z* = 386.1. **HRMS (ESI^+^) calculated for C_22_H_19_N_5_S:** [M + H]^+^*m*/*z* = 386.1435, found *m*/*z* = 386.1438.

#### 2-Amino-6-(piperidin-1-yl)-4-(3-(thiophen-3-yl)phenyl)pyridine-3,5-dicarbonitrile (**48**)

The product was prepared following general procedure E at 80 °C for 15 min, starting from 2-amino-4-(3-bromophenyl)-6-(piperidin-1-yl)pyridine-3,5-dicarbonitrile (58 mg, 0.16 mmol, 1 equiv) and 3-thiopheneboronic acid (23 mg, 1.2 equiv) and was isolated as a white powder (42 mg, 0.11 mmol, 73% isolated yield). **^1^H NMR (600 MHz, CDCl_3_) δ** 7.66–7.62 (m, 2H), 7.46 (t, *J* = 7.7 Hz, 1H), 7.44 (dd, *J* = 2.9, 1.4 Hz, 1H), 7.36–7.33 (m, 2H), 7.32 (dd, *J* = 5.0, 2.9 Hz, 1H), 5.33 (s, 2H), 3.76–3.72 (m, 4H), 1.69–1.62 (m, 6H). **^13^C NMR (151 MHz, CDCl_3_) δ** 162.2, 161.3, 159.6, 141.7, 136.6, 135.4, 129.4, 128.7, 127.5, 127.1, 126.59, 126.55, 121.4, 117.8, 116.7, 83.7, 81.8, 49.4, 26.1, 24.6. **MS (ESI^+^) calculated for C_22_H_19_N_5_S:** [M + Na]^+^*m*/*z* = 408.1, found *m*/*z* = 408.0. **HRMS (ESI^+^) calculated for C_22_H_19_N_5_S:** [M + H]^+^*m*/*z* = 386.1435, found *m*/*z* = 386.1438.

#### 4-(3-(1*H*-Pyrazol-5-yl)phenyl)-2-amino-6-(piperidin-1-yl)pyridine-3,5-dicarbonitrile (**49**)

A Suzuki cross-coupling step was performed following general procedure F starting from (3-(2-amino-3,5-dicyano-6-(piperidin-1-yl)pyridin-4-yl)phenyl)boronic acid pinacol ester (64 mg, 0.15 mmol, 1 equiv) and 5-bromo-1-(tetrahydro-2*H*-pyran-2-yl)-1*H*-pyrazole (42 mg, 1.2 equiv). The THP deprotection of the pyrazole was done in a dry 10 mL round-bottom flask flushed with argon. The intermediate (1 equiv) was solubilized in a 1:1 DCM/MeOH mixture (3 mL). *p*-Toluenesulfonic acid (39 mg, 1.5 equiv) was added, and the solution was stirred at rt for 20 h. Completion was assessed by TLC (*R*_*f*_ = 0.18 in toluene/acetone 8:2). Two successive purifications were performed by flash chromatography on a prepacked 4 g silica cartridge with a (toluene/acetone 4:1)/MeOH isocratic gradient (9:1, 12 CV) then a cyclo/DCM gradient (95:5, 3 CV; 95:5 → 0:10, 18 CV; methanol wash) (42 mg, 0.11 mmol, 76% overall isolated yield). **^1^H NMR (600 MHz, CDCl_3_) δ** 7.88 (dt, *J* = 7.8, 1.4 Hz, 1H), 7.86 (t, *J* = 1.8 Hz, 1H), 7.63 (s, 1H), 7.53 (t, *J* = 7.7 Hz, 1H), 7.44 (dt, *J* = 7.8, 1.3 Hz, 1H), 6.65 (s, 1H), 5.33 (s, 2H), 3.77–3.73 (m, 4H), 1.67–1.62 (m, 6H). **^13^C NMR (151 MHz, CDCl_3_) δ** 161.8, 161.1, 159.5, 135.8, 129.7, 129.2, 128.3, 126.7, 117.7, 116.6, 83.7, 81.8, 49.4, 26.2, 24.6. **MS (ESI^+^) calculated for C_21_H_19_N_7_:** [M + H]^+^*m*/*z* = 370.2, found *m*/*z* = 370.3. **HRMS (ESI^+^) calculated for C_21_H_19_N_7_:** [M + H]^+^*m*/*z* = 370.1775, found *m*/*z* = 370.1779.

#### 4-(3-(1,3,4-Oxadiazol-2-yl)phenyl)-2-amino-6-(piperidin-1-yl)pyridine-3,5-dicarbonitrile (**50**)

In a 25 mL round-bottom flask equipped with a magnetic stirrer and a condenser, methyl 3-(2-amino-3,5-dicyano-6-(piperidin-1-yl)pyridin-4-yl)benzoate (54 mg, 0.15 mmol, 1 equiv) was suspended in anhydrous methanol (1.5 mL). Hydrazine monohydrate (37 mg, 5 equiv) was added to the solution, and then the flask was purged with argon and sealed. The reaction mixture was heated at 80 °C for 16 h. Solvents were removed under reduced pressure, and the crude product was rinsed with DCM/MeOH 7:3 (10 mL) and dried under reduced pressure. In a 25 mL round-bottom flask equipped with a magnetic stirrer and a condenser, the intermediate was suspended in excess triethyl orthoformate (1 mL). The flask was flushed with argon and heated at 100 °C for 20 h. The reaction was quenched by addition of water (20 mL), and the crude product was extracted with EtOAc (3 × 15 mL). The organic phases were combined, washed with brine, dried over MgSO_4_, and filtered, and the solvent was removed under reduced pressure. The crude product was purified by flash chromatography on a 12 g silica cartridge, solid deposit, with a cyclo/EtOAc gradient (8:2, 3 CV; 8:2 → 6:4, 12 CV; 6:4, 6 CV) and isolated as a white powder (32 mg, 0.09 mmol, 58% overall isolated yield). **^1^H NMR (600 MHz, CDCl_3_) δ** 8.50 (s, 1H), 8.26 (dt, *J* = 7.6, 1.6 Hz, 1H), 8.21 (t, *J* = 1.8 Hz, 1H), 7.70 (t, *J* = 7.8 Hz, 1H), 7.67 (dt, *J* = 7.8, 1.6 Hz, 1H), 5.66 (s, 2H), 3.87–3.82 (m, 4H), 1.76–1.70 (m, 6H). **^13^C NMR (151 MHz, CDCl_3_) δ** 164.1, 161.2, 160.2, 159.2, 152.9, 136.0, 132.2, 130.1, 129.1, 127.5, 124.5, 117.2, 115.9, 83.6, 81.6, 49.6, 26.2, 24.4. **MS (ESI^+^) calculated for C_20_H_17_N_7_O:** [M + H]^+^*m*/*z* = 372.2, found *m*/*z* = 372.4. **HRMS (ESI^+^) calculated for C_20_H_17_N_7_O:** [M + H]^+^*m*/*z* = 372.1568, found *m*/*z* = 372.1565.

#### 4-(3-(1*H*-Tetrazol-5-yl)phenyl)-2-amino-6-(piperidin-1-yl)pyridine-3,5-dicarbonitrile (**51**)

The product was prepared following general procedure C starting from 3-(2-(tetrahydro-2*H*-pyran-2-yl)-2*H*-tetrazol-5-yl)benzaldehyde (60 mg, 0.23 mmol, 1 equiv) and purified by flash chromatography on a prepacked 4 g silica cartridge with a cyclo/DCM gradient (95:5, 3 CV; 95:5 → 0:10, 18 CV; methanol wash). The THP deprotection of the tetrazole was performed in the presence of Dowex 50WX8 H^+^ (1 g of resin for 100 mg of compound), in a 98:2 EtOH/H_2_O mixture (1 mL) at 80 °C for 16 h. The resin was filtered off and rinsed with warm EtOH (3 × 2 mL), and after solvent evaporation, the product was isolated as a white powder (10 mg, 0.03 mmol, 11% overall isolated yield). **^1^H NMR (600 MHz, CD_3_OD) δ** 8.22 (dt, *J* = 7.8, 1.4 Hz, 1H), 8.19 (t, *J* = 1.8 Hz, 1H), 7.63 (t, *J* = 7.7 Hz, 1H), 7.51 (ddd, *J* = 7.6, 1.9, 1.2 Hz, 1H), 3.82 (dd, *J* = 6.4, 4.1 Hz, 3H), 1.78–1.67 (m, 7H). **^13^C NMR (151 MHz, CD_3_OD) δ** 163.6, 162.9, 162.0, 161.8, 137.6, 131.7, 130.2, 130.0, 129.1, 128.0, 118.9, 117.2, 83.4, 82.5, 50.2, 27.2, 25.6. **MS (ESI^–^) calculated for C_19_H_17_N_9_:** [M – H]^−^*m*/*z* = 370.2, found *m*/*z* = 370.0. **HRMS (ESI^–^) calculated for C_19_H_17_N_9_:** [M – H]^−^*m*/*z* = 370.1524, found *m*/*z* = 370.1500.

#### 2-Amino-6-chloro-4-phenylpyridine-3,5-dicarbonitrile (**52**)

In a dry 25 mL round-bottom flask equipped with a stirrer and a condenser, trimethyl orthoformate (0.86 mL, 5.0 mmol) and malononitrile (0.66 g, 2 equiv) were solubilized in pyridine (0.40 mL, 1 equiv). The reaction was stirred under argon at 100 °C for 1 h and monitored by TLC until complete consumption of the starting orthoformate. The resulting dark red mixture was cooled in an ice bath, and 37% HCl was added dropwise under vigorous stirring (*Caution!* fumes). The mixture was heated to 80 °C for 2 h. The crude product was cooled in an ice bath, diluted with water (10 mL), and extracted by EtOAc (3 × 15 mL). The organic phases were combined, washed with brine, dried over MgSO_4_, and filtered, and the solvent was removed under reduced pressure. The crude product was stirred with cold Et_2_O, and the resulting solid was filtered, rinsed with cold Et_2_O (2 × 2 mL), and dried under reduced pressure for 24 h. The crude powder was used without further purification (224 mg, 0.88 mmol, 18% isolated yield). **^1^H NMR (600 MHz, (CD_3_)_2_SO) δ** 7.62–7.56 (m, 2H), 7.49–7.43 (m, 1H), 7.44–7.38 (m, 2H), 5.68 (s, 2H). **^13^C NMR (151 MHz, (CD_3_)_2_SO) δ** 186.9, 137.9, 130.6, 128.0, 127.5, 120.6, 119.1.

#### 2′-Fluoro-[1,1′-biphenyl]-3-carbaldehyde (**53**)

The product was prepared following general procedure C starting from 3-bromobenzaldehyde (0.12 mL, 1.0 mmol, 1 equiv) and 2-fluorophenylboronic acid (276 mg, 2 equiv) and was isolated as a translucent oil (135 mg, 0.68 mmol, 68% isolated yield). **^1^H NMR (600 MHz, CDCl_3_) δ** 10.09 (s, 1H), 8.06 (q, *J* = 1.9 Hz, 1H), 7.90 (dt, *J* = 7.7, 1.4 Hz, 1H), 7.83 (dtd, *J* = 7.7, 1.7, 1.2 Hz, 1H), 7.62 (t, *J* = 7.7 Hz, 1H), 7.48 (td, *J* = 7.7, 1.8 Hz, 1H), 7.38 (dddd, *J* = 8.3, 7.4, 5.0, 1.8 Hz, 1H), 7.25 (td, *J* = 7.5, 1.2 Hz, 1H), 7.19 (ddd, *J* = 10.8, 8.3, 1.2 Hz, 1H). **^13^C NMR (151 MHz, CDCl_3_) δ** 192.3 (d, *J* = 1.5 Hz), 159.9 (d, *J* = 248.6 Hz), 137.0, 136.8, 135.1 (d, *J* = 3.3 Hz), 130.8 (d, *J* = 3.2 Hz), 130.5 (d, *J* = 2.8 Hz), 129.9 (d, *J* = 8.2 Hz), 129.3, 128.9, 127.8 (d, *J* = 13.1 Hz), 124.8 (d, *J* = 3.7 Hz), 116.4 (d, *J* = 22.5 Hz).

#### 3′-Fluoro-[1,1′-biphenyl]-3-carbaldehyde (**54**)

The product was prepared following general procedure C starting from 3-bromobenzaldehyde (0.12 mL, 1.0 mmol, 1 equiv) and 3-fluorophenylboronic acid (276 mg, 2 equiv) and was isolated as a translucent oil (154 mg, 0.77 mmol, 77% isolated yield). **^1^H NMR (600 MHz, CDCl_3_) δ** 10.10 (s, 1H), 8.11–8.06 (m, 1H), 7.89 (dt, *J* = 7.6, 1.4 Hz, 1H), 7.84 (ddd, *J* = 7.7, 2.0, 1.2 Hz, 1H), 7.63 (t, *J* = 7.6 Hz, 1H), 7.46–7.40 (m, 2H), 7.33 (ddd, *J* = 9.9, 2.6, 1.7 Hz, 1H), 7.13–7.08 (m, 1H). **^13^C NMR (151 MHz, CDCl_3_) δ** 192.2 (d, *J* = 1.8 Hz), 163.4 (d, *J* = 246.2 Hz), 142.1 (d, *J* = 7.7 Hz), 141.1 (d, *J* = 2.3 Hz), 137.2, 133.1, 130.7 (d, *J* = 8.4 Hz), 129.8, 129.4, 128.2, 123.0 (d, *J* = 3.0 Hz), 115.0 (d, *J* = 21.1 Hz), 114.3 (d, *J* = 22.2 Hz).

#### 4′-Fluoro-[1,1′-biphenyl]-3-carbaldehyde (**55**)

The product was prepared following general procedure C starting from 3-bromobenzaldehyde (0.12 mL, 1.0 mmol, 1 equiv) and 4-fluorophenylboronic acid (276 mg, 2 equiv) and was isolated as a translucent oil (115 mg, 0.58 mmol, 58% isolated yield). **^1^H NMR (600 MHz, CDCl_3_) δ** 10.09 (s, 1H), 8.06 (t, *J* = 1.8 Hz, 1H), 7.86 (dt, *J* = 7.6, 1.4 Hz, 1H), 7.82 (ddd, *J* = 7.7, 2.0, 1.2 Hz, 1H), 7.63–7.57 (m, 3H), 7.19–7.14 (m, 2H). **^13^C NMR (151 MHz, CDCl_3_) δ** 192.4 (d, *J* = 2.0 Hz), 163.0 (d, *J* = 247.7 Hz), 141.4, 137.1, 136.0 (d, *J* = 3.2 Hz), 133.0, 129.7, 129.0 (d, *J* = 8.5 Hz), 128.9, 128.0, 116.1 (d, *J* = 21.7 Hz).

#### 2′-Methyl-[1,1′-biphenyl]-3-carbaldehyde (**56**)

The product was prepared following general procedure C starting from 3-bromobenzaldehyde (0.12 mL, 1.0 mmol, 1 equiv) and *o*-tolylboronic acid (202 mg, 1.5 equiv) and was isolated as a translucent oil (29 mg, 0.15 mmol, 15% isolated yield). **^1^H NMR (600 MHz, CDCl_3_) δ** 10.09 (s, 1H), 8.10 (t, *J* = 1.8 Hz, 1H), 7.87–7.83 (m, 2H), 7.60 (t, *J* = 7.6 Hz, 1H), 7.47–7.41 (m, 2H), 7.37 (t, *J* = 7.6 Hz, 1H), 7.22 (ddt, *J* = 7.5, 1.9, 1.0 Hz, 1H), 2.44 (s, 3H). **^13^C NMR (151 MHz, CDCl_3_) δ** 192.5, 142.5, 139.8, 138.8, 137.0, 133.2, 129.6, 129.1, 128.9, 128.7, 128.4, 128.1, 124.4, 21.7.

#### 3′-Methyl-[1,1′-biphenyl]-3-carbaldehyde (**57**)

The product was prepared following general procedure C starting from 3-bromobenzaldehyde (0.12 mL, 1.0 mmol, 1 equiv) and *m*-tolylboronic acid (202 mg, 1.5 equiv) and was isolated as a translucent oil (69 mg, 0.35 mmol, 35% isolated yield). **^1^H NMR (600 MHz, CDCl_3_) δ** 10.07 (s, 1H), 7.87 (dt, *J* = 6.8, 1.9 Hz, 1H), 7.85 (td, *J* = 1.6, 0.8 Hz, 1H), 7.63–7.56 (m, 2H), 7.32–7.27 (m, 3H), 7.23 (dt, *J* = 7.3, 1.2 Hz, 1H), 2.27 (s, 3H). **^13^C NMR (151 MHz, CDCl_3_) δ** 192.5, 192.5, 143.1, 140.5, 136.5, 135.4, 130.7, 130.6, 129.8, 129.0, 128.3, 128.1, 126.2, 20.5.

#### 4′-Methyl-[1,1′-biphenyl]-3-carbaldehyde (**58**)

The product was prepared following general procedure C starting from 3-bromobenzaldehyde (0.12 mL, 1.0 mmol, 1 equiv) and *p*-tolylboronic acid (202 mg, 1.5 equiv) and was isolated as a translucent oil (113 mg, 0.58 mmol, 58% isolated yield). **^1^H NMR (600 MHz, CDCl_3_) δ** 10.09 (s, 1H), 8.09 (t, *J* = 1.8 Hz, 1H), 7.86–7.83 (m, 2H), 7.60 (t, *J* = 7.6 Hz, 1H), 7.56–7.51 (m, 2H), 7.32–7.27 (m, 2H), 2.42 (s, 3H). **^13^C NMR (151 MHz, CDCl_3_) δ** 192.6, 142.3, 138.1, 136.9, 133.0, 129.9, 129.6, 128.5, 128.1, 127.1, 21.3.

#### 3-(Tetrazol-5-yl)benzaldehyde (**59**)

In a 100 mL round-bottom flask equipped with a magnetic stirrer and a condenser, the 3-cyanobenzaldehyde (393 mg, 3.0 mmol, 1 equiv) was solubilized in DMF (1 mL) and then diluted in water (75 mL). Both sodium azide (292 mg, 1.5 equiv) and zinc chloride (408 mg, 3 equiv) were added. The suspension was vigorously stirred and heated at 110 °C for 48 h. The reaction completion was assessed by TLC (*R*_*f*_ = 0.08 in DCM/MeOH 98:2) before being cooled in an ice bath. The precipitate was filtered, rinsed with ice-cold water (2 × 10 mL) and then with diethyl ether (2 × 5 mL), and dried under reduced pressure. The crude salts were then solubilized in aqueous 1 M HCl (10 mL) and extracted by EtOAc (3 × 15 mL) to remove the zinc salts. The crude powder could be used without further purification (220 mg, 1.3 mmol, 42% isolated yield). **^1^H NMR (600 MHz, (CD_3_)_2_SO) δ** 10.13 (s, 1H), 8.57 (t, *J* = 1.7 Hz, 1H), 8.36 (dt, *J* = 7.8, 1.5 Hz, 1H), 8.12 (dt, *J* = 7.7, 1.4 Hz, 1H), 7.86 (t, *J* = 7.7 Hz, 1H). **^13^C NMR (151 MHz, (CD_3_)_2_SO) δ** 192.7, 136.9, 132.4, 132.1, 130.4, 127.3. **MS (ESI^+^) calculated for C_8_H_6_N_4_O:** [M – H]^−^*m*/*z* = 173.0, found *m*/*z* = 173.0.

#### 3-(2-(Tetrahydro-2*H*-pyran-2-yl)-2*H*-tetrazol-5-yl)benzaldehyde (**60**)

In a dry 25 mL round-bottom flask equipped with a magnetic stirrer and a condenser, 3,4-dihydro-2*H*-pyrane (0.18 mL, 2 equiv) was solubilized in toluene (5 mL) and mixed with a solution of 3-(tetrazol-5-yl)benzaldehyde (174 mg, 1.0 mmol, 1 equiv) in DMF (0.79 mL). TFA (0.008 mL, 0.1 equiv) was added dropwise. The flask was purged with argon and heated at 110 °C for 24 h. The reaction progress was assessed by TLC (*R*_*f*_ = 0.57 in DCM/MeOH 98:2) before being quenched by the addition of a 10% Na_2_CO_3_ solution (10 mL) and water (20 mL). The crude product was extracted by EtOAc (3 × 15 mL), and the organic phases were combined, washed with brine (20 mL), dried over MgSO_4_, and filtered, and the solvents were removed under reduced pressure. The purification was done by flash chromatography on a 12 g silica cartridge with a cyclo/DCM gradient (8:2, 3 CV; 8:2 → 0:10, 6 CV; methanol wash), and the product was isolated as an oil (66 mg, 0.26 mmol, 26% isolated yield). **^1^H NMR (600 MHz, CDCl_3_) δ** 10.11 (s, 1H), 8.70 (t, *J* = 1.8 Hz, 1H), 8.47 (dt, *J* = 7.7, 1.5 Hz, 1H), 8.01 (dt, *J* = 7.7, 1.4 Hz, 1H), 7.68 (t, *J* = 7.7 Hz, 1H), 6.10 (dd, *J* = 7.6, 2.9 Hz, 1H), 4.08–4.02 (m, 1H), 3.88–3.80 (m, 1H), 2.57–2.46 (m, 1H), 2.25–2.15 (m, 2H), 1.88–1.71 (m, 3H). **^13^C NMR (151 MHz, CDCl_3_) δ** 191.81, 164.1, 137.1, 132.7, 130.8, 129.9, 129.0, 128.6, 88.2, 67.1, 29.2, 24.7, 20.9. **MS (ESI^–^) calculated for C_13_H_14_N_4_O_2_:** [M + Cl]^−^*m*/*z* = 293.1, found *m*/*z* = 293.1.

### cAMP Measurement Assay (CamBio) Protocol

CHO cells expressing A_2A_R and the biosensor were seeded in a black 384-well plate (Nunc) and grown at 37 °C and 5% CO_2_ overnight. They were preincubated for 6 min with compounds at varied concentrations up to 30 μM, before addition of the corresponding EC_80_ of A_2A_R agonist, 400 nM adenosine (Sigma). The cAMP biosensor allowed real-time monitoring of signals using an FDSS/μCELL (Hamamatsu). The assay was conducted in 1× Hanks Balanced Salt Solution (HBSS). The total volume of the reaction was 80 μL (45 μL of cells, 15 μL of antagonist, and 20 μL of agonist). Data analysis was performed using GraphPad Prism.

### NAM Mode-of-Action Confirmation (Progressive Fold-Shift Assay) Protocol

CHO cells expressing A_2A_R and the biosensor were seeded in a black 384-well plate (Nunc) and grown at 37 °C and 5% CO_2_ overnight. They were preincubated for 6 min with compounds at varied concentrations up to 30 μM, before adding adenosine at increasing concentrations up to 1 mM (CRC). The cAMP biosensor readings and assay volumes were as described in the CamBio assay. Data analysis was performed using GraphPad Prism.

A Schild regression plot was calculated for the compounds using the progressive fold-shift assay data. The log(Dose Ratio – 1) was plotted on the *y* axis against the log of the antagonist concentration on the *x* axis.

### Binding Cooperativity Factor α and Dissociation Constant *K*_B_ Determination

Using the data from the progressive fold-shift assay, α and *K*_B_ were calculated directly in GraphPad Prism using the nonlinear regression equation “allosteric EC_50_ shift, X is log(concentration)” as per instructions.

### Interaction Analysis by Grating-Coupled Interferometry (GCI)

Kinetic binding measurements were performed using GCI on a Creoptix WAVEdelta system (Creoptix, a Malvern Panalytical brand). A PCP-NTA Creoptix WAVEchip (sensor chip) was conditioned according to the manufacturer’s specifications. The surface was activated by a 120 s injection of 0.5 mM NiCl_2_ at a flow rate of 10 mL/min on 2 parallel flow channels. Purified A_2A_R was injected at 100 mg/mL in running buffer (20 mM Tris-HCl pH 7.5, 350 mM NaCl, 0.1% (w/v) *n*-dodecyl-β-d-maltopyranoside (DDM), 2% (v/v) dimethyl sulfoxide (DMSO) at a flow rate of 1 mL/min to capture the receptor via its C-terminal His-tag to a level of 5500 pg/mm^2^. The surface of both channels was stabilized with several injections of the running buffer. The kinetic measurements of XAC binding to A_2A_R were performed using A-B-A type injections, ensuring that tested compounds were present at 100 nM, 1 μM, or 10 μM, respectively, during the full measurement from baseline throughout XAC dissociation. XAC was injected in a dilution series from 1.6 to 200 nM for 60 s at a flow rate of 50 mL/min, followed by dissociation in running buffer for 120 s.

Data analysis was performed using the Creoptix WAVEcontrol software (Creoptix, a Malvern Panalytical brand). The response signals were double referenced, subtracting the signal recorded on the reference channel with no protein as well as the signal of blank injections with running buffer. Double-referenced signals were fit to a 1:1 Langmuir binding model, determining association rate (*k*_a_), dissociation rate (*k*_d_), maximum response (*R*_max_) and the dissociation constant (*K*_D_) for each interaction. Single injection cycles showing significant distortions or no binding response were excluded from the analysis.

### pCREB Measurement Protocol

PBMCs were isolated from healthy human whole blood. Thawed PBMCs were incubated with A_2A_R antagonists in the presence of NECA and were subsequently fixed and permeabilized using ice cold paraformaldehyde and methanol-based kit reagents (ThermoFisher and Miltenyi, respectively). Cells were washed with FACS buffer and stained with fluorochrome-conjugated antibodies directed against the following antigens: pCREB (Miltenyi), CD4 and CD8 (Life Technologies), and CD3 (BD Biosciences). Stained cells were acquired using a Fortessa flow cytometer (BD). Gating of the desired cell subsets was performed using FSC-A vs FSC-H to remove doublet cells and FSC vs SSC parameters to identify cells with lymphocyte-like morphology, followed by CD3^+^ T cell and CD4^+^ or CD8^+^ subgating. The mean fluorescence intensity (MFI) fold change in intracellular pCREB in gated CD4^+^ or CD8^+^ T cells was calculated using the DMSO-only condition and then normalized with the NECA-only condition. The results shown are an average of several experiments done on different donors, with a minimum of two repetitions for each donor.

### Caco-2 Bidirectional Permeability Assay

Caco-2 cells seeded onto polyethylene membranes (PET) in 96-well Corning Insert plates were incubated with the compounds (2 μM in HBSS with 10 mM HEPES, pH 7.4, 1% DMSO) for 2 h at 37 °C without shaking. All apical (A) and basolateral (B) samples were collected, mixed with ACN, and centrifugated. The Caco-2 monolayer integrity was confirmed by yellow luciferase rejection assay. Compound concentrations were analyzed by HPLC-MS/MS, and the apparent permeability coefficient (*P*_app_) was calculated using the following formula:
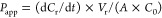
d*C*_r_/d*t* is the concentration of compound in the receiver chamber as a function of time (μM/s); *V*_r_ is the solution volume in the receiver chamber; *A* is the surface area for the transport; *C*_0_ is the initial concentration in the donor chamber (μM).

### Microsomal Stability Assay

Human or mouse liver microsomes in 100 mM potassium phosphate buffer were preincubated 10 min at 37 °C. Compound **45** (10 μM in ACN/DMSO 99:1) and then the NADPH cofactor solution were added to start the reaction. The final concentrations were 0.5 mg/mL of microsomes, 1 μM compound **45**, and 1 mM NADPH. The reaction was stopped by the addition of ACN, and the precipitated proteins were separated by centrifugation prior to the quantitative HPLC-MS/MS analysis. The compound’s stability was monitored over a 1 h time course. The results were plotted as % remaining compound over time.

## Supporting Information Available

Supporting figures; Progressive fold-shift assay plots; ^1^H NMR, ^13^C NMR, and HPLC analysis results of target compounds (PDF)
Molecular formula strings with biological data (CSV)

## Abbreviations

A_2A_R: Adenosine 2A receptor
ADME: absorption, distribution, metabolism and excretion
BCS: biopharmaceutics classification system
CHO: Chinese hamster ovary
CV: column volume
DCM: dichloromethane
4-DMAP: 4-dimethylaminopyridine
DMF: dimethylformamide
DMSO: dimethyl sulfoxide
ESI: electrospray ionization
GCI: grating-coupled interferometry
GPCR: G-protein coupled receptor
(HR)MS: (high resolution) mass spectrometry
MFI: mean fluorescence intensity
NADPH: nicotinamide adenine dinucleotide phosphate, reduced form
NAM: negative allosteric modulator
NECA: 5′-*N*-ethylcarboxamidoadenosine
NMR: nuclear magnetic resonance
PBMC: peripheral blood mononuclear cell
*P*_app_: apparent permeability coefficient
SAR: structure–activity relationship
TFA: trifluoroacetic acid
THF: tetrahydrofuran
THP: tetrahydropyran
TLC: thin layer chromatography
TME: tumor microenvironment
TOF: time of flight
