# Supplementary material for: Discovery of the First Efficacious Adenosine 2A Receptor Negative Allosteric Modulators for High Adenosine Cancer Immunotherapies
Source: J Med Chem. 2025 Jan 24;68(4):4059–78. doi: 10.1021/acs.jmedchem.4c01691 (PMC11873987; doi:10.1021/acs.jmedchem.4c01691)
Supplement: Supplementary file 1 — jm4c01691_si_001.pdf [file jm4c01691_si_001.pdf]

Supporting information for

Discovery of the first-efficacious $A_{2A}R$ negative allosteric modulators for high adenosine cancer immunotherapies

Margot Boujut[1,2 ‡], Margaux Héritier[1,2 ‡], Aurélie Gouiller[1,2], Camille Süess[1,2], Alessandro Scapozza[1], Thibaut De Smedt[1 †], Maxime Guibert[1], Sébastien Tardy[1,2], Hesham Hamed[1,2,3], David Pejoski[1,2,3] & Leonardo Scapozza[1,2,3] *

[1] School of Pharmaceutical Sciences, University of Geneva, 1206 Geneva, Switzerland

[2] Institute of Pharmaceutical Sciences of Western Switzerland, University of Geneva, 1206 Geneva, Switzerland

[3] Adoram Therapeutics, 1212 Grand-Lancy, Switzerland

* Correspondence should be addressed to Prof. Leonardo Scapozza Leonardo.Scapozza@unige.ch.

Supporting figures....S2
Supporting tables....S4
HPLC traces for selected examples....S9
^1H & ^{13}C NMR spectra for target compounds....S23
References....S70

Supporting figures

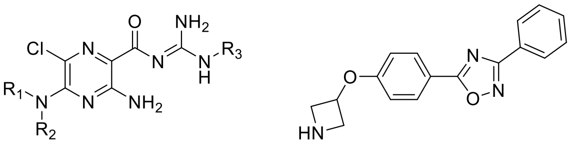

amiloride derivatives   Fg754

Figure S1. Structures of the known $A_{2A}R$ NAMs.[1,2]

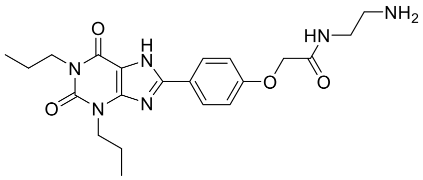

Figure S2. Structure of XAC (xanthine amine congener).[3,4]

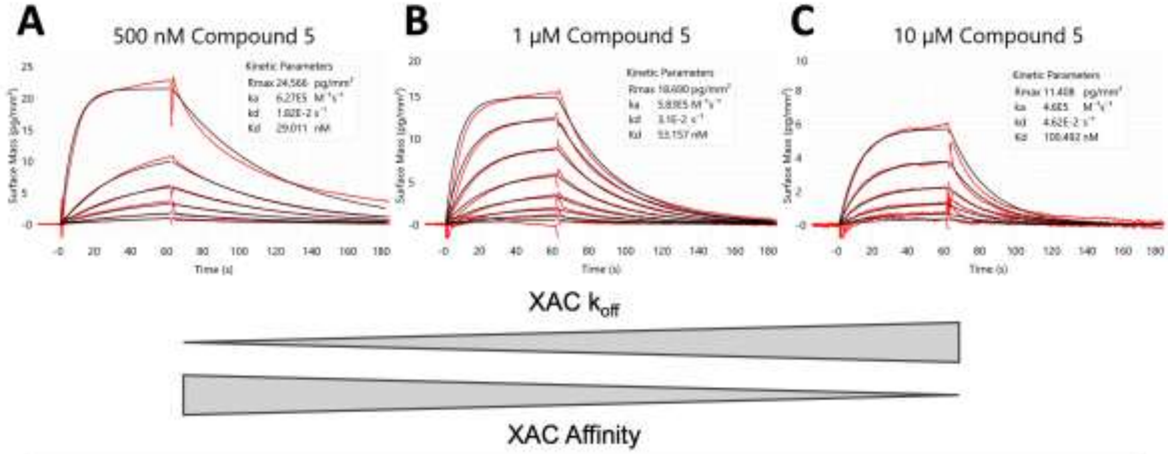

fixed [compound 5] (μM)	K_D (M)	k_{off} (s^{-1})	k_{on} (M^{-1} s^{-1})	R_{max} (pg/mm²)
0.5	2.90×10^{-8}	1.82×10^{-2}	6.27×10^{5}	24.57
1	5.31×10^{-8}	3.10×10^{-2}	5.83×10^{5}	18.69
10	1.00×10^{-7}	4.62×10^{-2}	4.60×10^{5}	11.41

Figure S3. GCI kinetic characterization of XAC binding to $A_{2A}R$ at different concentrations of compound ***5****: (A) 500 nM, (B) 1 μM and (C) 10 μM. Double-referenced binding signals are shown in red, 1:1 Langmuir interaction model fits are shown in black. Table: Kinetic parameters derived from fitting double-referenced binding signals to a 1:1 interaction model at two concentrations of compound* ***5****.*

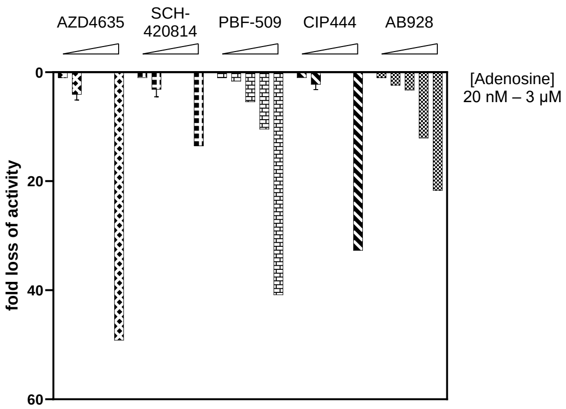

Figure S4. Fold loss of $A_{2A}R$ activity at increasing adenosine concentrations for some known $A_{2A}R$ orthosteric inhibitors; given relative to their activity in presence of 20 nM of adenosine.

Supporting tables

Table S1. Biological effects and mode-of-action confirmation in CamBio assay of known $A_{2A}R$ orthosteric inhibitors.

Compound	IC_{50} (nM)[1]	%inhibition @10 µM[1]	%activity @10 µM on parental cell line[2]	Progressive fold-shift assay[3]
AZD4635	6.0	118%	n.a.	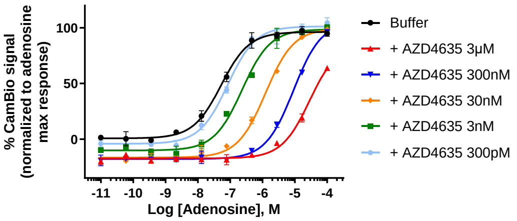
SCH-420814	2.6	118%	n.a.	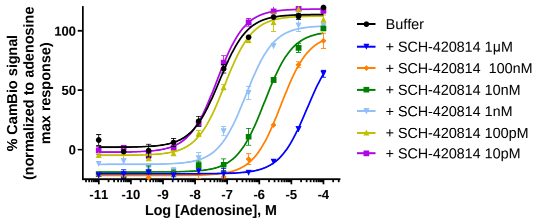
PBF-509	220	111%	n.a.	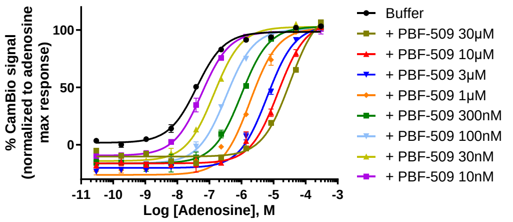

CPI444	7.8	117%	n.a.	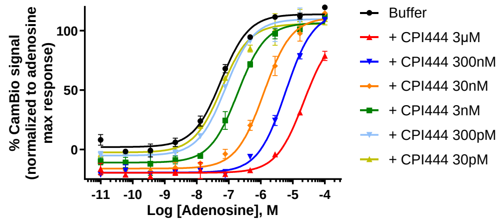
AB928	2.1	117%	n.a.	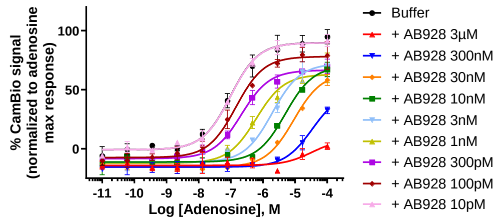

[1] IC_{50} (nM) in presence of 400 nM of adenosine and % inhibition at 10 µM values are given by CamBio assay on CHO cells expressing $A_{2A}R$, experimental repetitions N = 2-5.

[2] Specificity is given by CamBio assay on the parental CHO cell, experimental repetitions N = 2-5.

3 All Schild plots analysis derived from the progressive fold-shift assays have been generated and present the same linear profile as the one of AZD4635, Figure 6H.

Table S2. Progressive fold-shift assay plots for selected examples

Compound	Progressive fold-shift assay plot
Compound 4	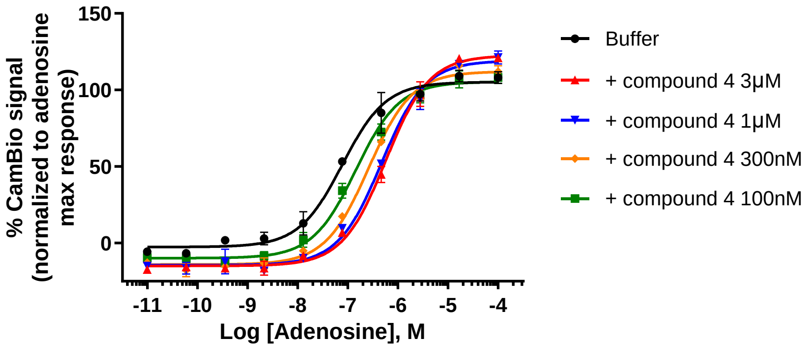

<table>
<tr><td>Compound 38</td><td>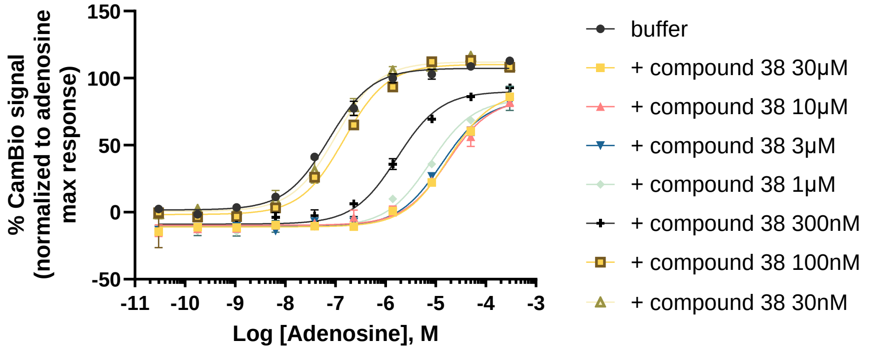
</td></tr>
<tr><td>Compound 39</td><td>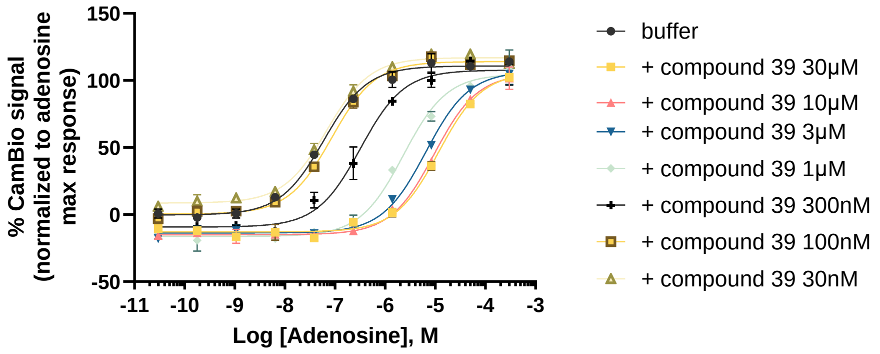
</td></tr>
<tr><td>Compound 41</td><td>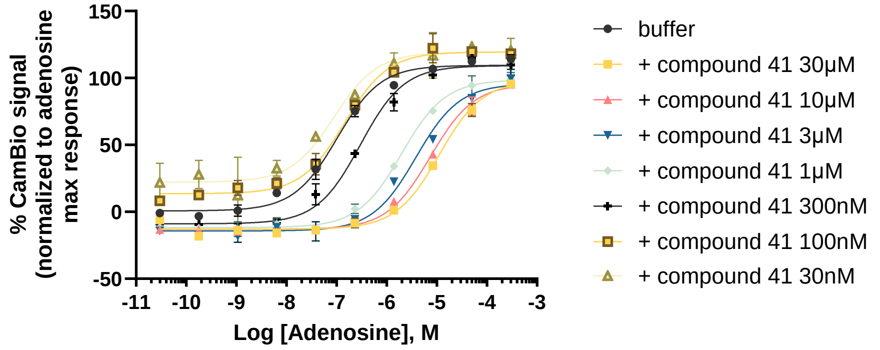
</td></tr>
<tr><td>Compound 42</td><td>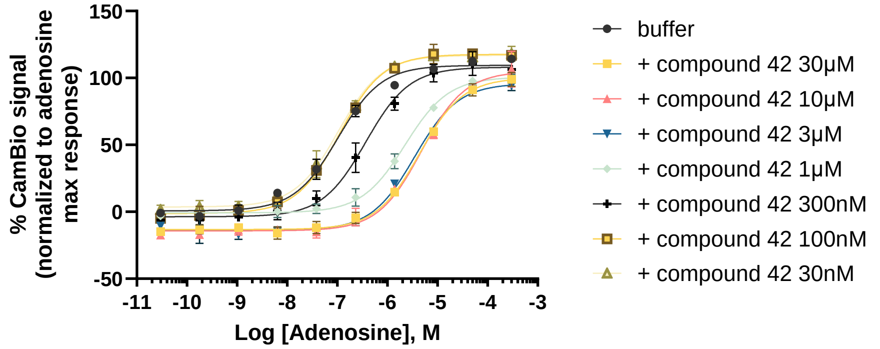
</td></tr>
</table>

<table>
<tr><td>Compound 44</td><td>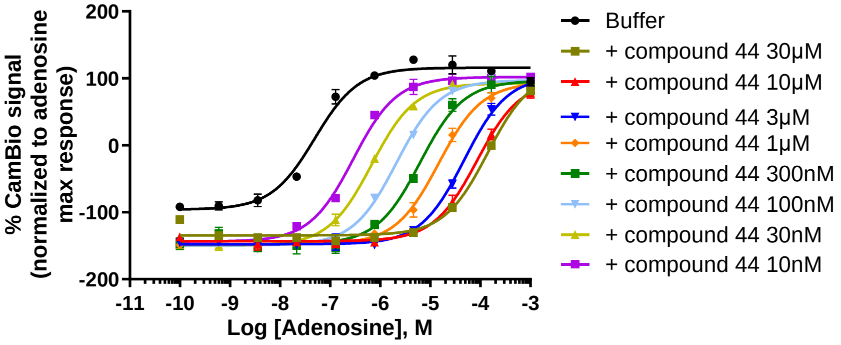
</td></tr>
<tr><td>Compound 46</td><td>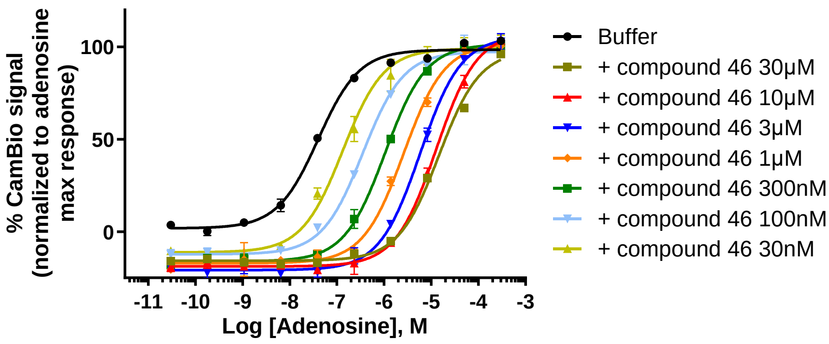
</td></tr>
<tr><td>Compound 47</td><td>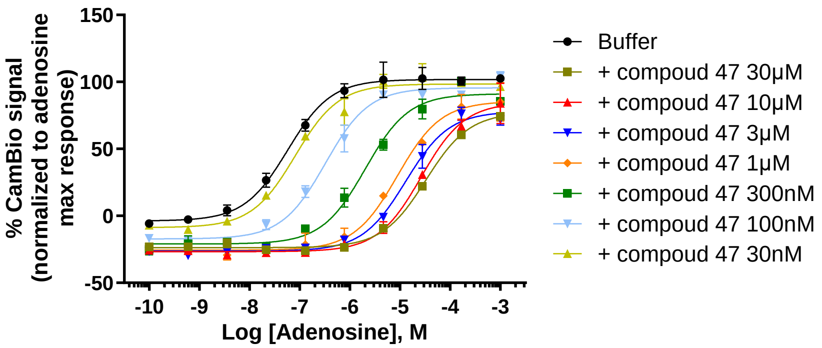
</td></tr>
<tr><td>Compound 48</td><td>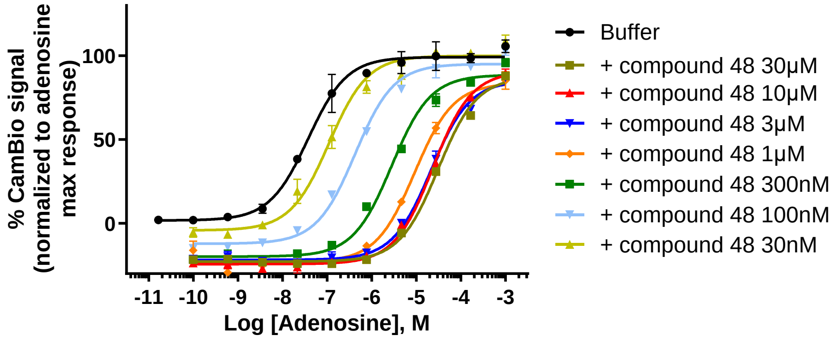
</td></tr>
</table>

Table S3. Bidirectional permeability measurements *in vitro* using Caco-2 cell monolayers as a model of gut barrier permeability.

Compound	Mean P_{app} (10^{-6} cm/s)		Mean %solution recovery		Efflux ratio	P_{app} rank	Efflux transporter substrate
	A to B	B to A	A to B	B to A			
**45**	3.18	3.98	21%[1]	52%	1.25	High	Poor to none

[1] *The insufficient recovery (%solution recovery values < 50.0) may be caused by cellular retention, metabolism, adsorption or other issues.*

Table S4. *In vitro* mice and human liver microsomal stability data with respect to testosterone.

Compound	Mice liver microsomes			Human liver microsomes		
	$t_{½}$ (min)	$CL_{int(mic)}$ (μL/min/mg)[1]	$CL_{int(liver)}$ (mL/min/kg)[2]	$t_{½}$ (min)	$CL_{int(mic)}$ (μL/min/mg)[1]	$CL_{int(liver)}$ (mL/min/kg)[2]
**45**	1.5	947	3749	1.5	911	820
**testosterone**	4.2	334	1322	11.6	120	108

[1] *$CL_{int(mic)}$: intrinsic clearance*

[2] *$CL_{int(liver)} = CL_{int(mic)} \times$ mg microsomal protein/g liver weight × g liver weight/kg body weight*

HPLC traces for selected examples

Compound 5:

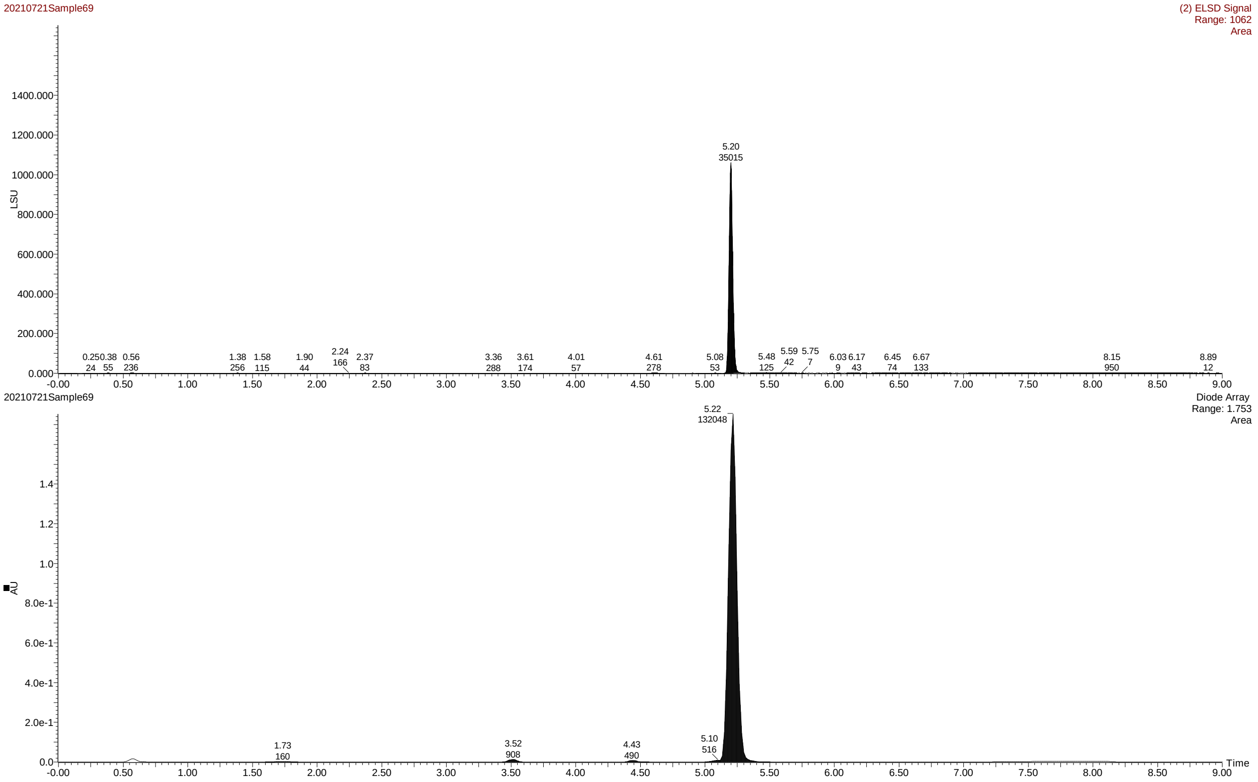

UV retention time: 5.22 min

UV purity: 98.4%

UV rt (min)	UV peak height	UV peak area
1.733	1402	160.017
3.517	12478	907.966
4.433	7463	490.216
5.100	8535	516.309
5.217	1752576	132047.500

Compound 11:

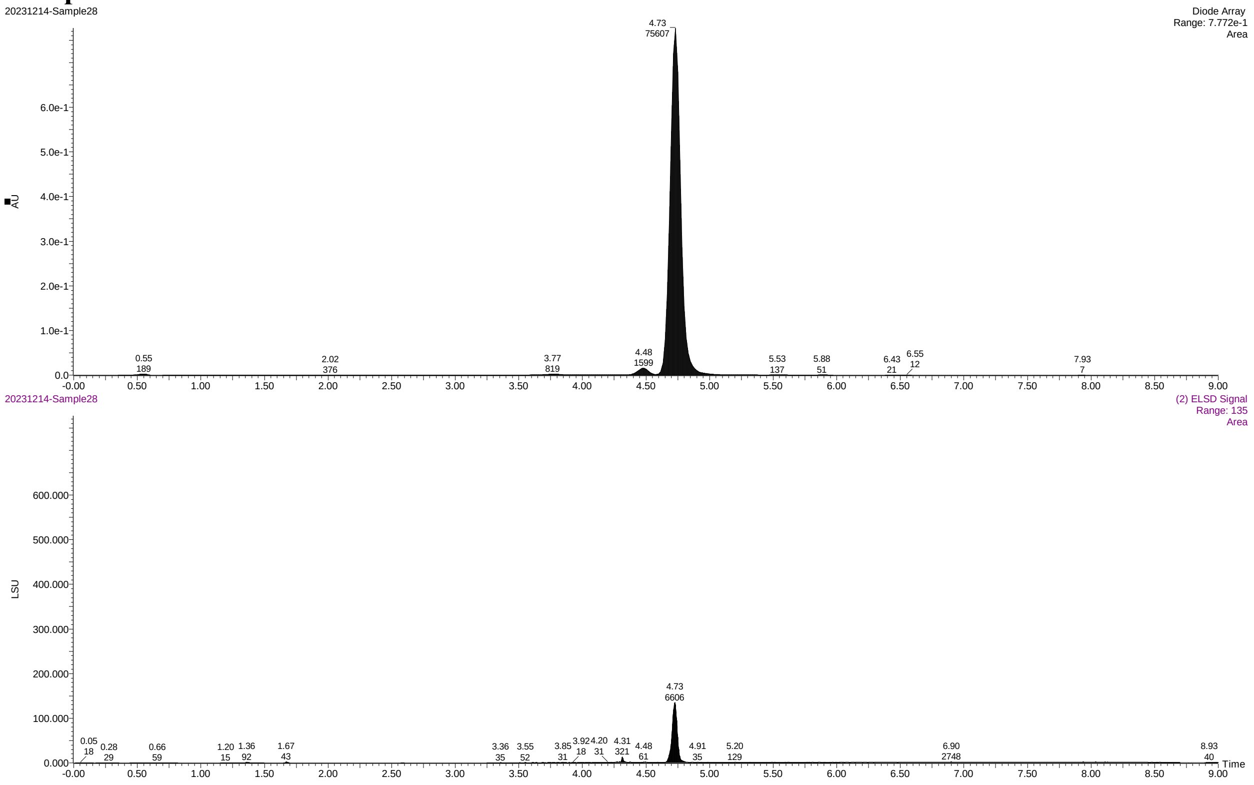

UV retention time (rt): 4.73 min

UV purity: 96.2%

UV rt (min)	UV peak height	UV peak area
2.017	296	376.017
3.767	2594	819.133
4.483	15429	1598.768
4.733	777216	75606.875
5.533	798	137.008
5.883	605	51.275
6.433	336	21.383
6.550	223	12.300

Compound 13:

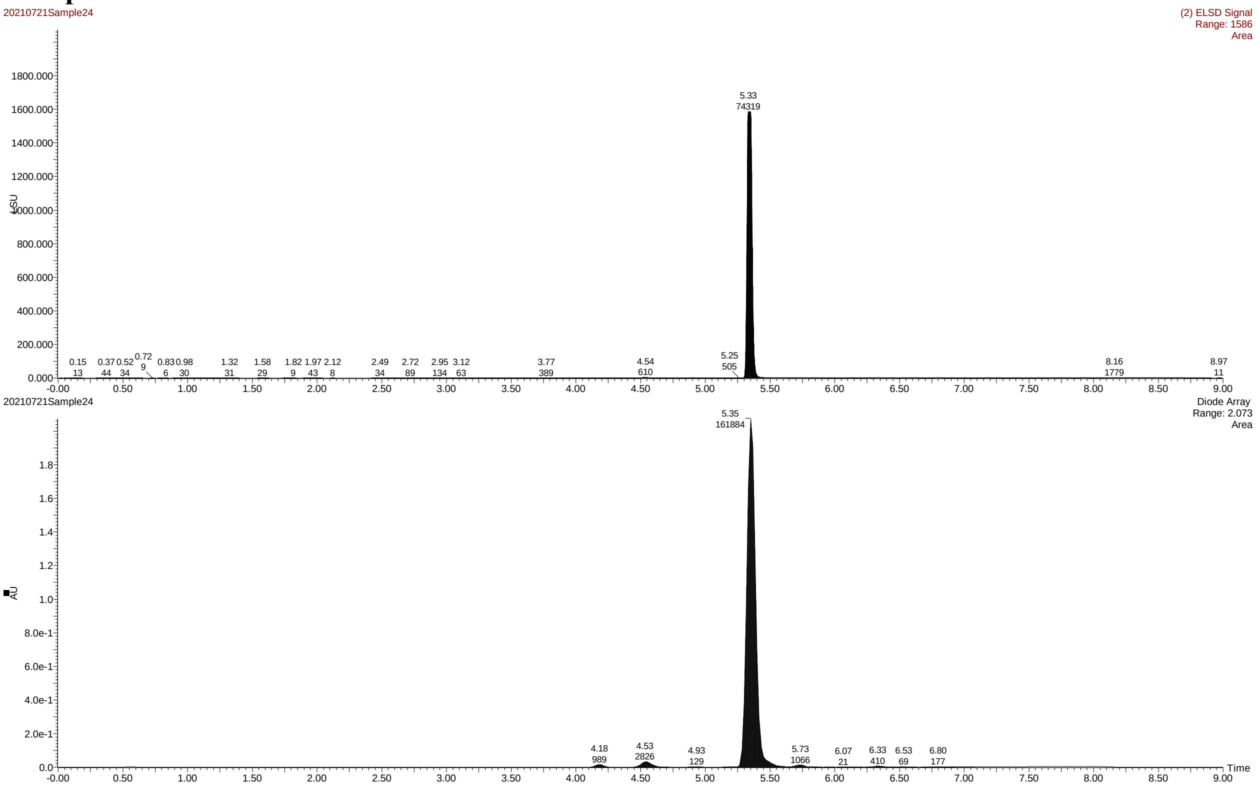

UV retention time: 5.35 min

UV purity: 96.6%

UV rt (min)	UV peak height	UV peak area
4.183	14805	989.066
4.533	31161	2826.334
4.933	2113	129.183
5.350	2072896	161883.547
5.733	11682	1066.234
5.950	92	3.683
6.067	261	20.508
6.333	5530	409.616
6.533	950	68.850
6.800	1434	176.613

Compound 14:

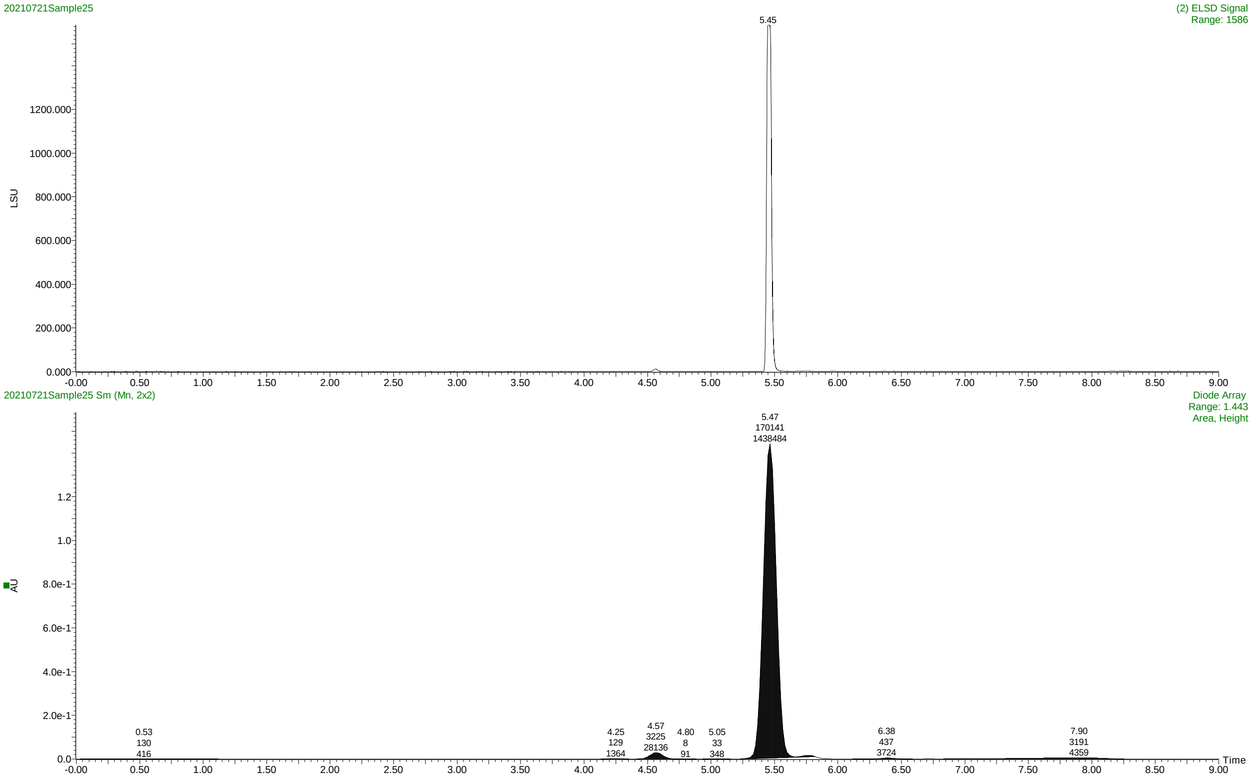

UV retention time: 5.47 min

UV purity: 95.0%

#	Name	RT	Area
**1**	compound	5.47	171011344
**2**	compound impurity 1	4.57	3226215
**3**	compound impurity 2	5.77	2259913
**4**	compound impurity 3	7.90	4010548

Compound 15:

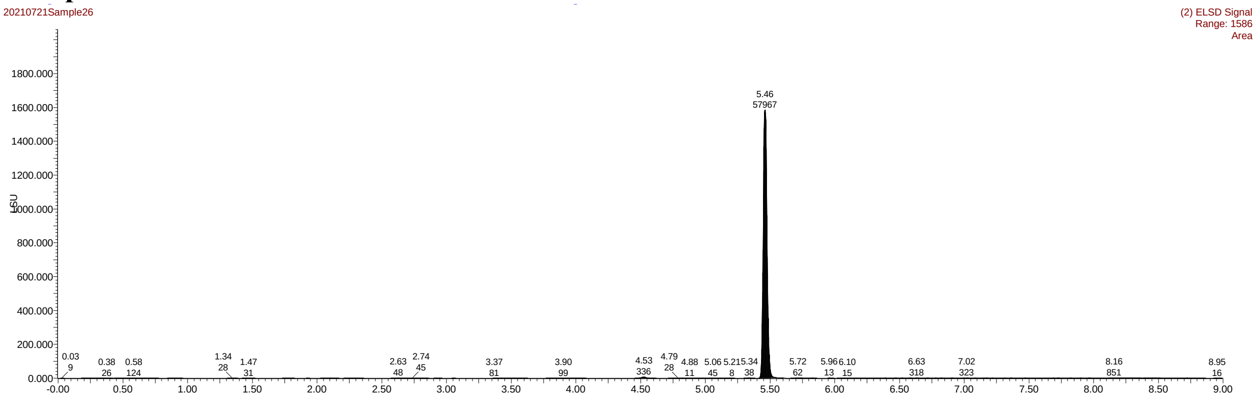

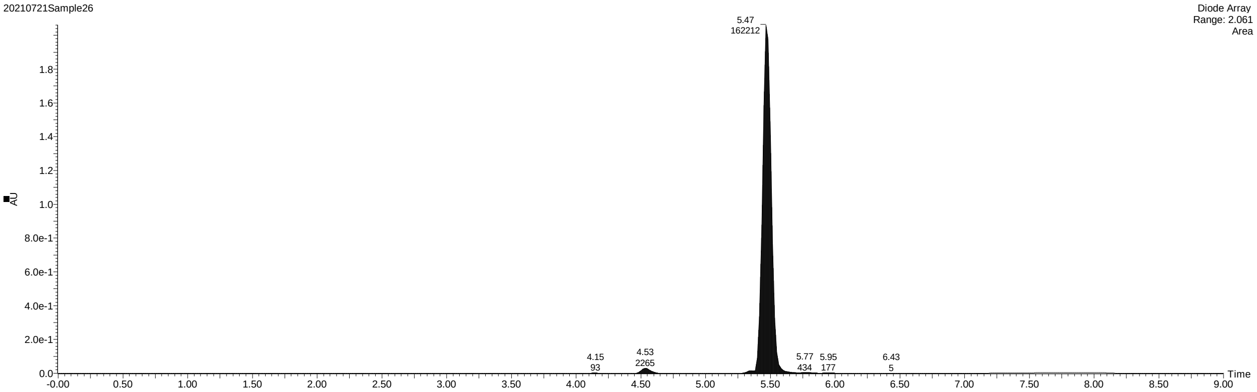

UV retention time: 5.47 min

UV purity: 98.2%

UV rt (min)	UV peak height	UV peak area
4.150	2246	93.150
4.533	29396	2265.468
5.467	2060672	162212.297
5.767	4618	434.258
5.950	2422	177.092
6.433	156	5.167

Compound 28:

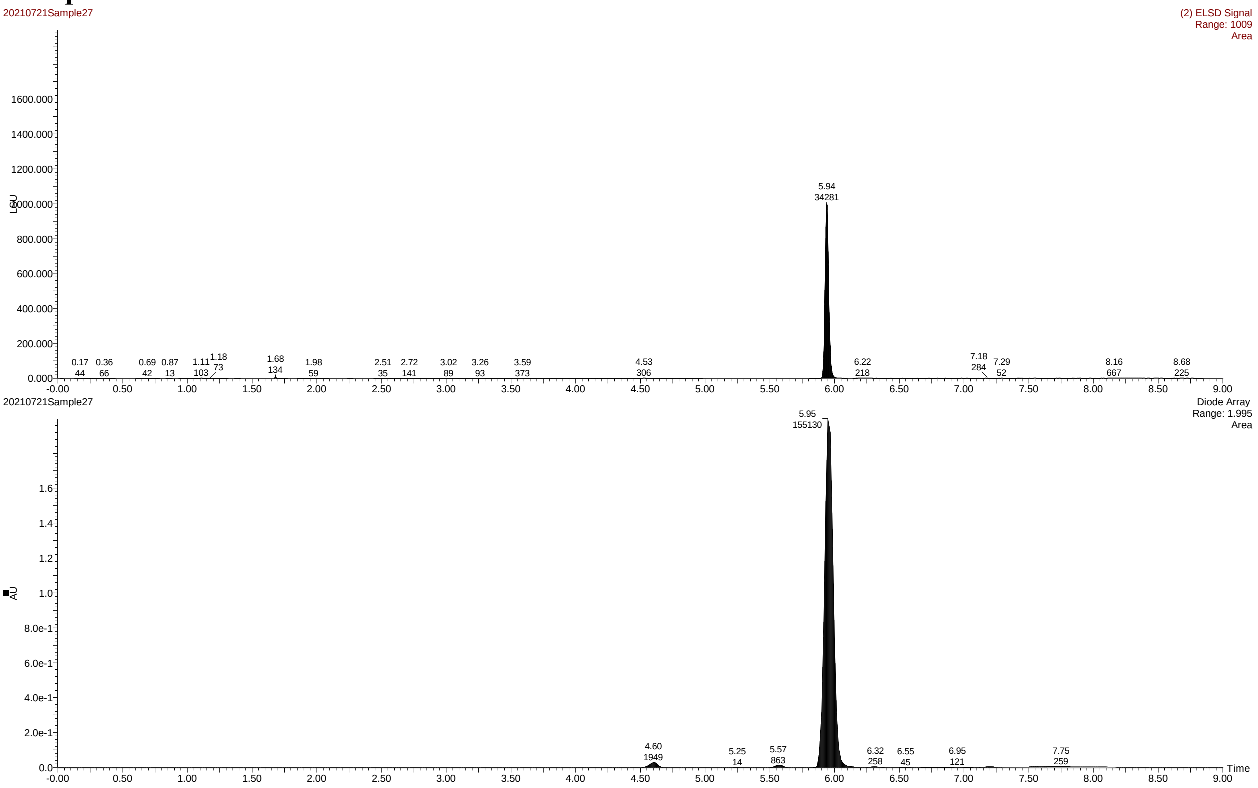

UV retention time: 5.95 min

UV purity: 97.8%

UV rt (min)	UV peak height	UV peak area
4.600	27780	1949.298
5.250	411	13.650
5.567	13004	862.633
5.950	1995328	155129.531
6.317	3430	258.200
6.550	903	45.100
6.950	1147	121.399
7.750	694	258.875

Compound 33:

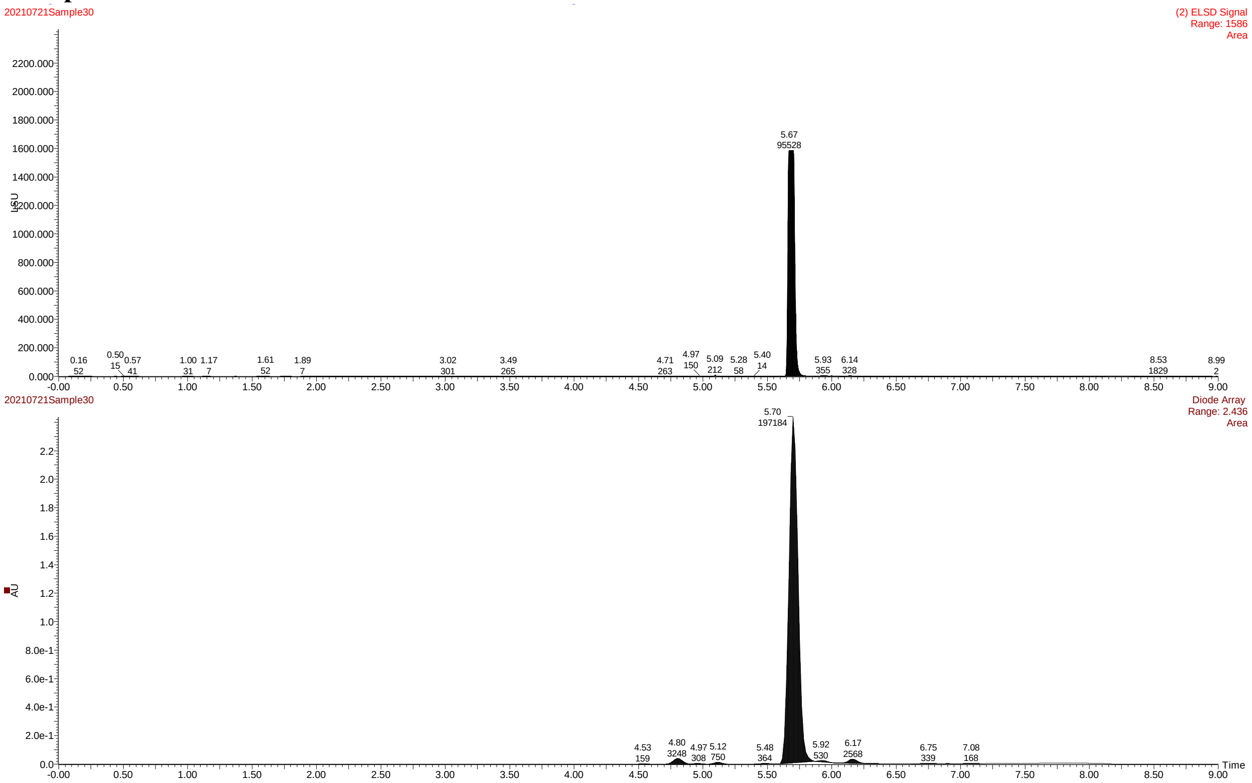

UV retention time: 5.70 min

UV purity: 96.1%

UV rt (min)	UV peak height	UV peak area
4.533	3517	159.183
4.800	38700	3247.774
4.967	4771	308.142
5.117	11409	750.383
5.483	4194	364.134
5.700	2427022	197183.859
5.917	7687	529.880
6.167	29927	2567.550

Compound 44:

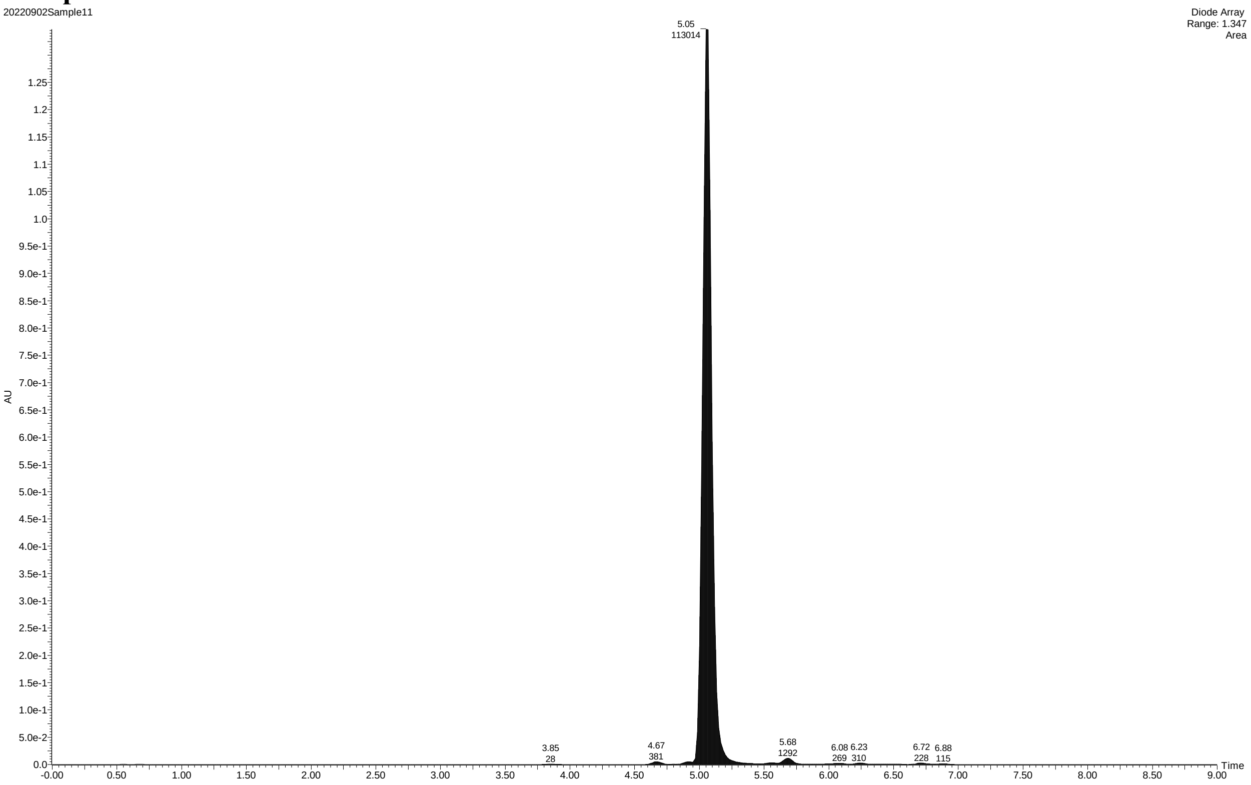

UV retention time: 5.05 min

UV purity: 97.7%

UV rt (min)	UV peak height	UV peak area
3.850	410	27.900
4.667	5091	381.200
5.050	1346926	113014.359
5.684	11398	1291.777
6.084	1863	269.141
6.234	2277	309.534
6.717	2457	227.516
6.884	1507	114.600

Compound 45:

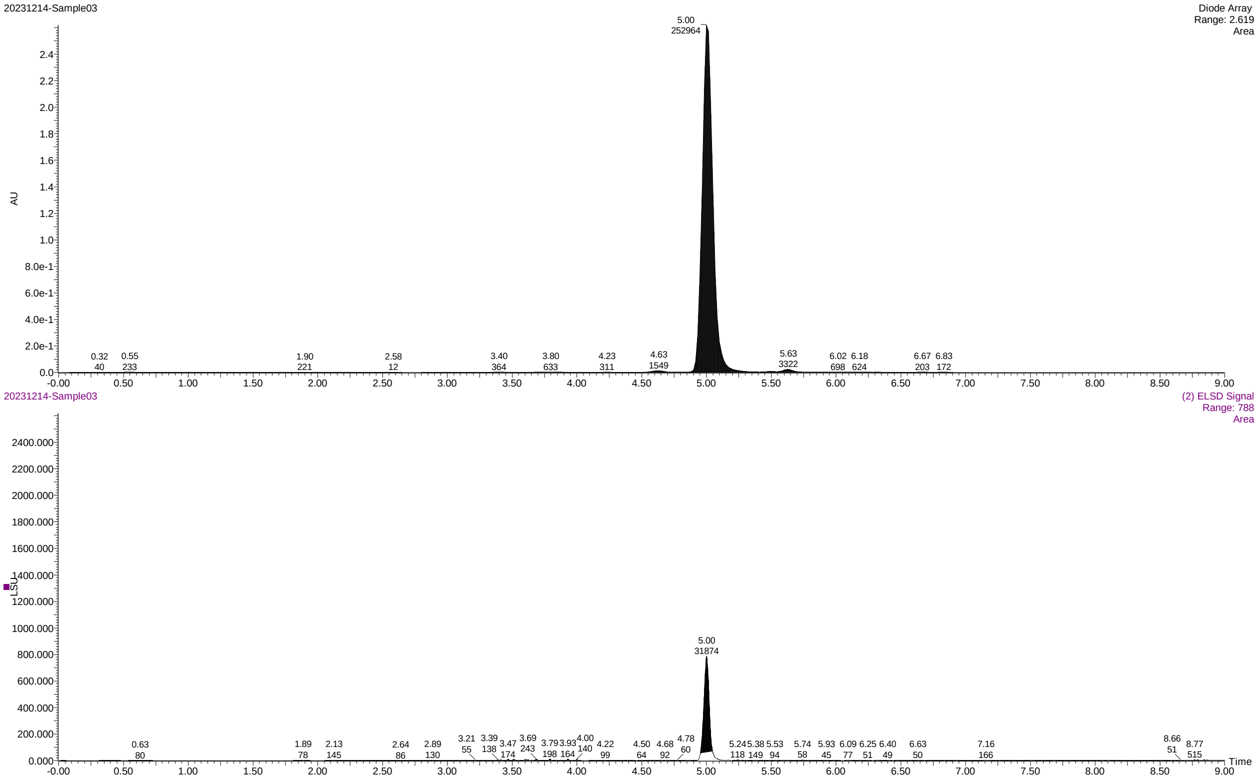

UV retention time (rt): 5.00 min

UV purity: 97.0%

UV rt (min)	UV peak height	UV peak area
1.900	219	220.883
2.583	150	12.117
3.400	1772	364.408
3.800	2446	633.387
4.233	1445	310.883
4.633	12797	1548.844
5.000	2618577	252963.563
5.633	22441	3321.590
6.017	3703	697.804
6.183	3475	624.450
6.667	2059	203.033

Compound 46:

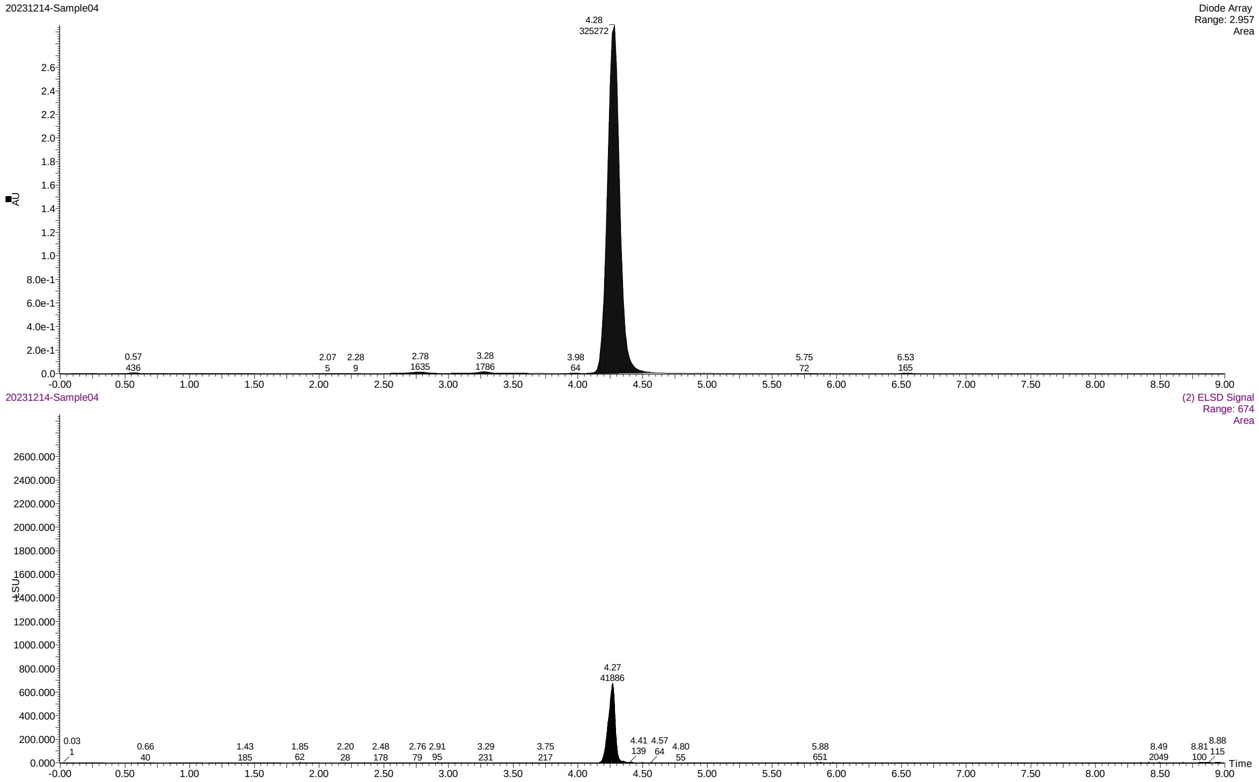

UV retention time: 4.28 min

UV purity: 98.8%

UV rt (min)	UV peak height	UV peak area
0.750	41	2.684
1.383	28	12.042
2.067	59	4.524
2.283	51	9.226
2.783	11288	1634.858
3.283	13041	1786.324
3.983	891	63.900
4.283	2953111	325271.969
5.750	833	72.367
6.533	2597	164.633

Compound 47:

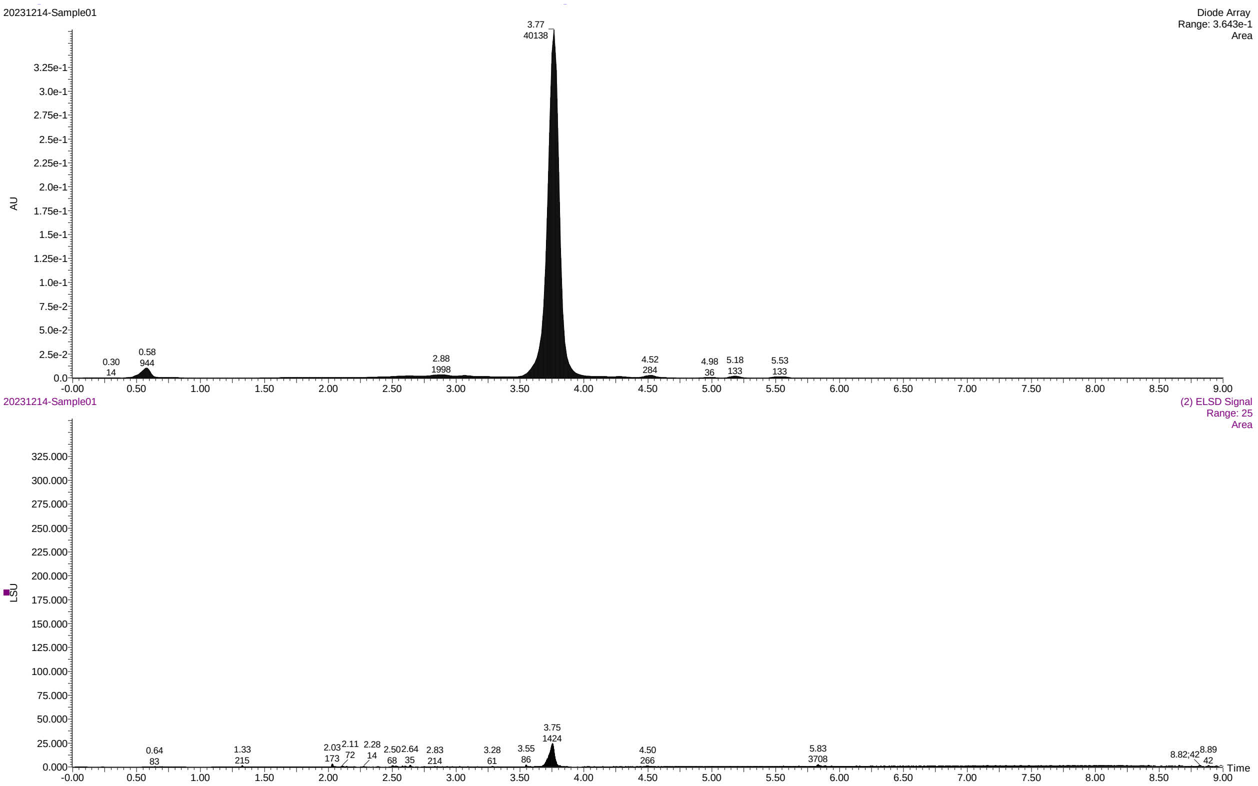

UV retention time: 3.77 min

UV purity: 96.0%

UV rt (min)	UV peak height	UV peak area
2.883	2385	1093.150
3.767	363191	39098.125
4.517	2237	218.458
4.983	544	36.233
5.183	1747	133.017
5.533	1232	132.617

Compound 48:

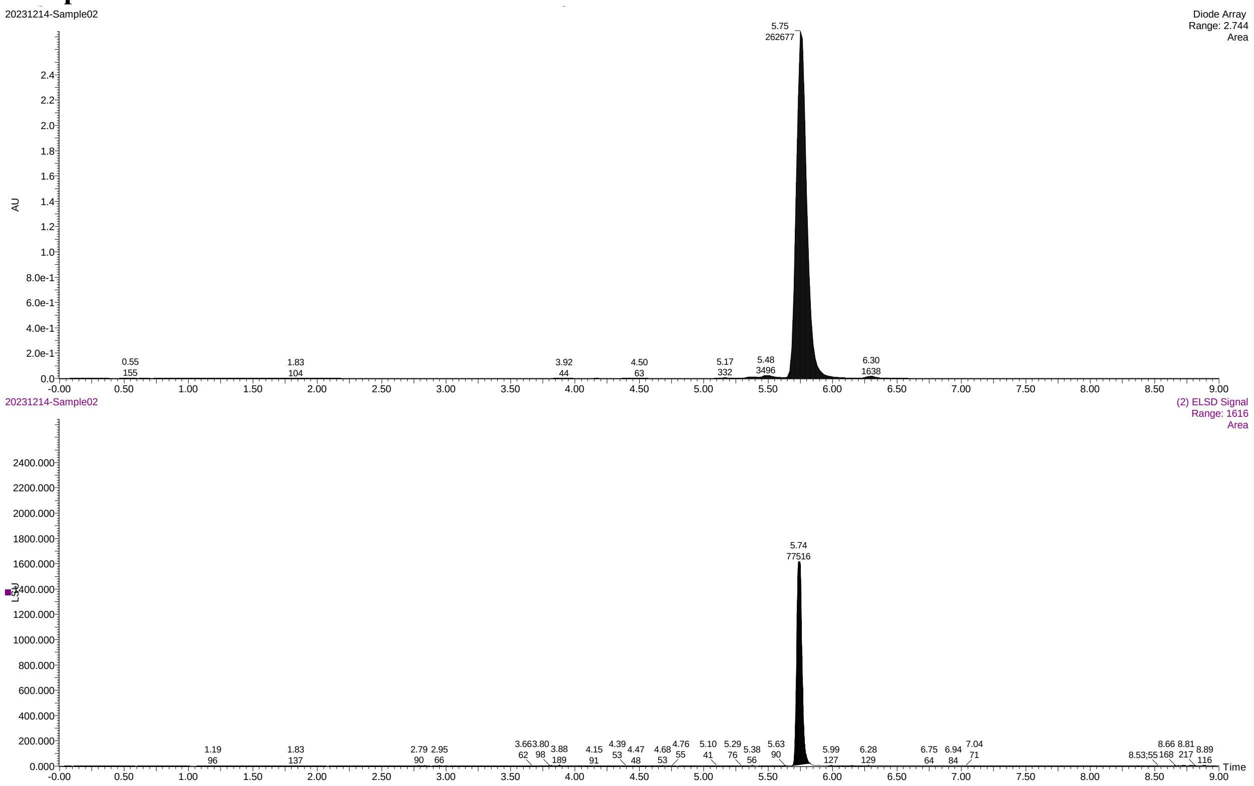

UV retention time (rt): 5.75 min

UV purity: 97.8%

UV rt (min)	UV peak height	UV peak area
0.650	64	2.783
1.833	146	103.950
3.917	647	44.100
4.167	13	0.217
4.500	778	63.250
5.167	4258	331.550
5.483	21699	3496.273
5.750	2743930	262677.031
6.300	15876	1637.712

Compound 49:

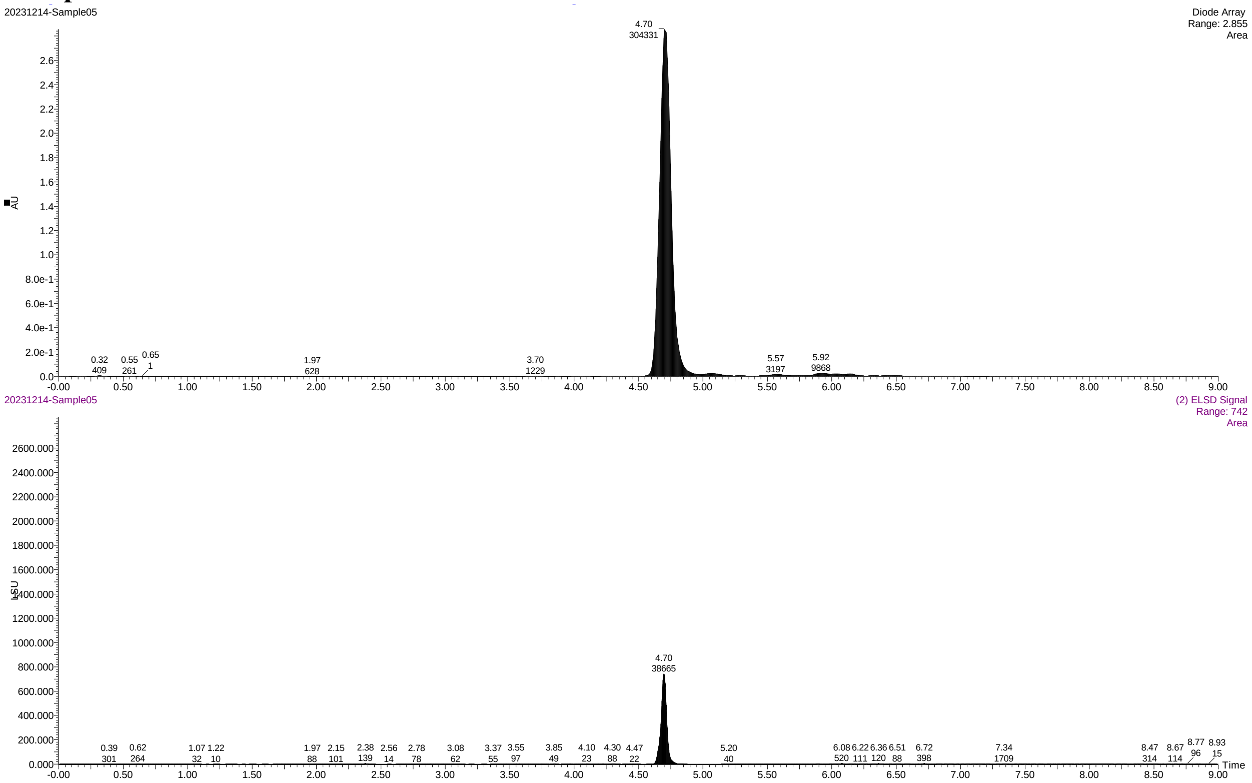

UV retention time (rt): 4.70 min

UV purity: 95.3%

UV rt (min)	UV peak height	UV peak area
0.650	29	0.783
1.967	441	627.643
3.700	2723	1229.253
4.700	2855391	304331.125
5.567	16487	3196.939
5.917	25647	9868.385

Compound 50:

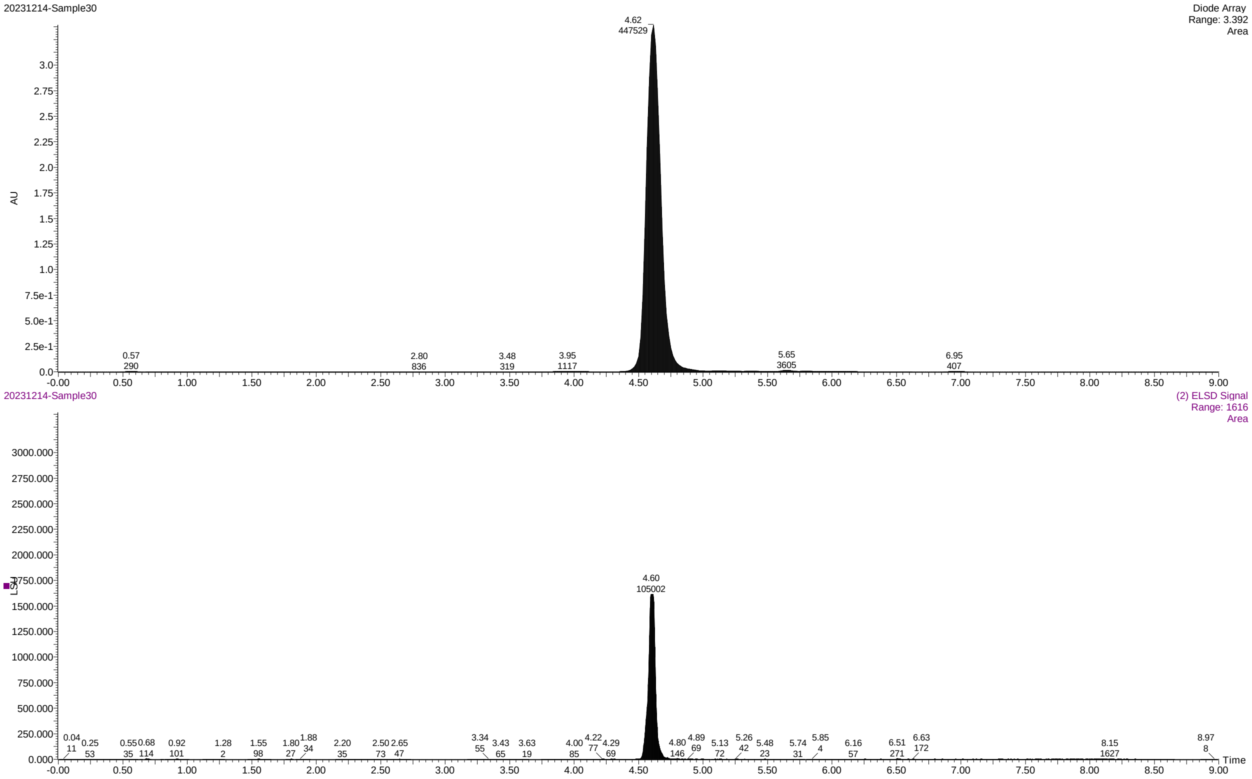

UV retention time (rt): 4.62 min

UV purity: 98.7%

UV rt (min)	UV peak height	UV peak area
2.800	1369	836.411
3.483	1256	319.470
3.950	3687	1117.057
4.617	3392246	447528.813
5.650	14945	3604.956

^{1}H & ^{13}C NMR spectra for target compounds

Compound 5:

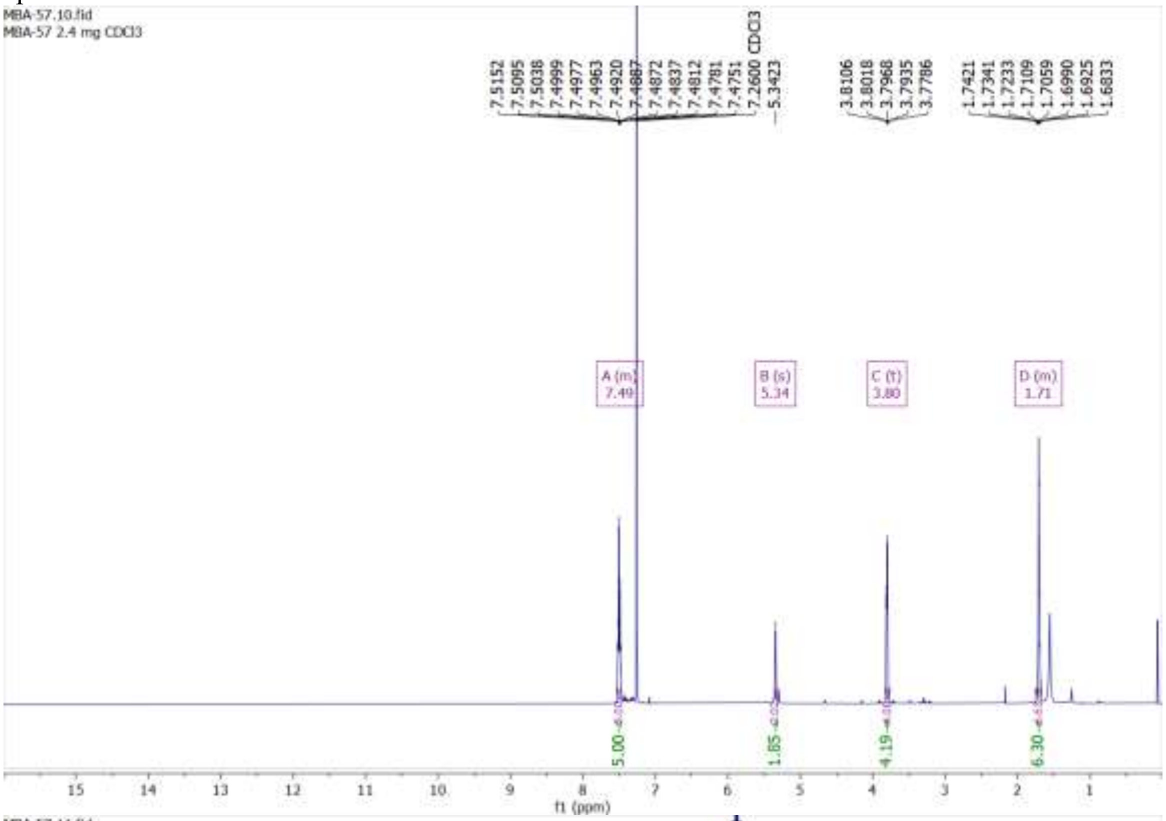
MBA-57.10.fid
MBA-57 2.4 mg CDCl3
7.5152
7.5095
7.5038
7.4999
7.4977
7.4963
7.4920
7.4887
7.4872
7.4837
7.4812
7.4781
7.4751
7.2600 CDCl3
5.3423
3.8106
3.8018
3.7968
3.7935
3.7786
1.7421
1.7341
1.7233
1.7109
1.7059
1.6990
1.6925
1.6833
A (m)
7.49
B (s)
5.34
C (t)
3.80
D (m)
1.71
5.00
1.85
4.19
6.30
f1 (ppm)

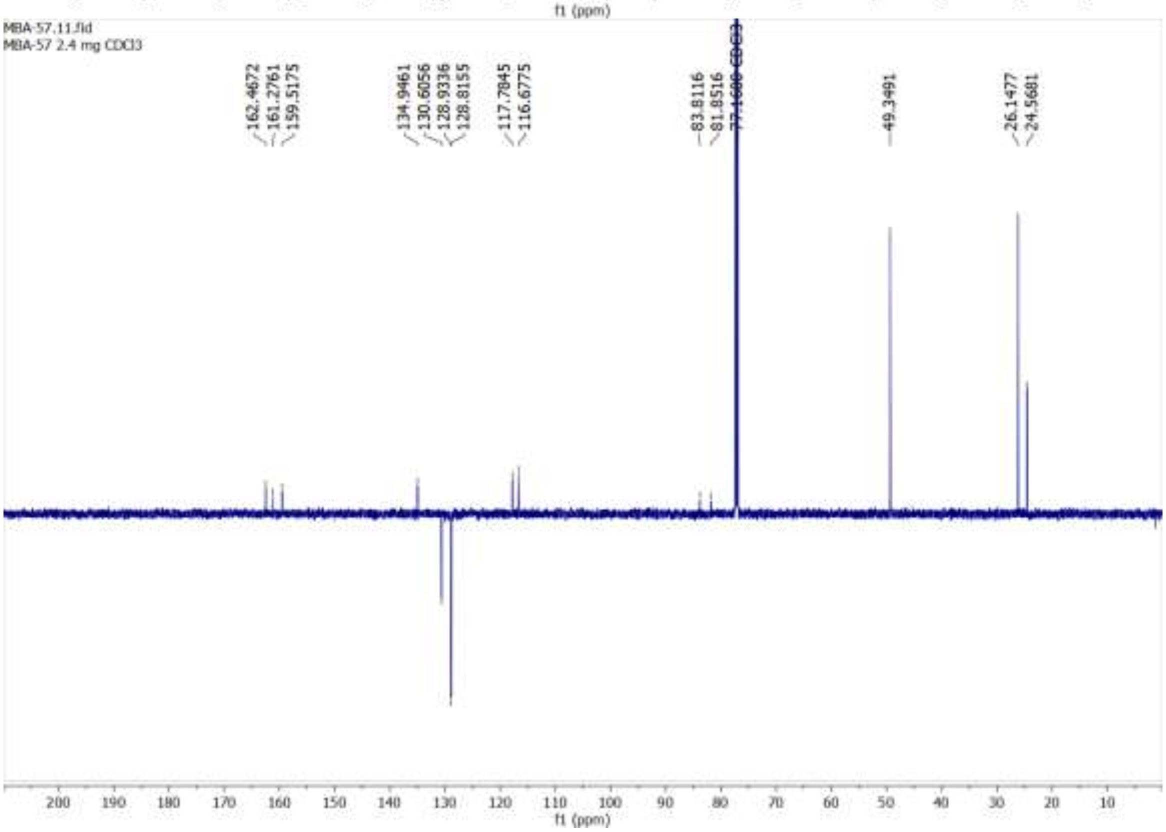
MBA-57.11.fid
MBA-57 2.4 mg CDCl3
162.4672
161.2761
159.5175
134.9461
130.6056
128.9336
128.8155
117.7845
116.6775
83.8116
81.8516
77.1600 CDCl3
49.3491
26.1477
24.5681
f1 (ppm)

Compound 6:

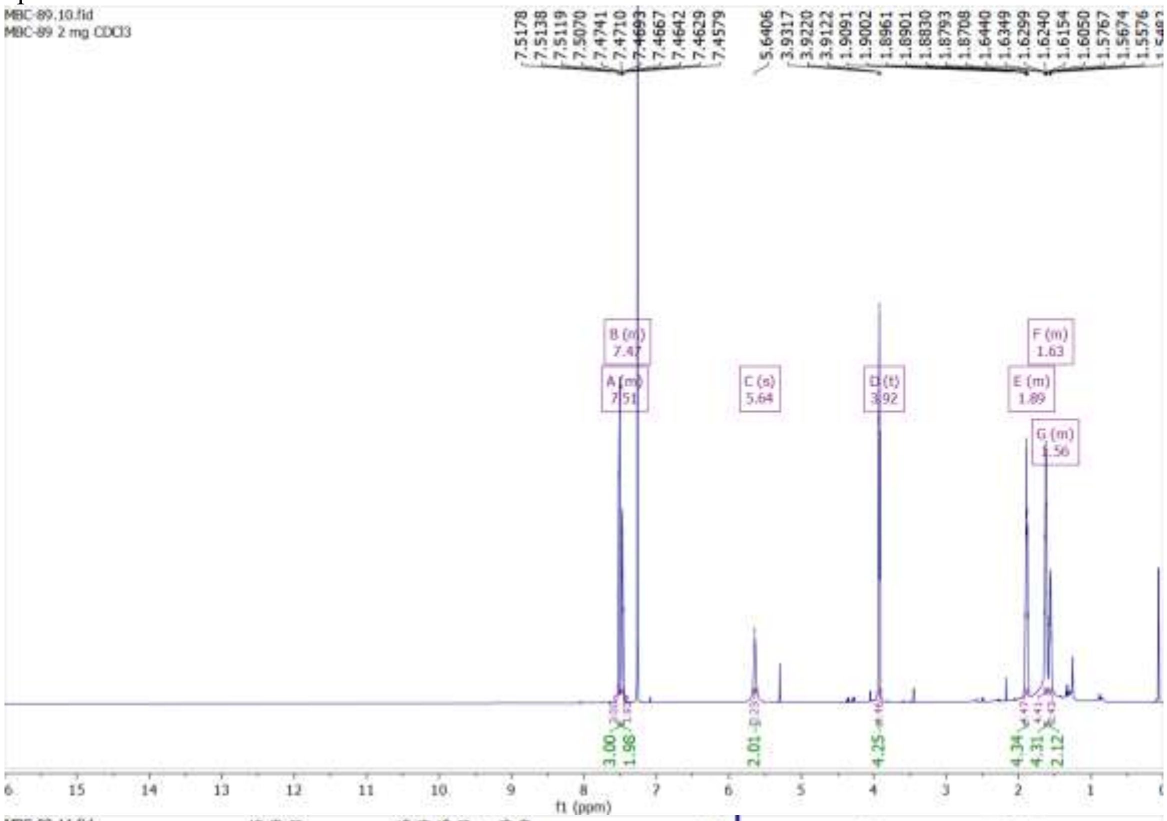
MBC-89.10.fid
MBC-89 2 mg CDCl3
7.5178
7.5138
7.5119
7.5070
7.4741
7.4710
7.4693
7.4667
7.4642
7.4629
7.4579
5.6406
3.9317
3.9220
3.9122
1.9091
1.9002
1.8961
1.8901
1.8830
1.8793
1.8708
1.6440
1.6349
1.6299
1.6240
1.6154
1.6050
1.5767
1.5674
1.5576
B (m)
7.47
A (m)
7.51
C (s)
5.64
D (t)
3.92
F (m)
1.63
E (m)
1.89
G (m)
1.56
3.00
1.98
2.01
4.25
4.34
4.31
2.12
f1 (ppm)

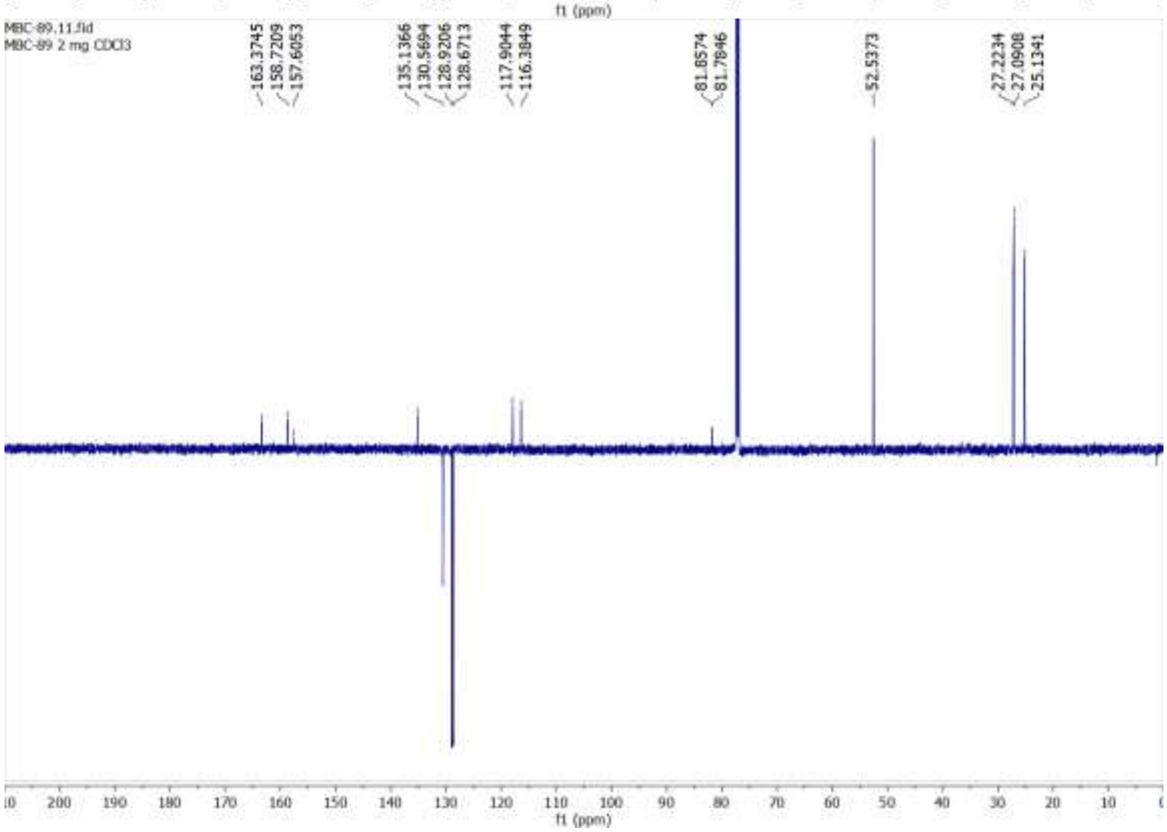
MBC-89.11.fid
MBC-89 2 mg CDCl3
163.3745
158.7209
157.6053
135.1366
130.5694
128.9206
128.6713
117.9044
116.3849
81.8574
81.7846
52.5373
27.2234
27.0908
25.1341
f1 (ppm)

Compound 7:

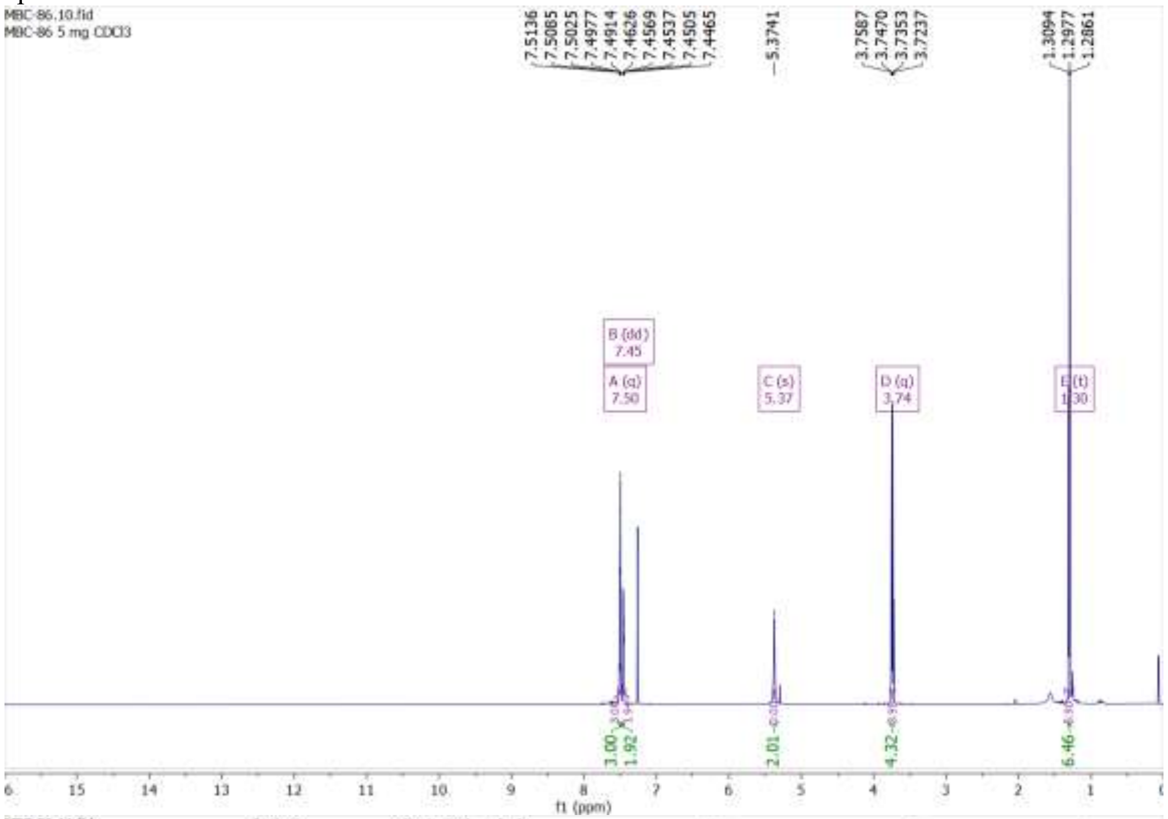
MBC-86.10.fid
MBC-86 5 mg CDCl3
7.5136
7.5085
7.5025
7.4977
7.4914
7.4626
7.4569
7.4537
7.4505
7.4465
5.3741
3.7587
3.7470
3.7353
3.7237
1.3094
1.2977
1.2861
B (dd)
7.45
A (q)
7.50
C (s)
5.37
D (q)
3.74
E (t)
1.30
3.00
1.92
2.01
4.32
6.46
f1 (ppm)

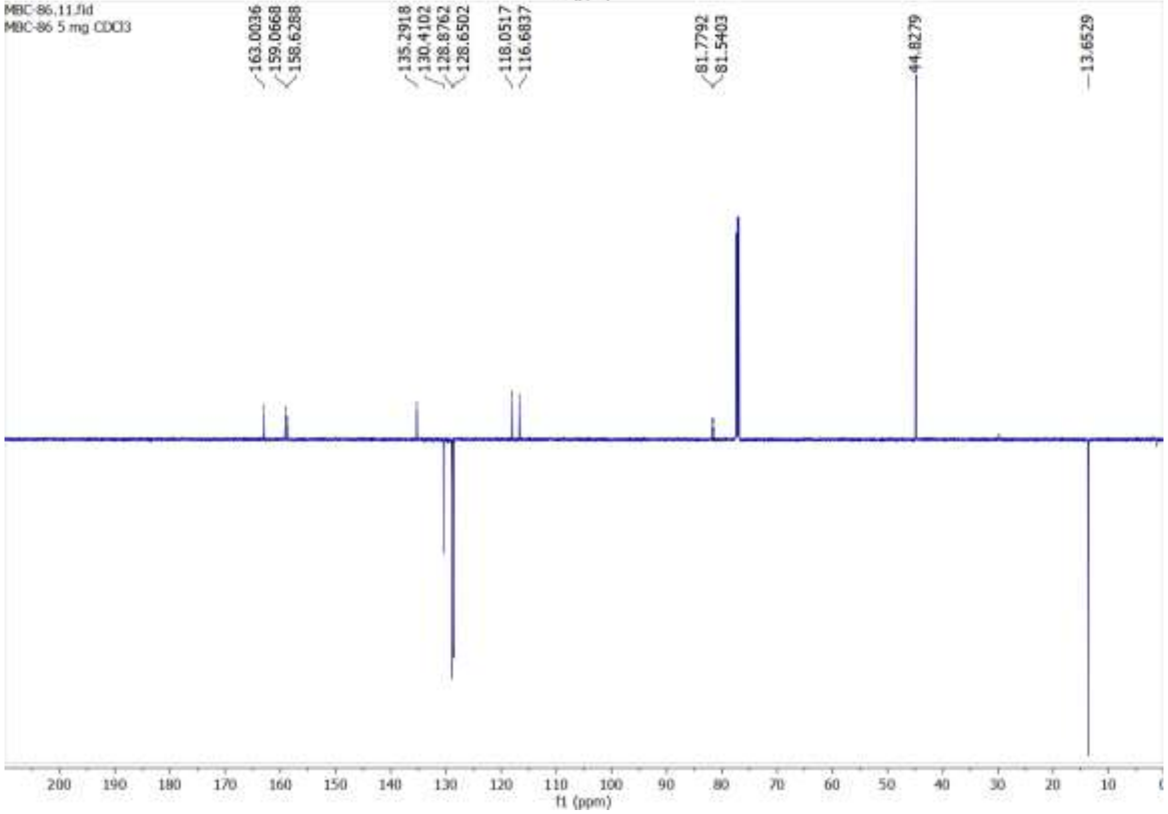
MBC-86.11.fid
MBC-86 5 mg CDCl3
163.0036
159.0668
158.6288
135.2918
130.4102
128.8762
128.6502
118.0517
116.6837
81.7792
81.5403
44.8279
13.6529
f1 (ppm)

Compound 8:

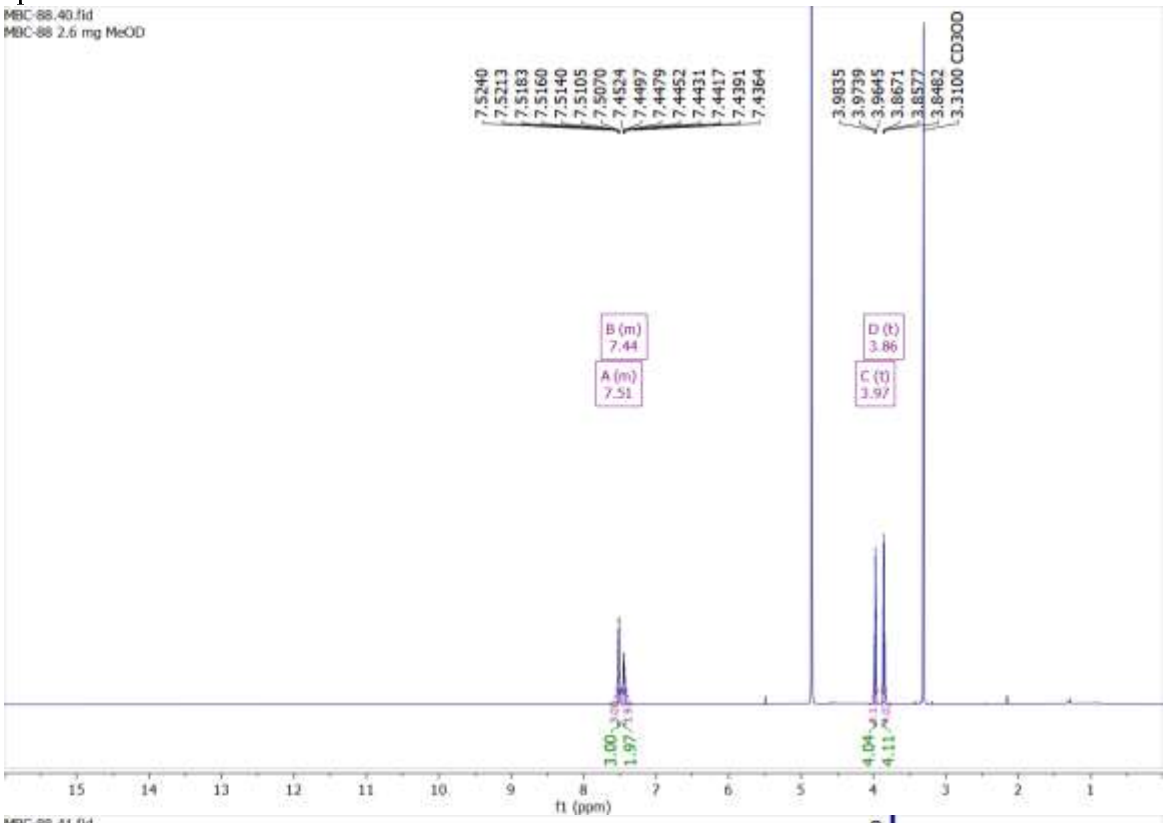
MBC-88.40.fid
MBC-88 2.6 mg MeOD
7.5240
7.5213
7.5183
7.5160
7.5140
7.5105
7.5070
7.4524
7.4497
7.4479
7.4452
7.4431
7.4417
7.4391
7.4364
3.9835
3.9739
3.9645
3.8671
3.8577
3.8482
3.3100 CD3OD
B (m)
7.44
A (m)
7.51
D (t)
3.86
C (t)
3.97
3.00
1.97
4.04
4.11
f1 (ppm)

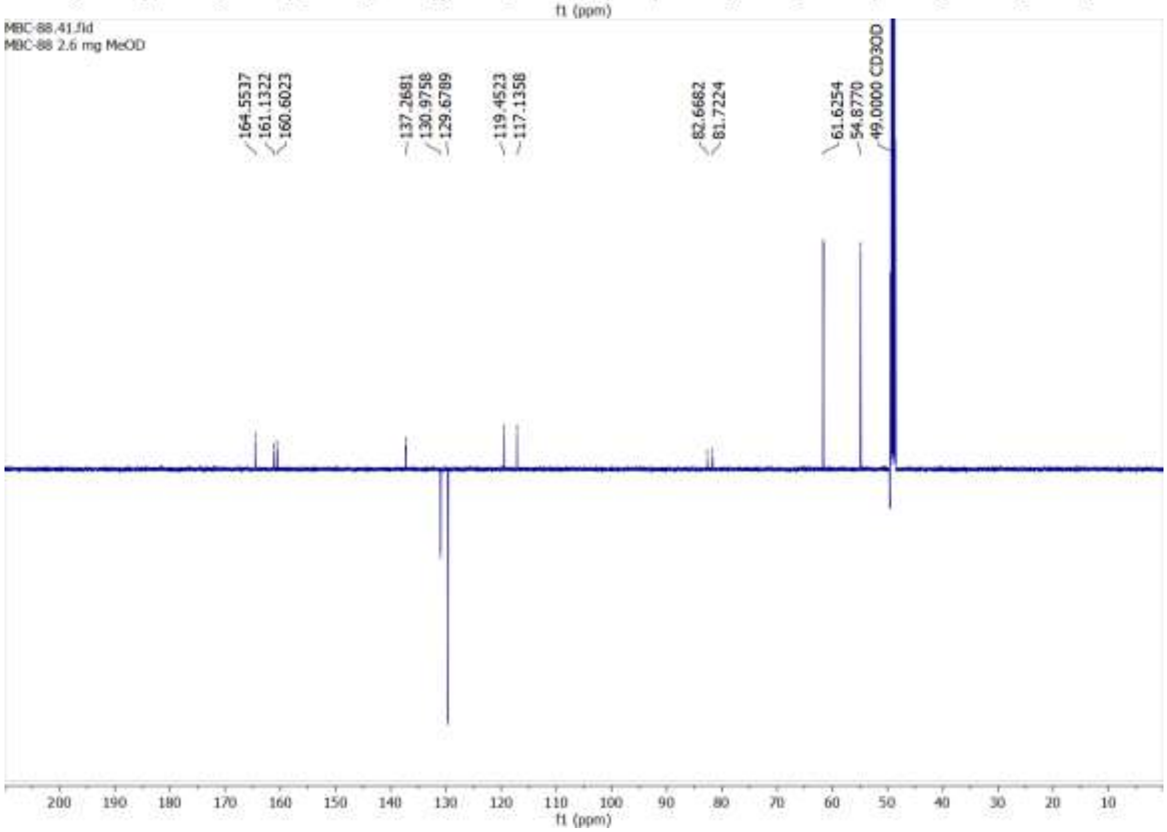
MBC-88.41.fid
MBC-88 2.6 mg MeOD
164.5537
161.1322
160.6023
137.2681
130.9758
129.6789
119.4523
117.1358
82.6682
81.7224
61.6254
54.8770
49.0000 CD3OD
f1 (ppm)

Compound 11:

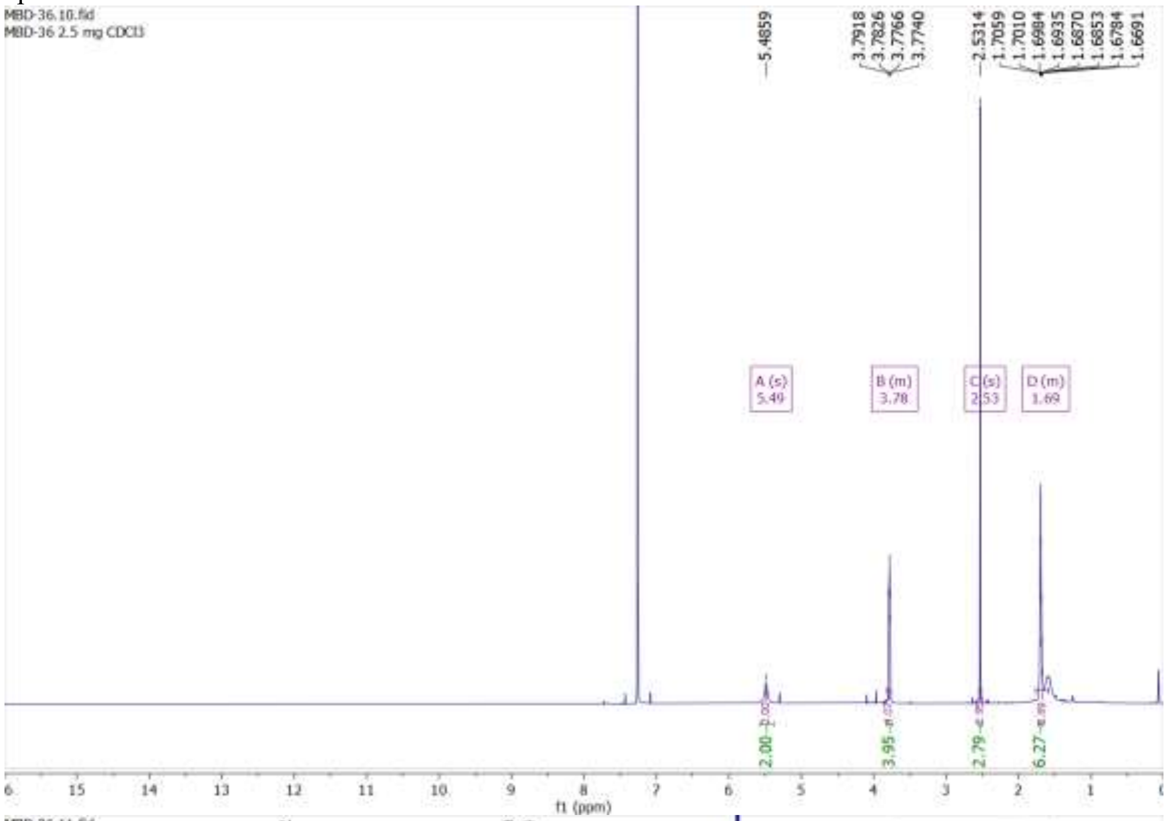
MBD-36.10.fid
MBD-36 2.5 mg CDCl3
5.4859
3.7918
3.7826
3.7766
3.7740
2.5314
1.7059
1.7010
1.6984
1.6935
1.6870
1.6853
1.6784
1.6691
A (s)
5.49
B (m)
3.78
C (s)
2.53
D (m)
1.69
2.00
3.95
2.79
6.27
f1 (ppm)

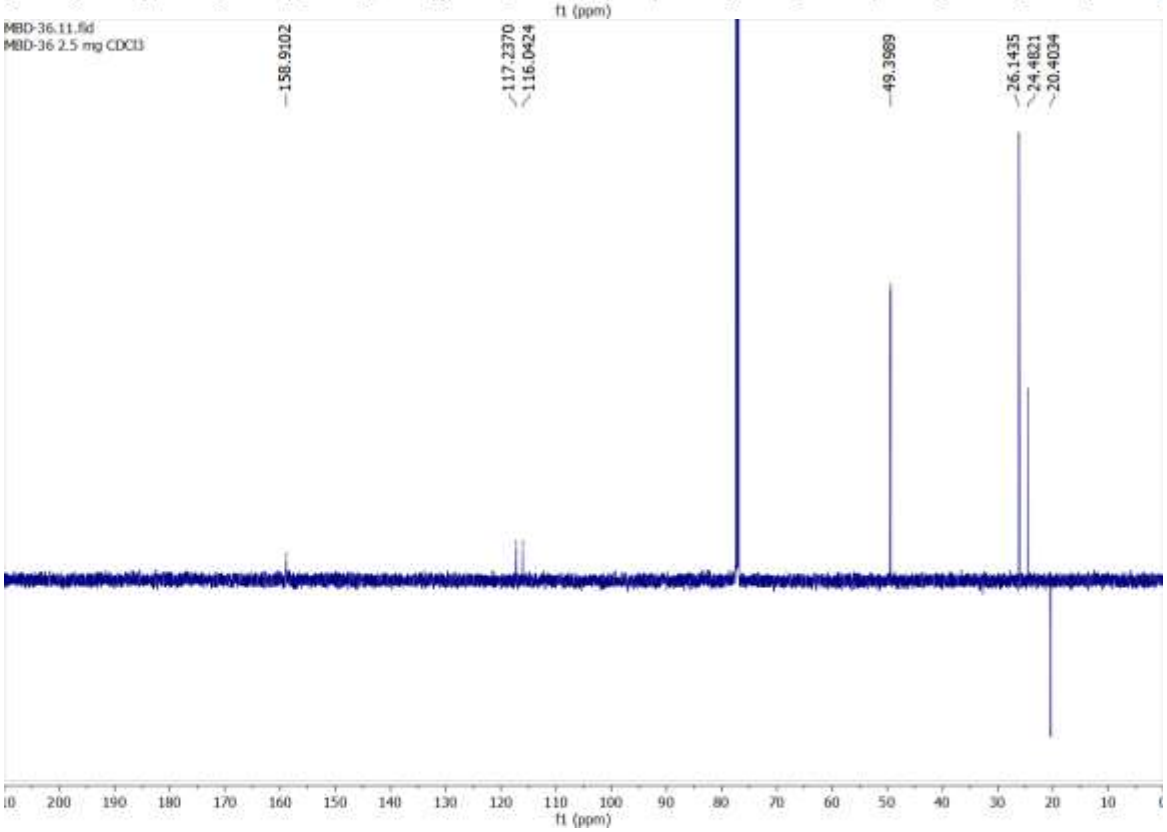
MBD-36.11.fid
MBD-36 2.5 mg CDCl3
158.9102
117.2370
116.0424
49.3989
26.1435
24.4821
20.4034
f1 (ppm)

Compound 12:

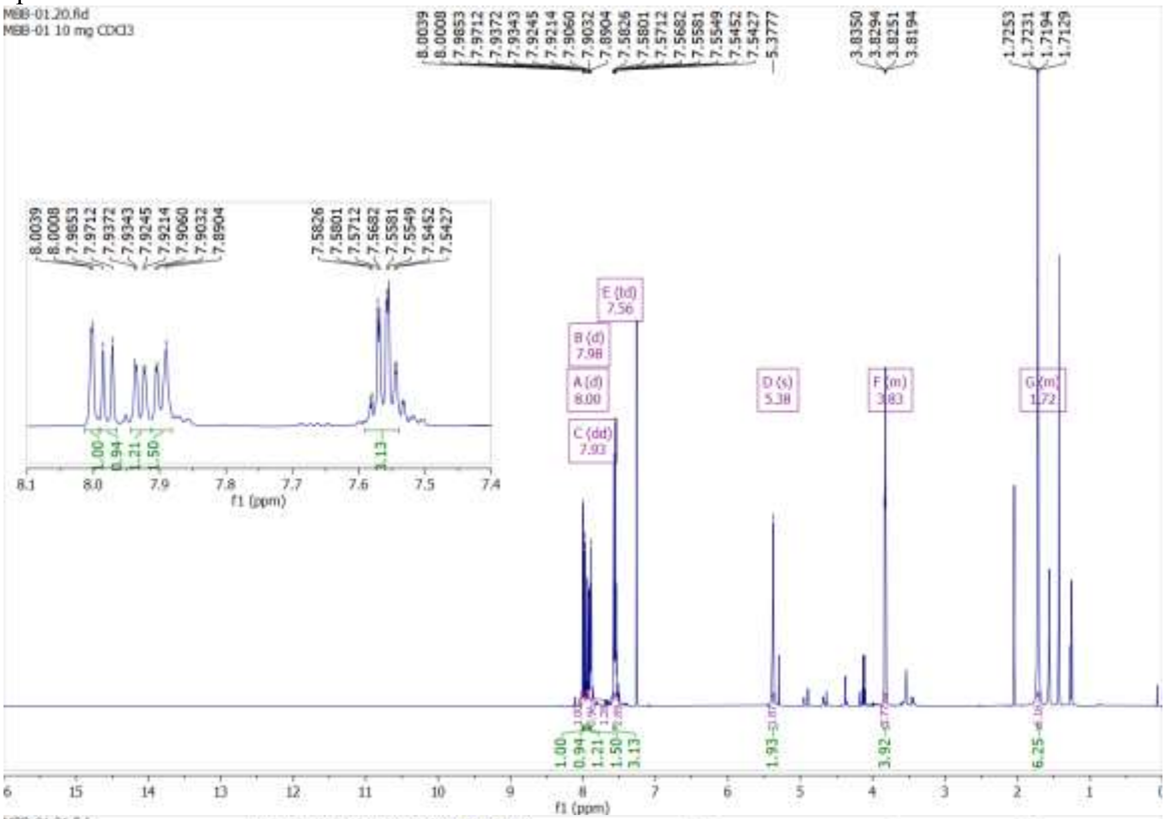
MBB-01.20.fid
MBB-01 10 mg CDCl3
8.0039
8.0008
7.9853
7.9712
7.9372
7.9343
7.9245
7.9214
7.9060
7.9032
7.8904
7.5826
7.5801
7.5712
7.5682
7.5581
7.5549
7.5452
7.5427
5.3777
3.8350
3.8294
3.8251
3.8194
1.7253
1.7231
1.7194
1.7129
E (td)
7.56
B (d)
7.98
A (d)
8.00
C (dd)
7.93
D (s)
5.38
F (m)
3.83
G (m)
1.72
1.00
0.94
1.21
1.50
3.13
1.93
3.92
6.25
f1 (ppm)

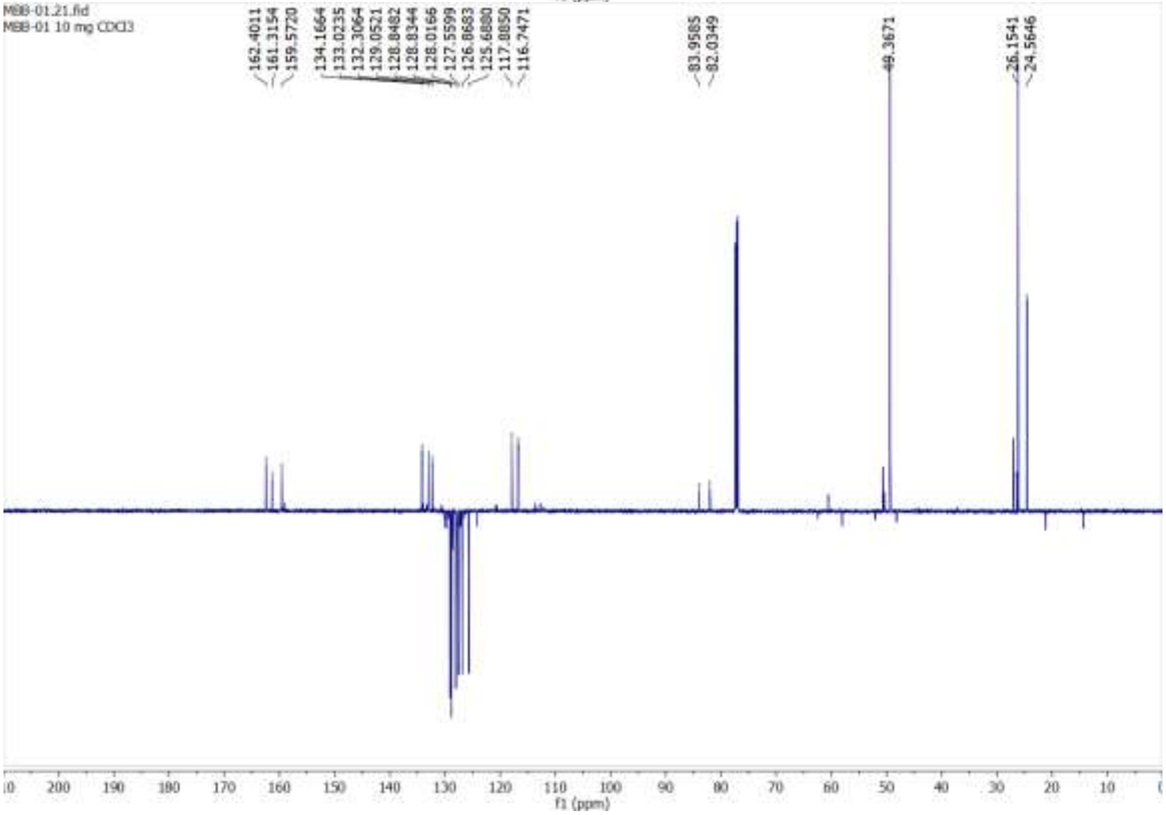
MBB-01.21.fid
MBB-01 10 mg CDCl3
162.4011
161.3154
159.5720
134.1664
133.0235
132.3064
129.0521
128.8482
128.8344
128.0166
127.5599
126.8683
125.6880
117.8850
116.7471
83.9585
82.0349
49.3671
26.1541
24.5646
f1 (ppm)

Compound 13:

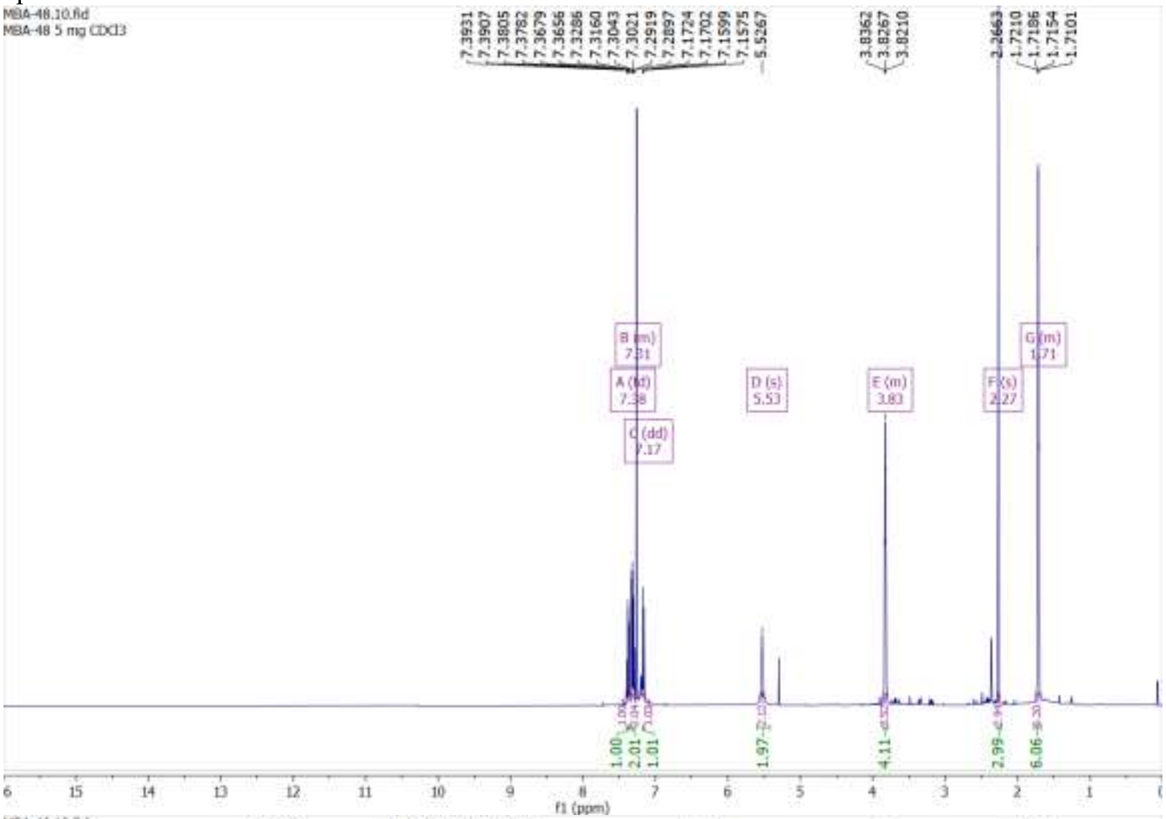
MBA-48.10.fid
MBA-48 5 mg CDCl3
7.3931
7.3907
7.3805
7.3782
7.3679
7.3656
7.3286
7.3160
7.3043
7.3021
7.2919
7.2897
7.1724
7.1702
7.1599
7.1575
5.5267
3.8362
3.8267
3.8210
2.2663
1.7210
1.7186
1.7154
1.7101
B (m) 7.31
A (td) 7.38
C (dd) 7.17
D (s) 5.53
E (m) 3.83
F (s) 2.27
G (m) 1.71
1.00
2.01
1.01
1.97
4.11
2.99
6.06
f1 (ppm)

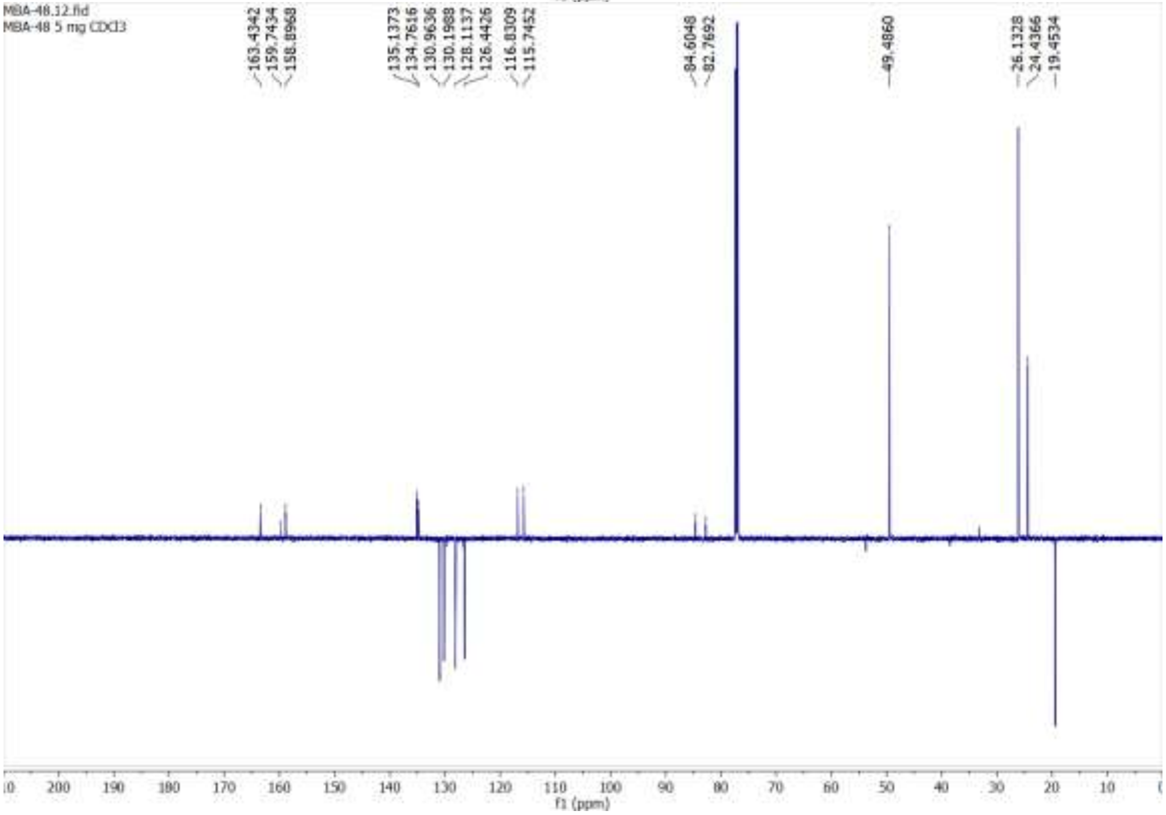
MBA-48.12.fid
MBA-48 5 mg CDCl3
163.4342
159.7434
158.8968
135.1373
134.7616
130.9636
130.1988
128.1137
126.4426
116.8309
115.7452
84.6048
82.7692
49.4860
26.1328
24.4366
19.4534
f1 (ppm)

Compound 14:

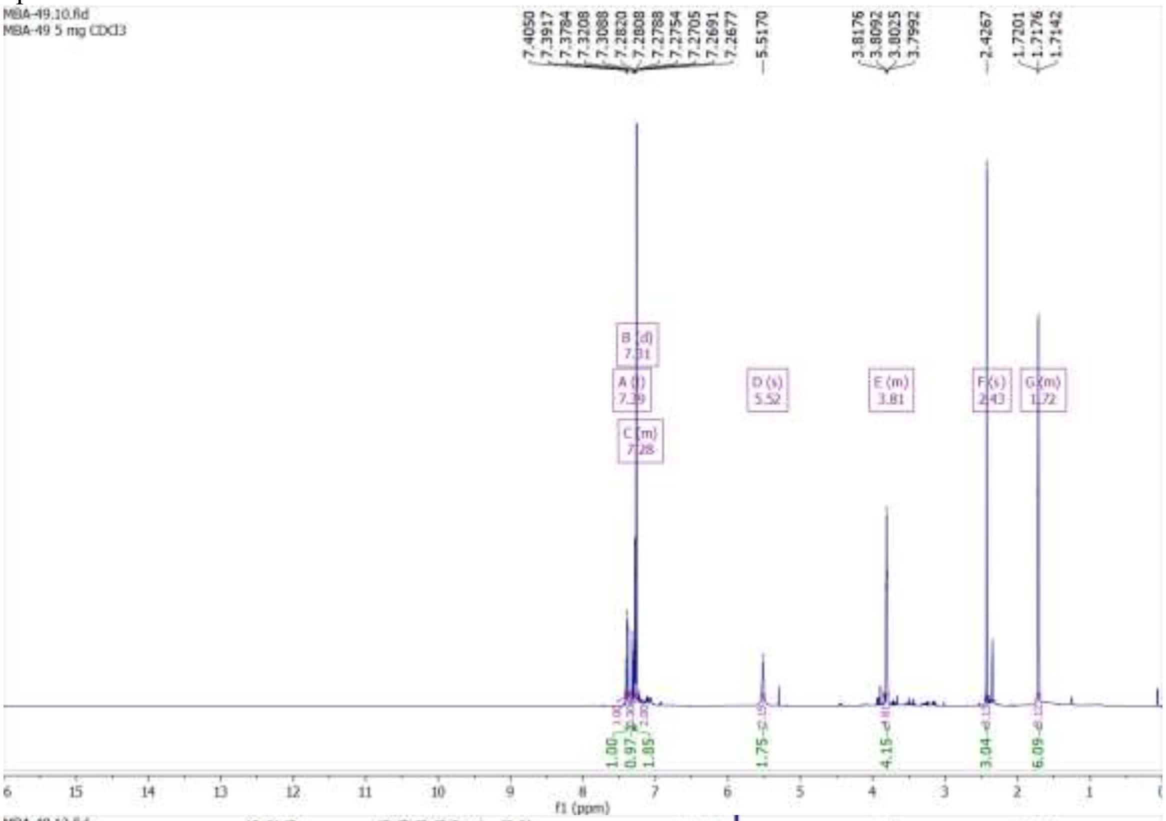
MBA-49.10.fid
MBA-49 5 mg CDCl3
7.4050
7.3917
7.3784
7.3208
7.3088
7.2820
7.2808
7.2788
7.2754
7.2705
7.2691
7.2677
5.5170
3.8176
3.8092
3.8025
3.7992
2.4267
1.7201
1.7176
1.7142
B (d)
7.31
A (t)
7.39
C (m)
7.28
D (s)
5.52
E (m)
3.81
F (s)
2.43
G (m)
1.72
1.00
0.97
1.85
1.75
4.15
3.04
6.09
f1 (ppm)

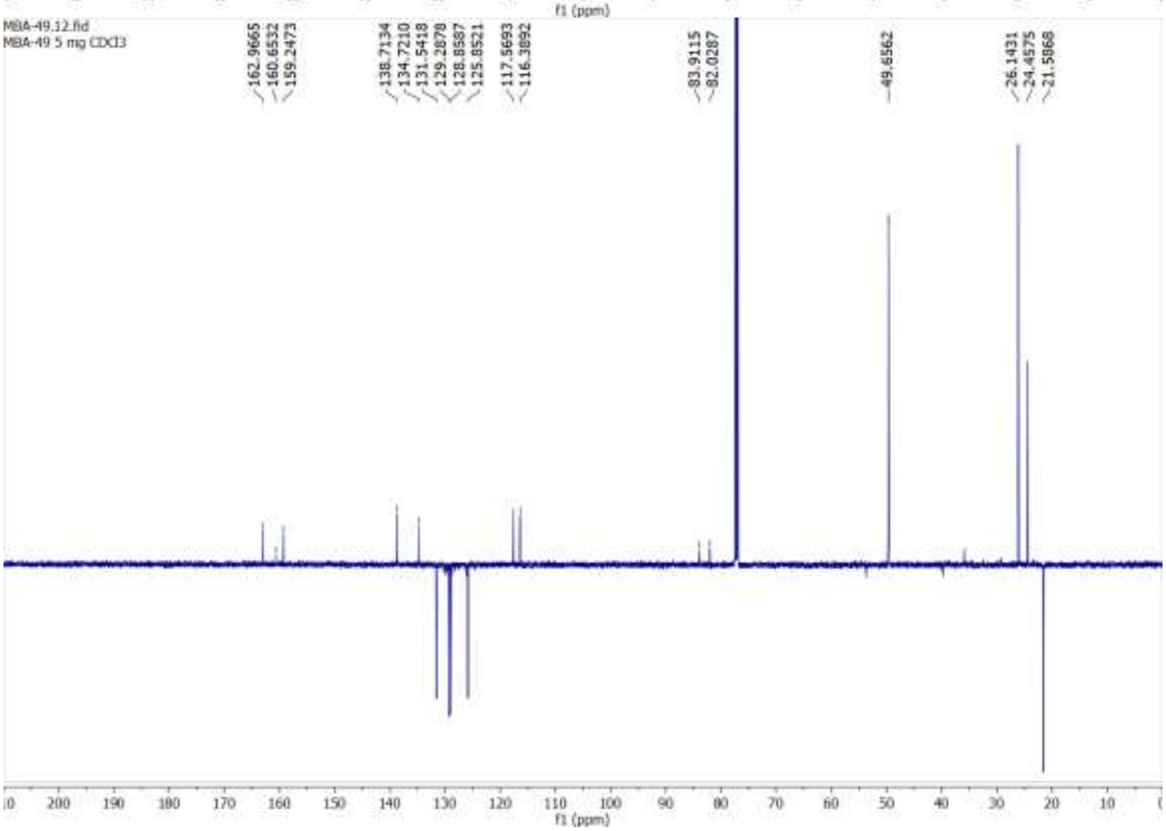
MBA-49.12.fid
MBA-49 5 mg CDCl3
162.9665
160.6532
159.2473
138.7134
134.7210
131.5418
129.2878
128.8587
125.8521
117.5693
116.3892
83.9115
82.0287
49.6562
26.1431
24.4575
21.5868
f1 (ppm)

Compound 15:

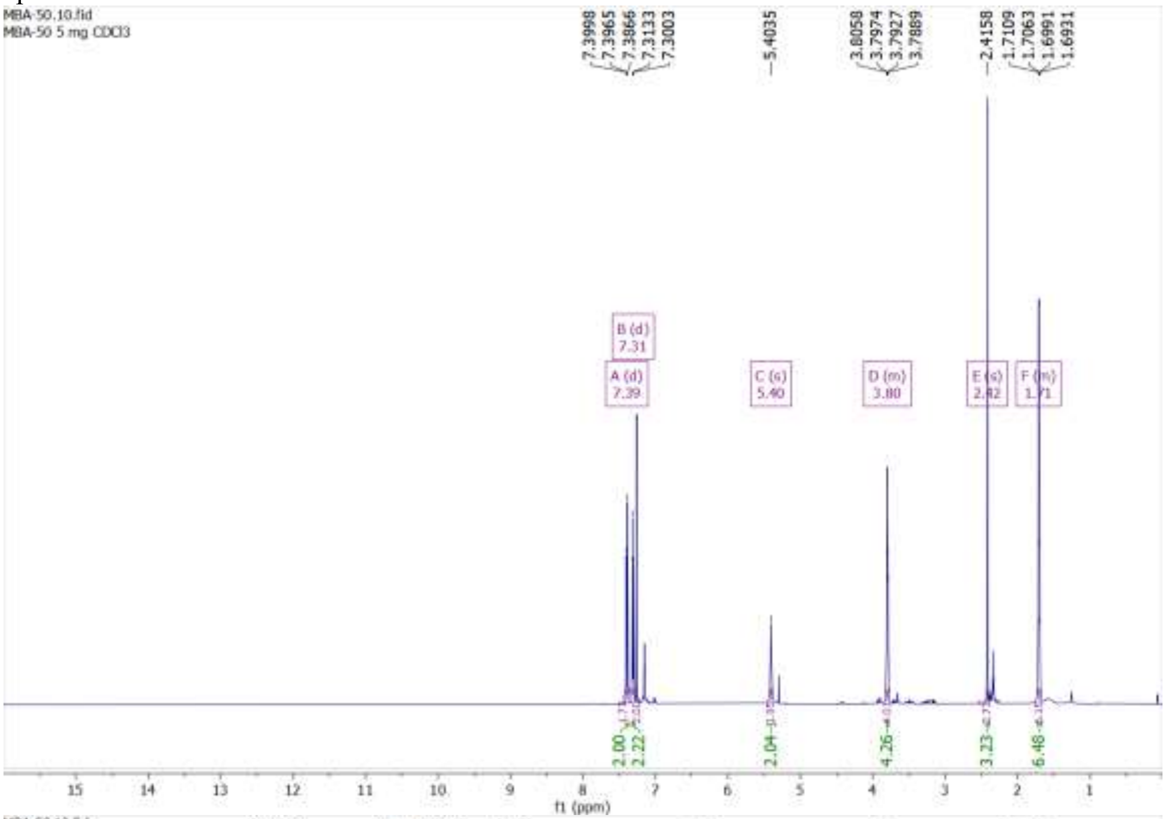

MBA-50.10.fid
MBA-50 5 mg CDCl3
7.3998
7.3965
7.3866
7.3133
7.3003
5.4035
3.8058
3.7974
3.7927
3.7889
2.4158
1.7109
1.7063
1.6991
1.6931
B (d)
7.31
A (d)
7.39
C (s)
5.40
D (m)
3.80
E (s)
2.42
F (m)
1.71
2.00
2.22
2.04
4.26
3.23
6.48
f1 (ppm)

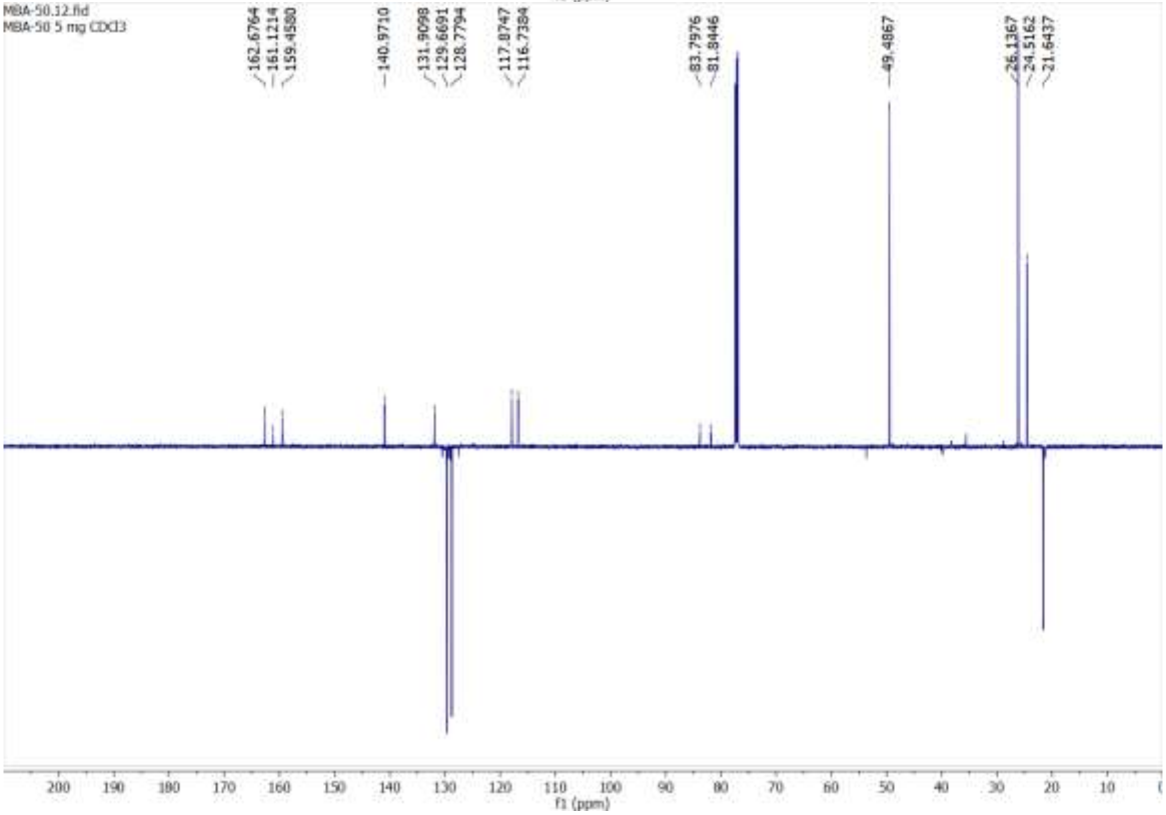

MBA-50.12.fid
MBA-50 5 mg CDCl3
162.6764
161.1214
159.4580
140.9710
131.9098
129.6691
128.7794
117.8747
116.7384
83.7976
81.8446
49.4867
26.1367
24.5162
21.6437
f1 (ppm)

Compound 16:

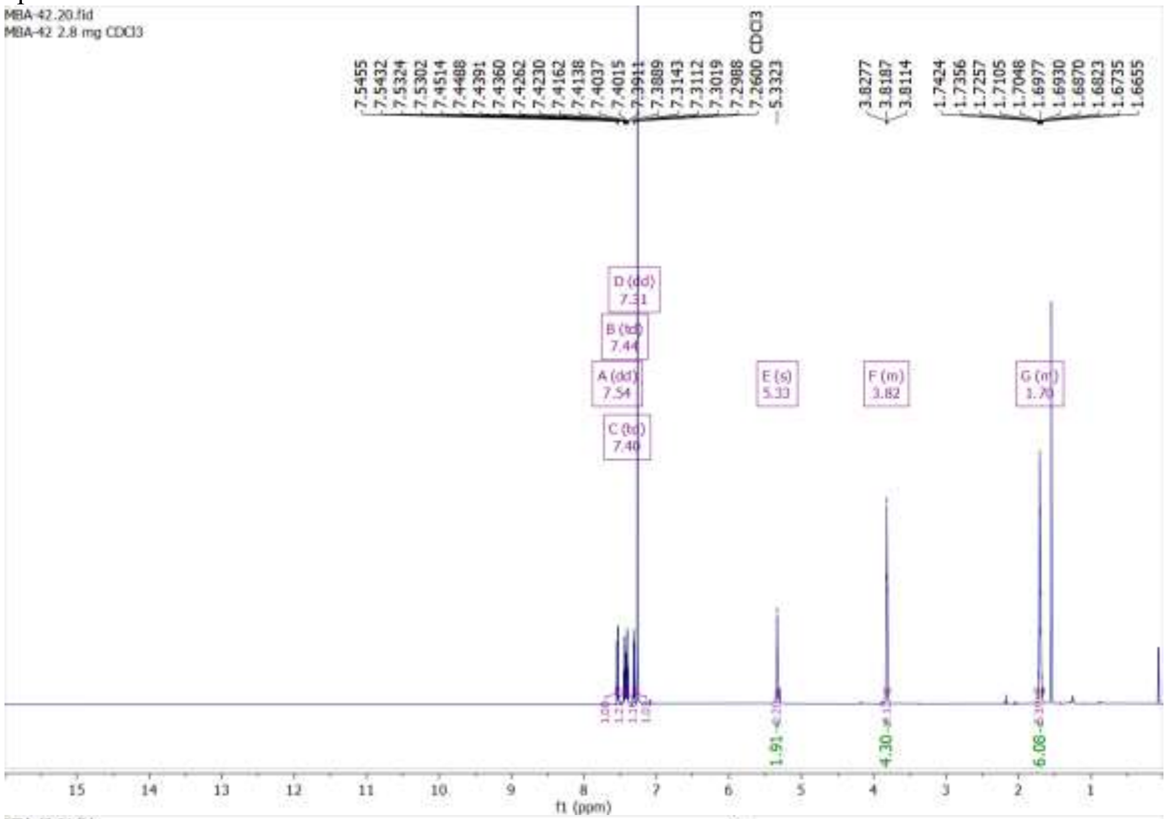
MBA-42.20.fid
MBA-42 2.8 mg CDCl3
7.5455
7.5432
7.5324
7.5302
7.4514
7.4488
7.4391
7.4360
7.4262
7.4230
7.4162
7.4138
7.4037
7.4015
7.3911
7.3889
7.3143
7.3112
7.3019
7.2988
7.2600 CDCl3
5.3323
3.8277
3.8187
3.8114
1.7424
1.7356
1.7257
1.7105
1.7048
1.6977
1.6930
1.6870
1.6823
1.6735
1.6655
D (dd) 7.31
B (td) 7.44
A (dd) 7.54
C (td) 7.40
E (s) 5.33
F (m) 3.82
G (m) 1.70
1.91
4.30
6.08
f1 (ppm)

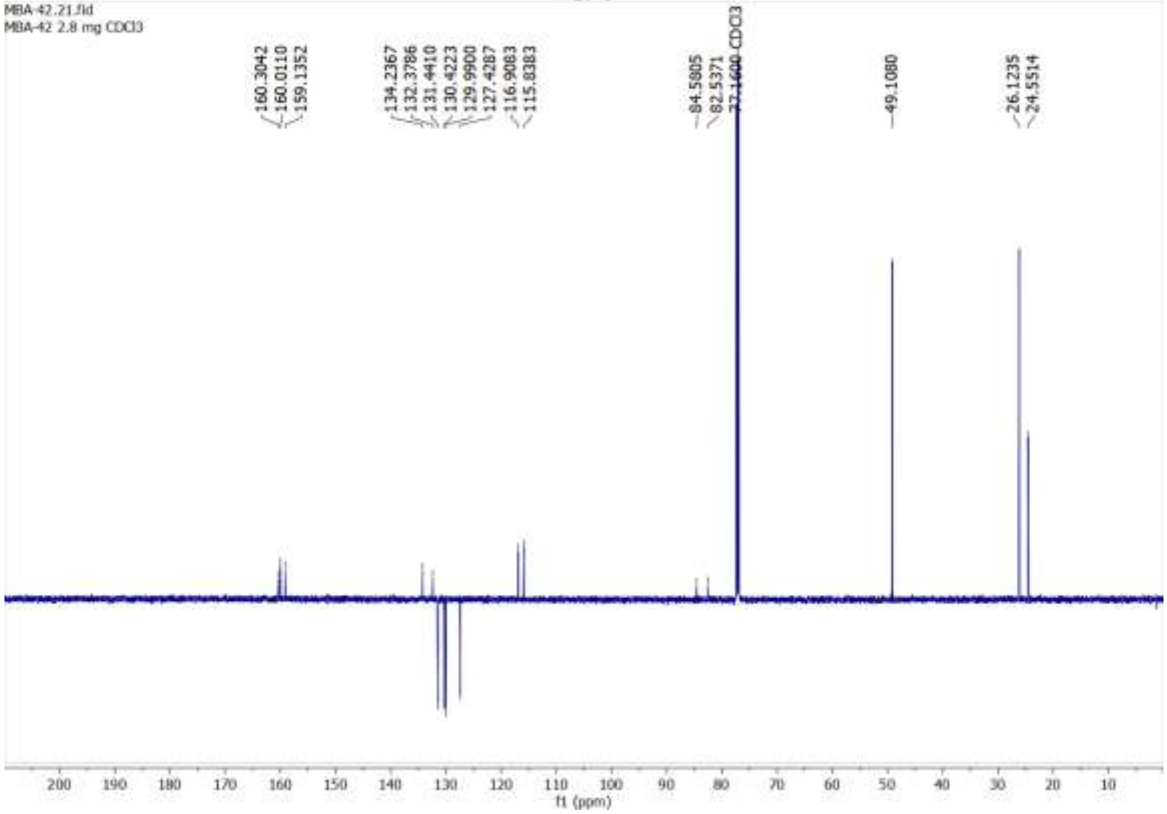
MBA-42.21.fid
MBA-42 2.8 mg CDCl3
160.3042
160.0110
159.1352
134.2367
132.3786
131.4410
130.4223
129.9900
127.4287
116.9083
115.8383
84.5805
82.5371
77.1600 CDCl3
49.1080
26.1235
24.5514
f1 (ppm)

Compound 17:

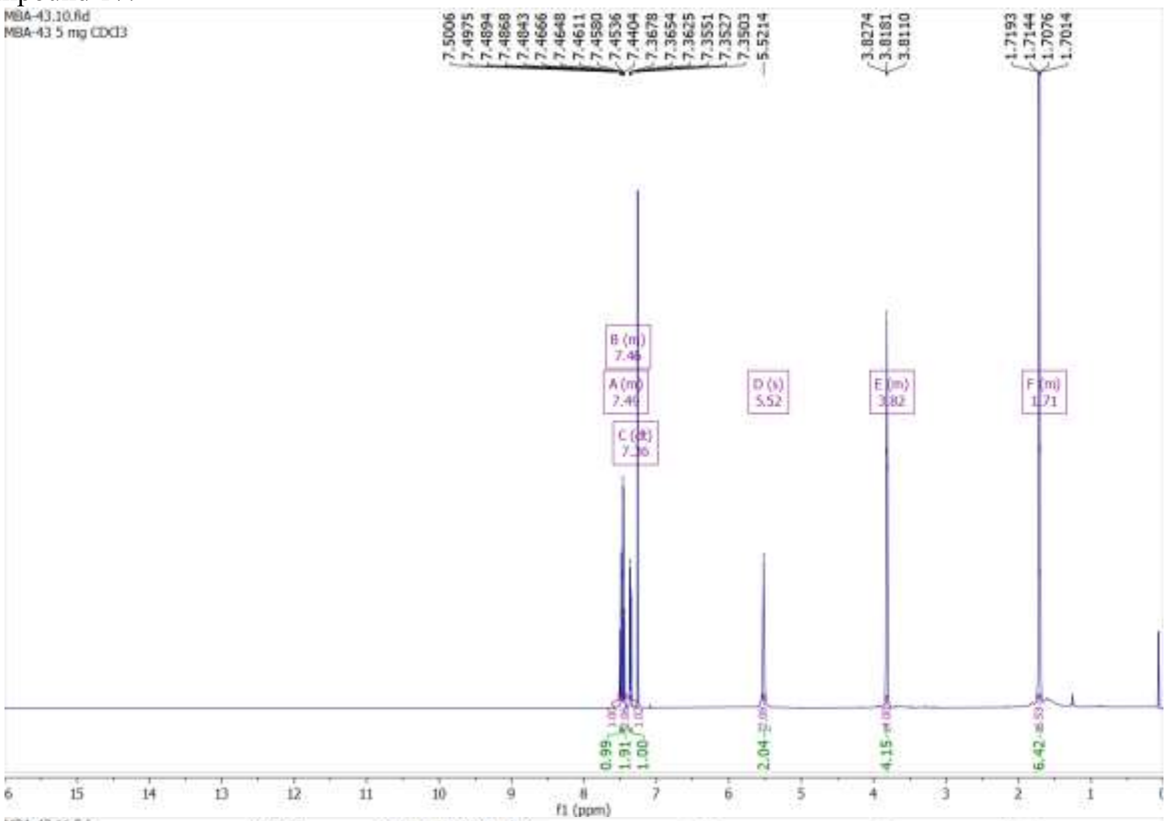

MBA-43.10.fid
MBA-43 5 mg CDCl3
7.5006
7.4975
7.4894
7.4868
7.4843
7.4666
7.4648
7.4611
7.4580
7.4536
7.4404
7.3678
7.3654
7.3625
7.3551
7.3527
7.3503
5.5214
3.8274
3.8181
3.8110
1.7193
1.7144
1.7076
1.7014
B (m)
7.46
A (m)
7.49
C (dt)
7.36
D (s)
5.52
E (m)
3.82
F (m)
1.71
0.99
1.91
1.00
2.04
4.15
6.42
f1 (ppm)

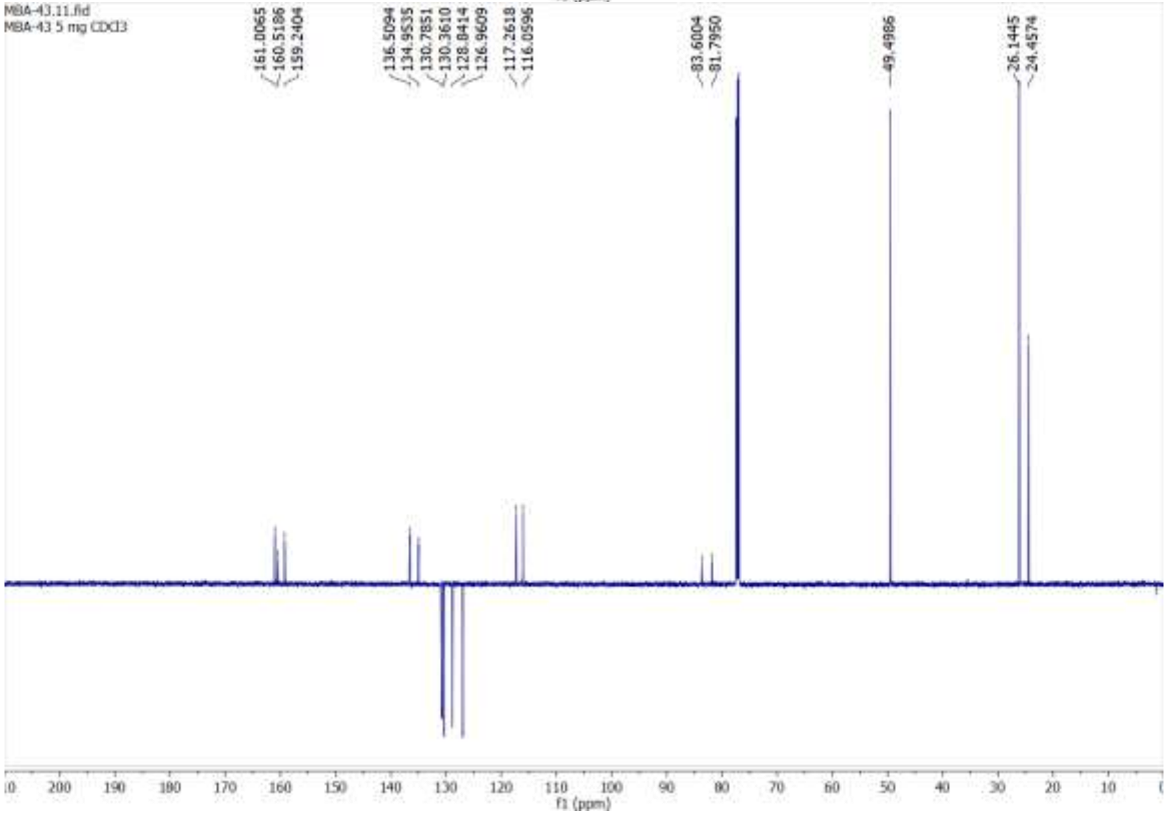

MBA-43.11.fid
MBA-43 5 mg CDCl3
161.0065
160.5186
159.2404
136.5094
134.9535
130.7851
130.3610
128.8414
126.9609
117.2618
116.0596
83.6004
81.7950
49.4986
26.1445
24.4574
f1 (ppm)

Compound 18:

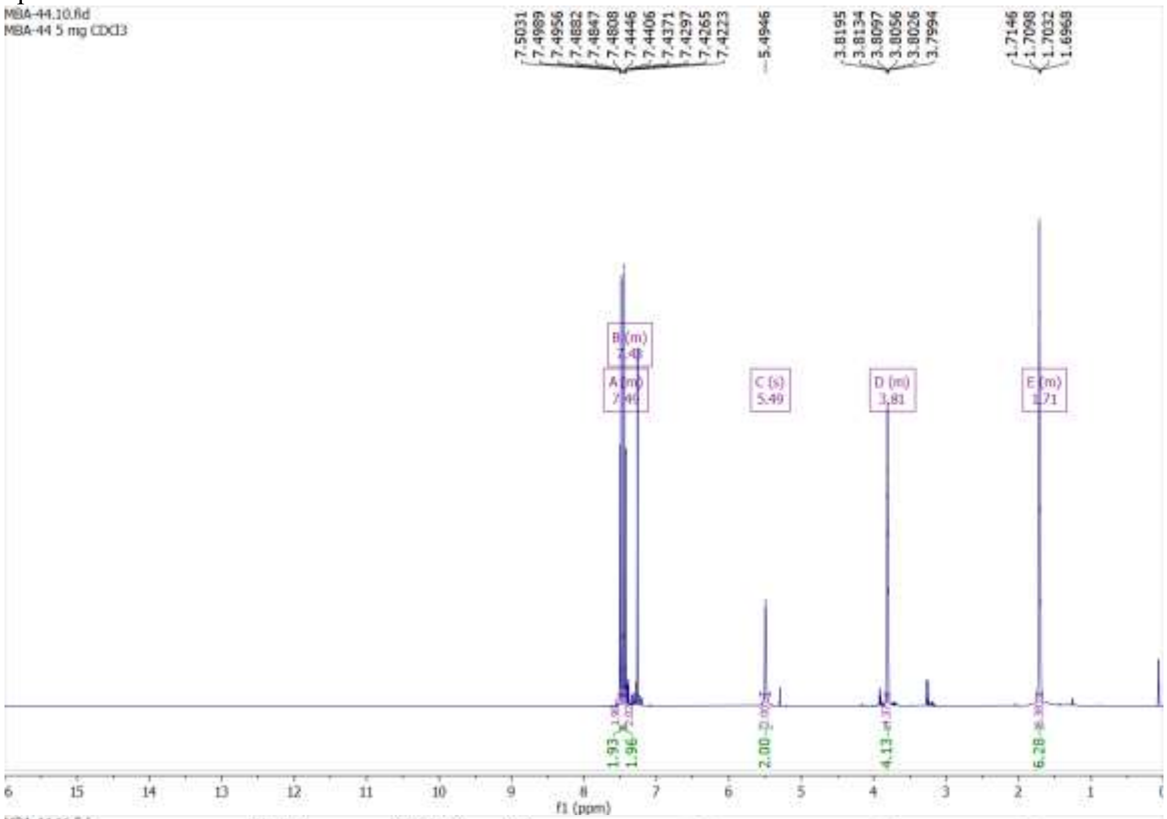
MBA-44.10.fid
MBA-44 5 mg CDCl3
7.5031
7.4989
7.4956
7.4882
7.4847
7.4808
7.4446
7.4406
7.4371
7.4297
7.4265
7.4223
5.4946
3.8195
3.8134
3.8097
3.8056
3.8026
3.7994
1.7146
1.7098
1.7032
1.6968
B (m)
7.48
A (m)
7.49
C (s)
5.49
D (m)
3.81
E (m)
1.71
1.93
1.96
2.00
4.13
6.28
f1 (ppm)

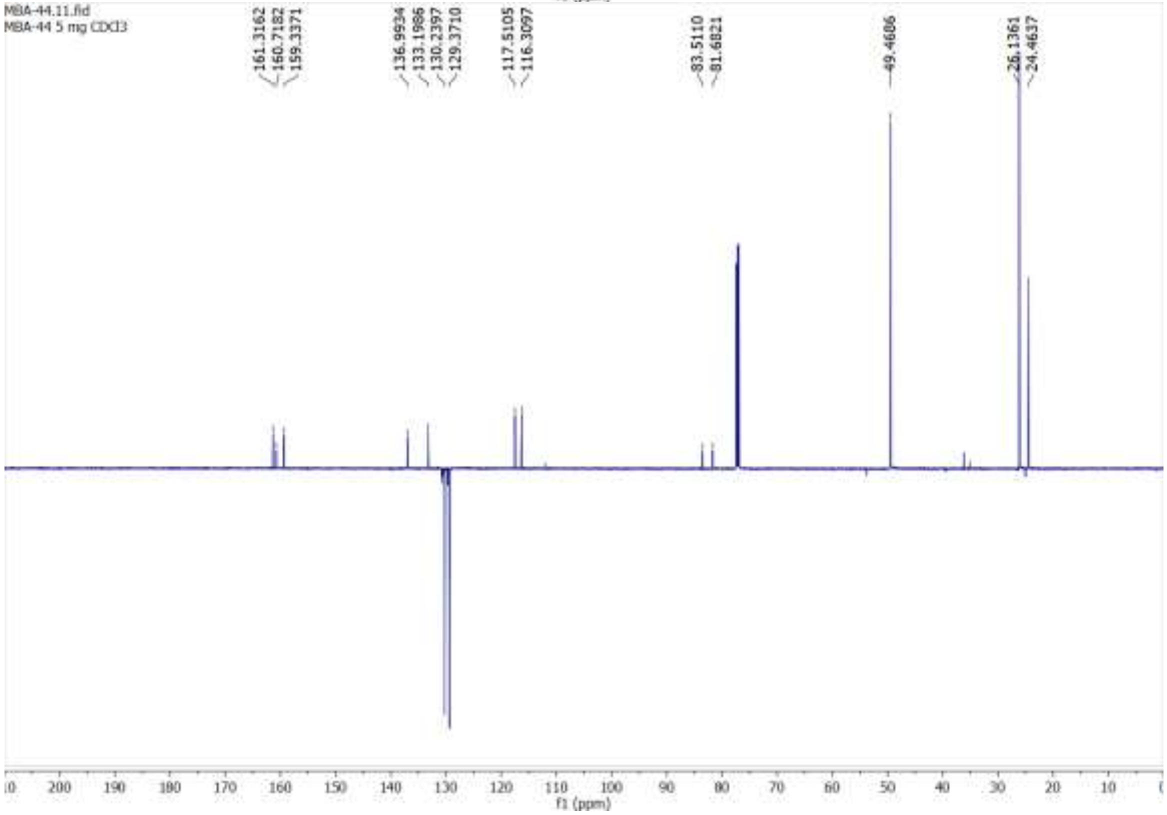
MBA-44.11.fid
MBA-44 5 mg CDCl3
161.3162
160.7182
159.3371
136.9934
133.1986
130.2397
129.3710
117.5105
116.3097
83.5110
81.6821
49.4686
26.1361
24.4637
f1 (ppm)

Compound 19:

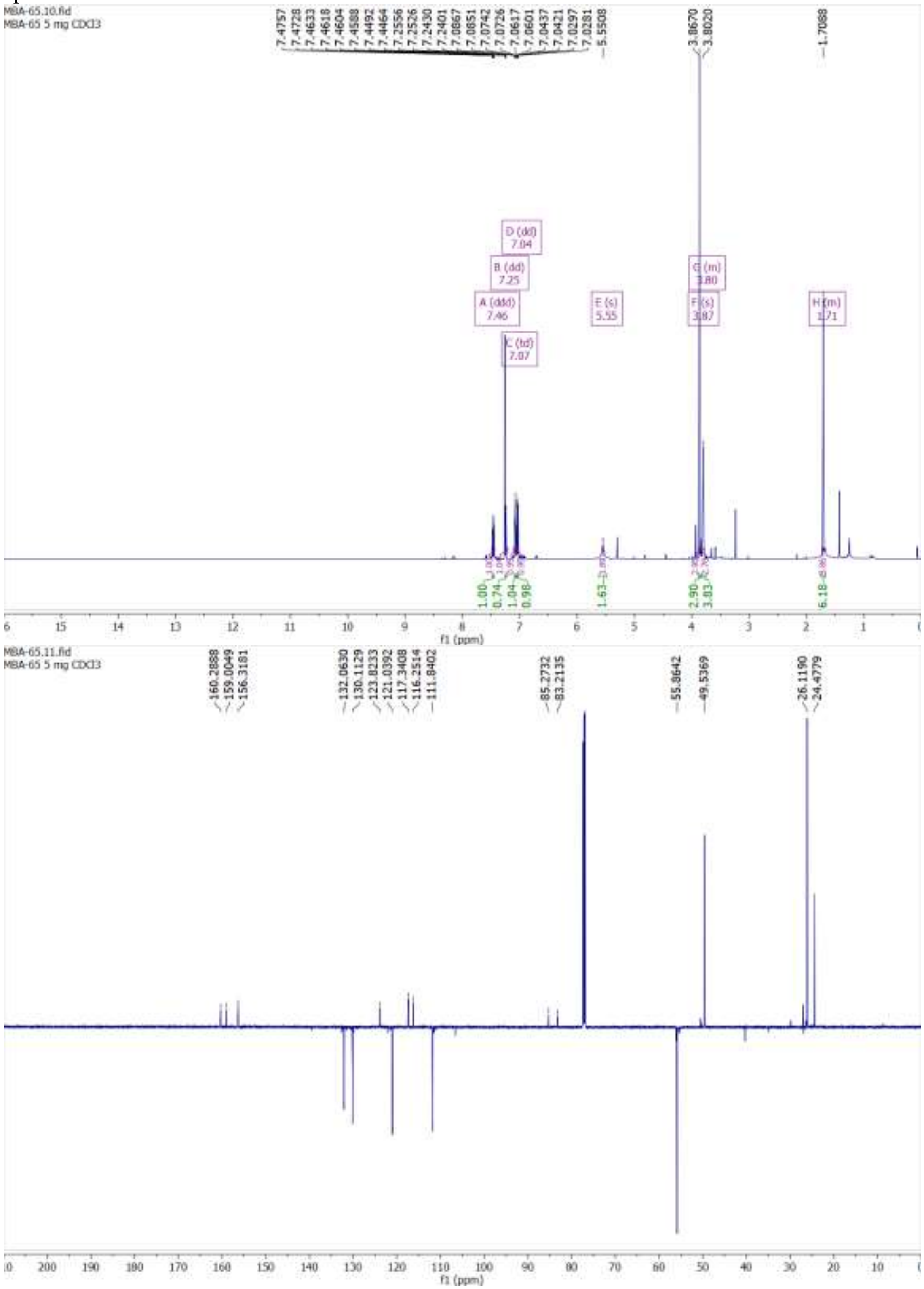
MBA-65.10.fid
MBA-65 5 mg CDCl3
7.4757 7.4728 7.4633 7.4618 7.4604 7.4588 7.4492 7.4464 7.2556 7.2526 7.2430 7.2401 7.0867 7.0851 7.0742 7.0726 7.0617 7.0601 7.0437 7.0421 7.0297 7.0281
5.5508
3.8670 3.8020
1.7088
D (dd) 7.04
B (dd) 7.25
A (ddd) 7.46
C (td) 7.07
E (s) 5.55
G (m) 3.80
F (s) 3.87
H (m) 1.71
1.00 0.74 1.04 0.98 1.63 2.90 3.03 6.18
f1 (ppm)
MBA-65.11.fid
MBA-65 5 mg CDCl3
160.2888 159.0049 156.3181
132.0630 130.1129 123.8233 121.0392 117.3408 116.2514 111.8402
85.2732 83.2135
55.8642 49.5369
26.1190 24.4779
f1 (ppm)

Compound 20:

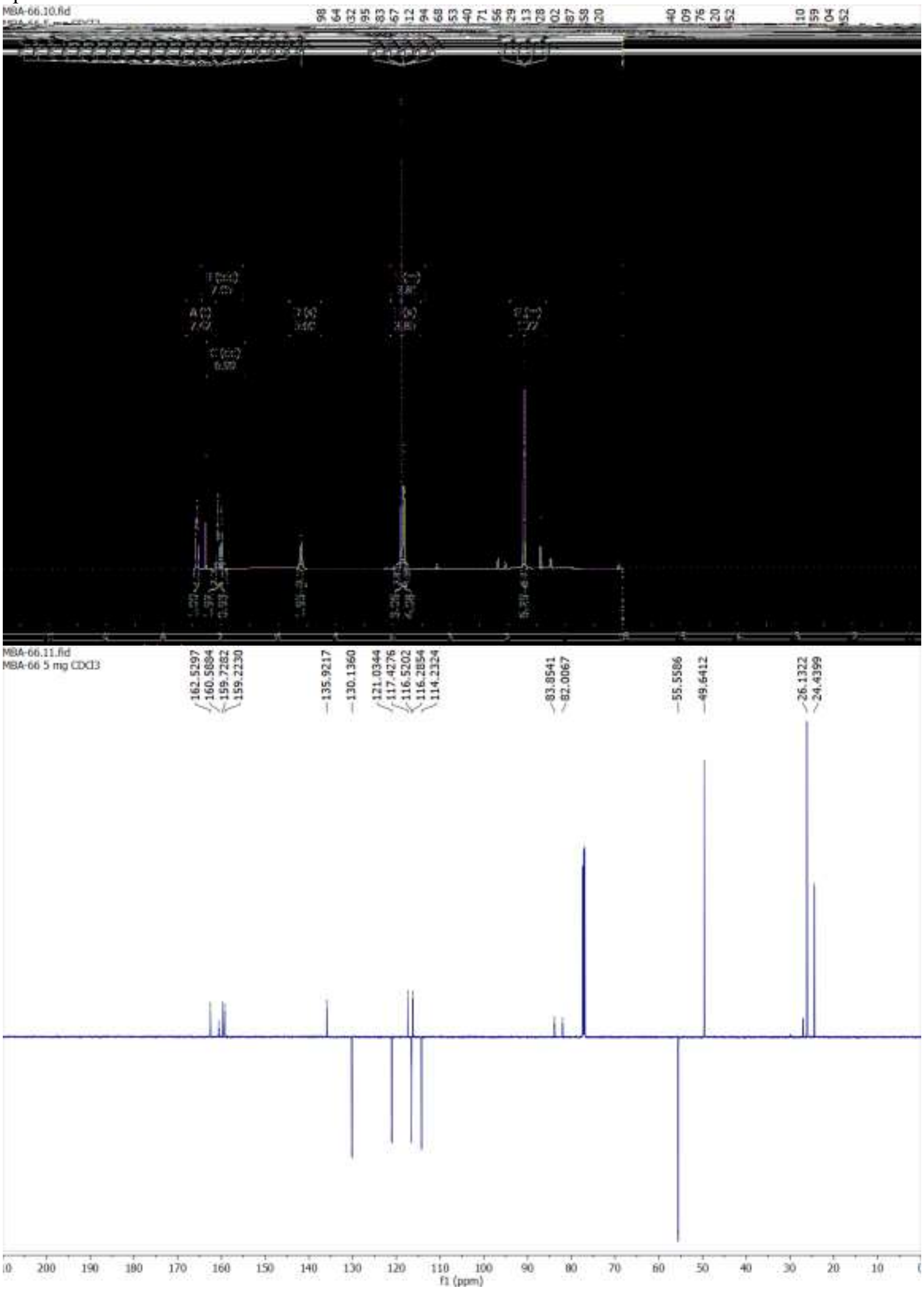

MBA-66.11.fid
MBA-66 5 mg CDCl3
162.5297
160.5884
159.7282
159.2230
135.9217
130.1360
121.0344
117.4276
116.5202
116.2854
114.2324
83.8541
82.0067
55.5586
49.6412
26.1322
24.4399
200
190
180
170
160
150
140
130
120
110
100
90
80
70
60
50
40
30
20
10
f1 (ppm)

Compound 21:

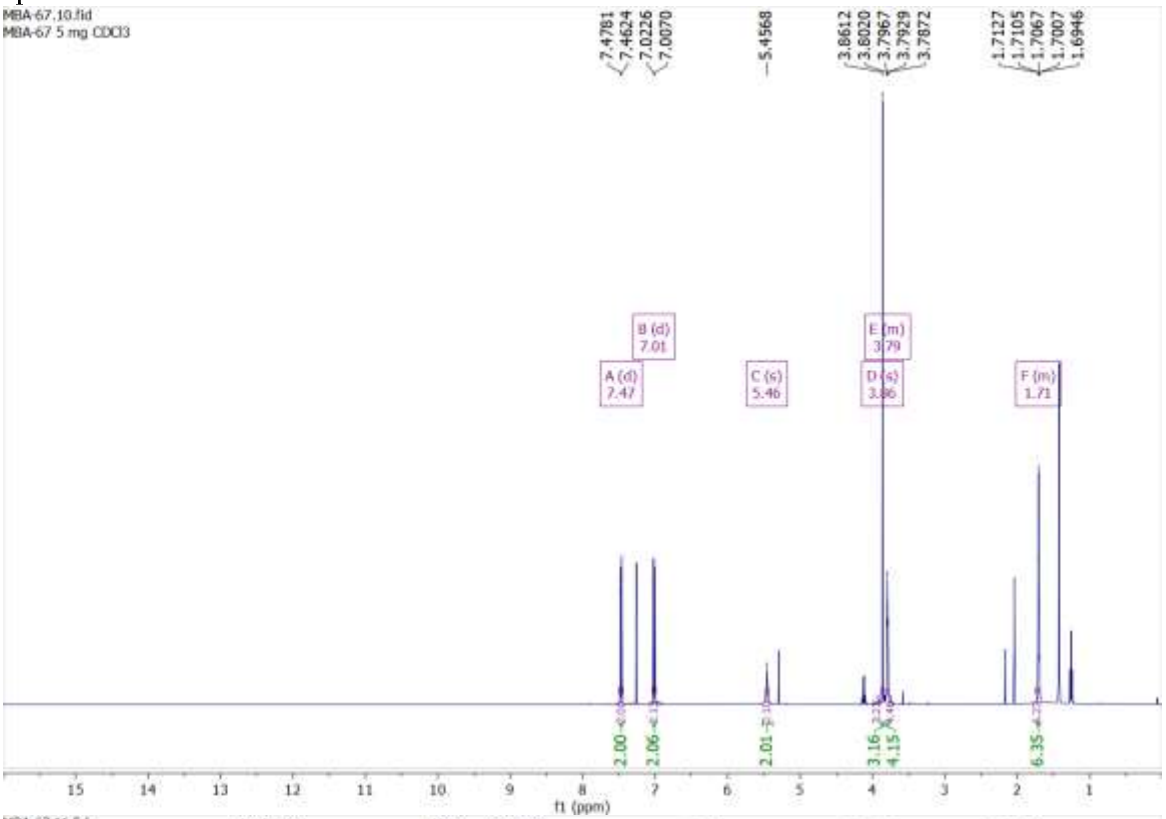
MBA-67.10.fid
MBA-67 5 mg CDCl3
7.4781
7.4624
7.0226
7.0070
5.4568
3.8612
3.8020
3.7967
3.7929
3.7872
1.7127
1.7105
1.7067
1.7007
1.6946
A (d)
7.47
B (d)
7.01
C (s)
5.46
D (s)
3.86
E (m)
3.79
F (m)
1.71
2.00
2.06
2.01
3.16
4.15
6.35
f1 (ppm)

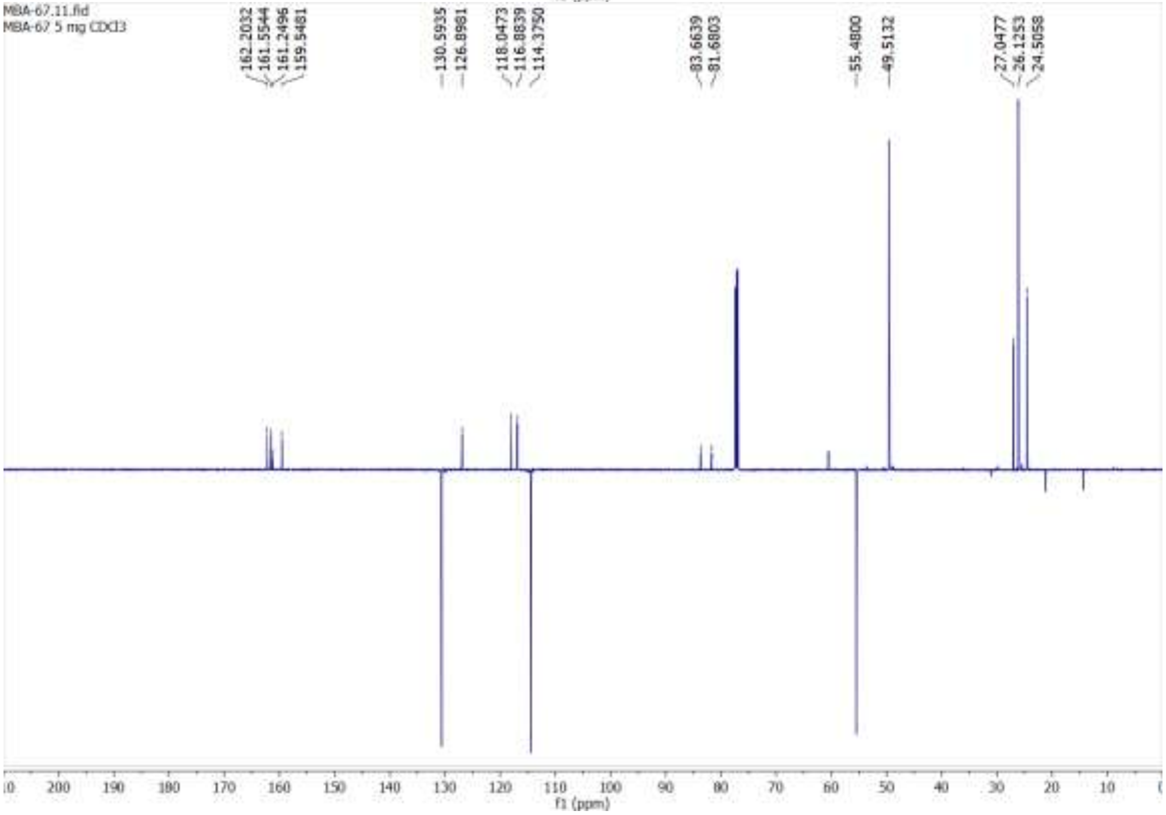
MBA-67.11.fid
MBA-67 5 mg CDCl3
162.2032
161.5544
161.2496
159.5481
130.5935
126.8981
118.0473
116.8839
114.3750
83.6639
81.6803
55.4800
49.5132
27.0477
26.1253
24.5058
f1 (ppm)

Compound 22:

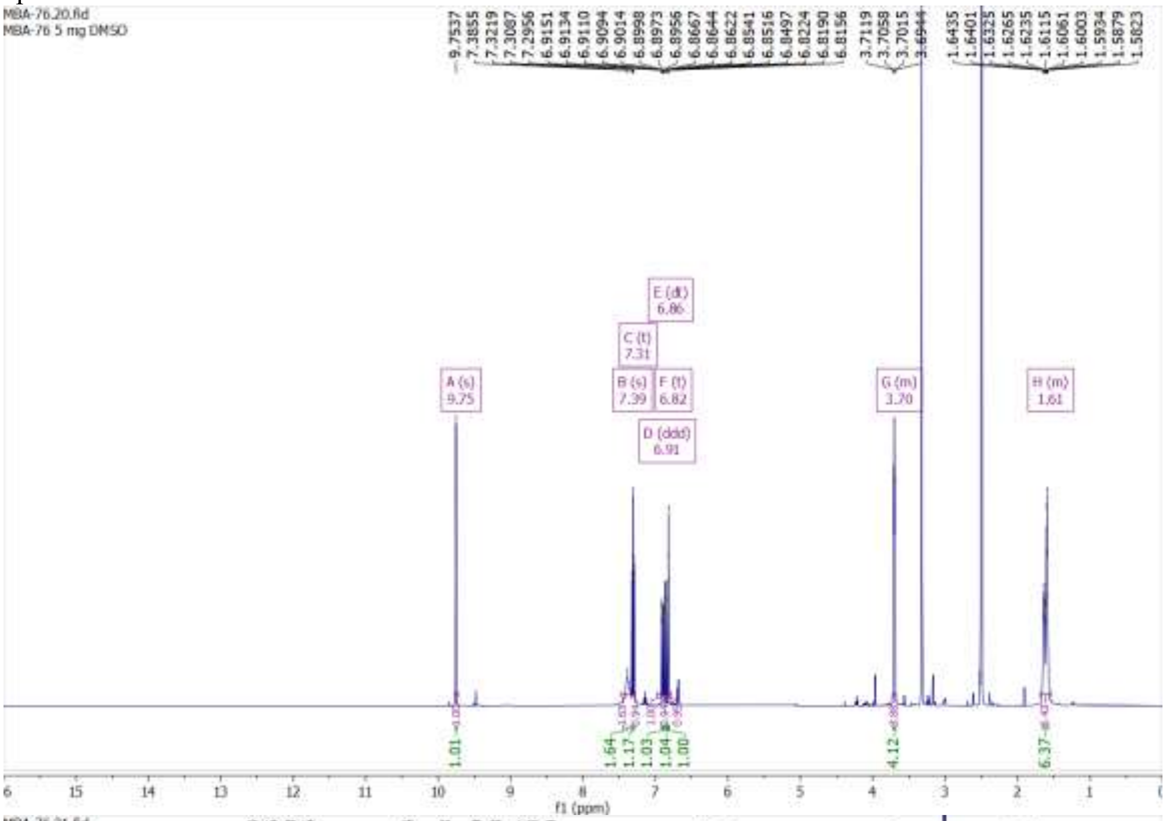
MBA-76.20.fid
MBA-76 5 mg DMSO
A (s) 9.75
B (s) 7.39
C (t) 7.31
D (ddd) 6.91
E (dt) 6.86
F (t) 6.82
G (m) 3.70
H (m) 1.61
f1 (ppm)

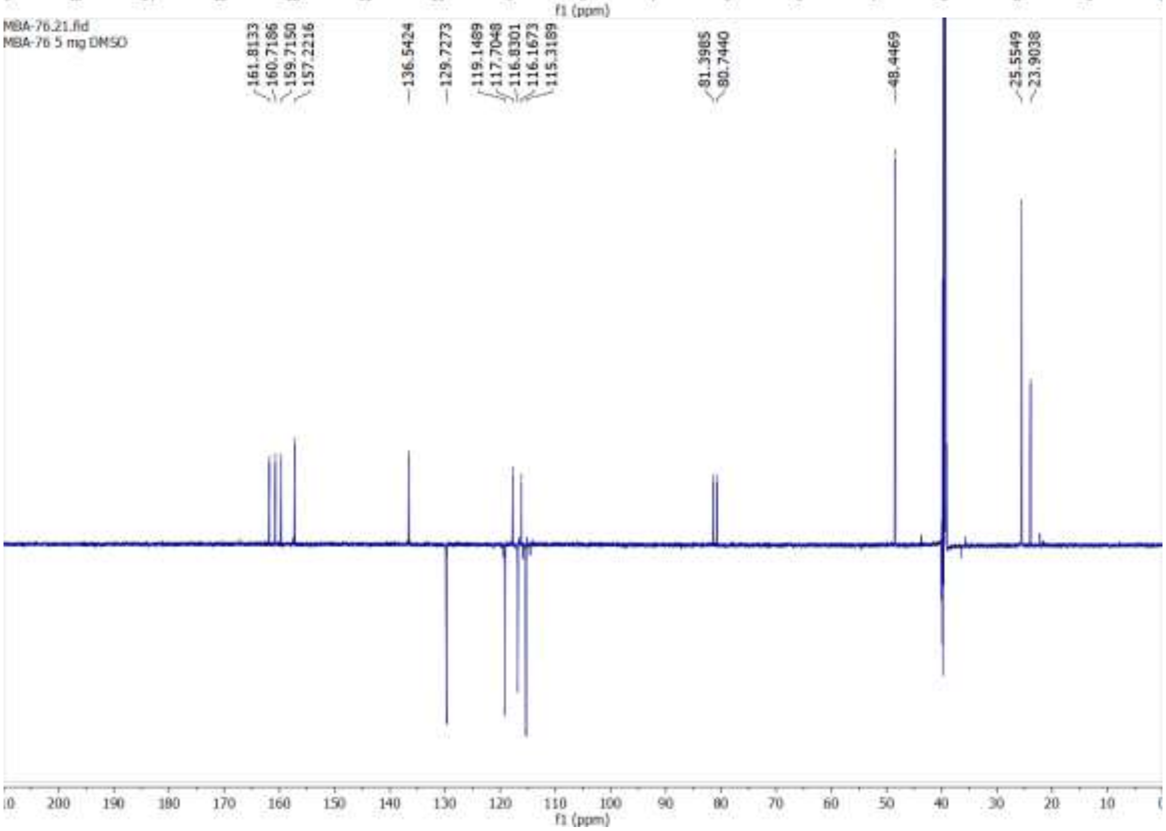
MBA-76.21.fid
MBA-76 5 mg DMSO
161.8133
160.7186
159.7150
157.2216
136.5424
129.7273
119.1489
117.7048
116.8301
116.1673
115.3189
81.3985
80.7440
48.4469
25.5549
23.9038
f1 (ppm)

Compound 23:

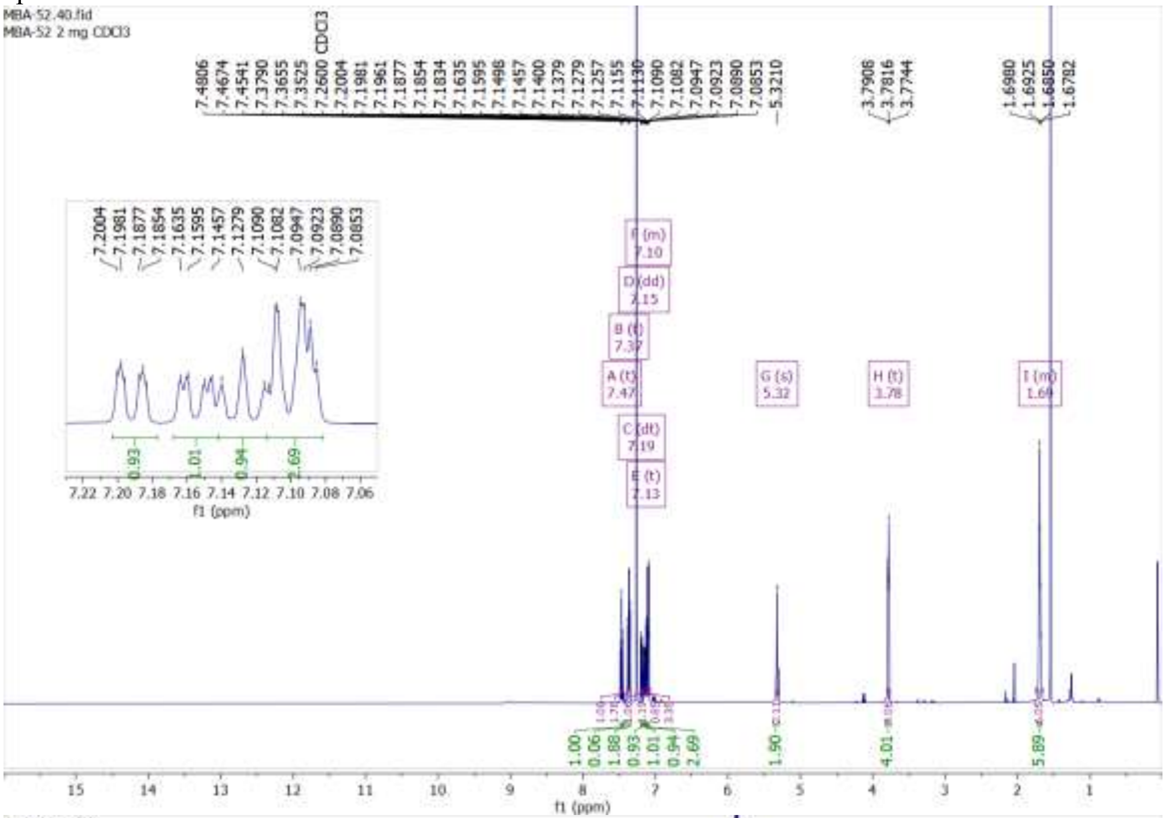
MBA-52.40.fid
MBA-52 2 mg CDCl3
7.4806
7.4674
7.4541
7.3790
7.3655
7.3525
7.2600 CDCl3
7.2004
7.1981
7.1961
7.1877
7.1854
7.1834
7.1635
7.1595
7.1498
7.1457
7.1400
7.1379
7.1279
7.1257
7.1155
7.1130
7.1090
7.1082
7.0947
7.0923
7.0890
7.0853
5.3210
3.7908
3.7816
3.7744
1.6980
1.6925
1.6850
1.6782
F (m) 7.10
D (dd) 7.15
B (t) 7.37
A (t) 7.47
C (dt) 7.19
E (t) 7.13
G (s) 5.32
H (t) 3.78
I (m) 1.69
1.00
0.06
1.88
0.93
1.01
0.94
2.69
1.90
4.01
5.89
f1 (ppm)

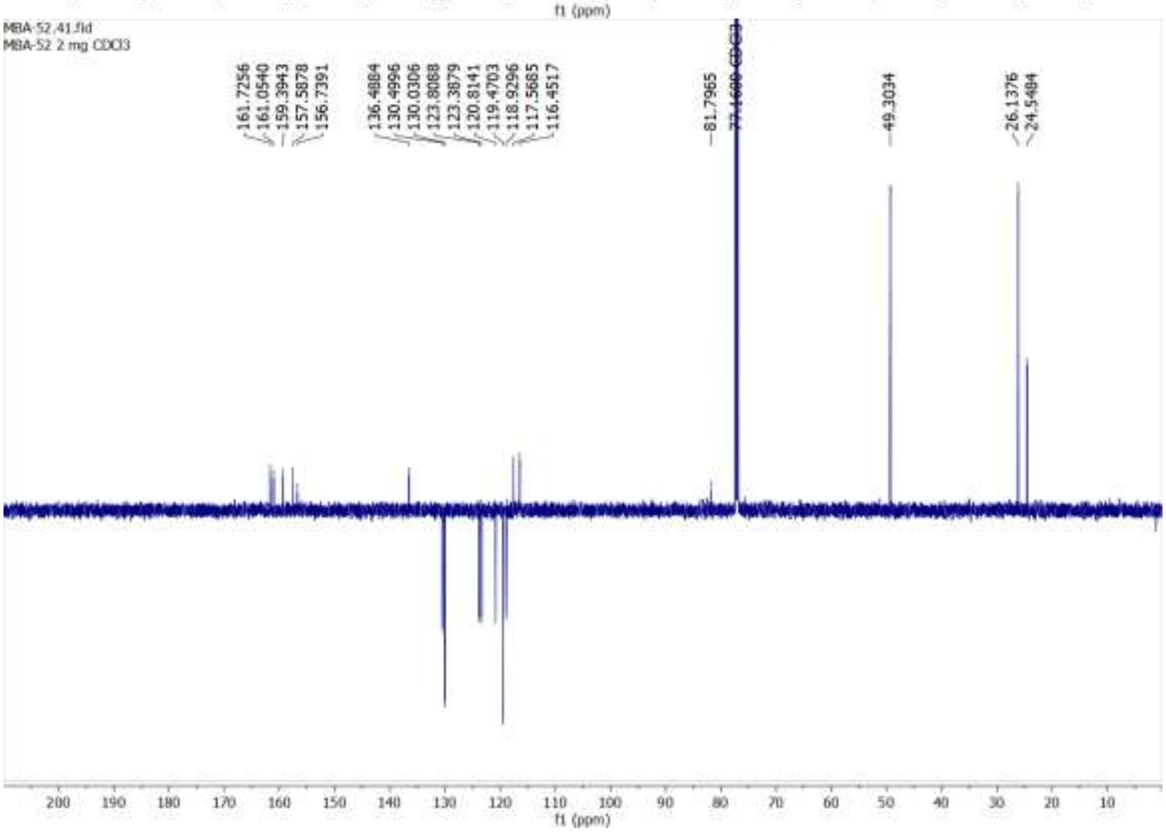
MBA-52.41.fid
MBA-52 2 mg CDCl3
161.7256
161.0540
159.3943
157.5878
156.7391
136.4884
130.4996
130.0306
123.8088
123.3879
120.8141
119.4703
118.9296
117.5685
116.4517
81.7965
77.1600 CDCl3
49.3034
26.1376
24.5484
f1 (ppm)

Compound 24:

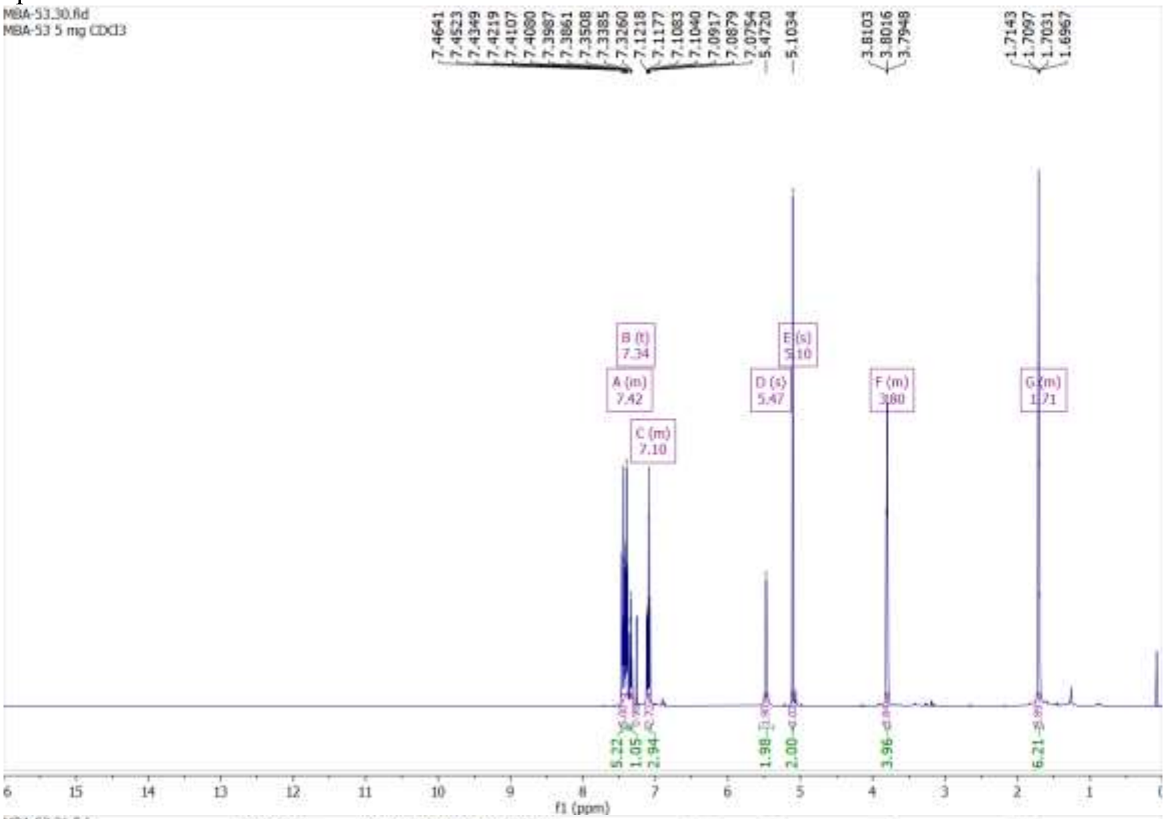
MBA-53.30.fid
MBA-53 5 mg CDCl3
7.4641
7.4523
7.4349
7.4219
7.4107
7.4080
7.3987
7.3861
7.3508
7.3385
7.3260
7.1218
7.1177
7.1083
7.1040
7.0917
7.0879
7.0754
5.4720
5.1034
3.8103
3.8016
3.7948
1.7143
1.7097
1.7031
1.6967
B (t) 7.34
A (m) 7.42
C (m) 7.10
D (s) 5.47
E (s) 5.10
F (m) 3.80
G (m) 1.71
5.22
1.05
2.94
1.98
2.00
3.96
6.21
f1 (ppm)

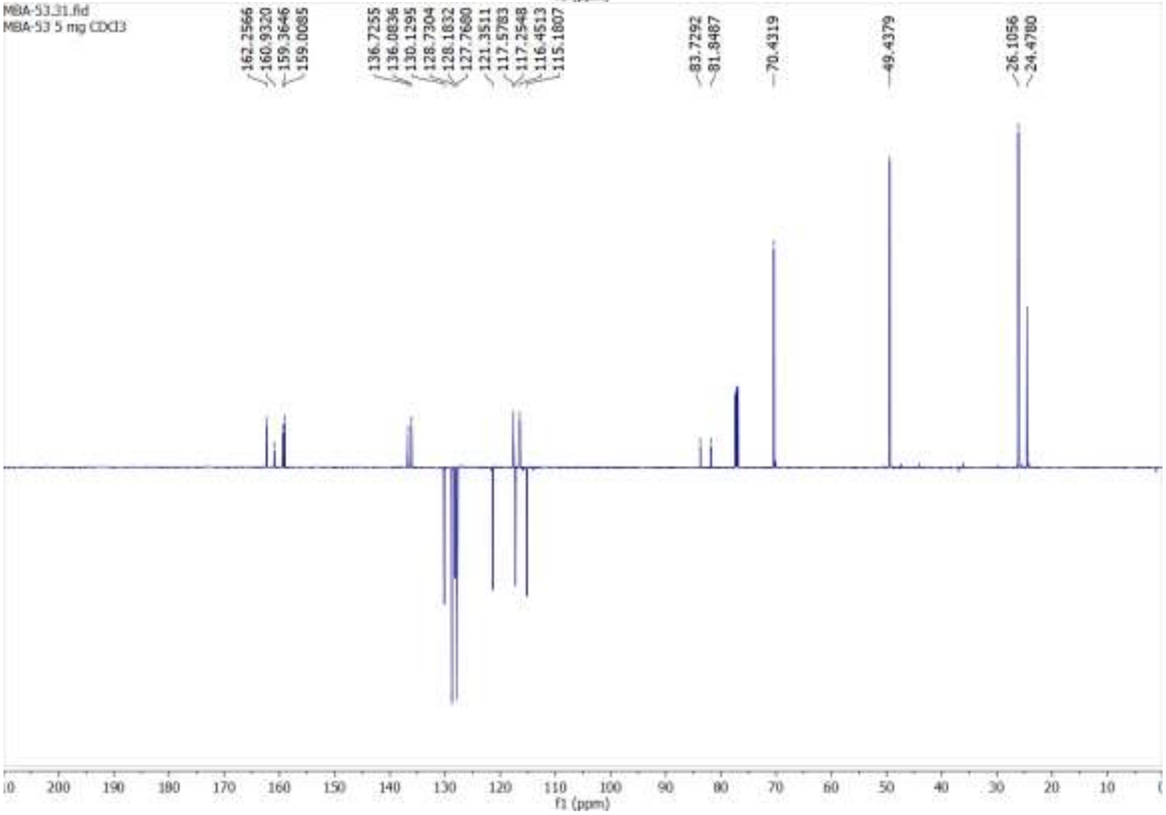
MBA-53.31.fid
MBA-53 5 mg CDCl3
162.2566
160.9320
159.3646
159.0085
136.7255
136.0836
130.1295
128.7304
128.1832
127.7680
121.3511
117.5783
117.2548
116.4513
115.1807
83.7292
81.8487
70.4319
49.4379
26.1056
24.4780
f1 (ppm)

Compound 25:

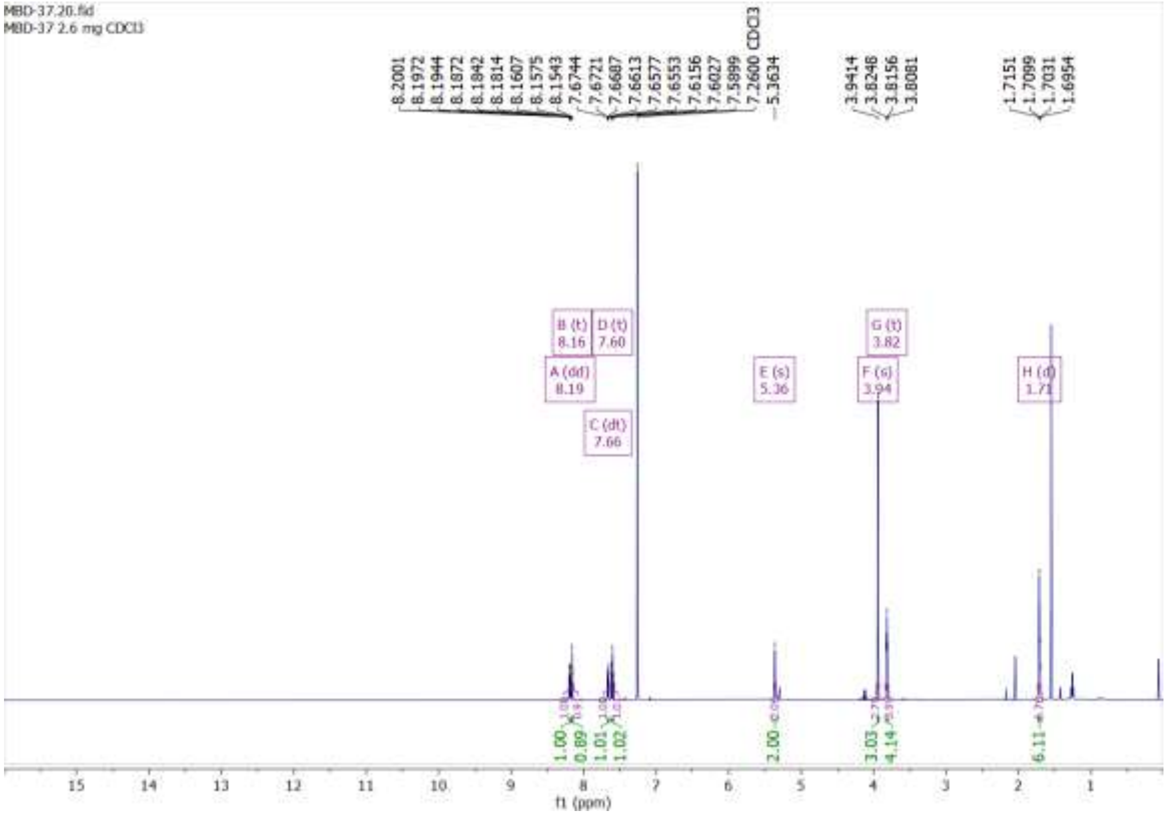

MBD-37.20.fid
MBD-37 2.6 mg CDCl3
8.2001
8.1972
8.1944
8.1872
8.1842
8.1814
8.1607
8.1575
8.1543
7.6744
7.6721
7.6687
7.6613
7.6577
7.6553
7.6156
7.6027
7.5899
7.2600 CDCl3
5.3634
3.9414
3.8248
3.8156
3.8081
1.7151
1.7099
1.7031
1.6954
B (t) 8.16
D (t) 7.60
A (dd) 8.19
C (dt) 7.66
E (s) 5.36
G (t) 3.82
F (s) 3.94
H (d) 1.71
1.00
0.89
1.01
1.02
2.00
3.03
4.14
6.11
15
14
13
12
11
10
9
8
7
6
5
4
3
2
1
f1 (ppm)

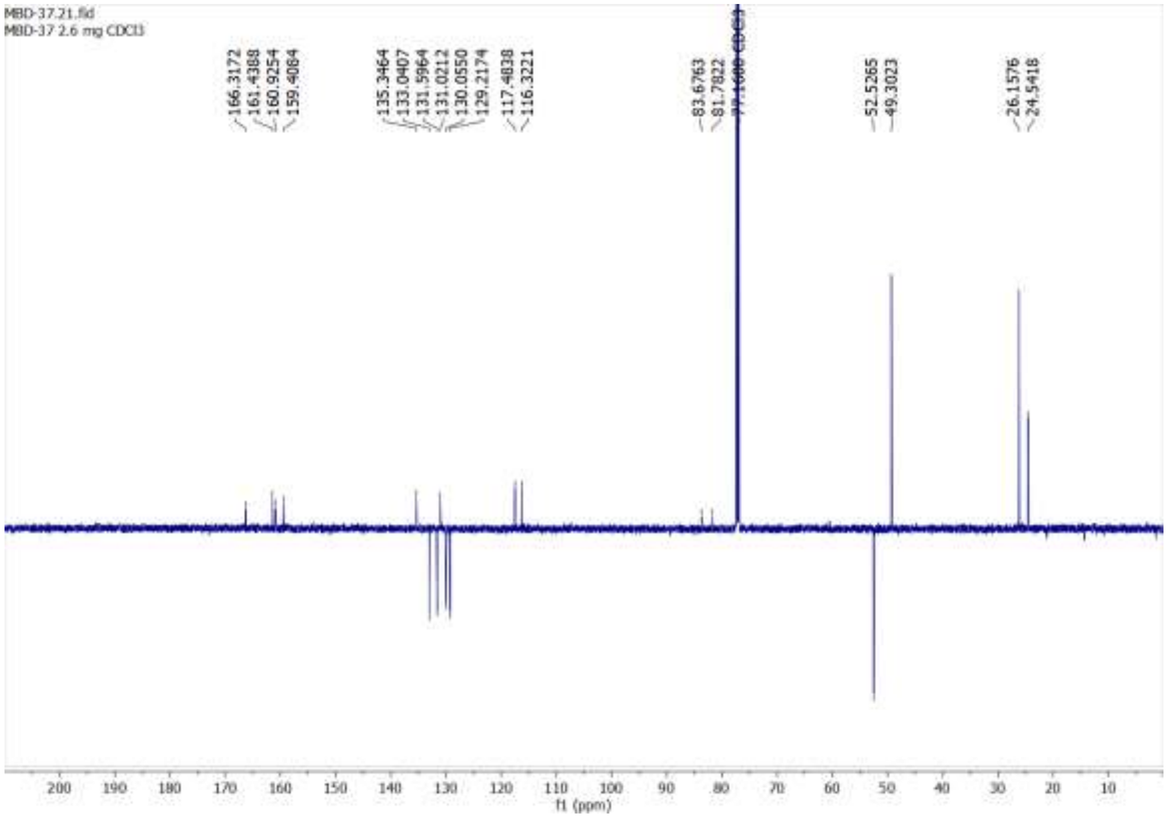

MBD-37.21.fid
MBD-37 2.6 mg CDCl3
166.3172
161.4388
160.9254
159.4084
135.3464
133.0407
131.5964
131.0212
130.0550
129.2174
117.4838
116.3221
83.6763
81.7822
77.1600 CDCl3
52.5265
49.3023
26.1576
24.5418
200
190
180
170
160
150
140
130
120
110
100
90
80
70
60
50
40
30
20
10
f1 (ppm)

Compound 26:

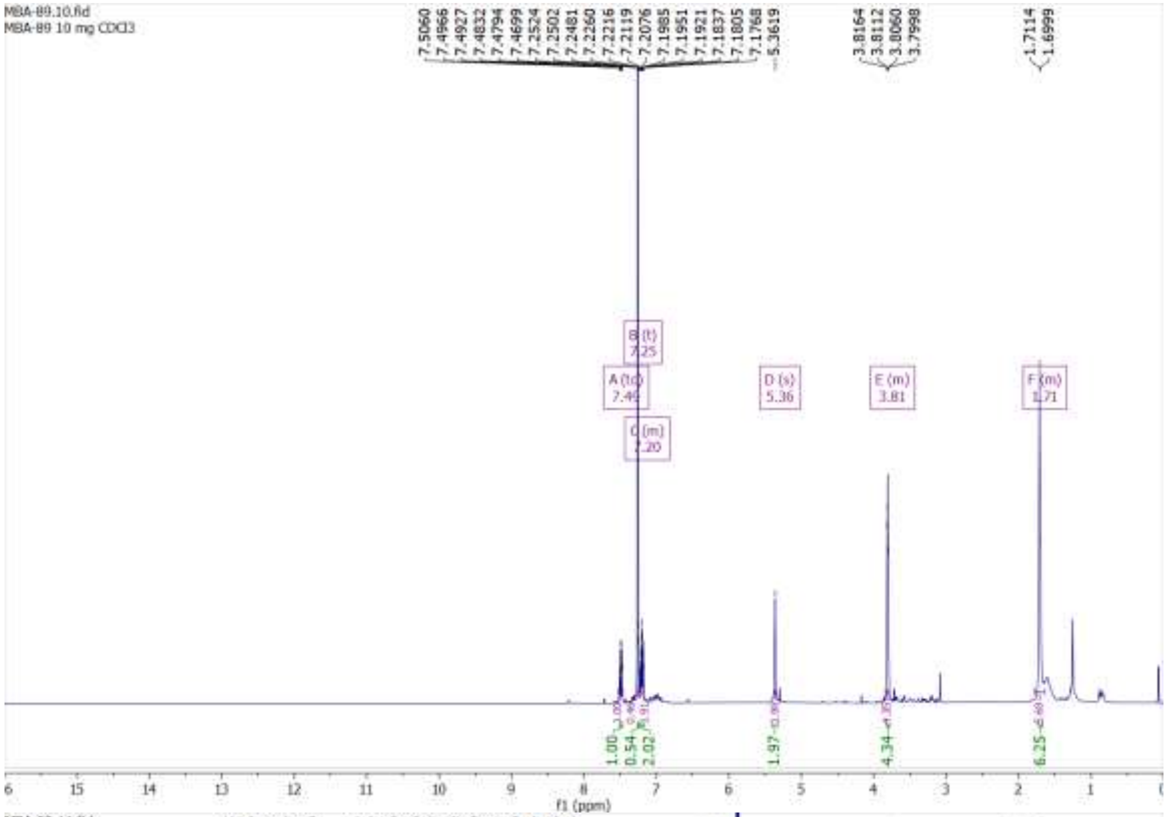

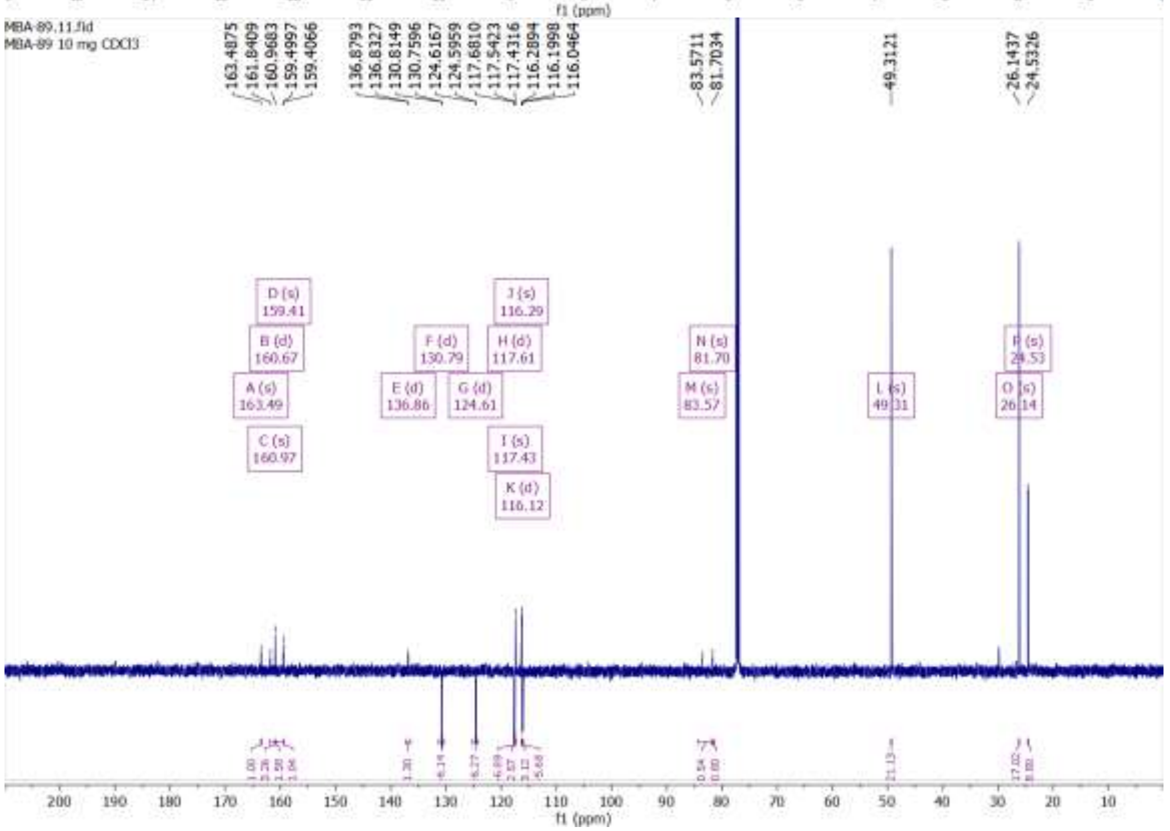

Compound 27:

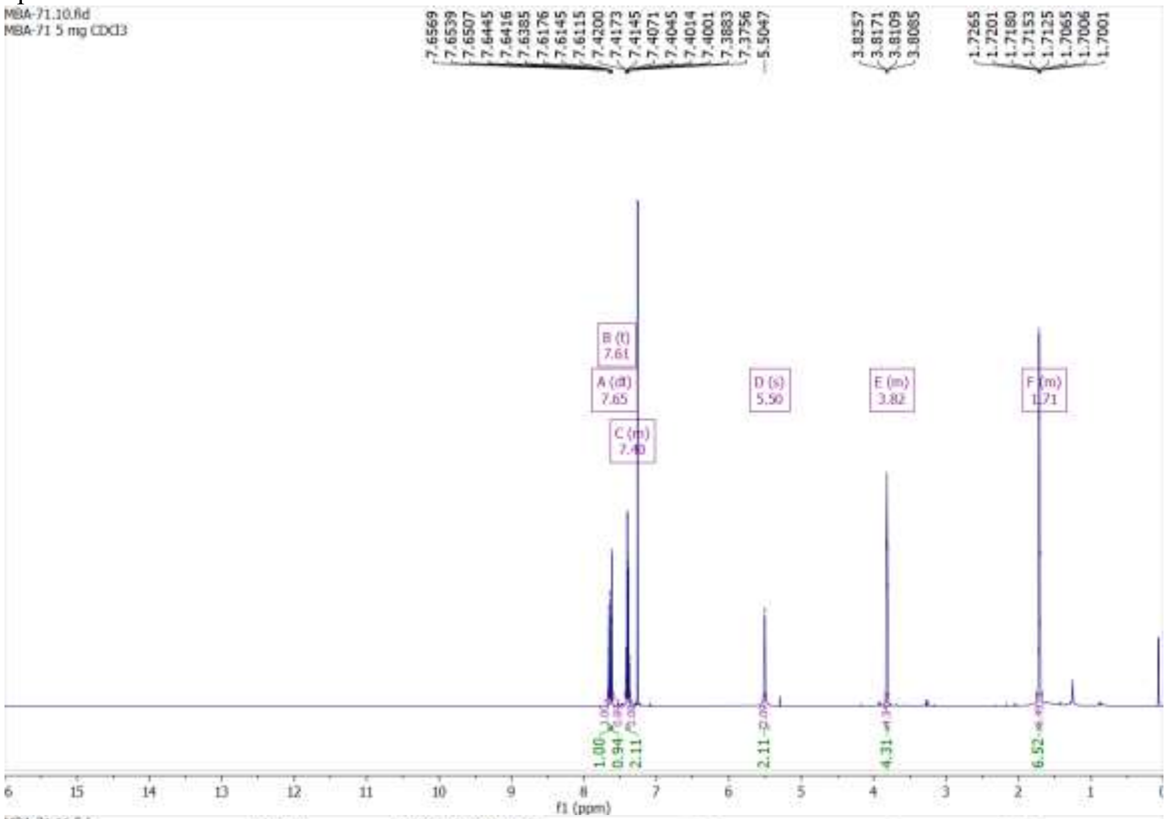
MBA-71.10.fid
MBA-71 5 mg CDCl3
7.6569
7.6539
7.6507
7.6445
7.6416
7.6385
7.6176
7.6145
7.6115
7.4200
7.4173
7.4145
7.4071
7.4045
7.4014
7.4001
7.3883
7.3756
5.5047
3.8257
3.8171
3.8109
3.8085
1.7265
1.7201
1.7180
1.7153
1.7125
1.7065
1.7006
1.7001
B (t)
7.61
A (dt)
7.65
C (m)
7.40
D (s)
5.50
E (m)
3.82
F (m)
1.71
1.00
0.94
2.11
2.11
4.31
6.52
f1 (ppm)

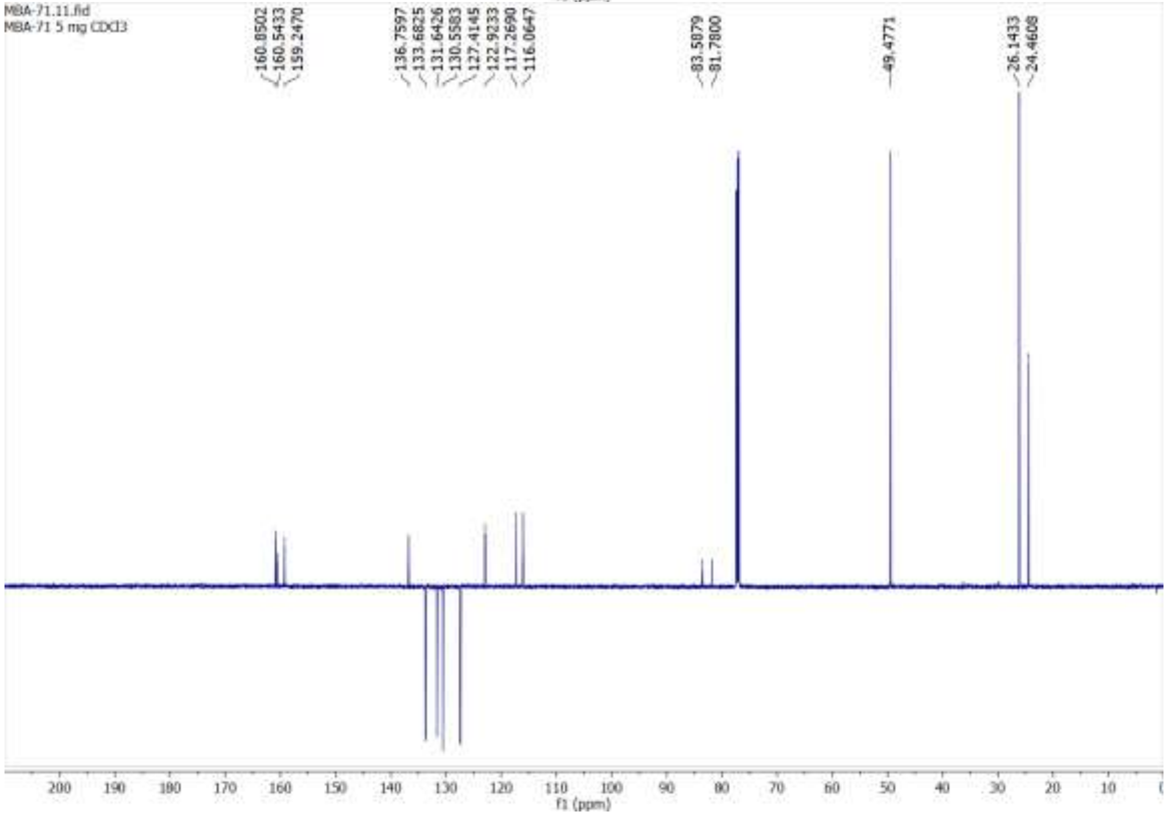
MBA-71.11.fid
MBA-71 5 mg CDCl3
160.8502
160.5433
159.2470
136.7597
133.6825
131.6426
130.5583
127.4145
122.9233
117.2690
116.0647
83.5079
81.7800
49.4771
26.1433
24.4608
f1 (ppm)

Compound 28:

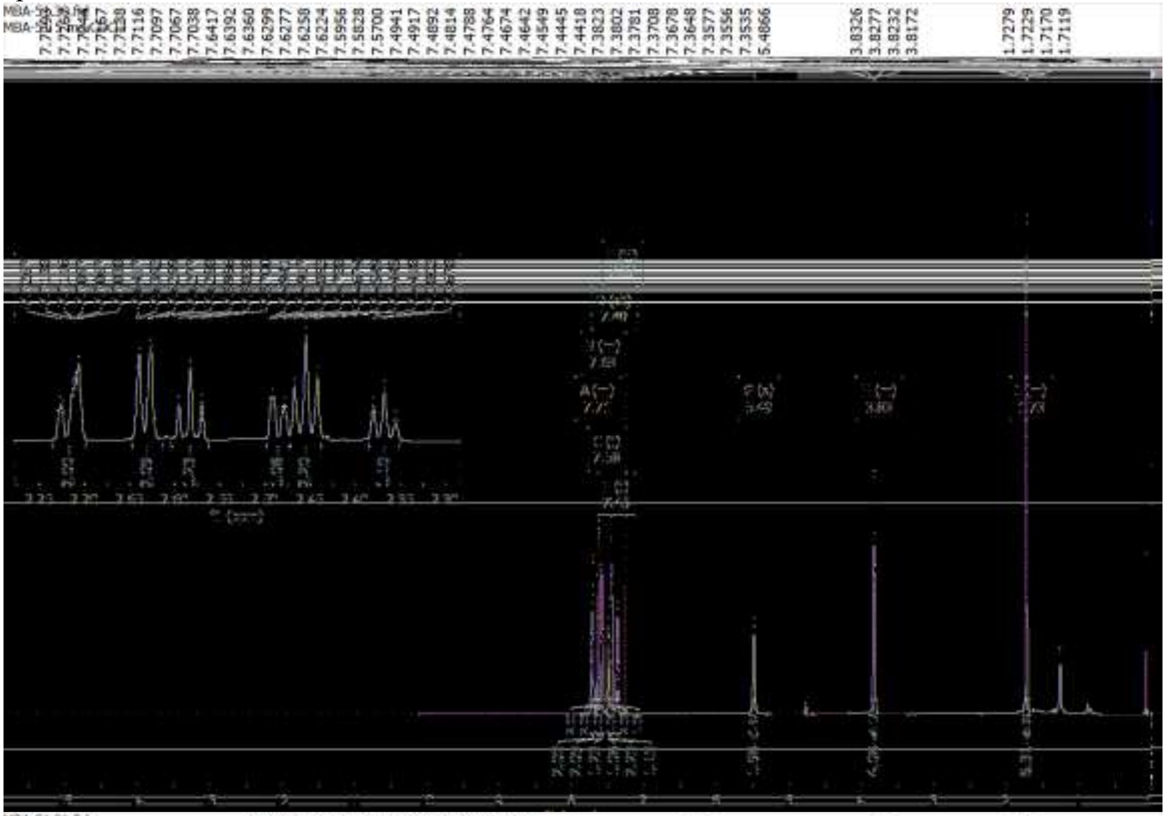

7.7116
7.7097
7.7067
7.7038
7.6417
7.6392
7.6360
7.6299
7.6277
7.6258
7.6224
7.5956
7.5828
7.5700
7.4941
7.4917
7.4892
7.4814
7.4788
7.4764
7.4674
7.4642
7.4549
7.4445
7.4418
7.3823
7.3802
7.3781
7.3708
7.3678
7.3648
7.3577
7.3556
7.3535
5.4866
3.8326
3.8277
3.8232
3.8172
1.7279
1.7229
1.7170
1.7119

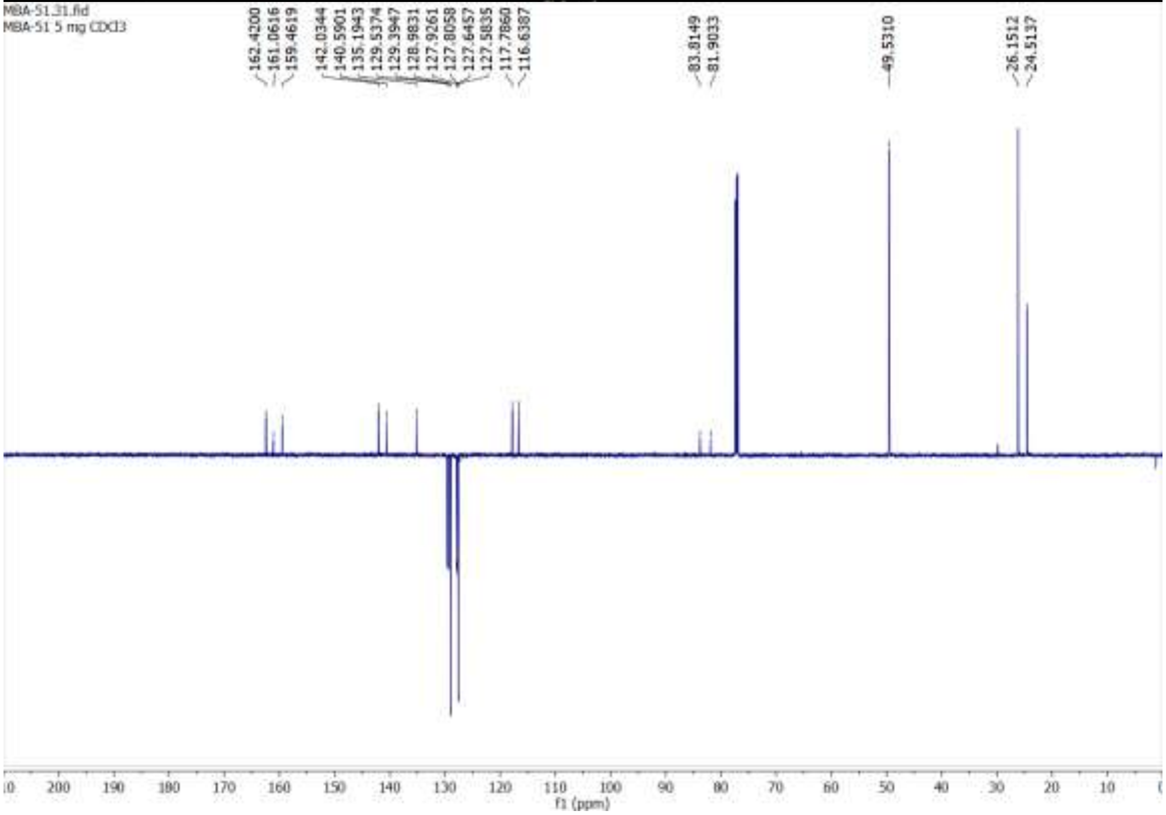

MBA-51.31.fid
MBA-51 5 mg CDCl3
162.4200
161.0616
159.4619
142.0344
140.5901
135.1943
129.5374
129.3947
128.9831
127.9261
127.8058
127.6457
127.5835
117.7860
116.6387
83.8149
81.9033
49.5310
26.1512
24.5137
200 190 180 170 160 150 140 130 120 110 100 90 80 70 60 50 40 30 20 10
f1 (ppm)

Compound 29:

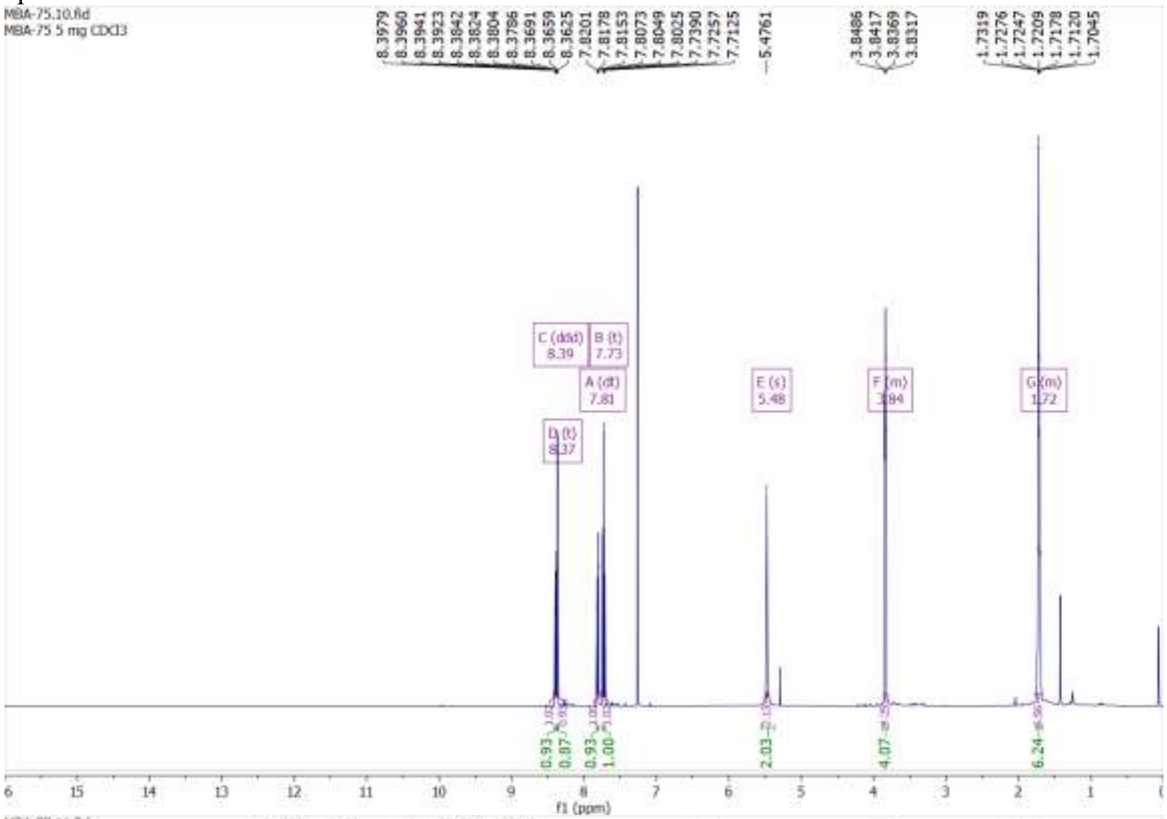
MBA-75.10.fid
MBA-75 5 mg CDCl3
8.3979
8.3960
8.3941
8.3923
8.3842
8.3824
8.3804
8.3786
8.3691
8.3659
8.3625
7.8201
7.8178
7.8153
7.8073
7.8049
7.8025
7.7390
7.7257
7.7125
5.4761
3.8486
3.8417
3.8369
3.8317
1.7319
1.7276
1.7247
1.7209
1.7178
1.7120
1.7045
C (ddd)
8.39
B (t)
7.73
A (dt)
7.81
D (t)
8.37
E (s)
5.48
F (m)
3.84
G (m)
1.72
0.93
0.87
0.93
1.00
2.03
4.07
6.24
f1 (ppm)

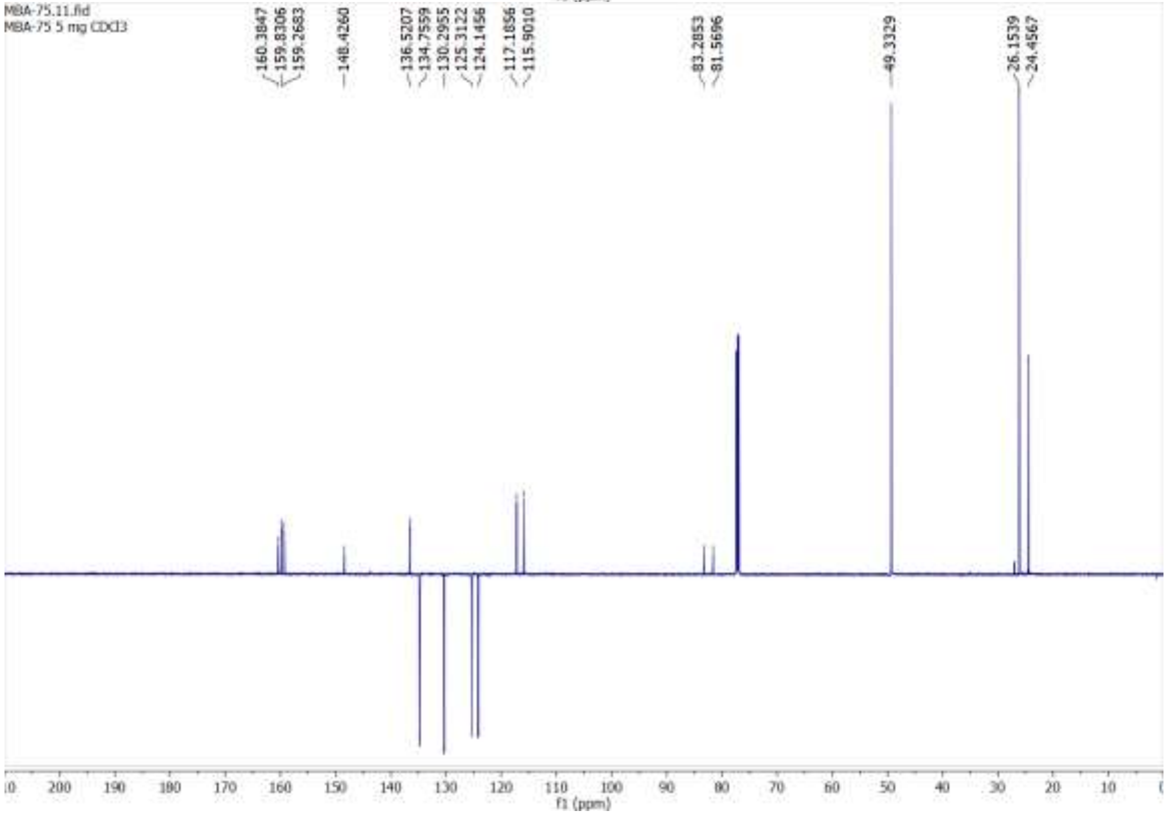
MBA-75.11.fid
MBA-75 5 mg CDCl3
160.3847
159.8306
159.2683
148.4260
136.5207
134.7559
130.2955
125.3122
124.1456
117.1856
115.9010
83.2853
81.5696
49.3329
26.1539
24.4567
f1 (ppm)

Compound 30:

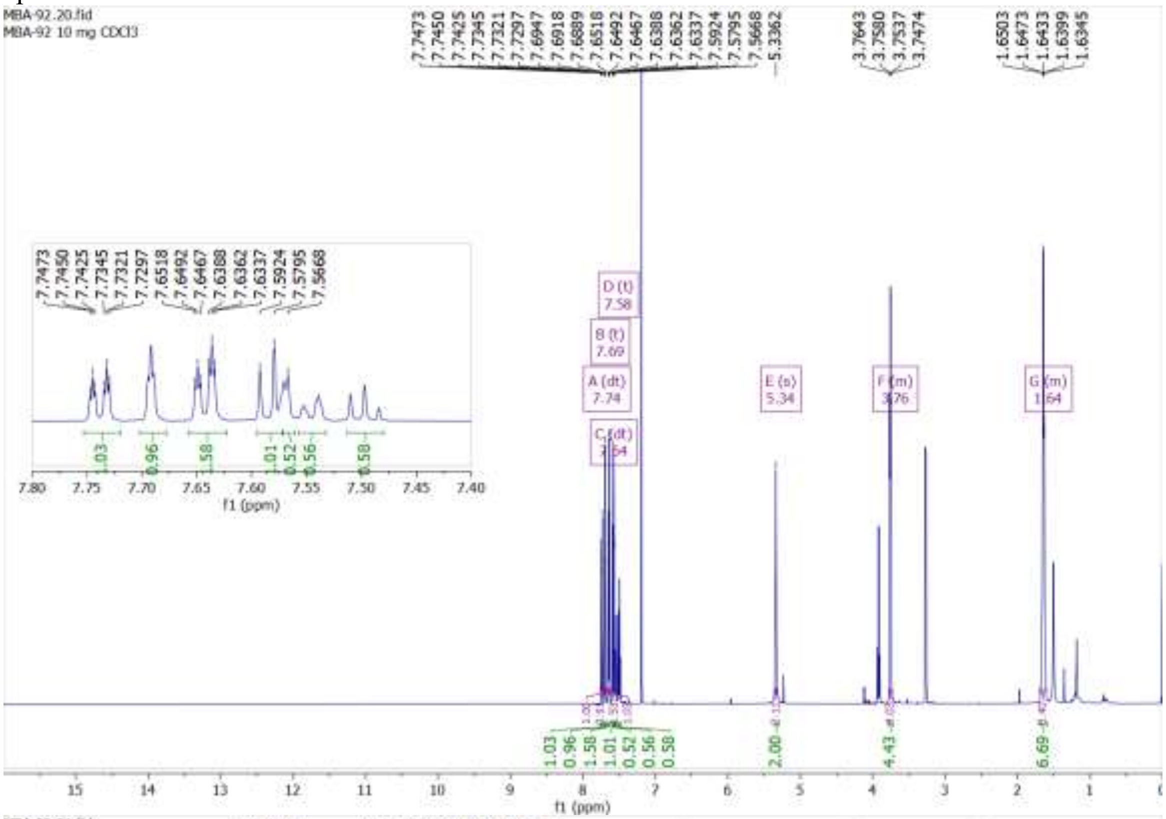
MBA-92.20.fid
MBA-92 10 mg CDCl3
7.7473
7.7450
7.7425
7.7345
7.7321
7.7297
7.6947
7.6918
7.6889
7.6518
7.6492
7.6467
7.6388
7.6362
7.6337
7.5924
7.5795
7.5668
5.3362
3.7643
3.7580
3.7537
3.7474
1.6503
1.6473
1.6433
1.6399
1.6345
D (t)
7.58
B (t)
7.69
A (dt)
7.74
C (dt)
E (s)
5.34
F (m)
G (m)
1.64
1.03
0.96
1.58
1.01
0.52
0.56
0.58
2.00
4.43
6.69
f1 (ppm)

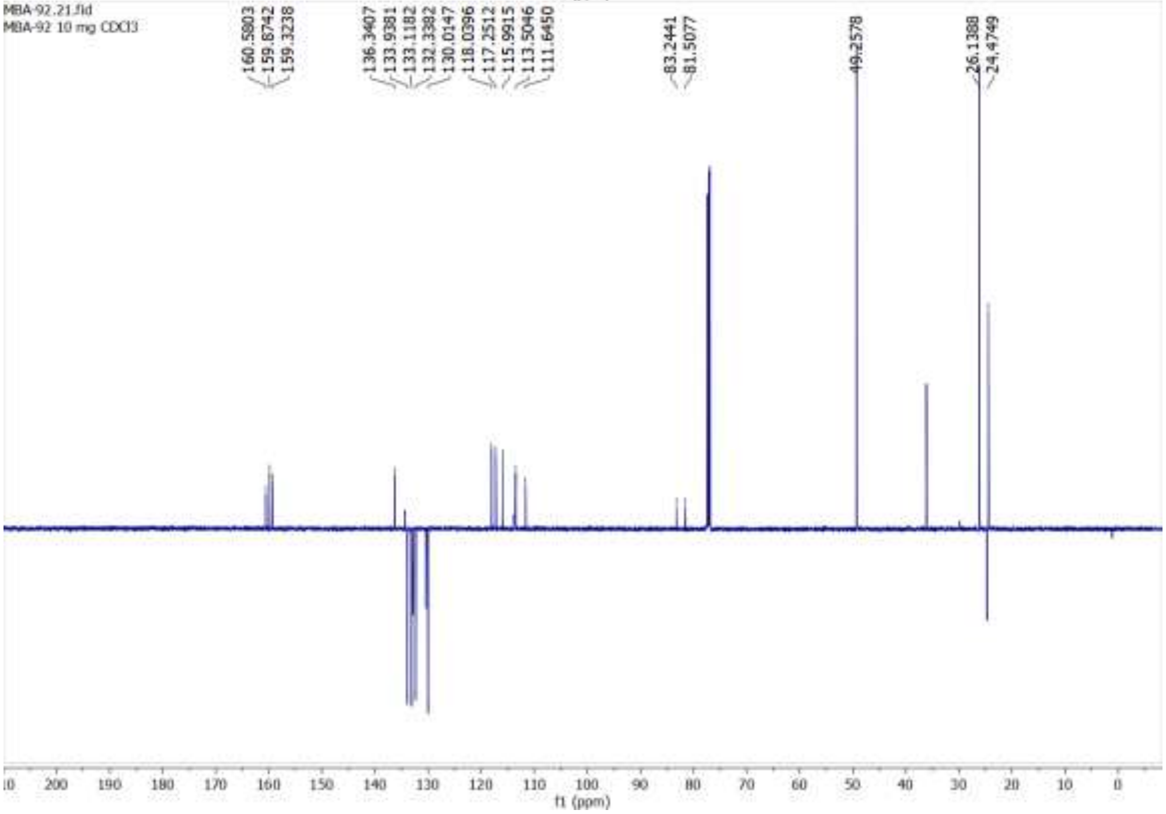
MBA-92.21.fid
MBA-92 10 mg CDCl3
160.5803
159.8742
159.3238
136.3407
133.9381
133.1182
132.3382
130.0147
118.0396
117.2512
115.9915
113.5046
111.6450
83.2441
81.5077
49.2578
26.1388
24.4749
f1 (ppm)

Compound 31:

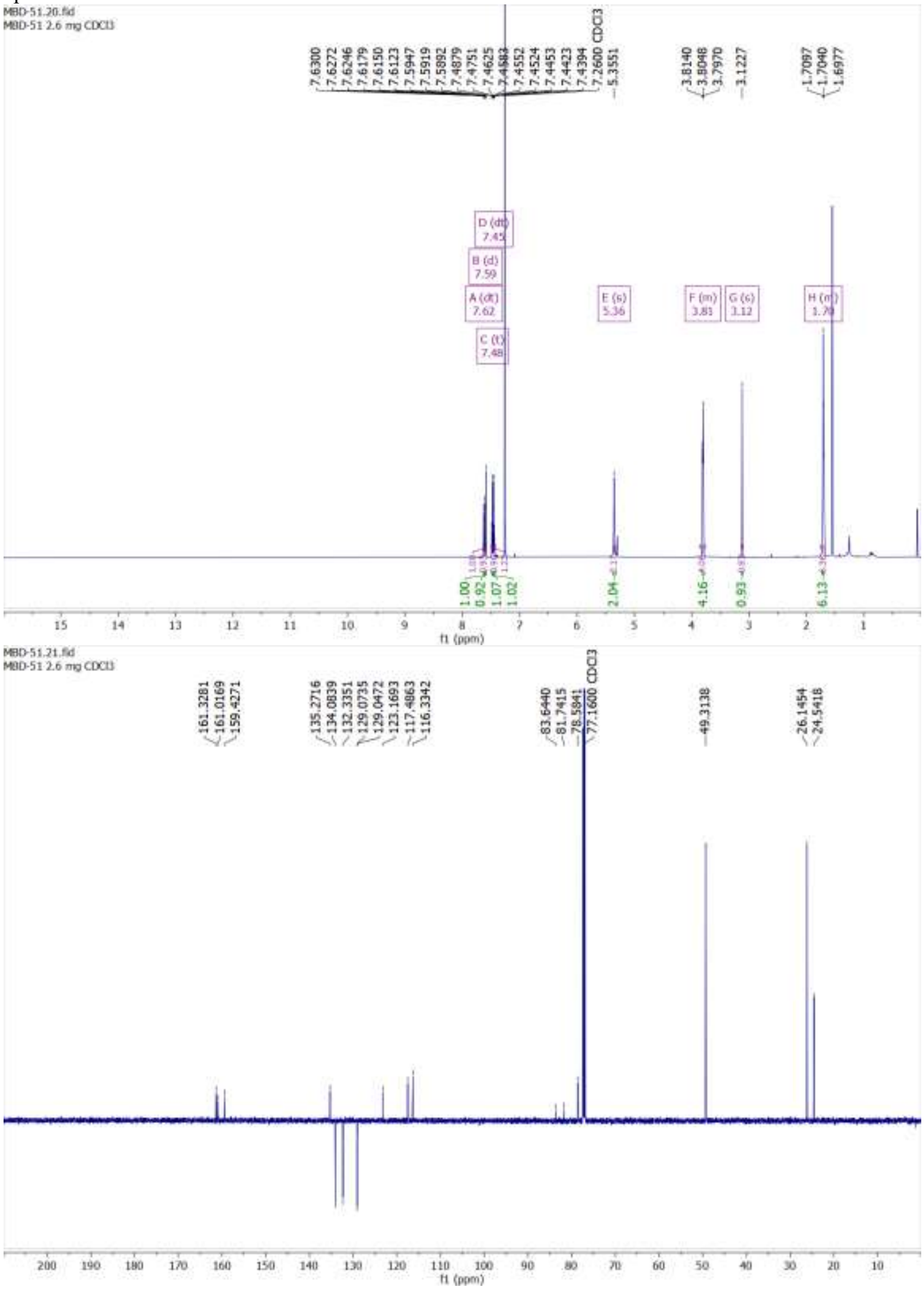
MBD-51.20.fid
MBD-51 2.6 mg CDCl3
7.6300
7.6272
7.6246
7.6179
7.6150
7.6123
7.5947
7.5919
7.5892
7.4879
7.4751
7.4625
7.4552
7.4524
7.4453
7.4423
7.4394
7.2600 CDCl3
5.3551
3.8140
3.8048
3.7970
3.1227
1.7097
1.7040
1.6977
D (dt)
7.45
B (d)
7.59
A (dt)
7.62
C (t)
7.48
E (s)
5.36
F (m)
3.81
G (s)
3.12
H (m)
1.70
1.00
0.92
1.07
1.02
2.04
4.16
0.93
6.13
f1 (ppm)
MBD-51.21.fid
MBD-51 2.6 mg CDCl3
161.3281
161.0169
159.4271
135.2716
134.0839
132.3351
129.0735
129.0472
123.1693
117.4863
116.3342
83.6440
81.7415
78.5841
77.1600 CDCl3
49.3138
26.1454
24.5418
f1 (ppm)

Compound 32:

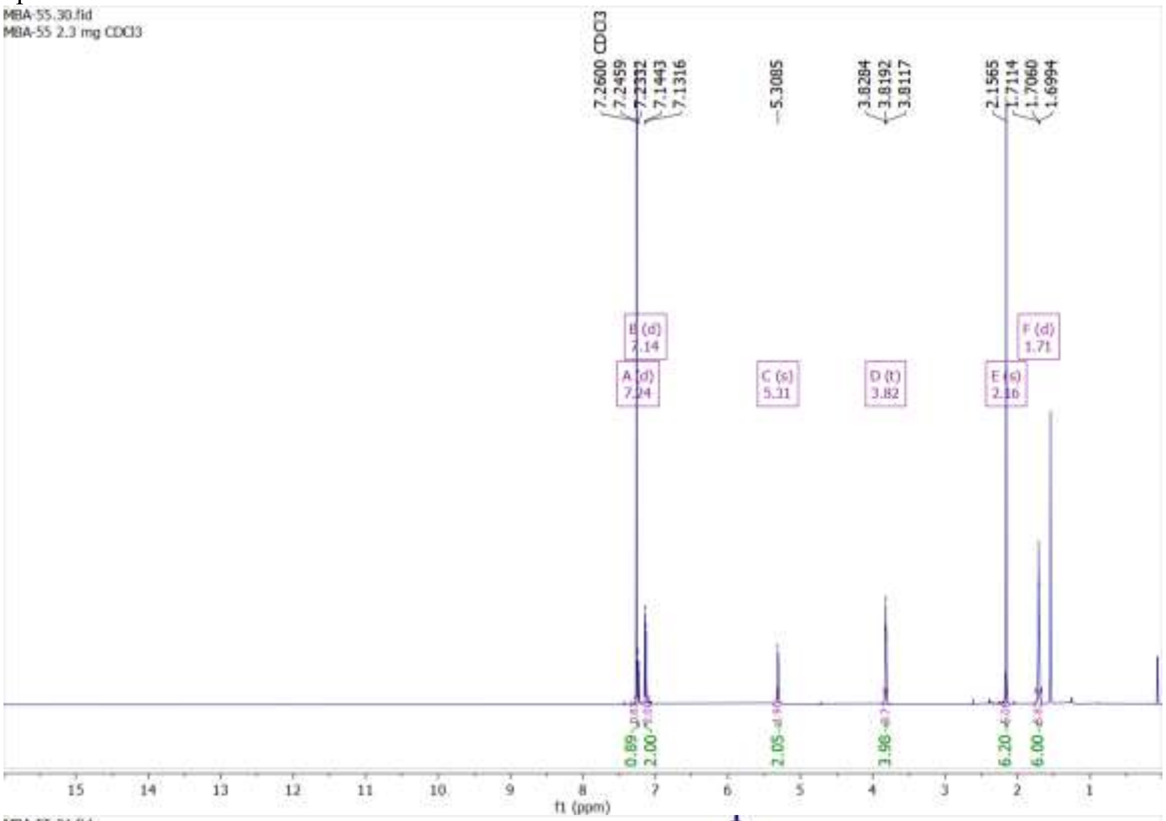
MBA-55.30.fid
MBA-55 2.3 mg CDCl3
7.2600 CDCl3
7.2459
7.2332
7.1443
7.1316
5.3085
3.8284
3.8192
3.8117
2.1565
1.7114
1.7060
1.6994
B (d) 7.14
A (d) 7.24
C (s) 5.31
D (t) 3.82
E (s) 2.16
F (d) 1.71
0.89
2.00
2.05
3.98
6.20
6.00
f1 (ppm)

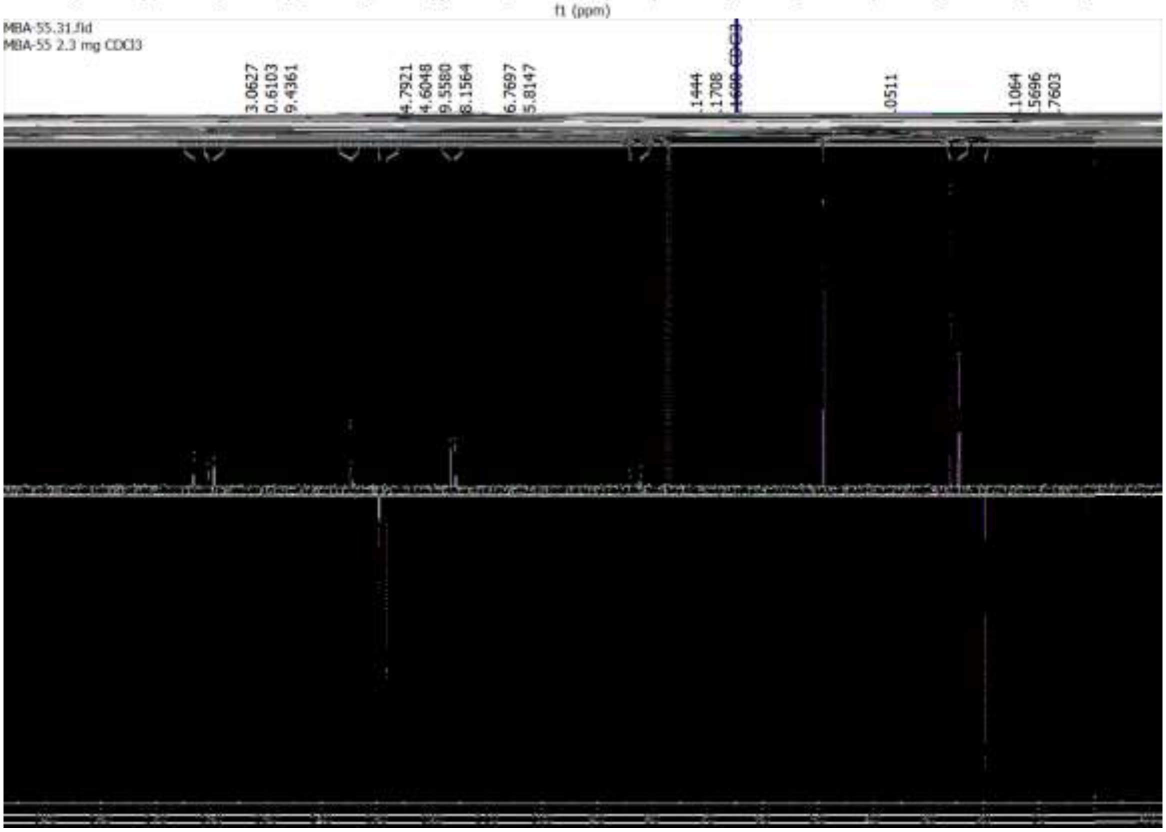
MBA-55.31.fid
MBA-55 2.3 mg CDCl3

Compound 33:

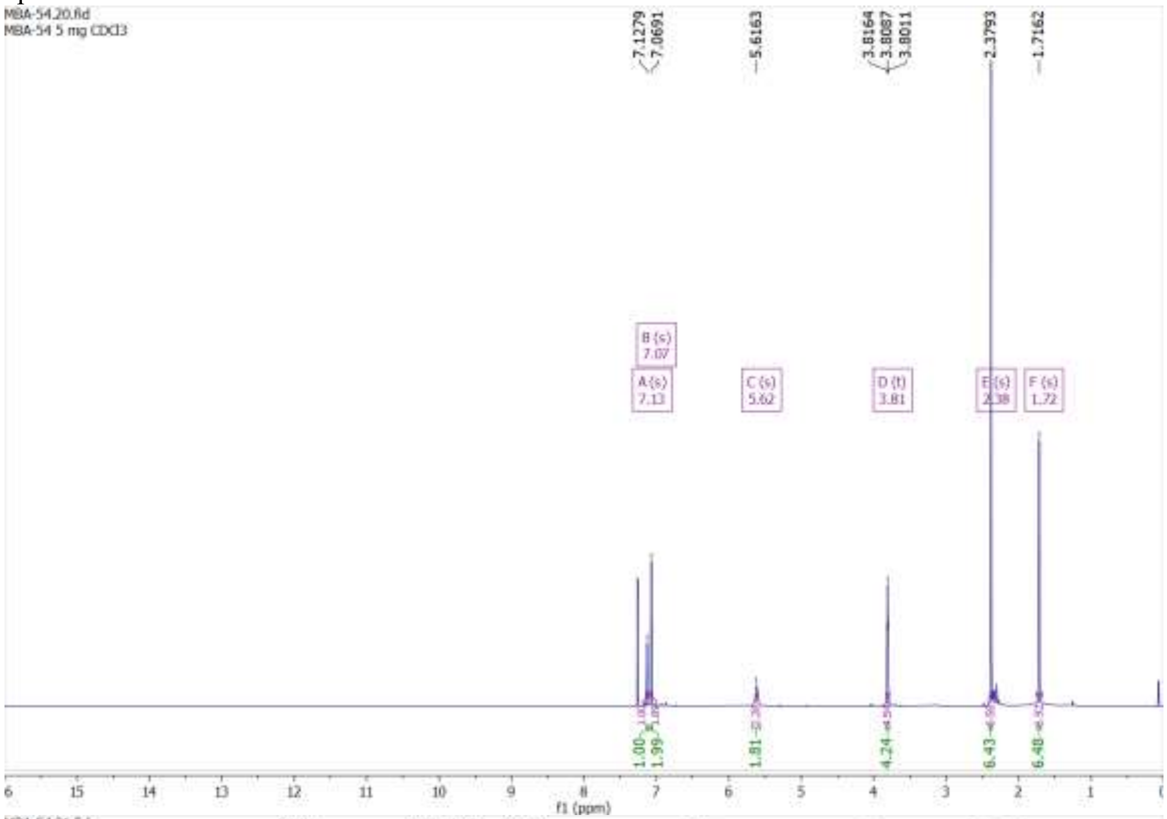
MBA-54.20.fid
MBA-54 5 mg CDCl3
7.1279
7.0691
5.6163
3.8164
3.8087
3.8011
2.3793
1.7162
B (s) 7.07
A (s) 7.13
C (s) 5.62
D (t) 3.81
E (s) 2.38
F (s) 1.72
1.00
1.99
1.81
4.24
6.43
6.48
f1 (ppm)

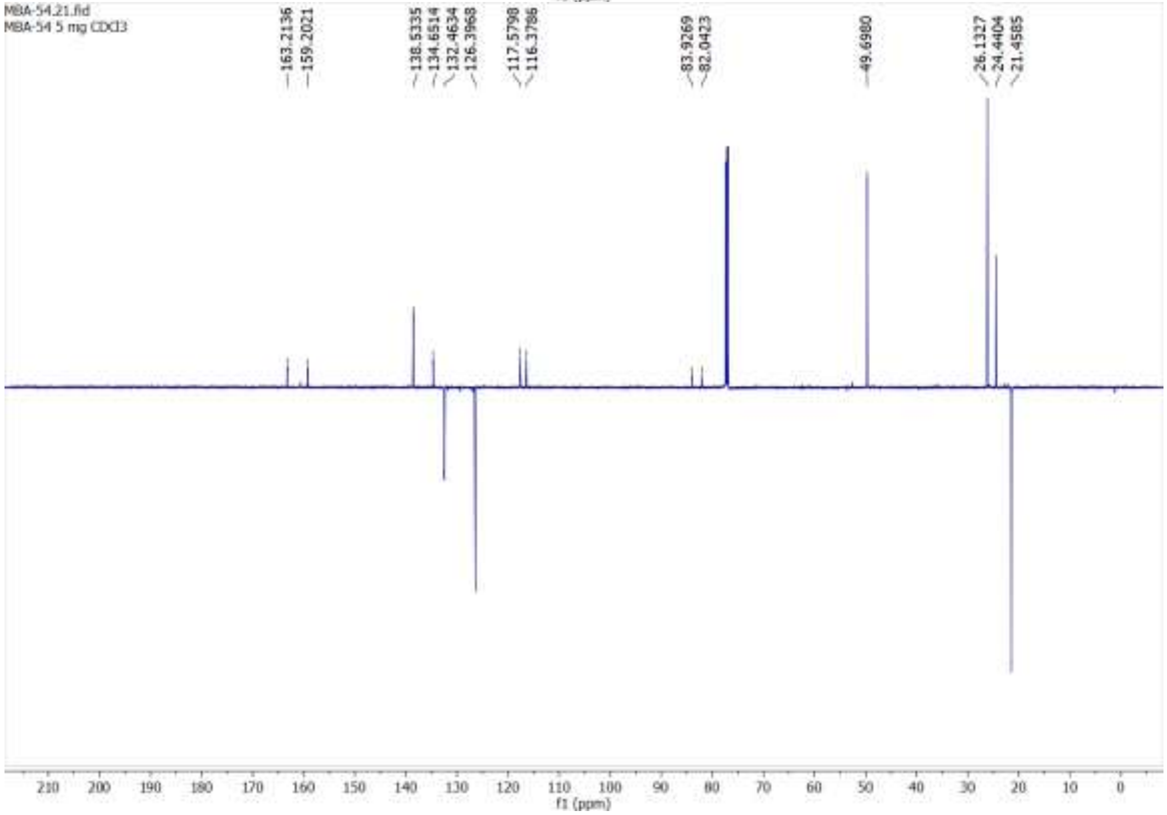
MBA-54.21.fid
MBA-54 5 mg CDCl3
163.2136
159.2021
138.5335
134.6514
132.4634
126.3968
117.5798
116.3786
83.9269
82.0423
49.6980
26.1327
24.4404
21.4585
f1 (ppm)

Compound 34:

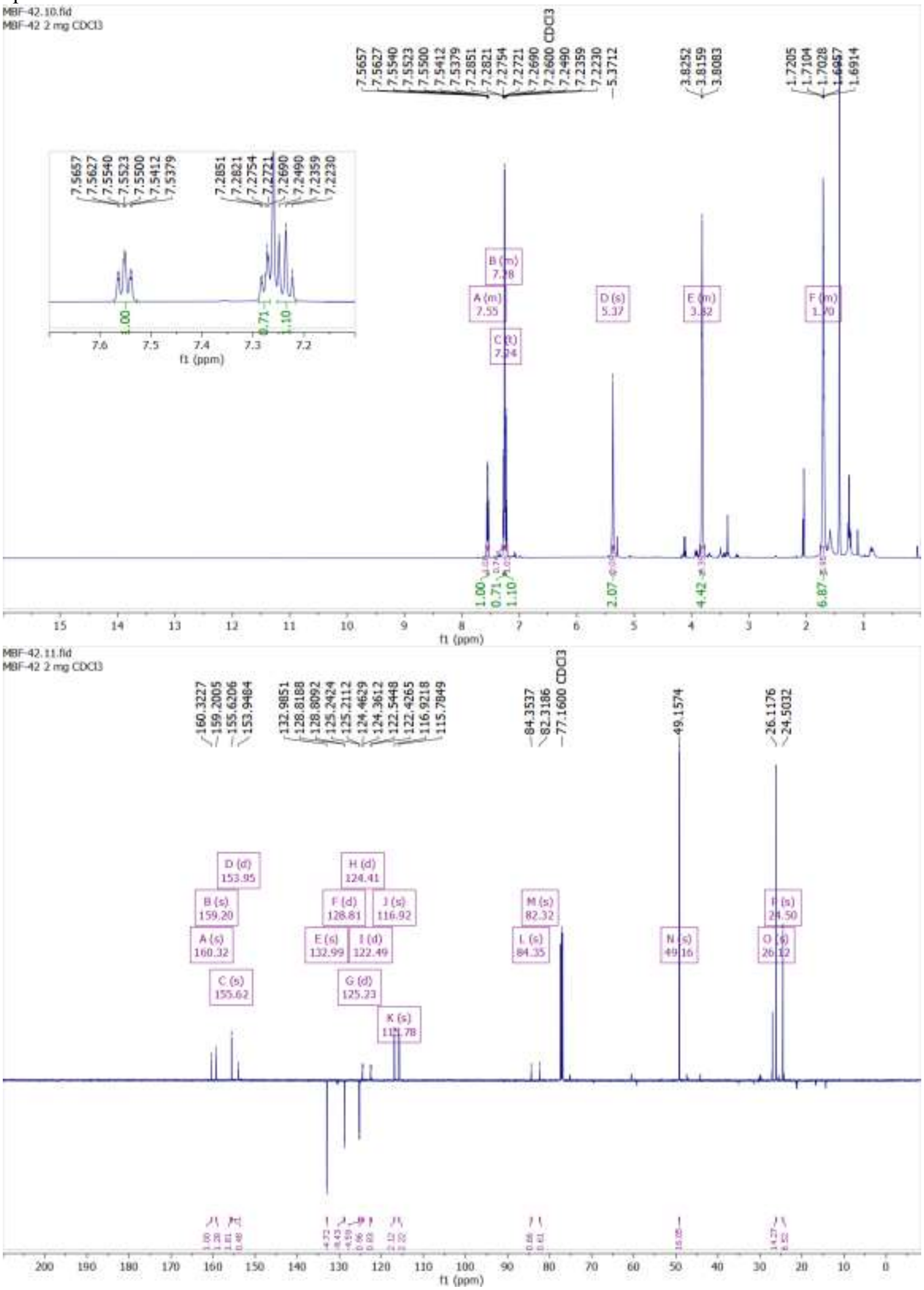

MBF-42.10.fid
MBF-42 2 mg CDCl3
7.5657
7.5627
7.5540
7.5523
7.5500
7.5412
7.5379
7.2851
7.2821
7.2754
7.2721
7.2690
7.2600 CDCl3
7.2490
7.2359
7.2230
5.3712
3.8252
3.8159
3.8083
1.7205
1.7104
1.7028
1.6957
1.6914
A (m) 7.55
B (m) 7.28
C (t) 7.24
D (s) 5.37
E (m) 3.82
F (m) 1.70
1.00
0.71
1.10
2.07
4.42
6.87
f1 (ppm)
MBF-42.11.fid
MBF-42 2 mg CDCl3
160.3227
159.2005
155.6206
153.9484
132.9851
128.8188
128.8092
125.2424
125.2112
124.4629
124.3612
122.5448
122.4265
116.9218
115.7849
84.3537
82.3186
77.1600 CDCl3
49.1574
26.1176
24.5032
A (s) 160.32
B (s) 159.20
C (s) 155.62
D (d) 153.95
E (s) 132.99
F (d) 128.81
G (d) 125.23
H (d) 124.41
I (d) 122.49
J (s) 116.92
K (s) 115.78
L (s) 84.35
M (s) 82.32
N (s) 49.16
O (s) 26.12
P (s) 24.50
f1 (ppm)

Compound 35:

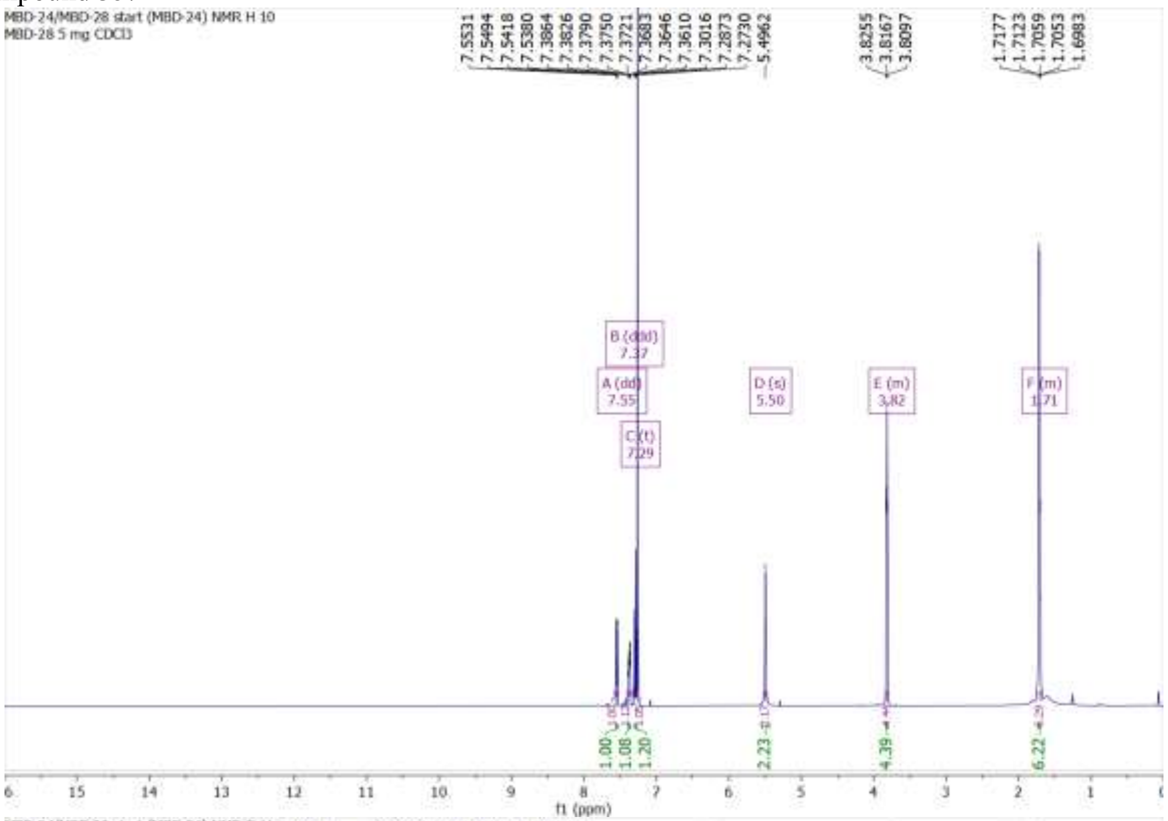
MBD-24/MBD-28 start (MBD-24) NMR H 10
MBD-28 5 mg CDCl3
7.5531
7.5494
7.5418
7.5380
7.3864
7.3826
7.3790
7.3750
7.3721
7.3683
7.3646
7.3610
7.3016
7.2873
7.2730
5.4962
3.8255
3.8167
3.8097
1.7177
1.7123
1.7059
1.7053
1.6983
B (ddd) 7.37
A (dd) 7.55
C (t) 7.29
D (s) 5.50
E (m) 3.82
F (m) 1.71
1.00
1.08
1.20
2.23
4.39
6.22
f1 (ppm)

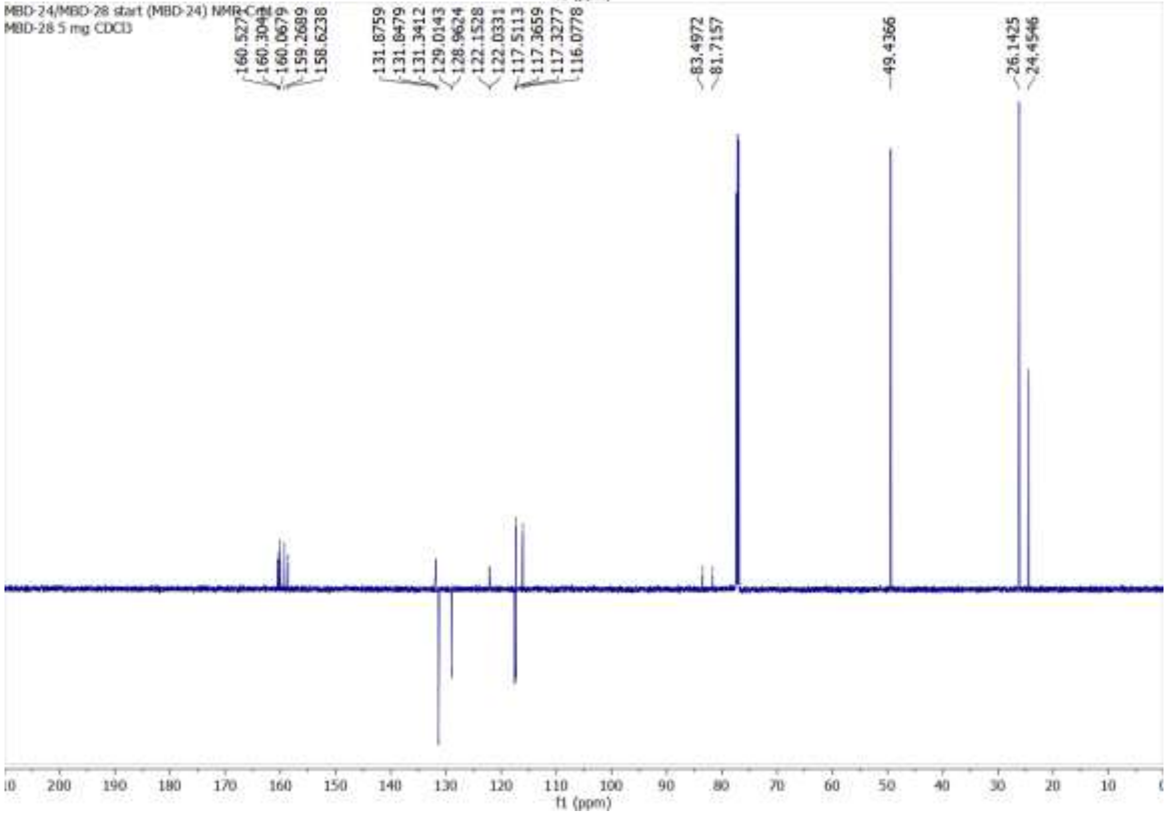
MBD-24/MBD-28 start (MBD-24) NMR C
MBD-28 5 mg CDCl3
160.3042
160.0679
159.2689
158.6238
131.8759
131.8479
131.3412
129.0143
128.9624
122.1528
122.0331
117.5113
117.3659
117.3277
116.0778
83.4972
81.7157
49.4366
26.1425
24.4546
f1 (ppm)

Compound 36:

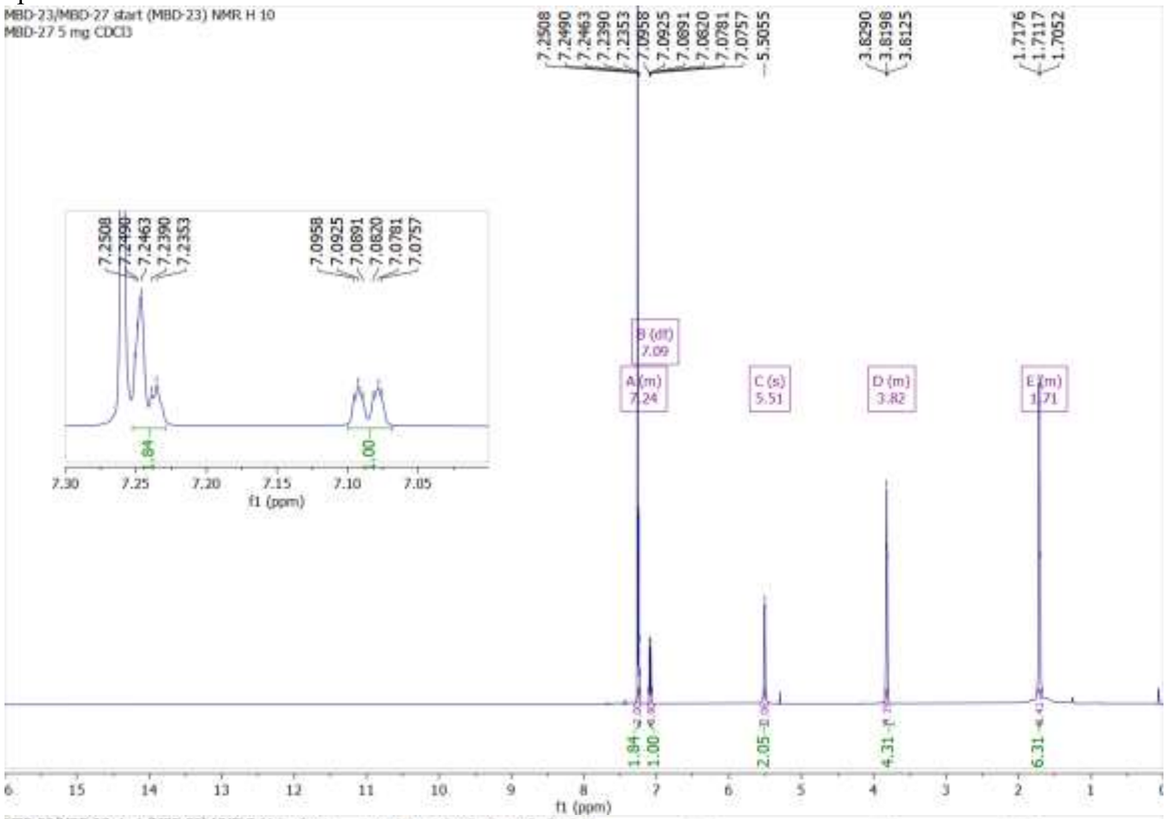
MBD-23/MBD-27 start (MBD-23) NMR H 10
MBD-27 5 mg CDCl3
7.2508
7.2490
7.2463
7.2390
7.2353
7.0958
7.0925
7.0891
7.0820
7.0781
7.0757
5.5055
3.8290
3.8198
3.8125
1.7176
1.7117
1.7052
B (dt) 7.09
A (m) 7.24
C (s) 5.51
D (m) 3.82
E (m) 1.71
1.84
1.00
2.05
4.31
6.31
f1 (ppm)

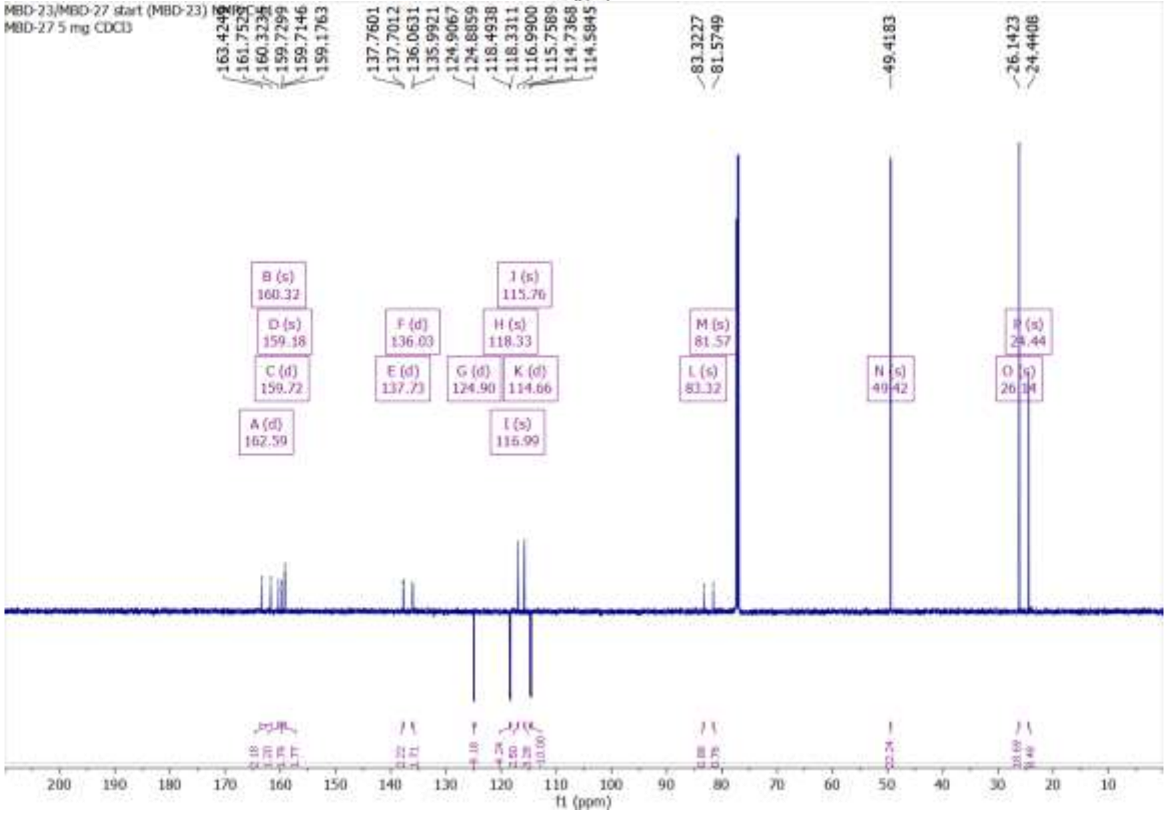
MBD-23/MBD-27 start (MBD-23) NMR C
MBD-27 5 mg CDCl3
163.4249
161.7528
160.3236
159.7299
159.7146
159.1763
137.7601
137.7012
136.0631
135.9921
124.9067
124.8859
118.4938
118.3311
116.9900
115.7589
114.7368
114.5845
83.3227
81.5749
49.4183
26.1423
24.4408
B (s) 160.32
D (s) 159.18
C (d) 159.72
A (d) 162.59
F (d) 136.03
E (d) 137.73
G (d) 124.90
J (s) 115.76
H (s) 118.33
K (d) 114.66
I (s) 116.99
M (s) 81.57
L (s) 83.32
N (s) 49.42
P (s) 24.44
O (s) 26.14
f1 (ppm)

Compound 37:

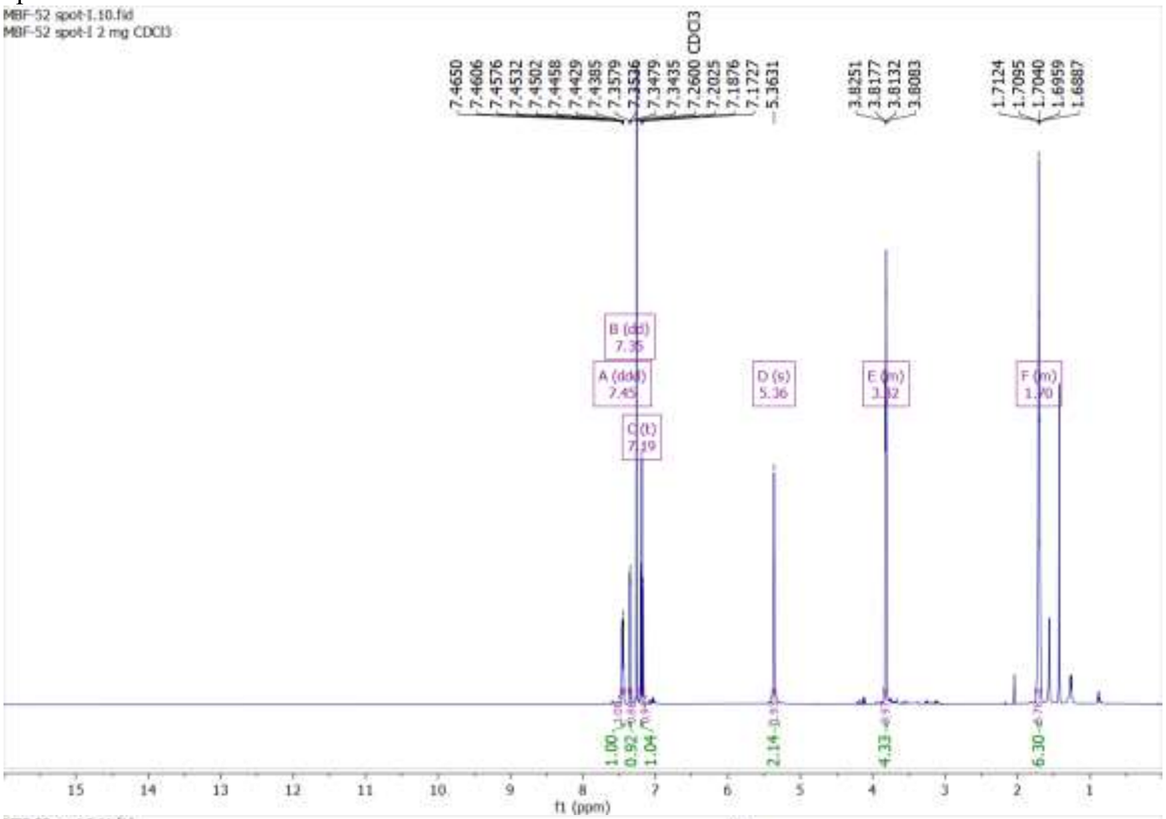
MBF-52 spot-I.10.fid
MBF-52 spot-I 2 mg CDCl3
CDCl3
f1 (ppm)

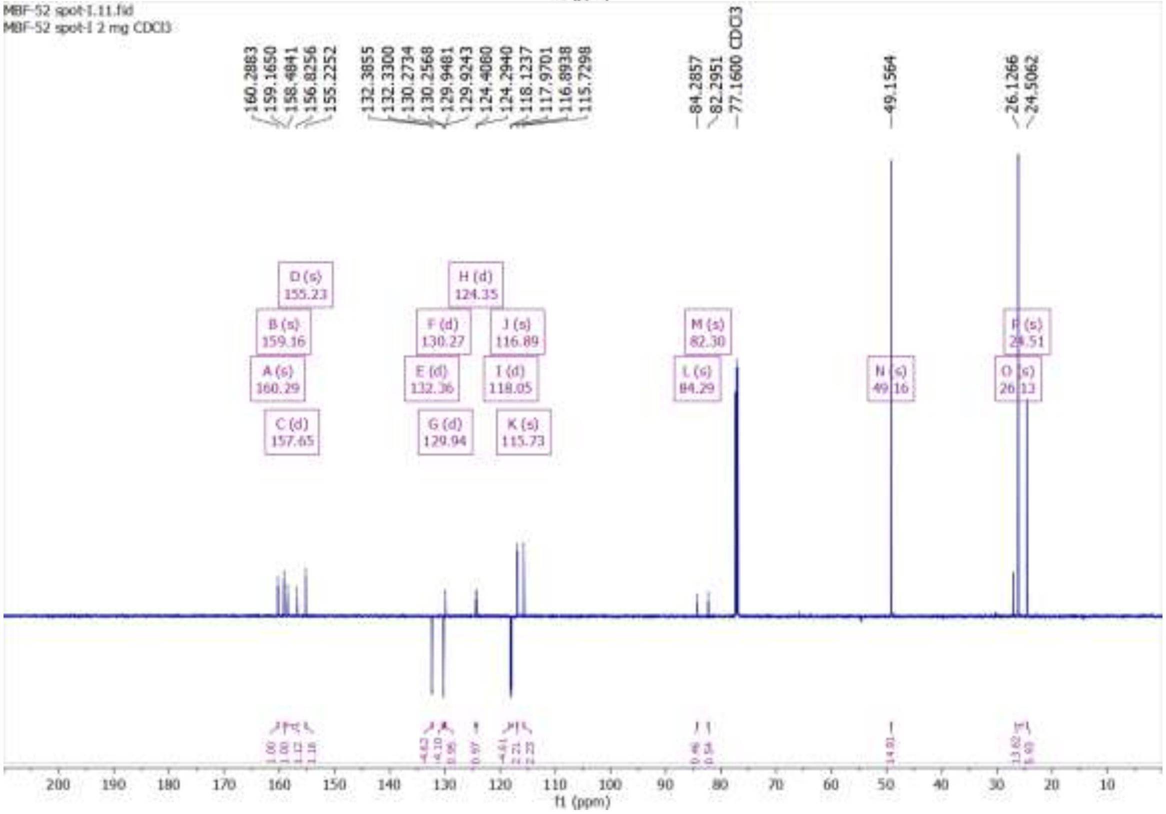
MBF-52 spot-I.11.fid
MBF-52 spot-I 2 mg CDCl3
CDCl3
f1 (ppm)

Compound 38:

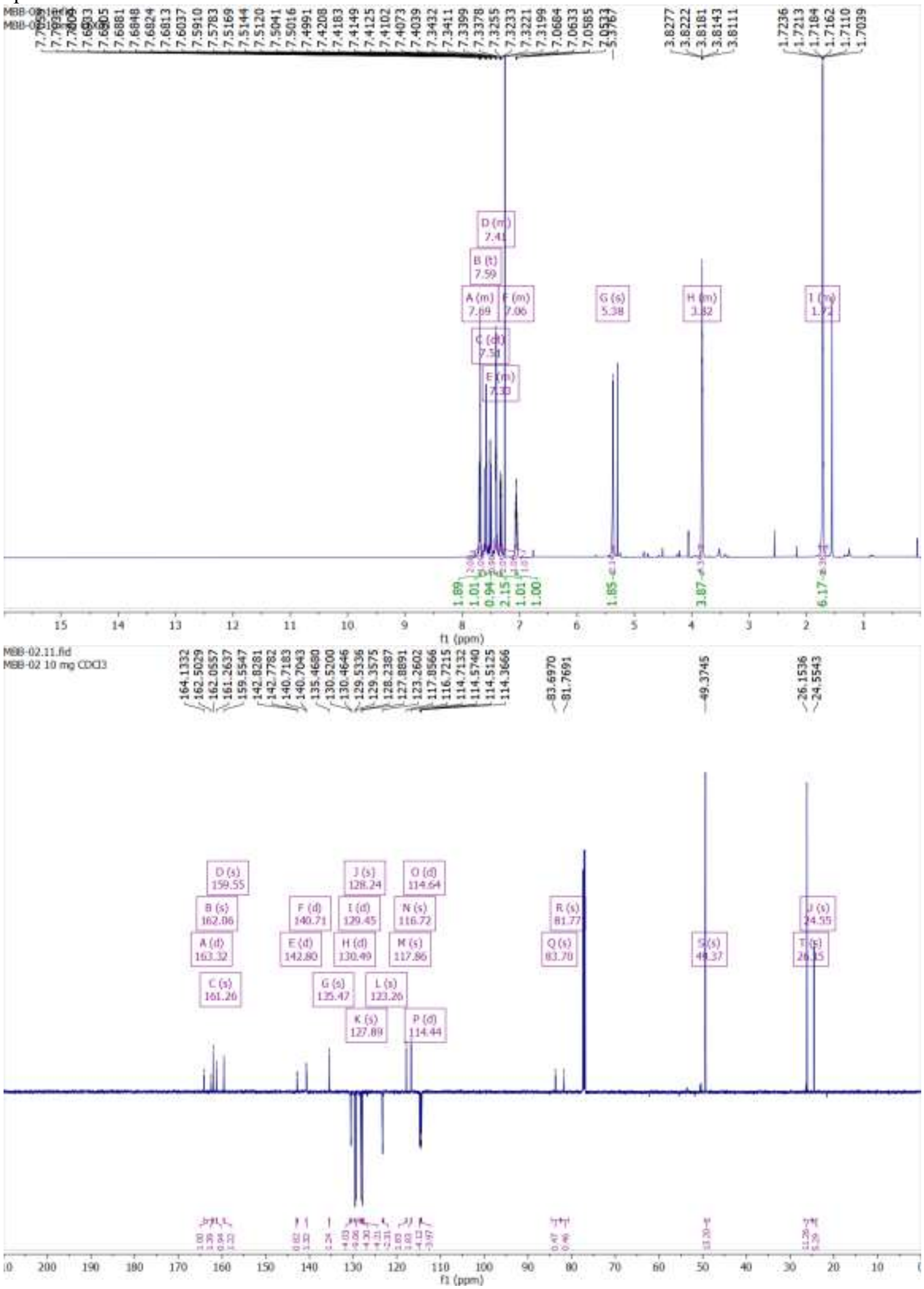

Compound 39:

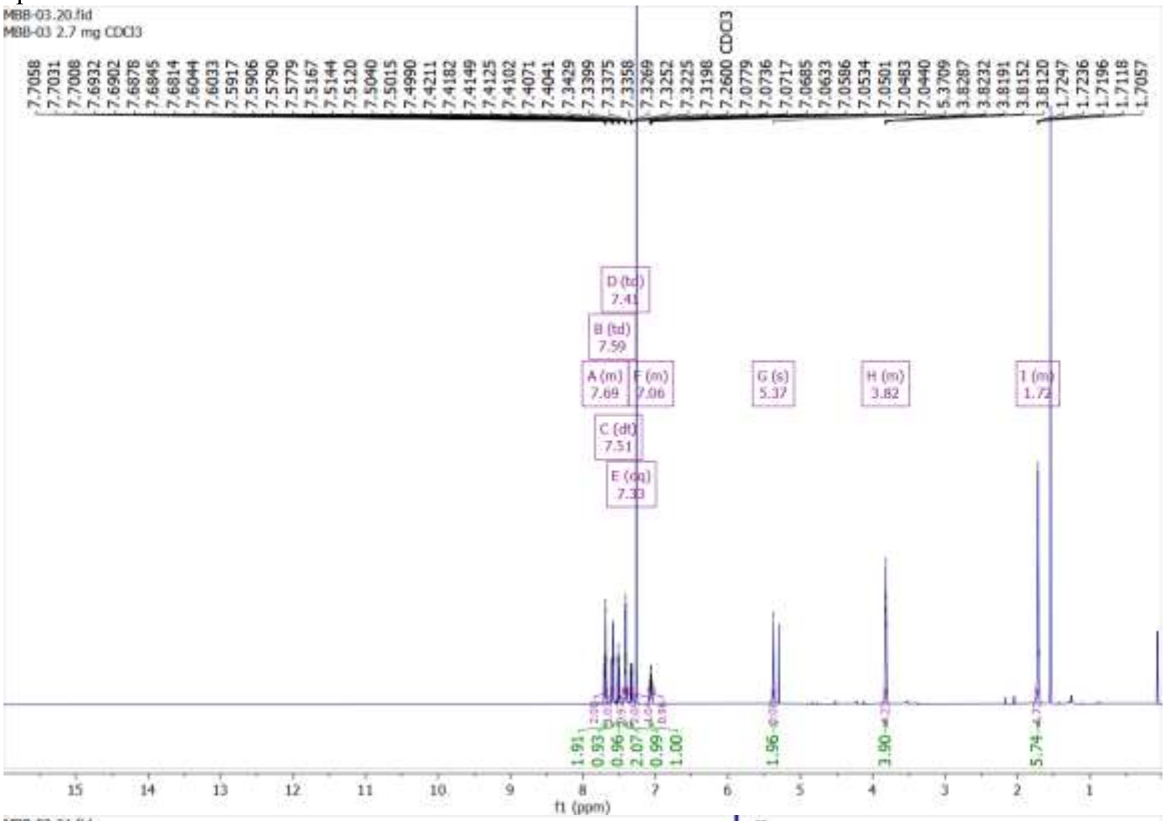
MBB-03.20.fid
MBB-03 2.7 mg CDCl3
f1 (ppm)

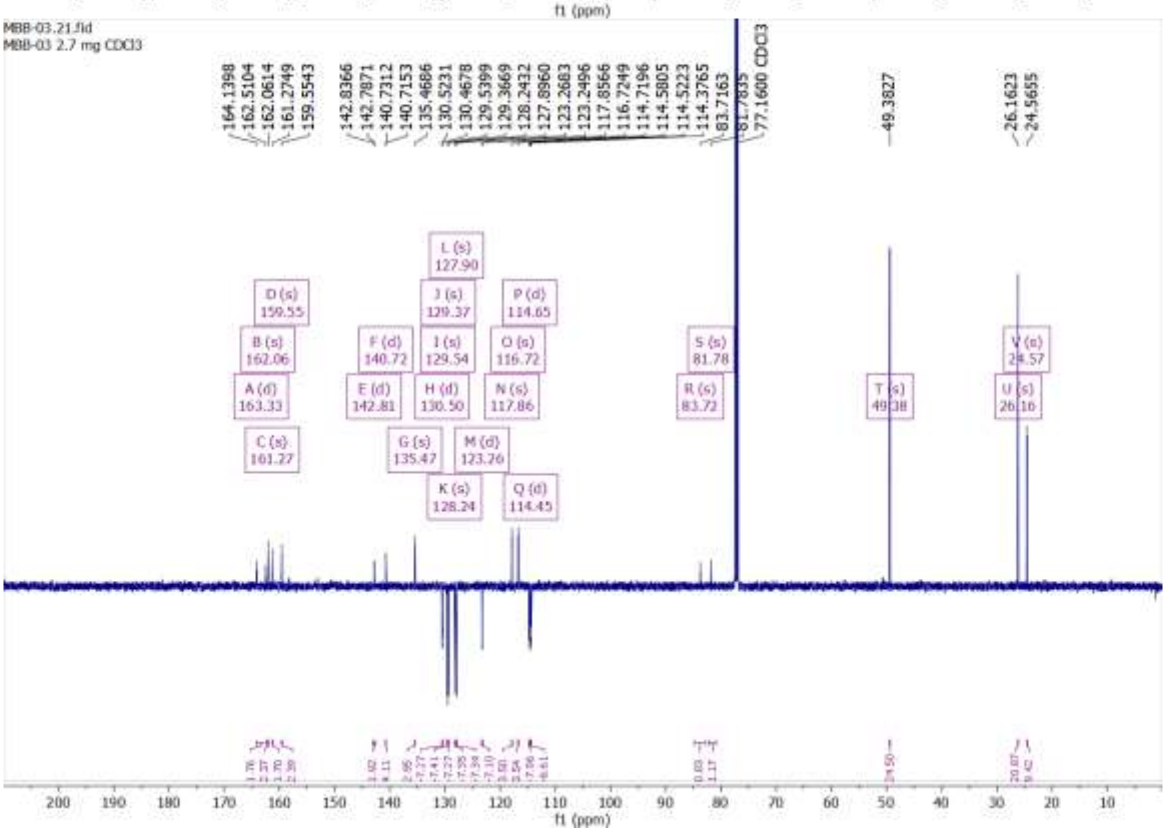
MBB-03.21.fid
MBB-03 2.7 mg CDCl3
f1 (ppm)

Compound 40:

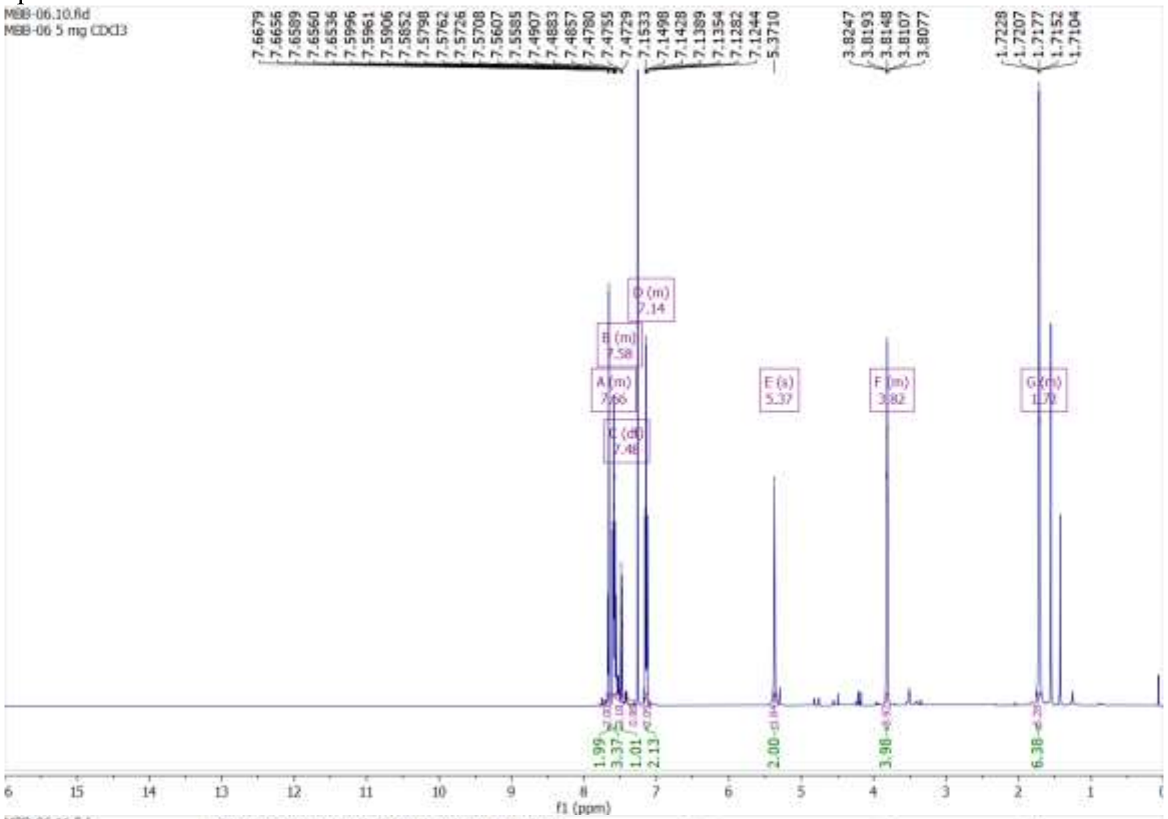

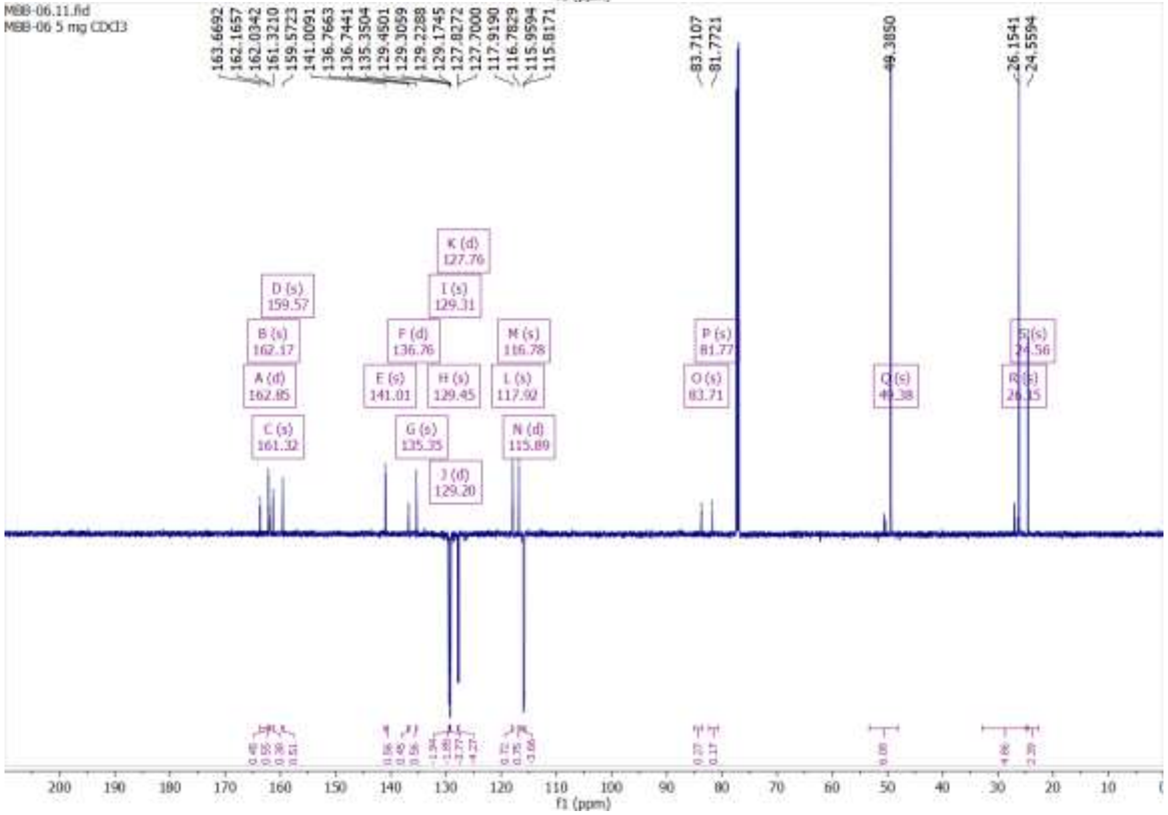

Compound 41:

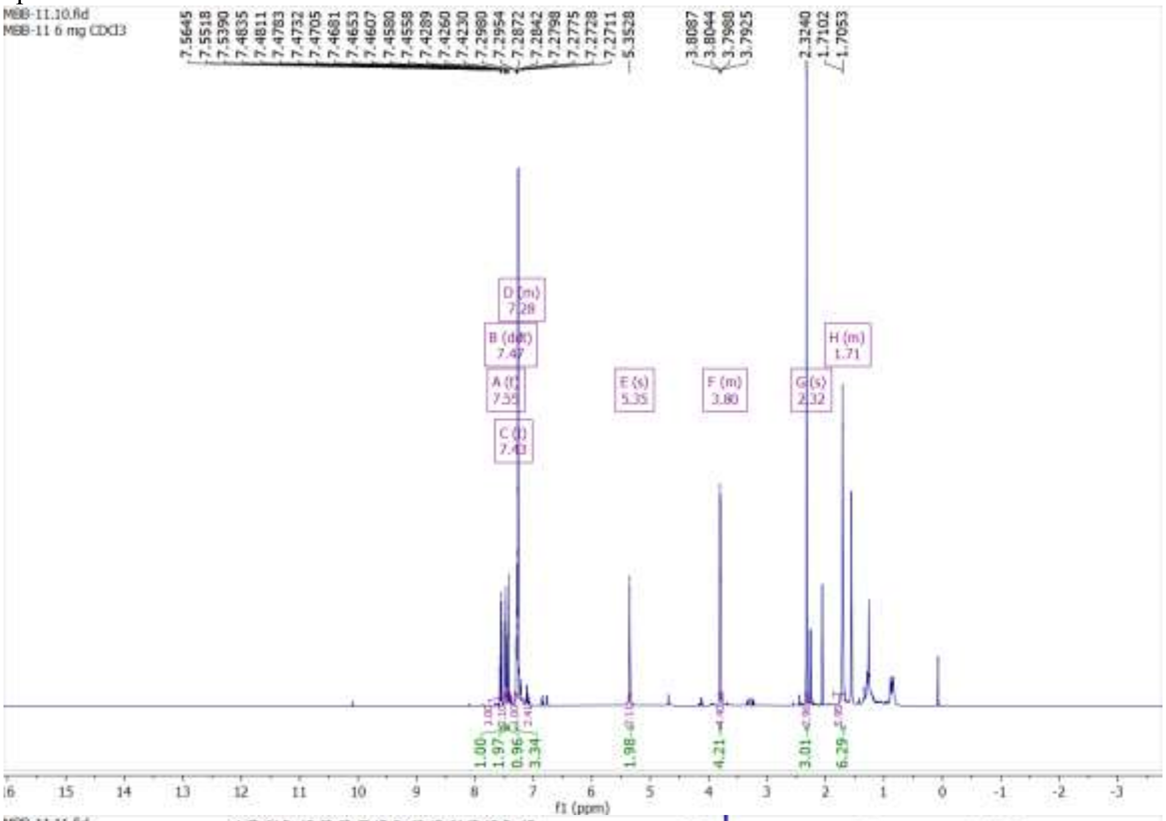
MBB-11.10.fid
MBB-11 6 mg CDCl3
7.5645
7.5518
7.5390
7.4835
7.4811
7.4783
7.4732
7.4705
7.4681
7.4653
7.4607
7.4580
7.4558
7.4289
7.4260
7.4230
7.2980
7.2954
7.2872
7.2842
7.2798
7.2775
7.2728
7.2711
5.3528
3.8087
3.8044
3.7988
3.7925
2.3240
1.7102
1.7053
D (m)
7.28
B (ddt)
7.47
A (t)
7.55
C (t)
7.43
E (s)
5.35
F (m)
3.80
G (s)
2.32
H (m)
1.71
1.00
1.97
0.96
3.34
1.98
4.21
3.01
6.29
f1 (ppm)

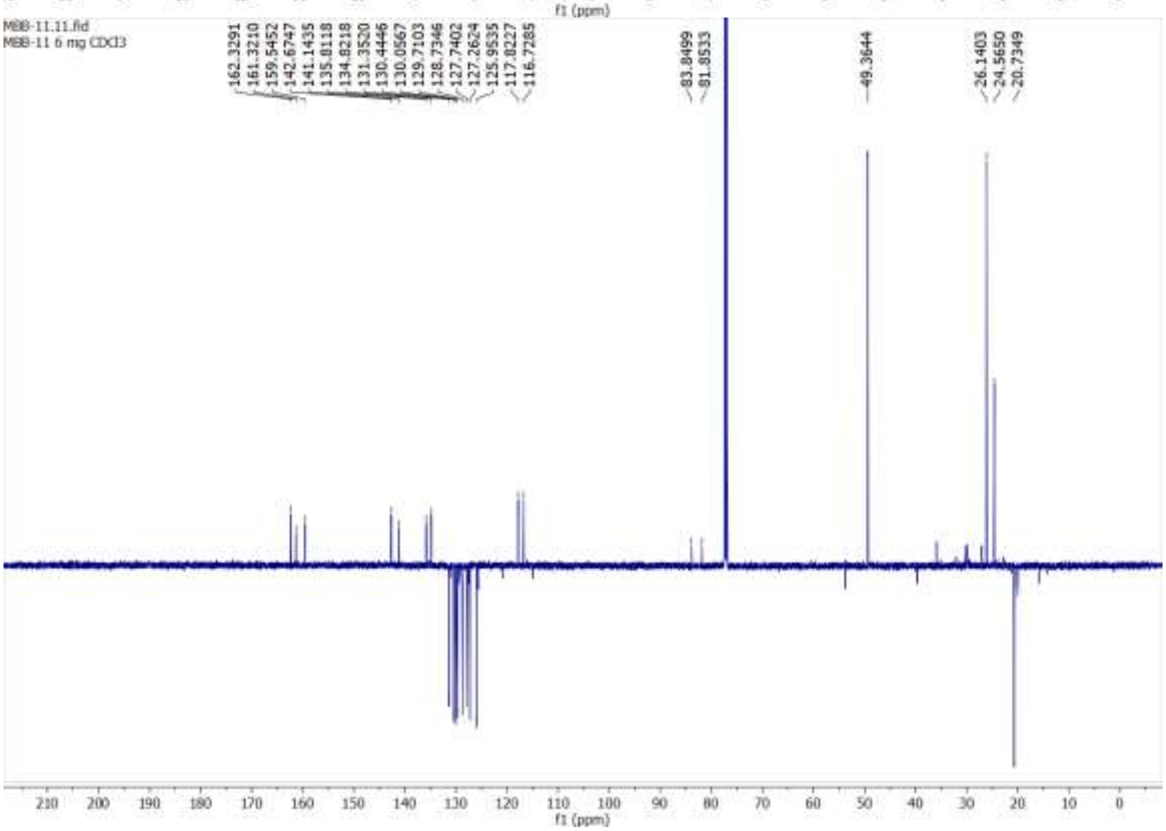
MBB-11.11.fid
MBB-11 6 mg CDCl3
162.3291
161.3210
159.5452
142.6747
141.1435
135.8118
134.8218
131.3520
130.4446
130.0567
129.7103
128.7346
127.7402
127.2624
125.9535
117.8227
116.7285
83.8499
81.8533
49.3644
26.1403
24.5650
20.7349
f1 (ppm)

Compound 42:

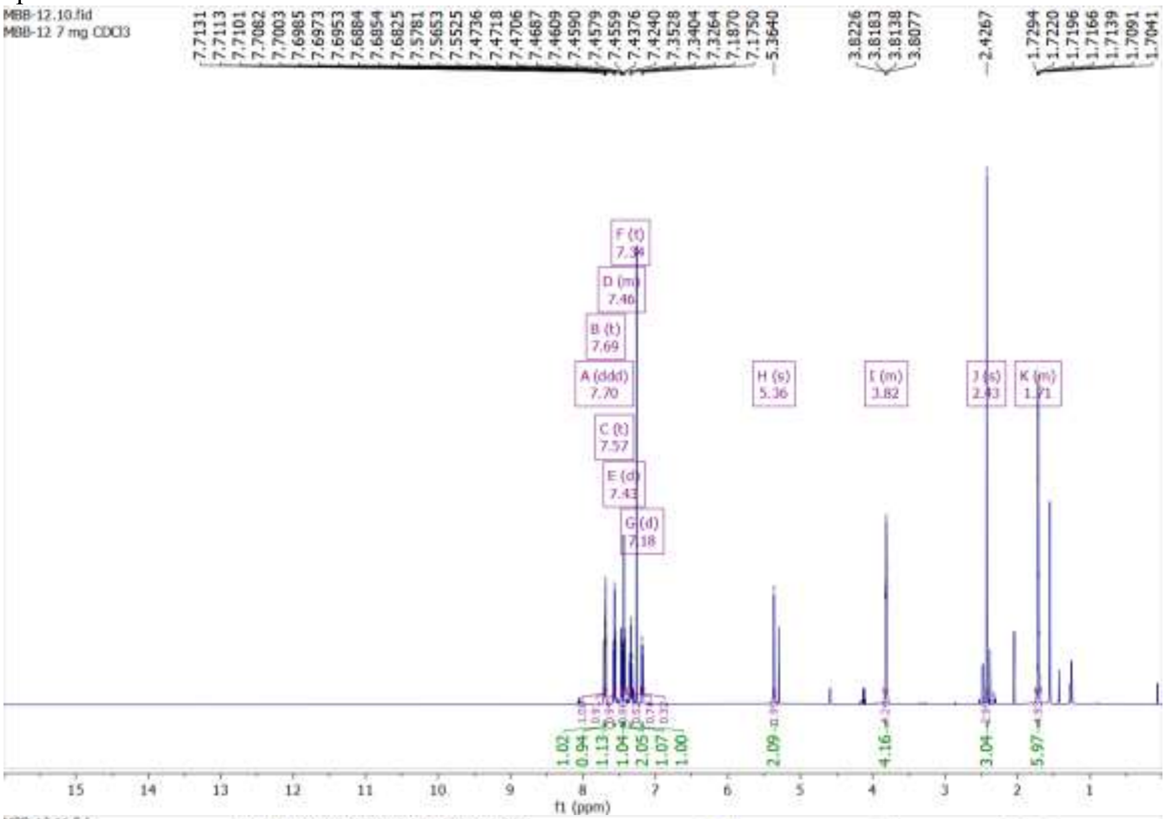
MBB-12.10.fid
MBB-12 7 mg CDCl3
7.7131
7.7113
7.7101
7.7082
7.7003
7.6985
7.6973
7.6953
7.6884
7.6854
7.6825
7.5781
7.5653
7.5525
7.4736
7.4718
7.4706
7.4687
7.4609
7.4590
7.4579
7.4559
7.4376
7.4240
7.3528
7.3404
7.3264
7.1870
7.1750
5.3640
3.8226
3.8183
3.8138
3.8077
2.4267
1.7294
1.7220
1.7196
1.7166
1.7139
1.7091
1.7041
F (t) 7.34
D (m) 7.46
B (t) 7.69
A (ddd) 7.70
C (t) 7.57
E (d) 7.43
G (d) 7.18
H (s) 5.36
I (m) 3.82
J (s) 2.43
K (m) 1.71
1.02
0.94
1.13
1.04
2.05
1.07
1.00
2.09
4.16
3.04
5.97
f1 (ppm)

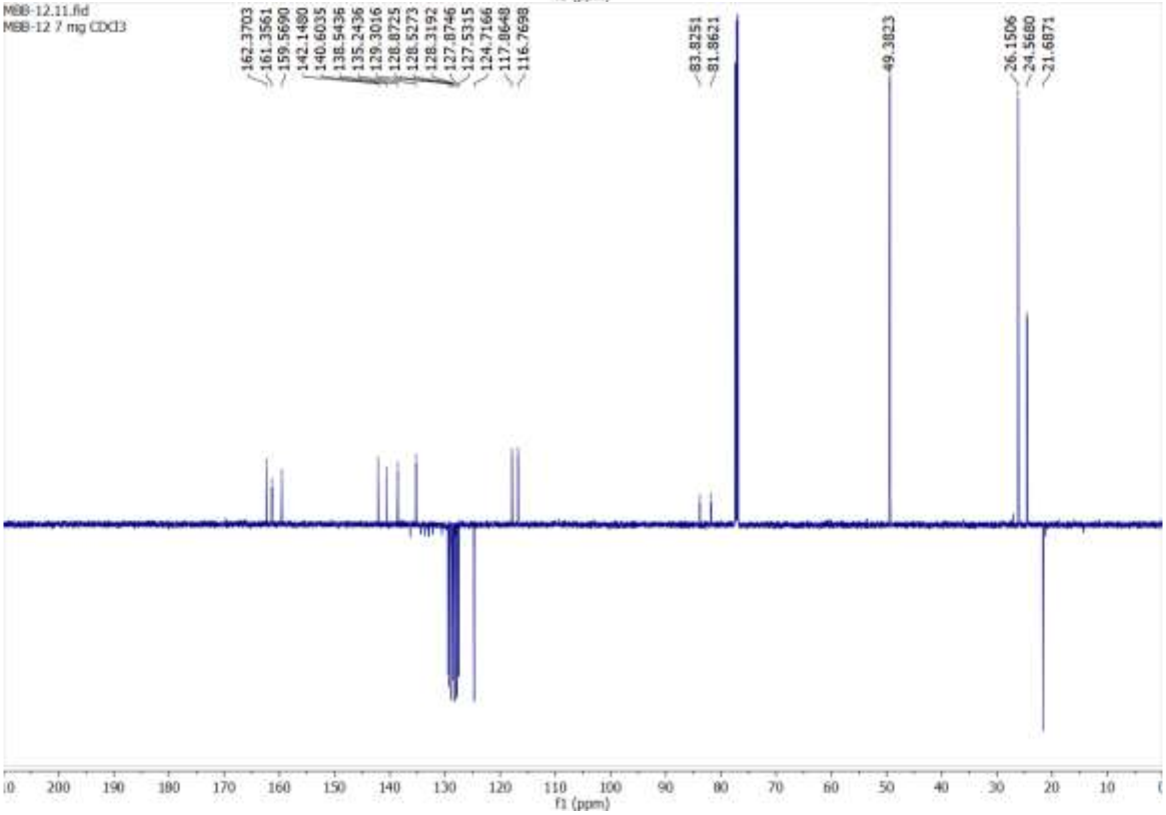
MBB-12.11.fid
MBB-12 7 mg CDCl3
162.3703
161.3561
159.5690
142.1480
140.6035
138.5436
135.2436
129.3016
128.8725
128.5273
128.3192
127.8746
127.5315
124.7166
117.8648
116.7698
83.8251
81.8621
49.3823
26.1506
24.5680
21.6871
f1 (ppm)

Compound 43:

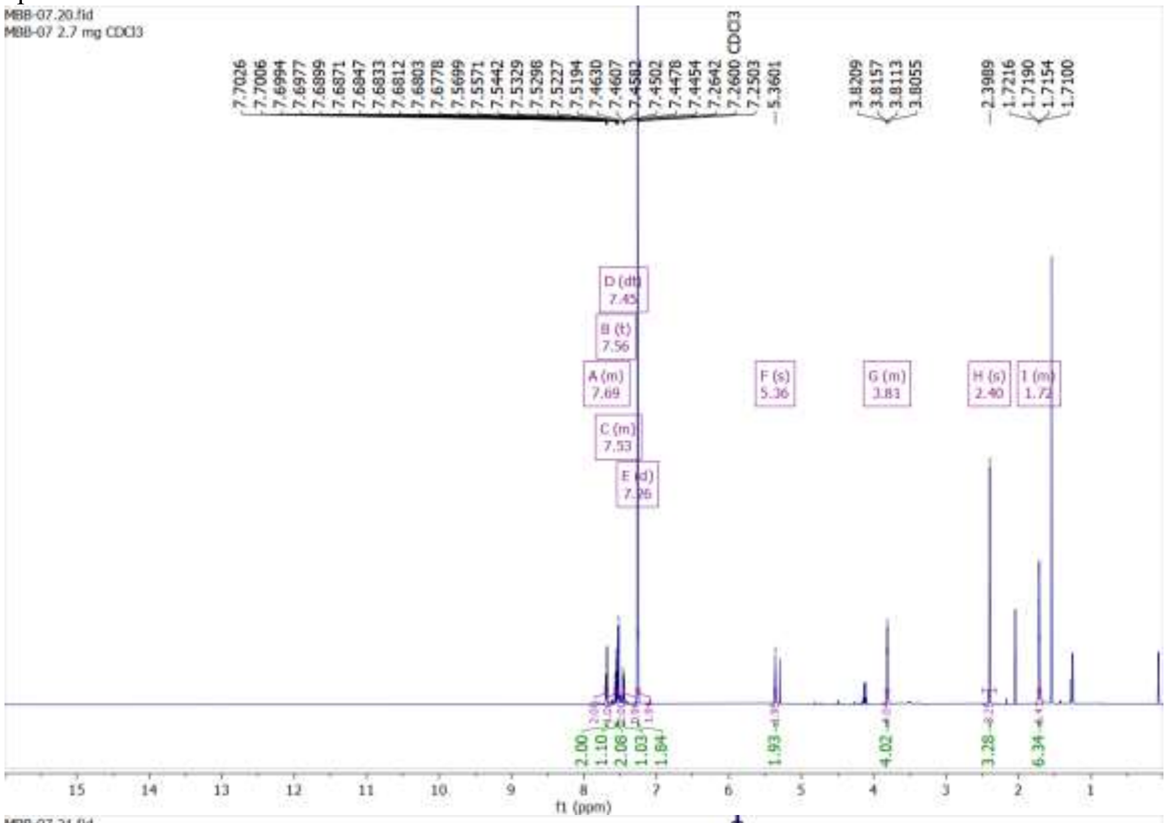
MBB-07.20.fid
MBB-07 2.7 mg CDCl3
7.7026
7.7006
7.6994
7.6977
7.6899
7.6871
7.6847
7.6833
7.6812
7.6803
7.6778
7.5699
7.5571
7.5442
7.5329
7.5298
7.5227
7.5194
7.4630
7.4607
7.4582
7.4502
7.4478
7.4454
7.2642
7.2600 CDCl3
7.2503
5.3601
3.8209
3.8157
3.8113
3.8055
2.3989
1.7216
1.7190
1.7154
1.7100
D (dt) 7.45
B (t) 7.56
A (m) 7.69
C (m) 7.53
E (d) 7.26
F (s) 5.36
G (m) 3.81
H (s) 2.40
I (m) 1.72
2.00
1.10
2.08
1.03
1.84
1.93
4.02
3.28
6.34
f1 (ppm)

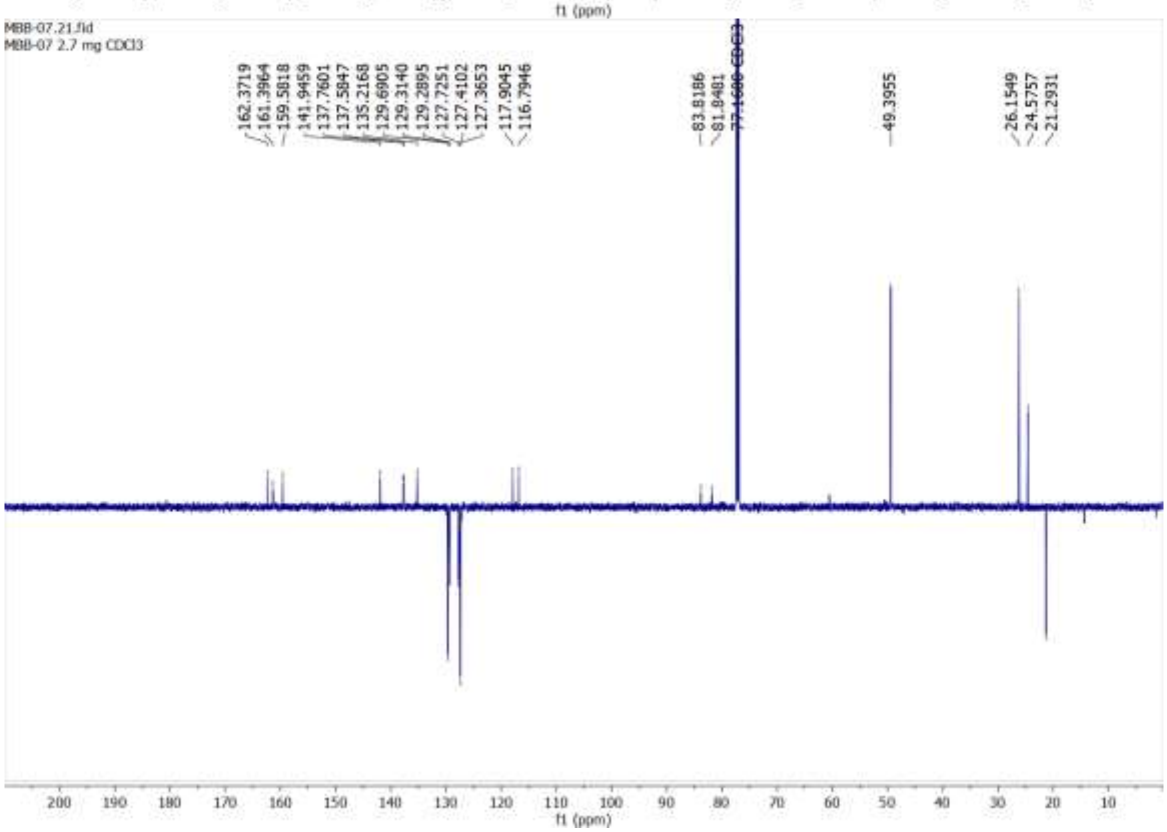
MBB-07.21.fid
MBB-07 2.7 mg CDCl3
162.3719
161.3964
159.5818
141.9459
137.7601
137.5847
135.2168
129.6905
129.3140
129.2895
127.7251
127.4102
127.3653
117.9045
116.7946
83.8186
81.8481
77.1600 CDCl3
49.3955
26.1549
24.5757
21.2931
f1 (ppm)

Compound 44:

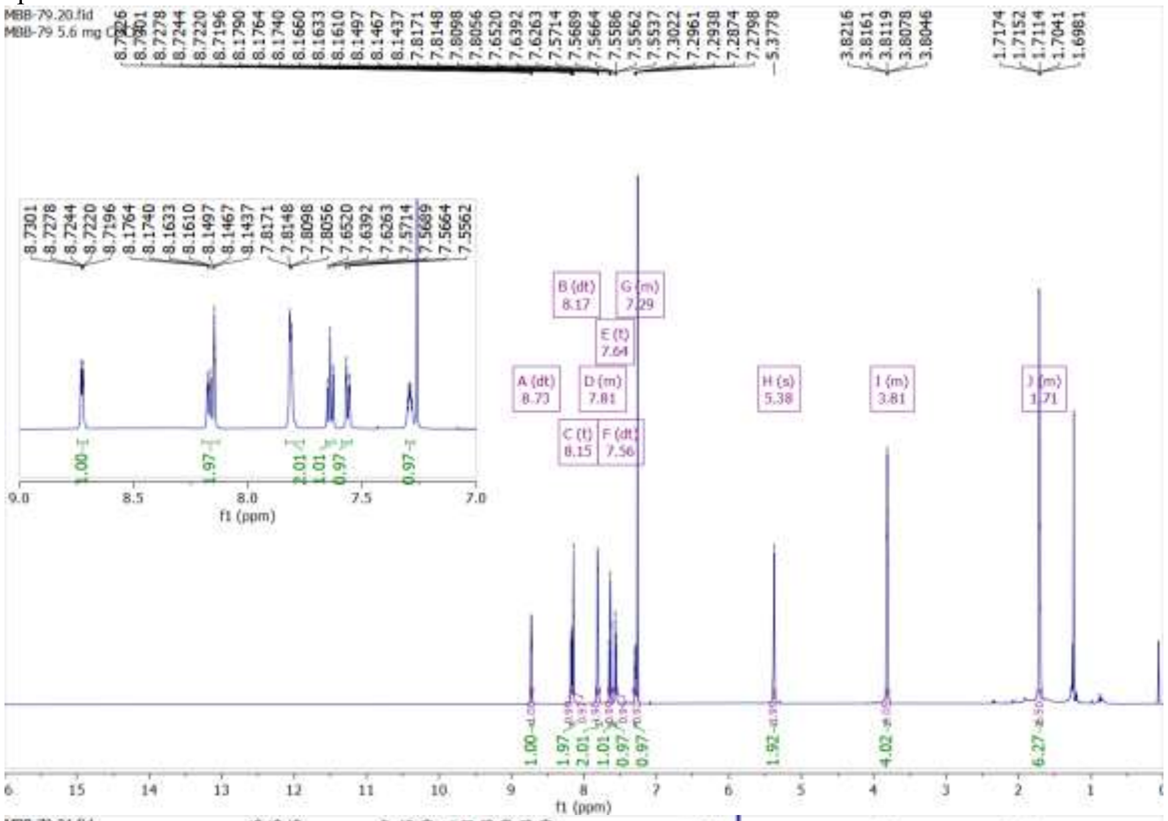

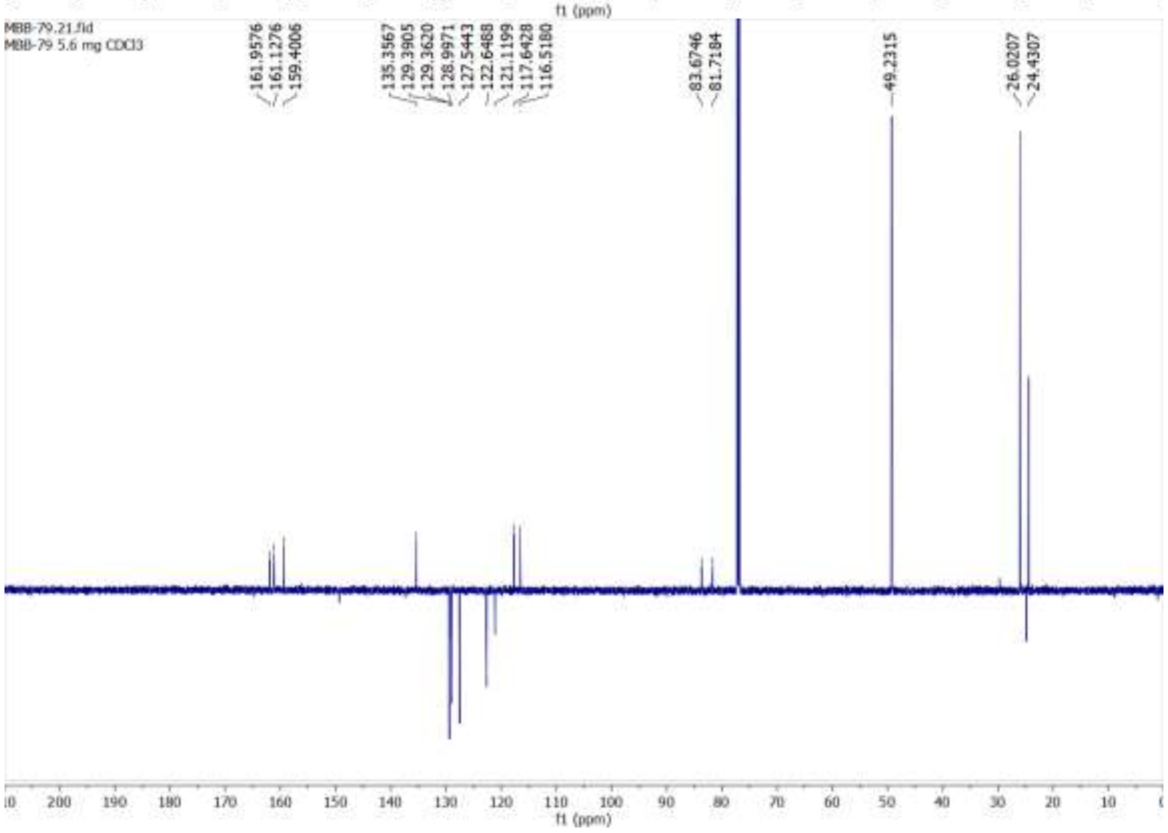

Compound 45:

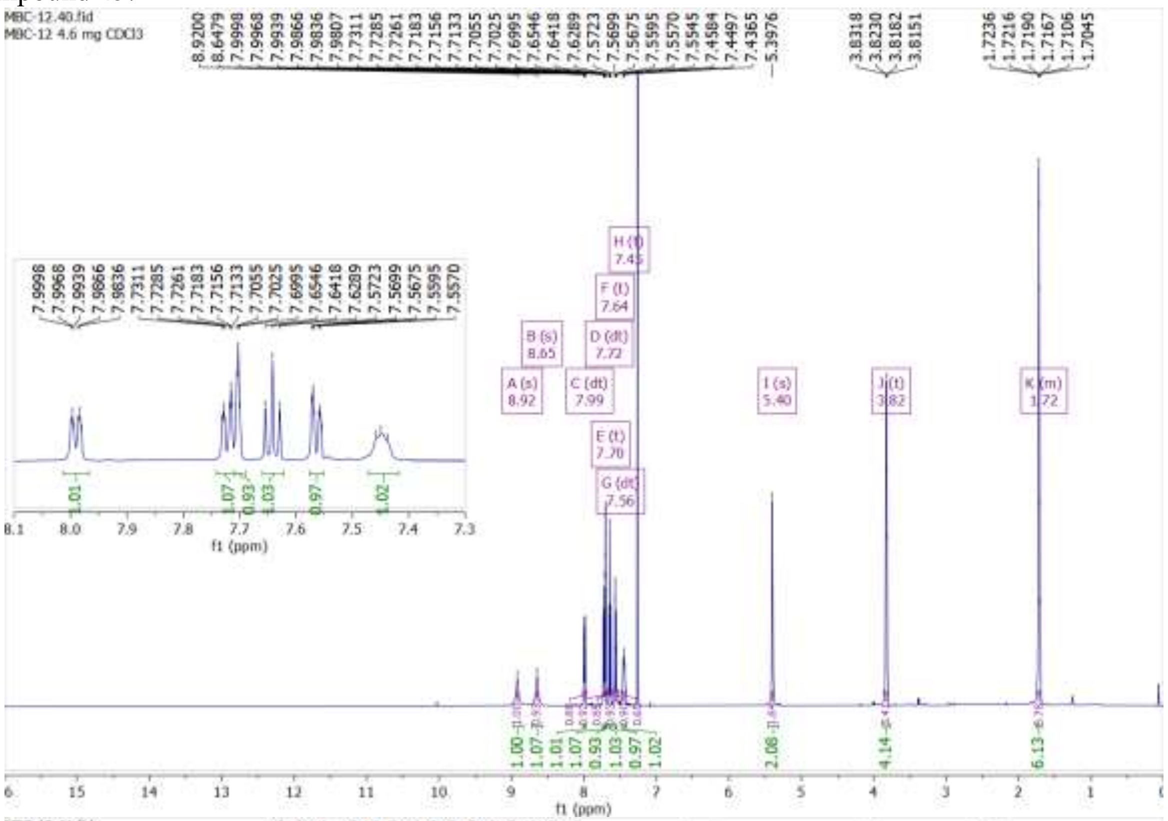

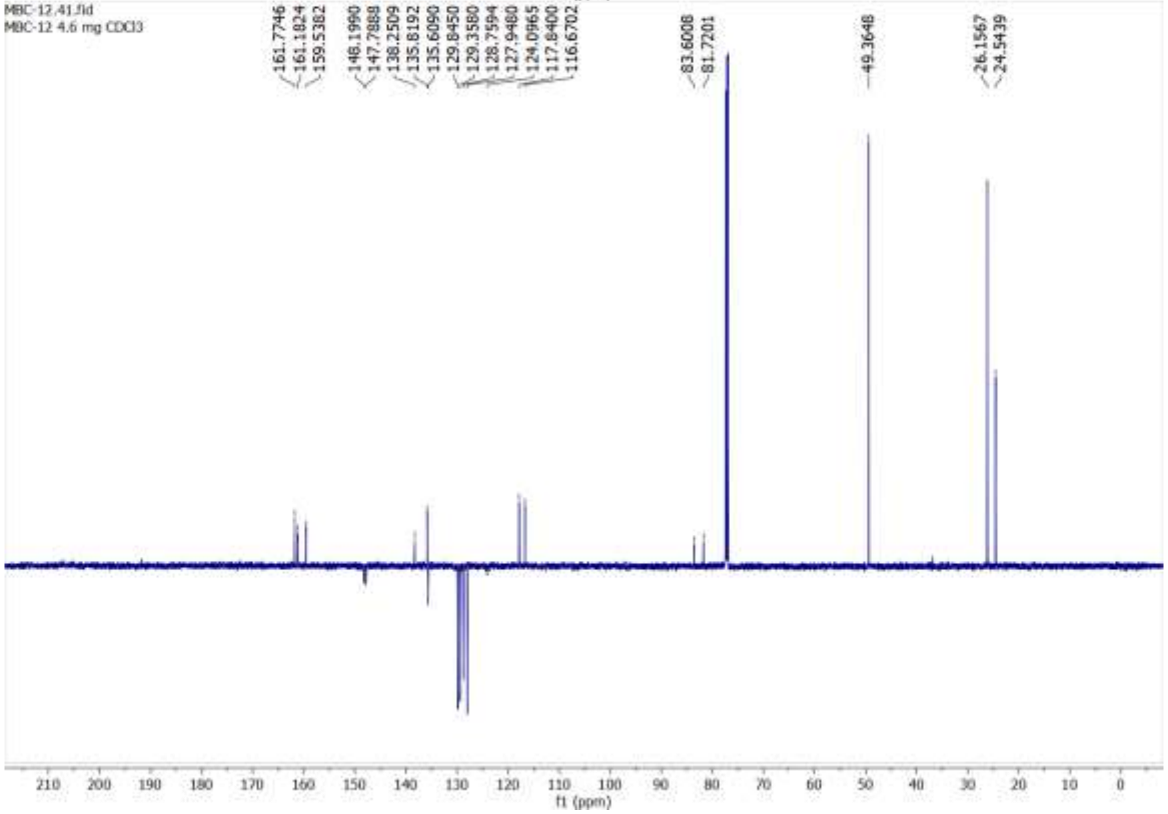

Compound 46:

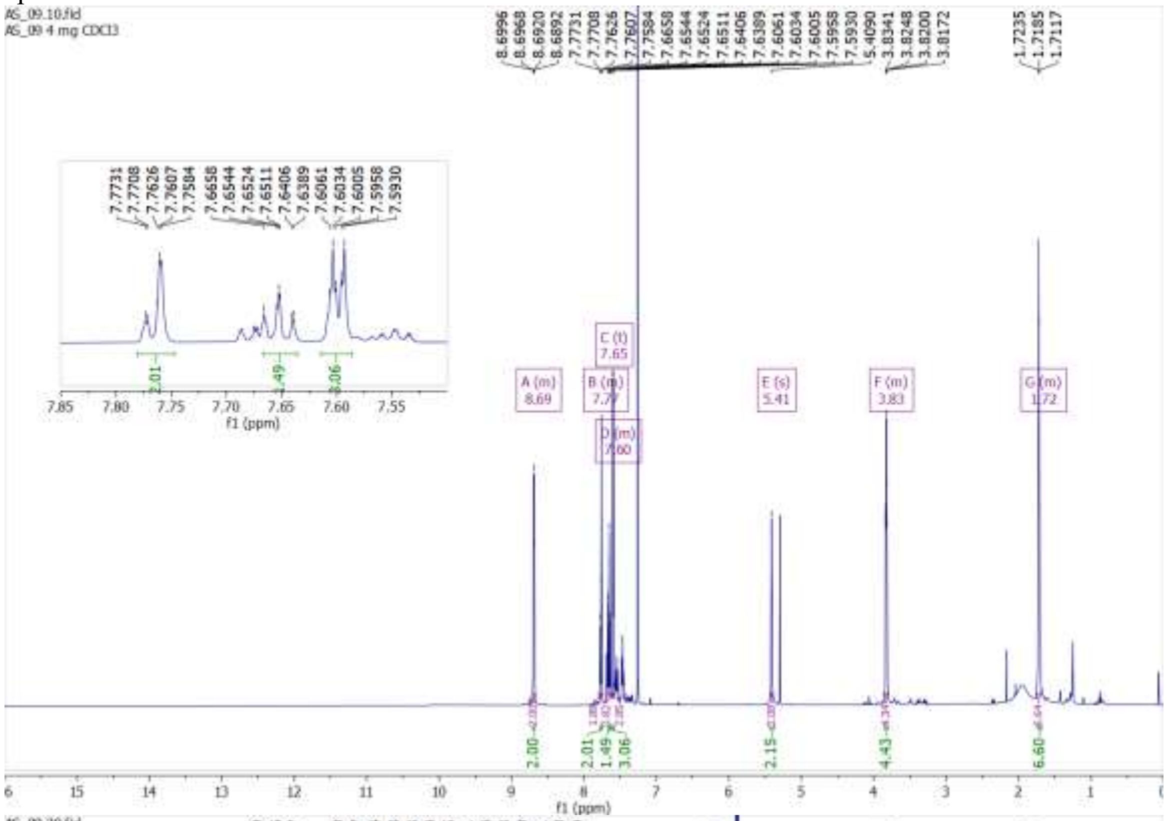

AS_09.10.fid
AS_09 4 mg CDCl3
A (m) 8.69
B (m) 7.77
C (t) 7.65
D (m) 7.60
E (s) 5.41
F (m) 3.83
G (m) 1.72
f1 (ppm)

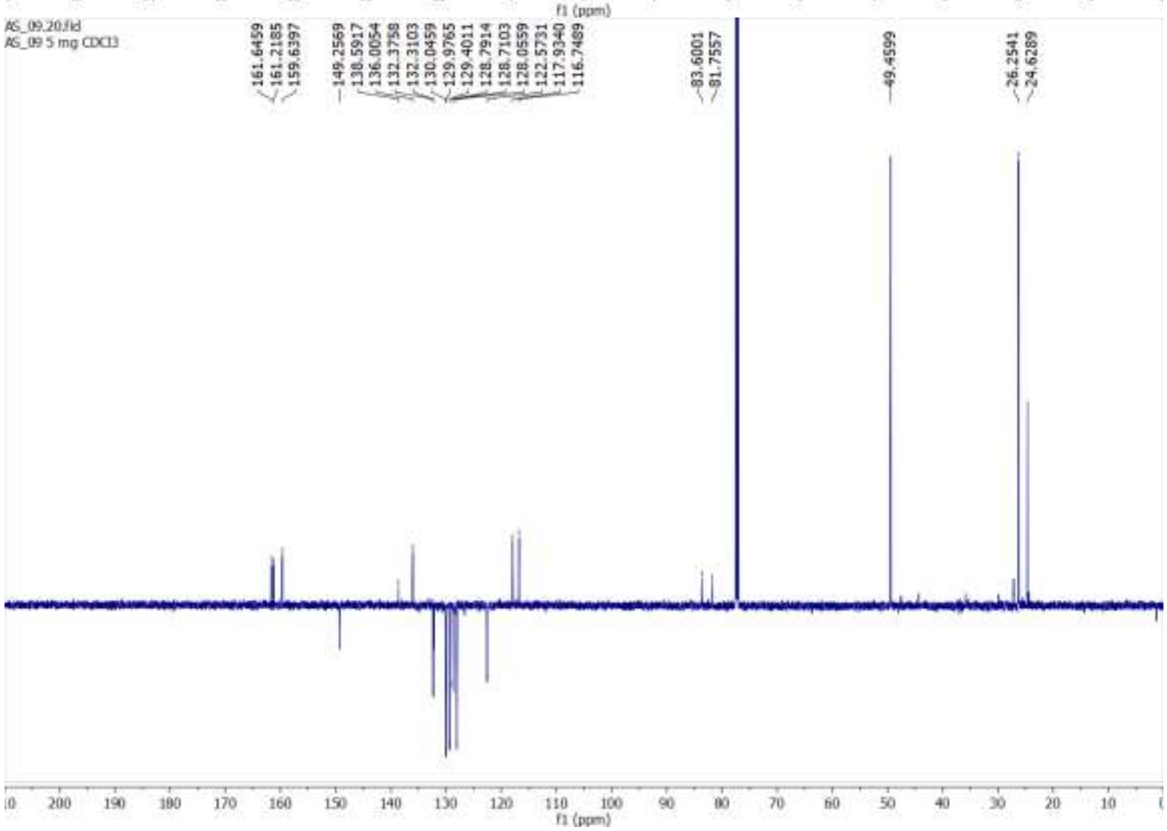

AS_09.20.fid
AS_09 5 mg CDCl3
161.6459
161.2185
159.6397
149.2569
138.5917
136.0054
132.3758
132.3103
130.0459
129.9765
129.4011
128.7914
128.7103
128.0559
122.5731
117.9340
116.7489
83.6001
81.7557
49.4599
26.2541
24.6289
f1 (ppm)

Compound 47:

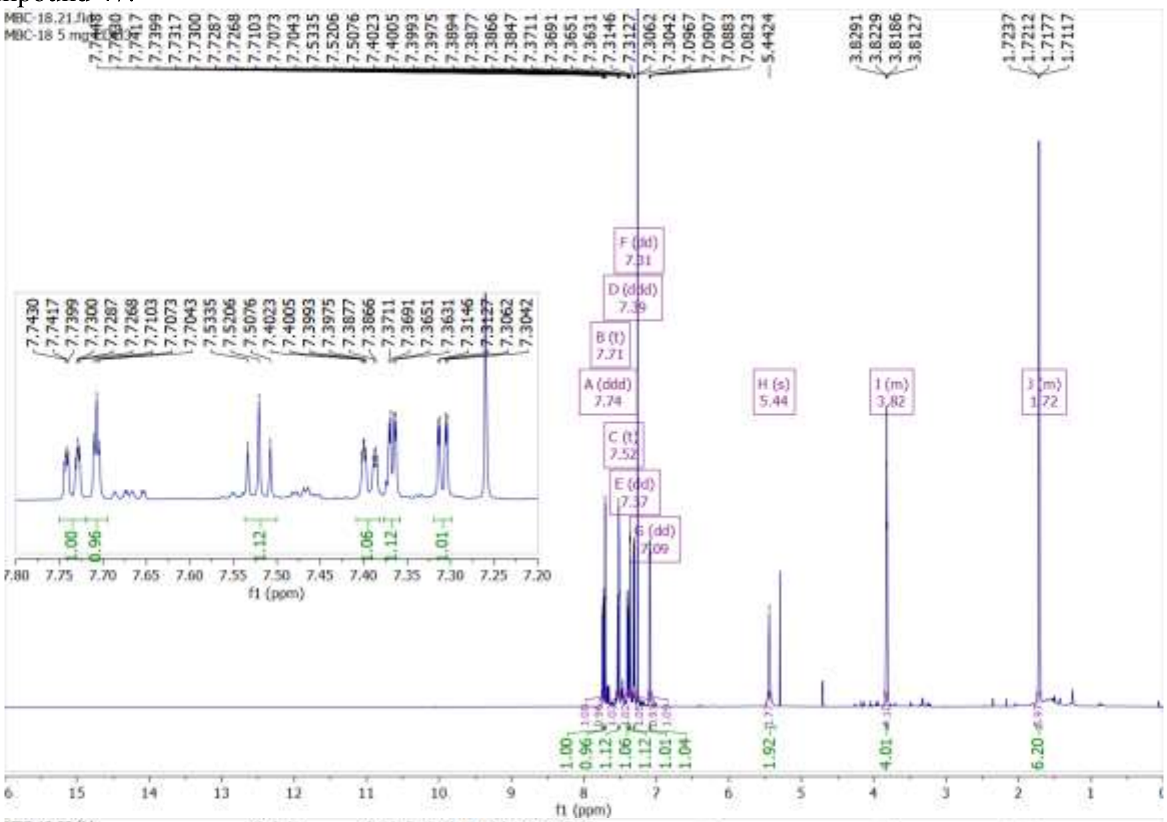
MBC-18.21.fid
MBC-18 5 mg CDCl3
7.7448 7.7430 7.7417 7.7399 7.7317 7.7300 7.7287 7.7268 7.7103 7.7073 7.7043 7.5335 7.5206 7.5076 7.4023 7.4005 7.3993 7.3975 7.3894 7.3877 7.3866 7.3847 7.3711 7.3691 7.3651 7.3631 7.3146 7.3127 7.3062 7.3042 7.0967 7.0907 7.0883 7.0823
5.4424
3.8291 3.8229 3.8186 3.8127
1.7237 1.7212 1.7177 1.7117
F (dd) 7.31
D (ddd) 7.39
B (t) 7.71
A (ddd) 7.74
C (t) 7.52
E (dd) 7.37
G (dd) 7.09
H (s) 5.44
I (m) 3.82
J (m) 1.72
1.00 0.96 1.12 1.06 1.12 1.01 1.04 1.92 4.01 6.20
f1 (ppm)

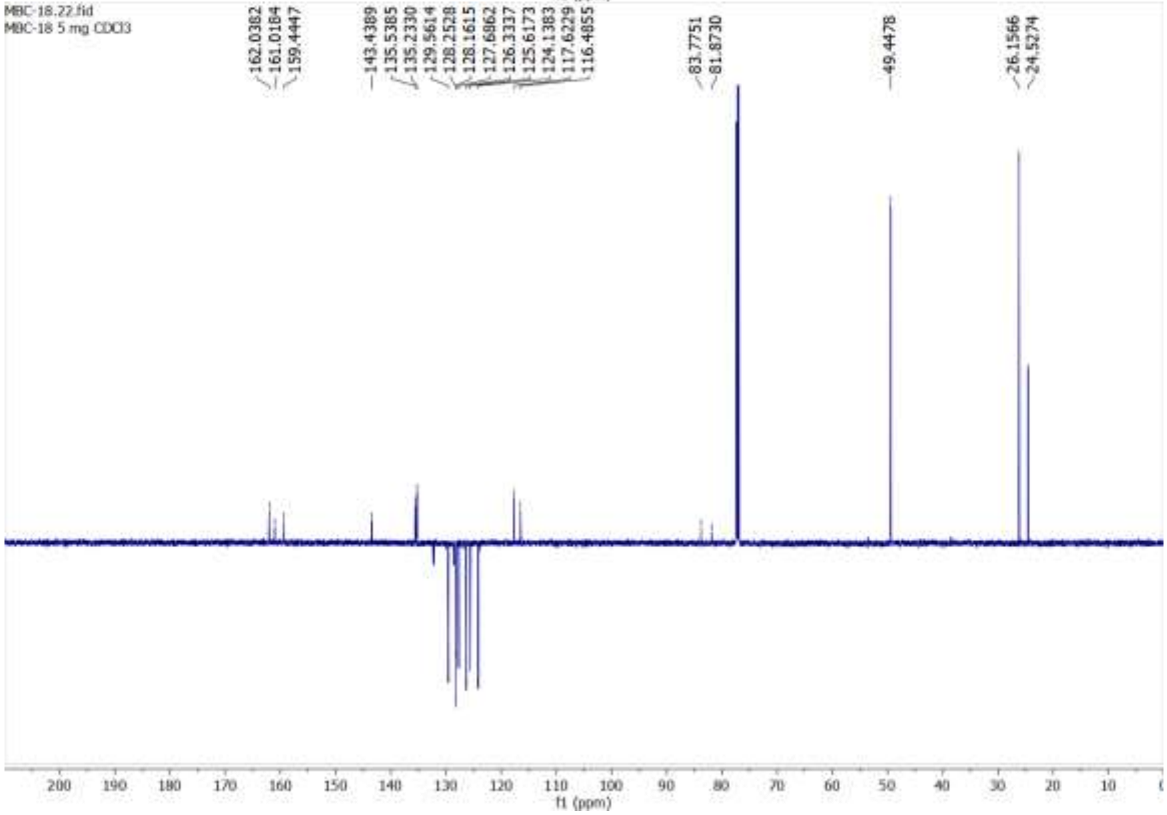
MBC-18.22.fid
MBC-18 5 mg CDCl3
162.0382 161.0184 159.4447 143.4389 135.5385 135.2330 129.5614 128.2528 128.1615 127.6862 126.3337 125.6173 124.1383 117.6229 116.4855
83.7751 81.8730
49.4478
26.1566 24.5274
f1 (ppm)

Compound 48:

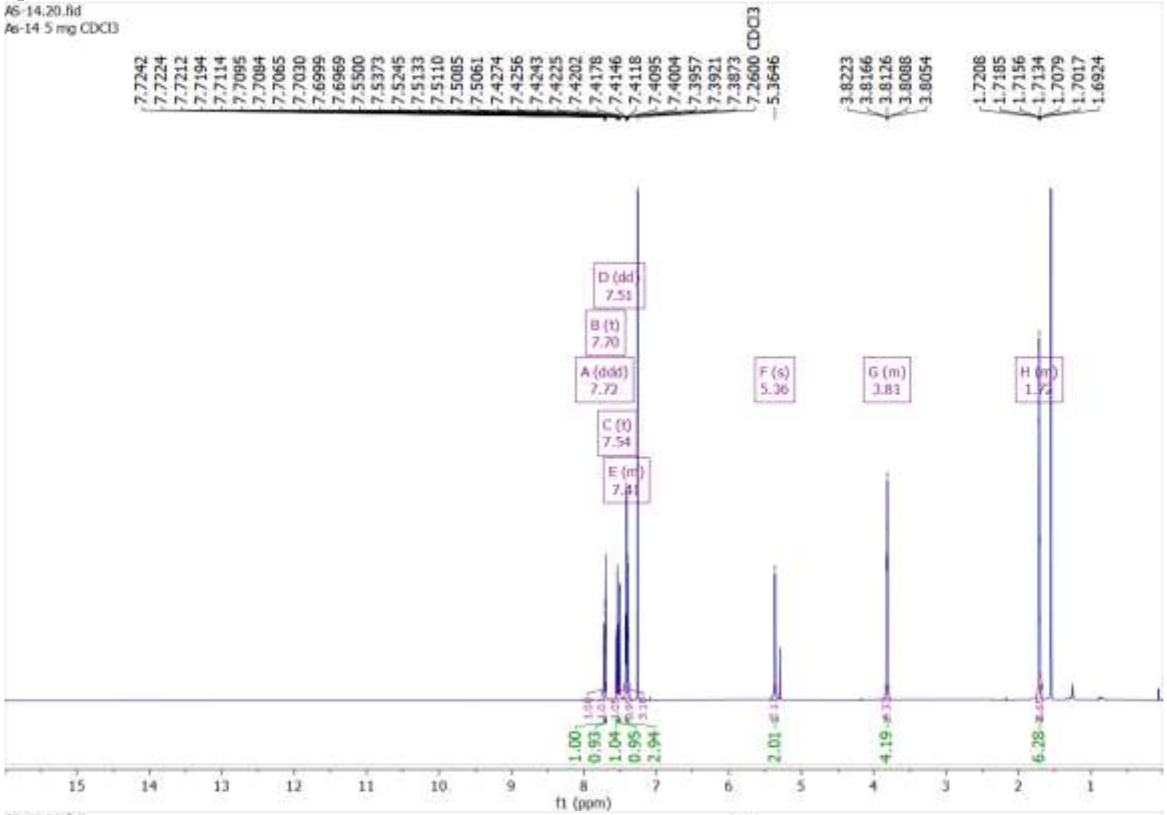
AS-14.20.fid
As-14 5 mg CDCl3
7.7242
7.7224
7.7212
7.7194
7.7114
7.7095
7.7084
7.7065
7.7030
7.6999
7.6969
7.5500
7.5373
7.5245
7.5133
7.5110
7.5085
7.5061
7.4274
7.4256
7.4243
7.4225
7.4202
7.4178
7.4146
7.4118
7.4095
7.4004
7.3957
7.3921
7.3873
7.2600 CDCl3
5.3646
3.8223
3.8166
3.8126
3.8088
3.8054
1.7208
1.7185
1.7156
1.7134
1.7079
1.7017
1.6924
D (dd)
7.51
B (t)
7.70
A (ddd)
7.72
C (t)
7.54
E (m)
7.41
F (s)
5.36
G (m)
3.81
H (m)
1.72
1.00
0.93
1.04
0.95
2.94
2.01
4.19
6.28
f1 (ppm)

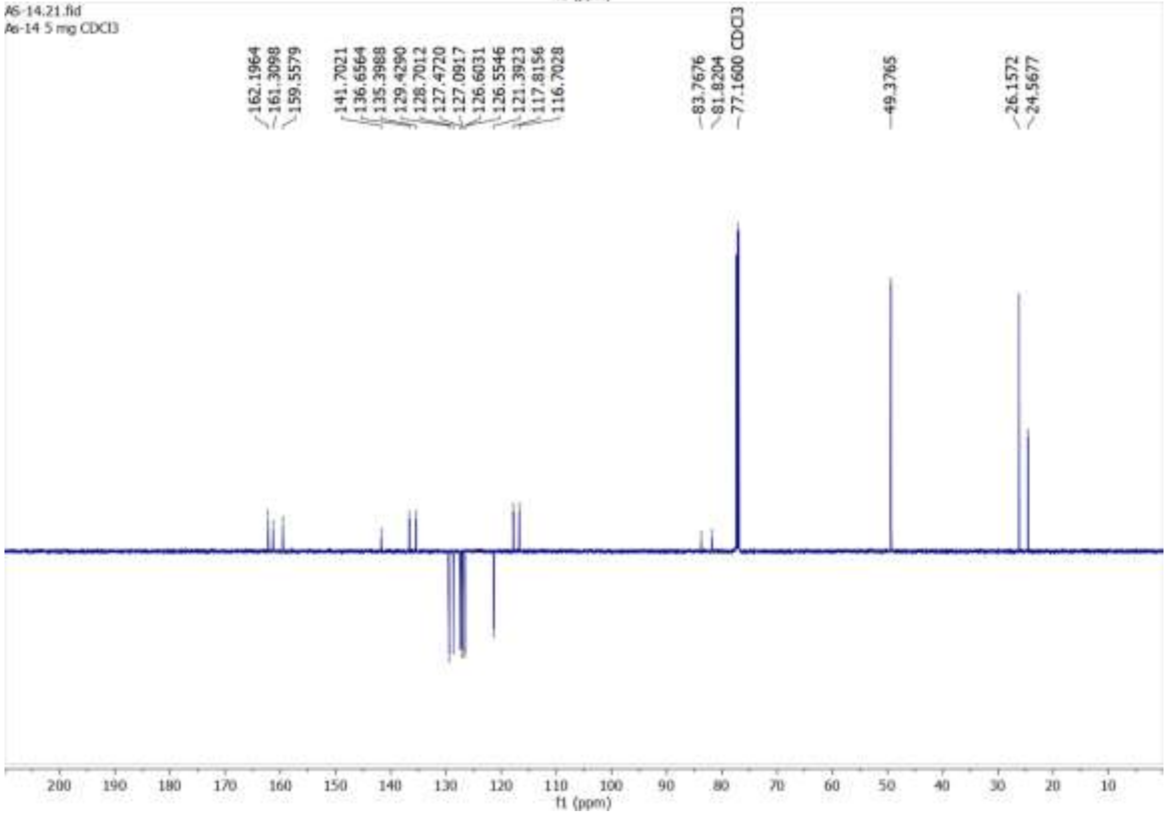
AS-14.21.fid
As-14 5 mg CDCl3
162.1964
161.3098
159.5579
141.7021
136.6564
135.3988
129.4290
128.7012
127.4720
127.0817
126.6031
126.5546
121.3923
117.8156
116.7028
83.7676
81.8204
77.1600 CDCl3
49.3765
26.1572
24.5677
f1 (ppm)

Compound 49:

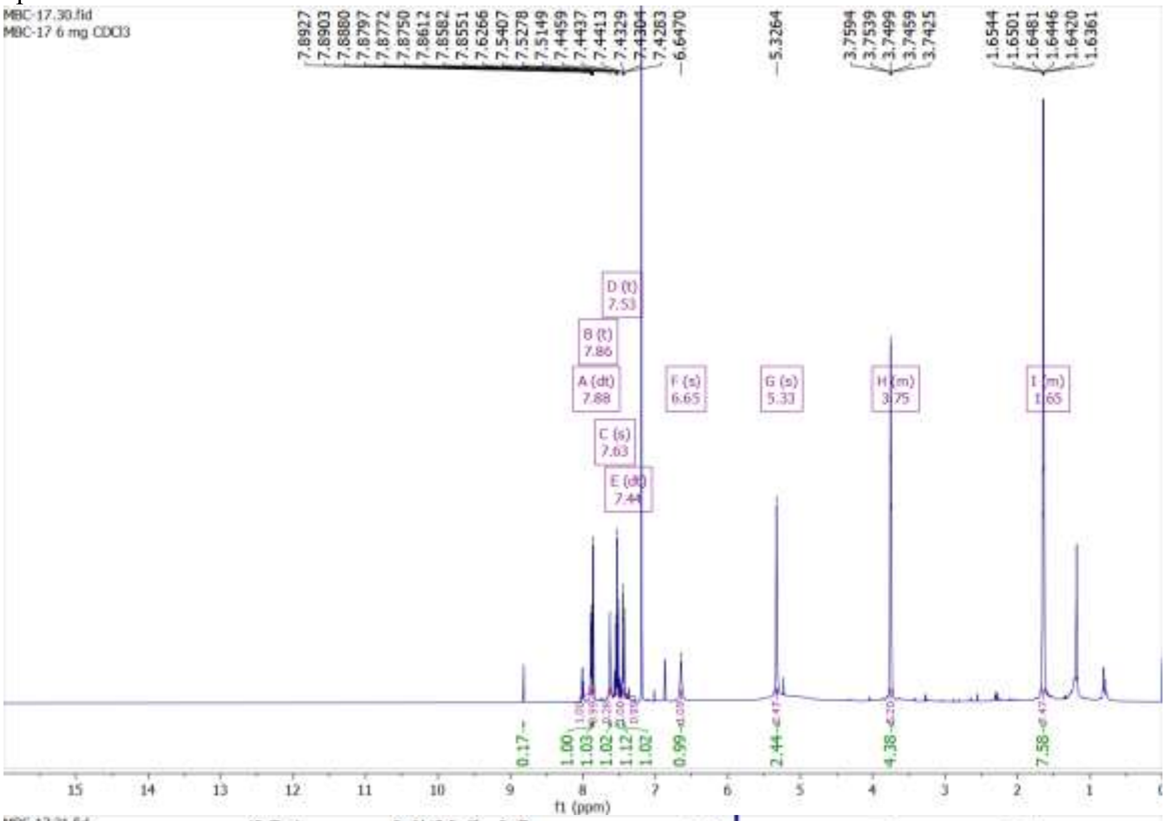
MBC-17.30.fid
MBC-17 6 mg CDCl3
7.8927
7.8903
7.8880
7.8797
7.8772
7.8750
7.8612
7.8582
7.8551
7.6266
7.5407
7.5278
7.5149
7.4459
7.4437
7.4413
7.4329
7.4304
7.4283
6.6470
5.3264
3.7594
3.7539
3.7499
3.7459
3.7425
1.6544
1.6501
1.6481
1.6446
1.6420
1.6361
D (t)
7.53
B (t)
7.86
A (dt)
7.88
C (s)
7.63
E (dt)
7.44
F (s)
6.65
G (s)
5.33
H (m)
3.75
I (m)
1.65
0.17
1.00
1.03
1.02
1.12
1.02
0.99
2.44
4.38
7.58
f1 (ppm)

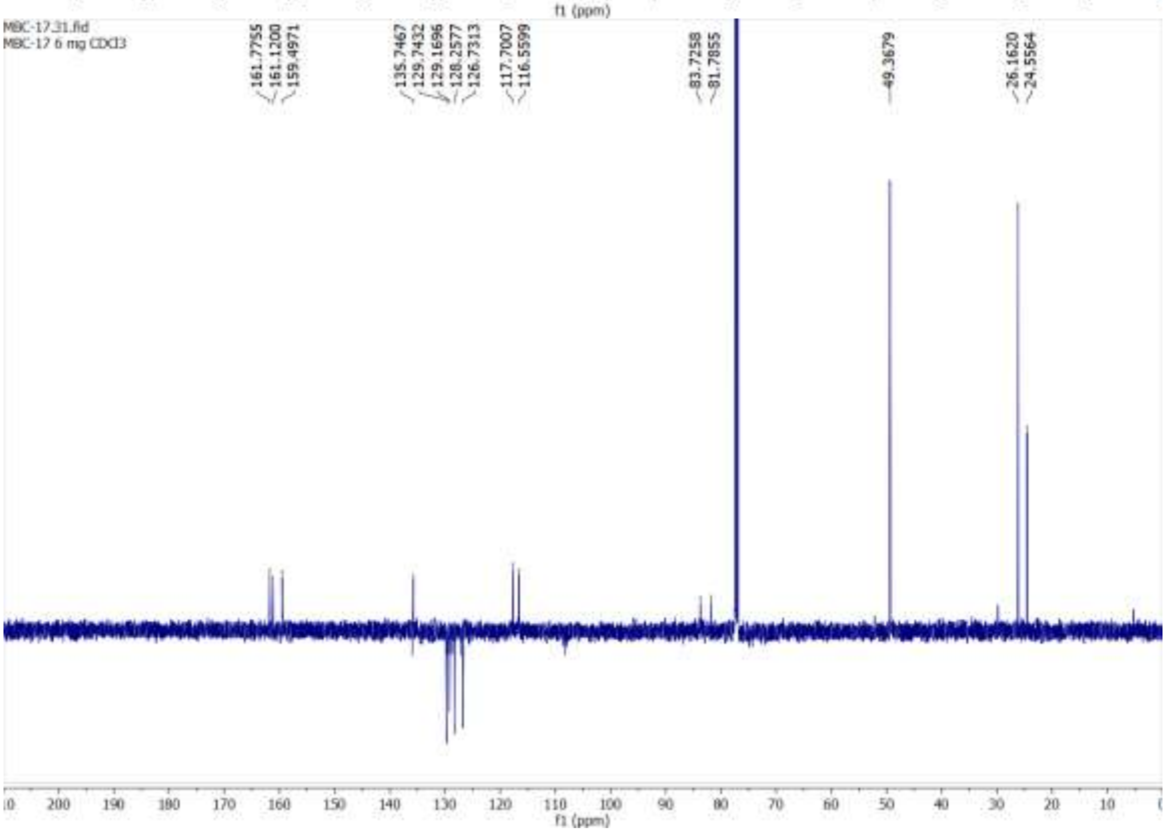
MBC-17.31.fid
MBC-17 6 mg CDCl3
161.7755
161.1200
159.4971
135.7467
129.7432
129.1696
128.2577
126.7313
117.7007
116.5599
83.7258
81.7855
49.3679
26.1620
24.5564
f1 (ppm)

Compound 50:

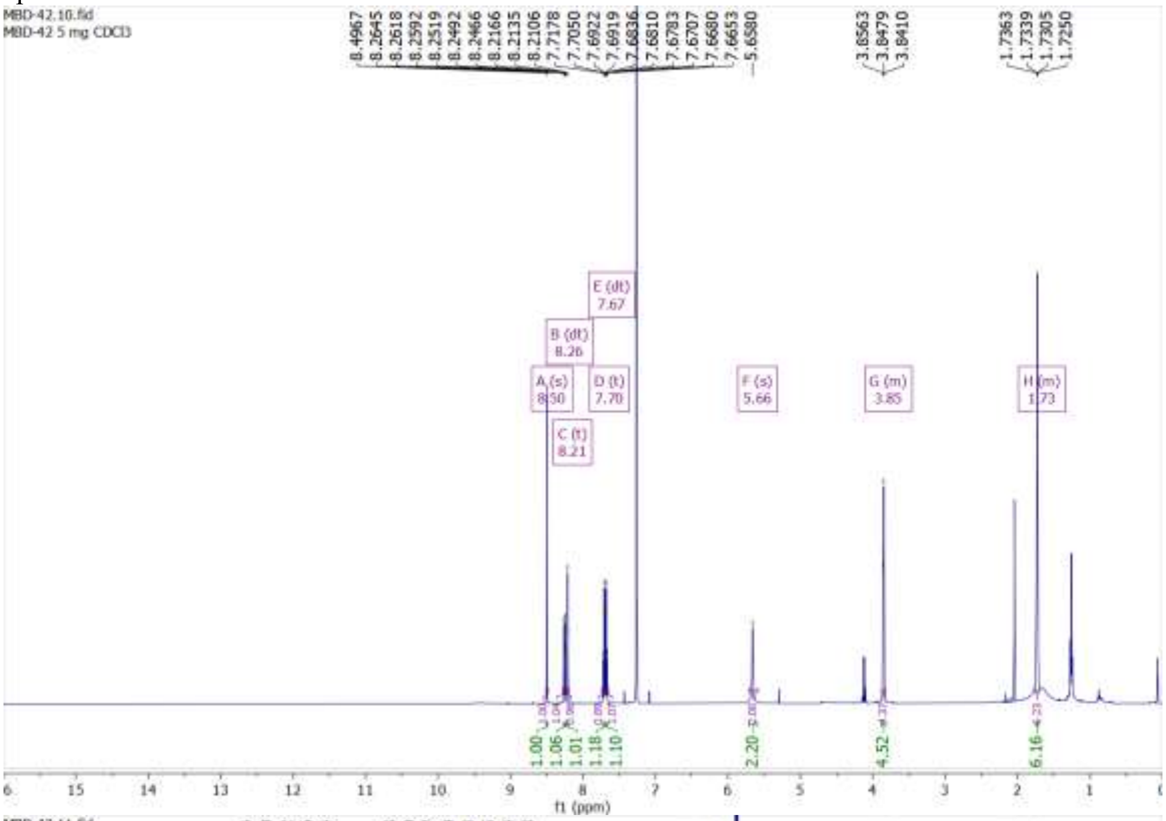
MBD-42.10.fid
MBD-42 5 mg CDCl3
8.4967
8.2645
8.2618
8.2592
8.2519
8.2492
8.2466
8.2166
8.2135
8.2106
7.7178
7.7050
7.6922
7.6919
7.6836
7.6810
7.6783
7.6707
7.6680
7.6653
5.6580
3.8563
3.8479
3.8410
1.7363
1.7339
1.7305
1.7250
E (dt) 7.67
B (dt) 8.26
A (s) 8.50
D (t) 7.70
C (t) 8.21
F (s) 5.66
G (m) 3.85
H (m) 1.73
1.00
1.06
1.01
1.18
1.10
2.20
4.52
6.16
f1 (ppm)

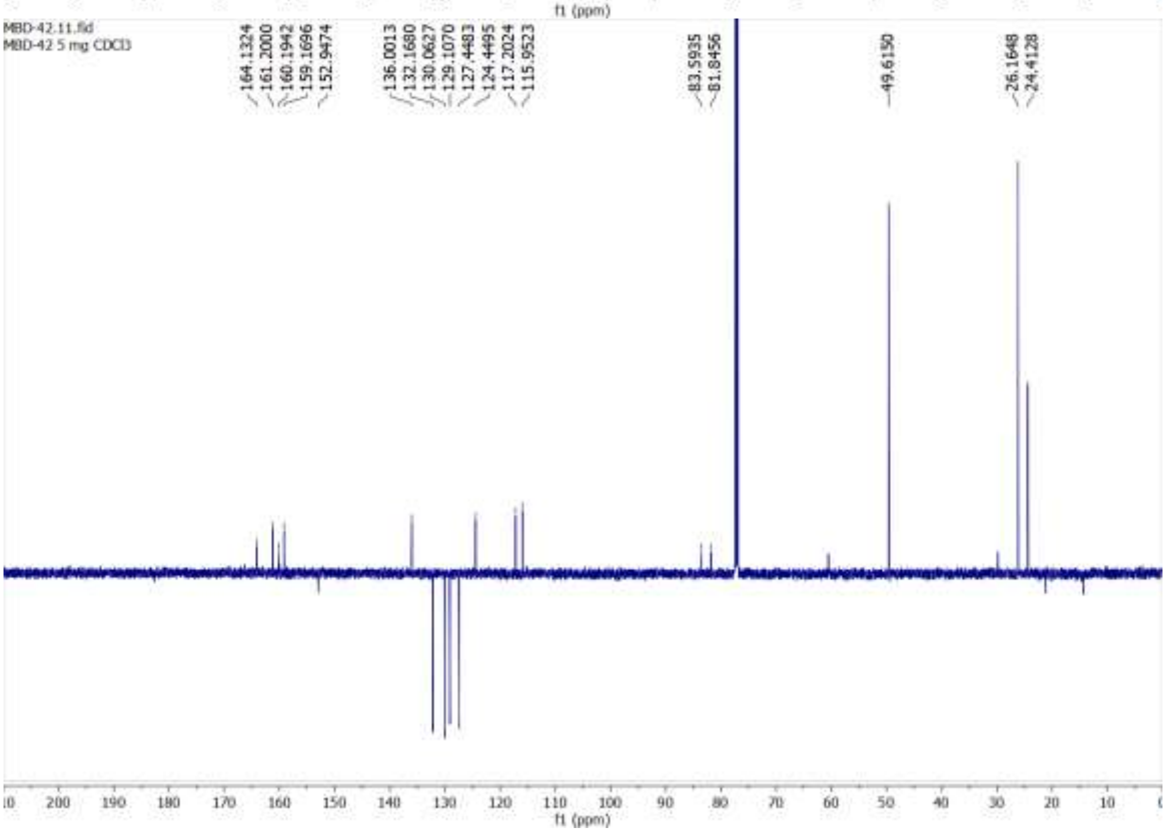
MBD-42.11.fid
MBD-42 5 mg CDCl3
164.1324
161.2000
160.1942
159.1696
152.9474
136.0013
132.1680
130.0627
129.1070
127.4483
124.4495
117.2024
115.9523
83.5935
81.8456
49.6150
26.1648
24.4128
f1 (ppm)

Compound 51:

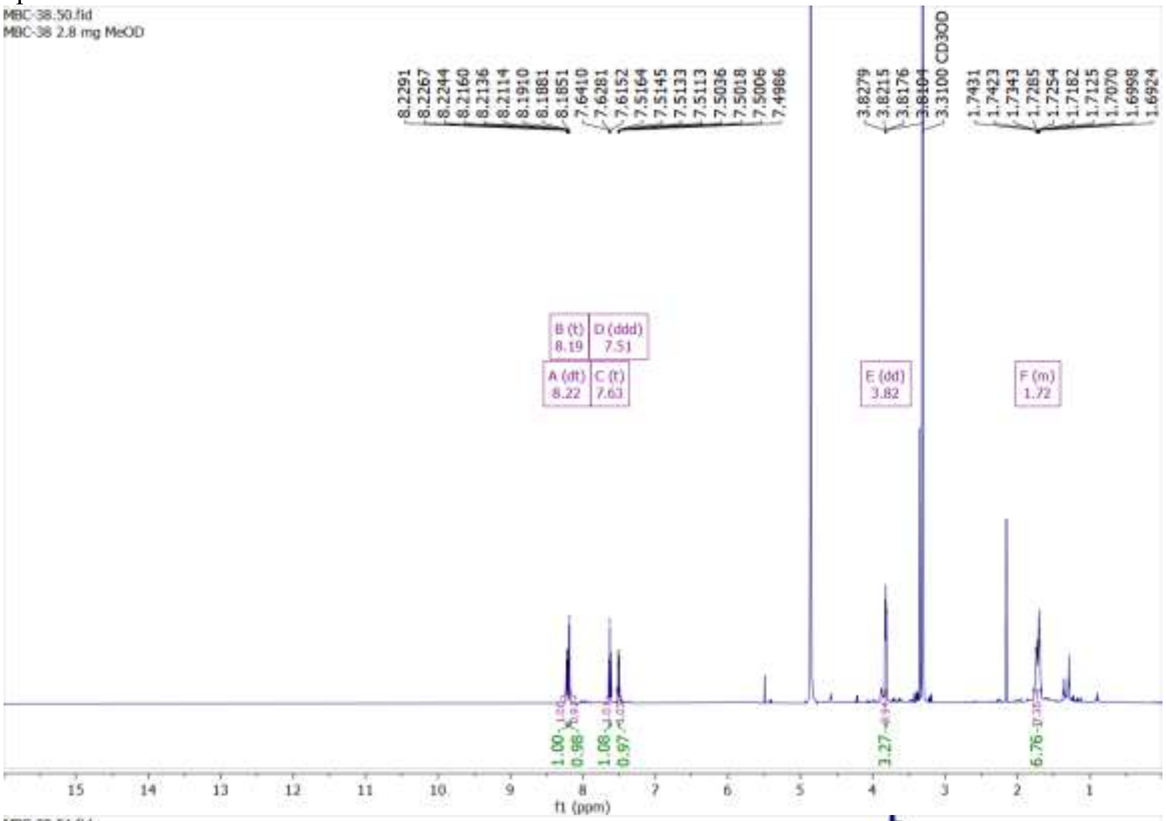

MBC-38.50.fid
MBC-38 2.8 mg MeOD
8.2291
8.2267
8.2244
8.2160
8.2136
8.2114
8.1910
8.1881
8.1851
7.6410
7.6281
7.6152
7.5164
7.5145
7.5133
7.5113
7.5036
7.5018
7.5006
7.4986
3.8279
3.8215
3.8176
3.8104
3.3100 CD3OD
1.7431
1.7423
1.7343
1.7285
1.7254
1.7182
1.7125
1.7070
1.6998
1.6924
B (t) 8.19
D (ddd) 7.51
A (dt) 8.22
C (t) 7.63
E (dd) 3.82
F (m) 1.72
1.00
0.98
1.08
0.97
3.27
6.76
f1 (ppm)

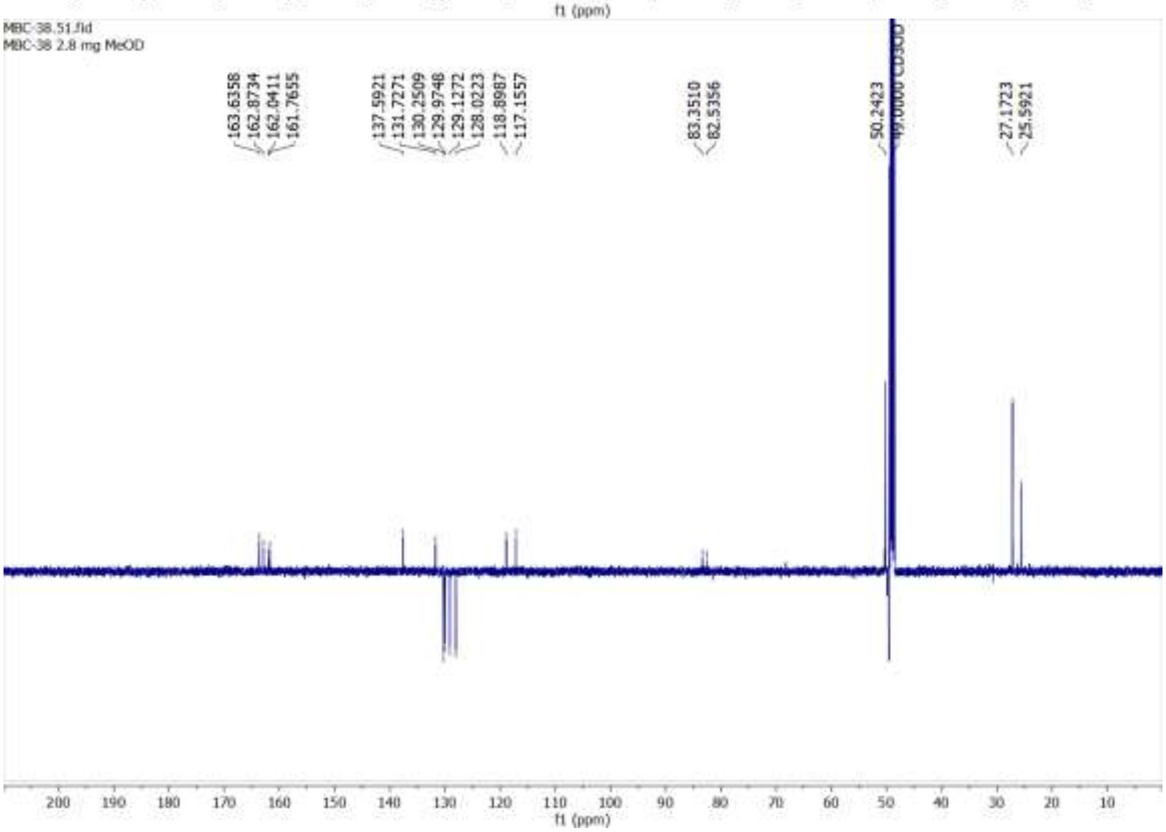

MBC-38.51.fid
MBC-38 2.8 mg MeOD
163.6358
162.8734
162.0411
161.7655
137.5921
131.7271
130.2509
129.9748
129.1272
128.0223
118.8987
117.1557
83.3510
82.5356
50.2423
49.0000 CD3OD
27.1723
25.5921
f1 (ppm)

References

(1) Gao, Z.-G.; Ijzerman, A. P. Allosteric Modulation of A2A Adenosine Receptors by Amiloride Analogues and Sodium Ions. *Biochemical Pharmacology* **2000**, *60* (5), 669–676. https://doi.org/10.1016/S0006-2952(00)00360-9.

(2) Lu, Y.; Liu, H.; Yang, D.; Zhong, L.; Xin, Y.; Zhao, S.; Wang, M.-W.; Zhou, Q.; Shui, W. Affinity Mass Spectrometry-Based Fragment Screening Identified a New Negative Allosteric Modulator of the Adenosine A2A Receptor Targeting the Sodium Ion Pocket. *ACS Chem. Biol.* **2021**, *16* (6), 991–1002. https://doi.org/10.1021/acschembio.0c00899.

(3) Jacobson, K. A.; Ukena, D.; Kirk, K. L.; Daly, J. W. [3H]Xanthine Amine Congener of 1,3-Dipropyl-8-Phenylxanthine: An Antagonist Radioligand for Adenosine Receptors. *Proc. Natl. Acad. Sci. USA* **1986**, *83*, 4089–4093. https://doi.org/10.1073/pnas.83.11.4089.

(4) Doré, A. S.; Robertson, N.; Errey, J. C.; Ng, I.; Hollenstein, K.; Tehan, B.; Hurrell, E.; Bennett, K.; Congreve, M.; Magnani, F.; Tate, C. G.; Weir, M.; Marshall, F. H. Structure of the Adenosine A2A Receptor in Complex with ZM241385 and the Xanthines XAC and Caffeine. *Structure* **2011**, *19* (9), 1283–1293. https://doi.org/10.1016/j.str.2011.06.014.
